# Catalyst-Controlled, Regiodivergent Aminooxygenation Reactions of Dienes

**DOI:** 10.1021/acs.joc.5c02010

**Published:** 2025-12-22

**Authors:** Caitlyn P. McNichol, Liván Borrego, Erhan Ertekin, Shauna M. Paradine

**Affiliations:** Department of Chemistry, 6927 University of Rochester, 120 Trustee Rd, Rochester, New York 14627, United States

## Abstract

We report a method for an aerobic, regiodivergent aminooxygenation of dienes via catalyst control. A simple switch in the copper catalyst source allows for selective access to either the 1,2- or 1,4-aminooxygenated product from a single starting material. Mechanistic studies indicate that the coordinating ligand on the copper salt is responsible for the divergent regiochemical outcomes and that the origin of regiodivergence lies in competing mechanisms for oxygen delivery to the carbon-centered radical intermediate.

## Graphical abstract

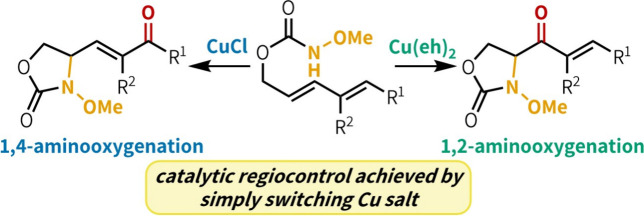

Conjugated 1,3-dienes are versatile substrates for transition metal catalysis due to their abundance and range of reactivity, although the numerous plausible regiochemical outcomes often lead to selectivity challenges in diene difunctionalization reactions (Figure 1A).^1^ Substrate control is a common approach to achieving regiocontrol, but this approach inherently limits structural diversity in product libraries and can require the pre-installation of functional groups that will later need to be removed. In contrast, catalyst control enables selective access to one or more regioisomeric products from unbiased substrates (Figure 1B); this is typically achieved by enhancing substrate bias or inducing preference for a single regioisomeric product.^2,3^ Here, accessing a different regioisomer is only possible through entirely distinct catalytic systems.^4^ A less common, although desirable, strategy entails switching ligands to selectively access two regioisomeric products from a unified set of reaction conditions and a single starting material.^5^ This approach has been used in reductive copper catalysis^6^ (Figure 1B), but there are no examples of this approach being applied under oxidative copper catalysis.

**Figure 1:**
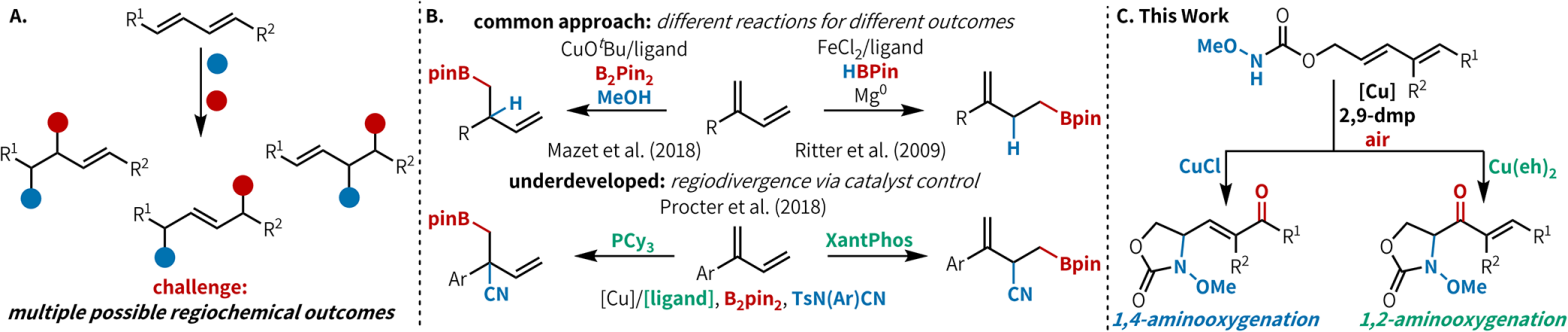
Regioselectivity in diene difunctionalization reactions. (A) Challenge: multiple possible regiochemical outcomes in diene difunctionalization reactions. (B) Approaches to catalytic regiocontrol, with selected examples. (C) This work: regiodivergent, catalyst-controlled aerobic aminooxygenation enables selective access to 1,4- and 1,2-aminooxygenated products, which are useful synthetic intermediates.

The aminooxygenation of olefins allows for the rapid construction of functionally rich organic scaffolds. While traditional aminooxygenation methods typically require precious metal catalysts and/or stoichiometric oxidants,^7^ aerobic aminooxygenation uses abundant molecular oxygen as both the terminal oxidant and the source of oxygen functionality.^8^ Our lab recently reported a substrate-promoted, copper-catalyzed aerobic aminooxygenation reaction that operated under ambient air conditions and engaged internal alkene substrates.^9^ While aminooxygenation reactions of corresponding dienes are known,^2b^ hydroamination is the most common aminofunctionalization reaction of dienes.^11^ Here, we report that our aerobic aminooxygenation method can be applied to dienes^12^ via a catalyst-controlled, regiodivergent intramolecular difunctionalization of dienyl carbamates under mild and aerobic conditions to selectively access both 1,4- and 1,2-aminooxygenated products (Figure 1C). Both product classes map onto compounds bearing aminoalcohol motifs that, for example, serve as ligand backbones^13^ or bioactive molecules.^14^

Two nearly identical sets of conditions were identified for this transformation using dienyl carbamate **1a** as the model substrate (Table 1). Conditions **A**, using copper­(I) chloride (CuCl) and 2,9-dimethylphenanthroline (2,9-dmp), favored 1,4-aminooxygenated product **2a** (entry 1). With copper­(II) 2-ethylhexanoate (Cu­(eh)_2_), i.e., conditions **B**, the regioselectivity switched to favor 1,2-aminooxygenation product **2a′** (entry 6). The oxidation state of the copper salt did not substantially alter the regiochemical outcome (entries 1–2, 7–8). In contrast, the coordinating ligand on copper had a significant impact. Copper halides favored **2a** (entries 1–4), whereas copper carboxylates favored **2a′** (entries 6–8).^15^ As a control, we performed the reaction under conditions **A** with the addition of AgOAc, which switched the regioselectivity to favor **2a′** (entry 5, 19:81 vs 90:10 **2a**/**2a′**). Likewise, performing the reaction under conditions **B** with TBACl (tetrabutylammonium chloride) switched the regioselectivity to favor **2a** (entry 9, > 99:1 vs 8:92 **2a**/**2a′**). Together, these results suggest that the coordinating ligand on copper plays the primary determining role in the regiochemical outcome.

**Table 1:**
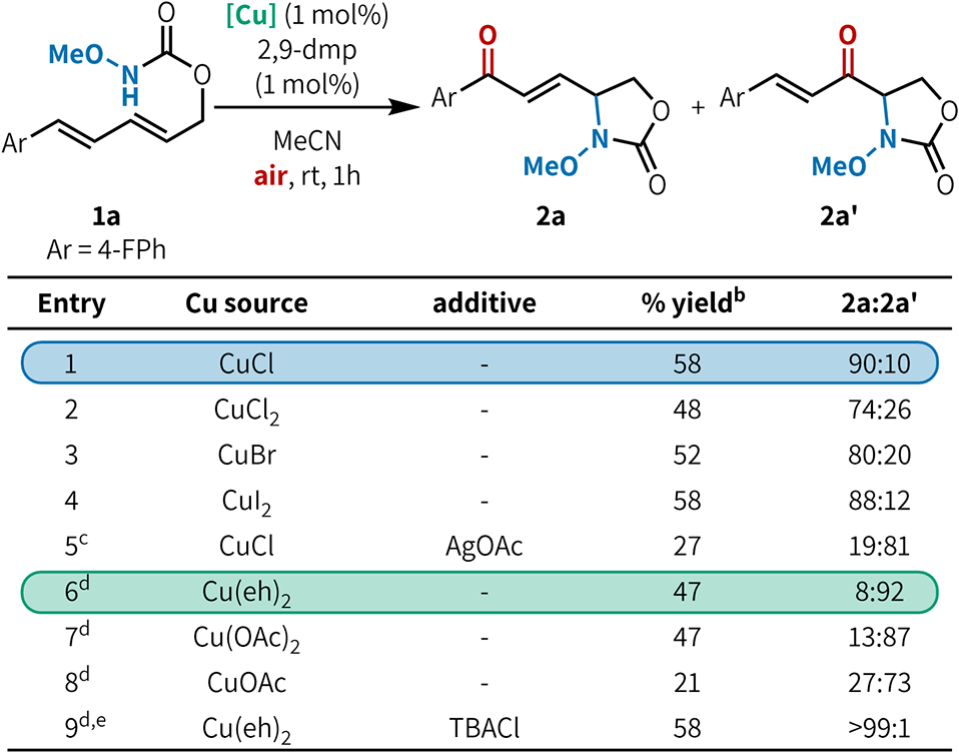
Effect of the Cu Source on the Regiochemical Outcome in Aerobic Aminooxygenation of Dienes^a^

a Reaction conditions: **1a** (0.05 mmol), [Cu] (0.5 μmol), 2,9-dmp (0.5 μmol), MeCN (0.2M), rt, air, 1 h. 2,9-dmp = 2,9-dimethylphenanthroline.

b Yields were determined by GC analysis using anisole as the internal standard (**2a** + **2a′**) and are an average of two runs.

c 50 mol % AgOAc added.

d 10 mol % [Cu] and 10 mol % 2,9-dmp used.

e 50 mol % TBACl added.

Using both sets of optimized conditions,^15^ we investigated reactivity trends in these reactions (Figure 2). Under conditions **A**, **1a** afforded the aminooxygenated product in 56% yields and 89:11 **2a**/**2a′**. The same substrate was subjected to conditions **B** to afford primarily the 1,2-aminooxygenated product (56%, 22:78 **2a**/**2a′**). Under conditions **A**, aromatic substrates all proceeded efficiently (50–58%) with good regioselectivity favoring 1,4-aminooxygenated products **2b**–**e** (generally >80:20 **2**/**2′**). With conditions **B**, these same substrates favored 1,2-aminooxygenated products **2b′–d′** in moderate yields (34–56%) and moderate to good regioselectivity, except for electron-poor aromatic substitution (46:54 **2e/2e′**). Under both conditions, the reaction could be scaled to 1.5 mmol with comparable results (**1b**). With a substrate bearing an ortho-methyl substituent, the product was obtained with modest but opposite regioselectivity under conditions **A** and **B** (67:33 and 34:66 **2f**/**2f′**, respectively). When substitution was introduced at the C3 position of the diene (**1g**, **1h**), both conditions **A** and **B** converged to the 1,4-aminooxygenated products (**2g** and **2h**, respectively) with excellent selectivity (>99:1). The diene C4 dimethylated substrate was unreactive (**1i**), while the monomethylated substrate (**1j**) afforded products with >99:1 **2j**/**2j′** selectivity under both reaction conditions. Substrates bearing 2-substituted heterocycles favored 1,2-aminooxygenation under conditions **A** and **B** (**1k**, **1l**).

**Figure 2:**
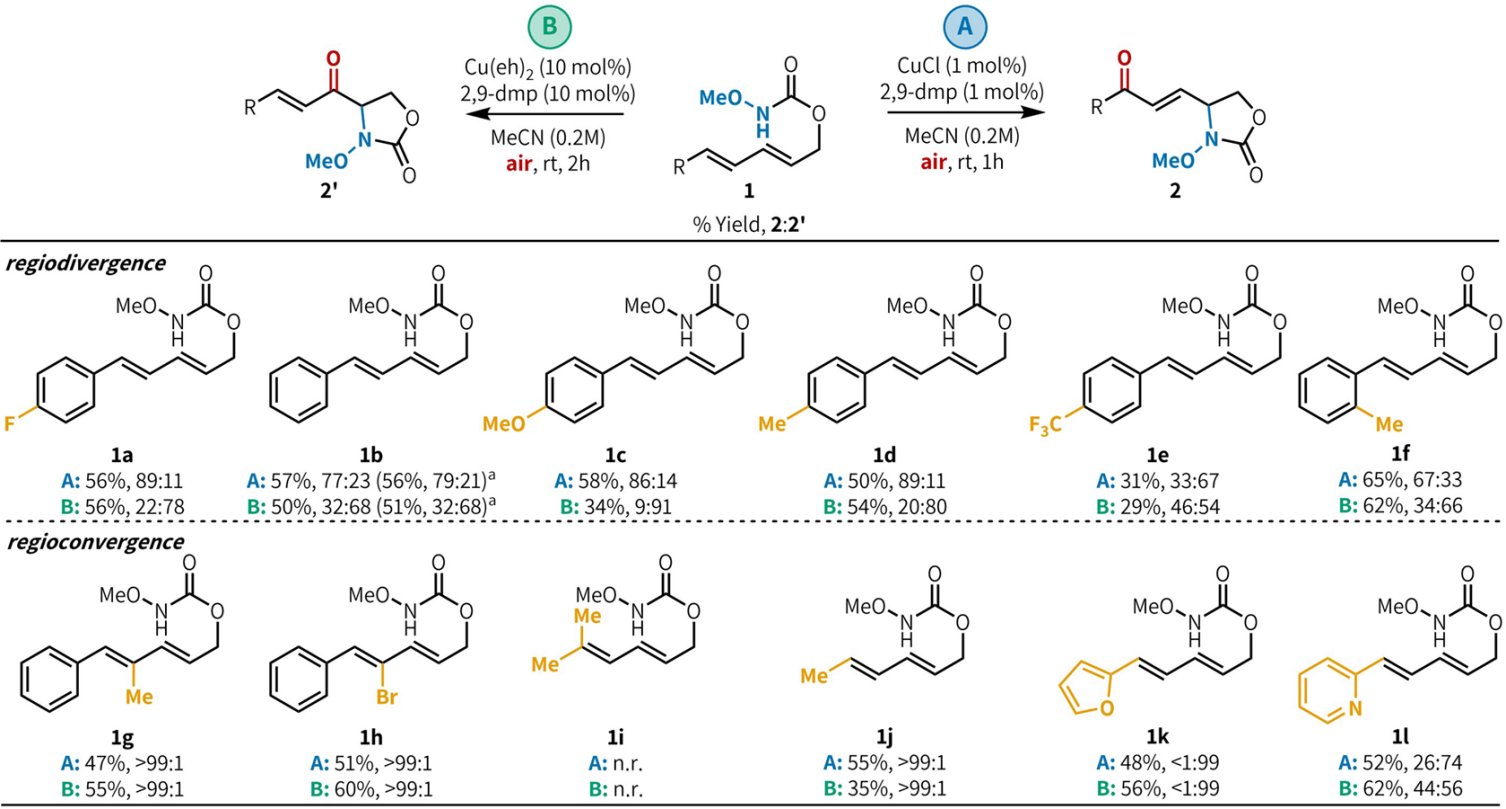
Substrate scope. Reported yields (**2** + **2′**) are an average of three runs. Regioselectivity was determined by ^1^H NMR of crude reaction mixtures. Conditions **A**: CuCl (5.0 μmol), 2,9-dmp (5.0 μmol), **1** (0.5 mmol), MeCN (0.2M), air, rt, 1 h. Conditions **B**: Cu­(eh)_2_ (0.03 mmol), dmp (0.03 mmol), **1** (0.3 mmol), MeCN (0.2M), air, rt, 2 h. ^a^ Reactions in parentheses were run at a 1.5 mmol scale.

To gain further insight into the source of regiodivergence in these reactions, we conducted a series of mechanistic experiments. When either product **2a** or **2a′** was subjected to reaction conditions that favored the opposite product,^15^ no interconversion between **2a** and **2a′** occurred, and the products were recovered quantitatively, meaning regioselectivity is not determined by product equilibrium and that oxidative cleavage does not occur from overoxidation of the ketone product. Under reaction conditions **B**, cinnamaldehydes were obtained as significant byproducts, resulting from Cu-promoted oxidative cleavage,^16^ while under conditions **A**, no aldehyde byproducts were observed. Likewise, when the nitrogen of the carbamate was methylated, only the starting material was recovered, ruling out the possibility that the oxidative cleavage pathway diverges prior to cyclization. These results are consistent with distinct mechanisms for oxygen delivery under conditions **A** and **B**. Kinetic experiments illustrate differences in the chemoselectivity of conditions **A** and **B** (Figure 3A), with only trace amounts of **3a** observed under conditions **A** but significant amounts of **3a** observed under conditions **B**. Additionally, there are changes in both **2a**/**2a′** and (**2a** + **2a′**)/**3a** selectivities as the reactions progress, consistent with changes in the dominant catalytic species over the course of the reaction. This is also consistent with our previous observations (Table 1, entries 1, 5, 6, and 9), indicating that the relative concentration of the halide vs carboxylate anion in solution significantly impacts the selectivity of **2a**/**2a′**. On the basis of these experiments as well as substantial literature on differential speciation of copper–oxygen complexes,^17^ we hypothesize that the observed regiodivergence arises from competing mechanisms for oxygen delivery resulting from differently speciated copper–oxygen complexes (Figure 3B); this phenomenon has been previously observed in aerobic, Cu-catalyzed C–H oxidation reactions.^17a^

**Figure 3:**
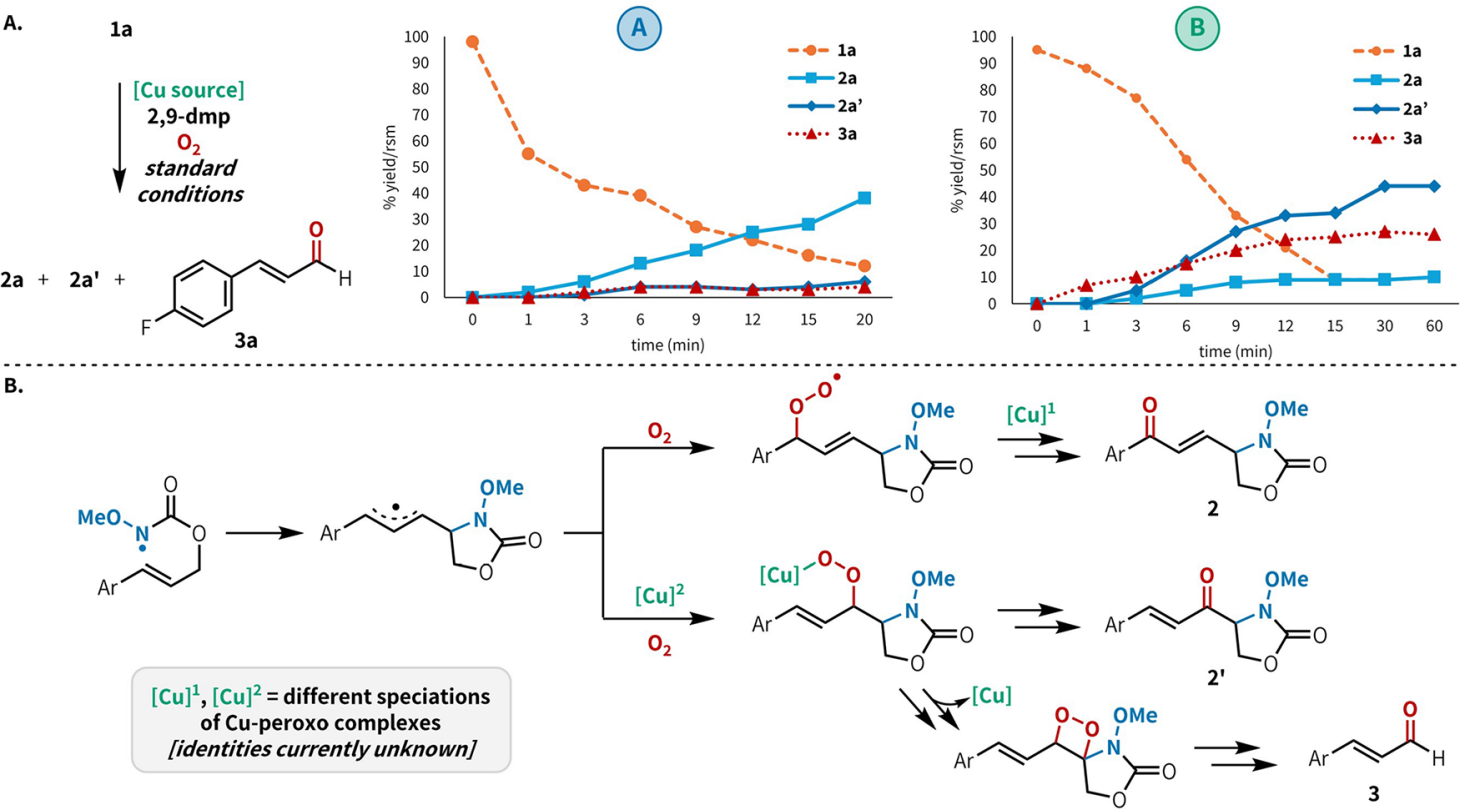
Mechanistic analysis. (A) Reaction time course plots under standard conditions **A** and **B**. (B) Mechanistic proposal for regiodivergence arising from differences in the speciation of Cu–oxygen complexes.

We have established a catalyst-controlled, regiodivergent aminooxygenation reaction of diene-tethered carbamates.^18^ Our mechanistic investigations reveal a mechanism consistent with a Cu-promoted amidyl radical cyclization step followed by an oxygenation step occurring via one of two pathways, selectively leading to regioisomeric products depending on the catalyst source used. Through this method, we can selectively generate two distinctly substituted libraries of functionalized oxazolidinone motifs from a single starting material. Future studies will focus on further investigation into the mechanism of oxygen delivery and identifying relevant catalytic species as well as leveraging our mechanistic insights for the expansion of our catalyst-controlled, regiodivergent methods.

## Supplementary Material



## Data Availability

The data underlying this study are available in the published article and its Supporting Information.

## Notes

Additional experimental details, experimental procedures, compound characterization, NMR spectra for all new compounds, single-crystal X-ray data (PDF)
