# Supplementary material for: Catalyst-Controlled, Regiodivergent Aminooxygenation Reactions of Dienes
Source: J Org Chem. 2025 Dec 22;91(1):749–53. doi: 10.1021/acs.joc.5c02010 (PMC12797283; doi:10.1021/acs.joc.5c02010)

Supporting Information
Catalyst-Controlled Regiodivergent Aminooxygenation Reactions of Dienes

Caitlyn P. McNichol, Liván Borrego, Erhan Ertekin, and Shauna M. Paradine*
Department of Chemistry, University of Rochester, 120 Trustee Road, Rochester, NY 14627 (USA)
*Corresponding Author: sparadin@ur.rochester.edu

Contents

General Information....S2
Methods....S2
Materials and Reagents....S2
Instrumentation....S2
Abbreviations Used....S3
Summary of 2,4-dien-1-yl *N*-methoxycarbamates used....S4
Preparation of 2,4-dien-1-yl *N*-methoxycarbamates....S5
Synthesis from cinnamaldehyde derivatives....S5
Reaction Investigations....S13
Copper Source Studies....S13
Ligand Studies....S17
Base and Solvent Screening....S23
Counterion Control Experiments....S33
Reaction Kinetics Experiments....S34
Product Interconversion Experiments....S37
Preparation of Precatalysts....S38
UV/Vis Spectra of Reaction Components and Kinetic Data....S40
Substrate Scope....S43
Reaction Conditions A....S43
Reaction Conditions B....S51
Scaled Reaction....S59
Methodology Limitations....S60
References Cited....S60
1H NMR, 13C NMR, and 19F NMR Spectra for New Compounds....S60

General Information

Methods

All reactions were carried out under a nitrogen atmosphere in flame–dried glassware with magnetic stir bar unless otherwise specified. Stainless steel gas-tight syringes were used to transfer air- and moisture-sensitive liquids. Reactions were monitored by thin–layer chromatography (TLC) on pre–coated silica gel 60 F254 glass–supported plates from EMD and visualized under UV light (254 nm) or with p–anisaldehyde followed by heating. Flash chromatography was performed using SiliaFlash P60 (230–400 mesh, SiliCycle). Reported product yields were determined based on material isolated after column purification. Room temperature (rt) for the laboratory is 20 °C.

Materials and Reagents

Reagents were used as obtained from commercial suppliers without further purification unless otherwise noted. Copper salts were purchased from Strem and stored in a glovebox; 1,10-phenanthroline and 2,2-bipyrdiyl based ligands were used without purification and stored in a glovebox. Reaction solvents – Tetrahydrofuran (THF), acetonitrile (MeCN), dichloromethane (DCM) and methanol (MeOH) – were purchased from Fisher and dried by passing through columns of activated alumina (Pure Process Technology SPS). *N,N*-Dimethylformamide (DMF) (Fisher) was stored over 3Å molecular sieves. Anhydrous dimethylacetamide (DMAc) was purchased from Aldrich. Deuterated solvents $CDCl_3$, and DMSO-d_6 (Cambridge Isotope Laboratories), and HPLC solvents (Fisher) were used without further purification.

Instrumentation

Proton nuclear magnetic resonance (^{1}H NMR) and proton-decoupled carbon nuclear magnetic resonance (^{13}C, ^{1}H NMR) spectra were recorded on a Bruker DPX–400 instrument (operating at 400 MHz for ^{1}H, 100 MHz for ^{13}C) or a Bruker DPX–500 instrument (operating at 500 MHz for ^{1}H, 125 MHz for ^{13}C) at ambient temperature. Proton resonances are referenced to residual protium in the NMR solvent. Carbon resonances are referenced to the carbon resonances of the NMR solvent. Data are represented as follows: chemical shift, multiplicity (br = broad, s = singlet, d = doublet, t = triplet, q = quartet, dd = doublet of doublets, dt = doublet of triplets, m = multiplet, app = apparent), coupling constants (J) in Hertz (Hz), and integration. Mass spectrometry (MS) data were obtained on a Thermo Fisher Q Exactive Plus spectrometer (University of Rochester Medical Center Mass Spectrometry Resource Laboratory) using an Orbitrap mass analyzer. Infrared (IR) spectra were obtained using a Shimadzu IRAffinity-1 FTIR spectrometer equipped with an attenuated total reflectance (ATR) single reflection unit. High-performance liquid chromatography (HPLC) analysis was performed using a Shimadzu Prominence–I LC–2030 Plus system with commercially available Restek Pinnacle DB Cyano column (5 μm, 150x4.6 mm) or a Daicel CHIRALCEL® OD-H cellulose tris(3,5-dimethylphenylcarbamate) column (5 μm, 150x4.6 mm). UV-vis spectroscopy was conducted on a Shimadzu UV-2401PC spectrophotometer using a quartz cuvette with a 1.0 cm path length.

Abbreviations Used

aq. = aqueous, bpy = 2,2-bipyridyl, CDI = carbonyldiimidazole, cm = centimeter, DCM = dichloromethane, DIBAL-H = diisobutylaluminum hydride, DIPEA = *N,N*-diisopropylethylamine, DMF = *N,N*-dimethylformamide, dmp = 2,9-dimethyl-1,10-phenanthroline, d.r. = diastereomeric ratio, DTBP = di-*tert*-butyl peroxide, EDC = 1-ethyl-3-(-3-dimethylaminopropyl) carbodiimide hydrochloride, equiv. = equivalents, EtOAc = ethyl acetate, Et_2O = diethyl ether, gen = general, h = hours, min = min, mp = melting point, NaH= Sodium Hydride, NaOH= sodium hydroxide, rbf= round bottom flask, rpm = revolutions per minute, RSM = recovered starting material, rt = room temperature, sat = saturated, TBS = tertbutyldimethylsilyl, THF = tetrahydrofuran.

Summary of 2,4-dien-1-yl *N*-methoxycarbamates used

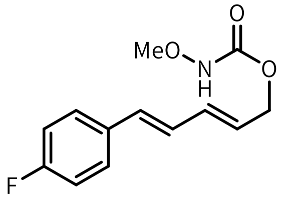

1a

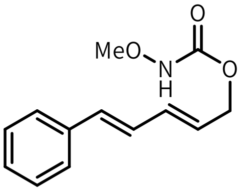

1b

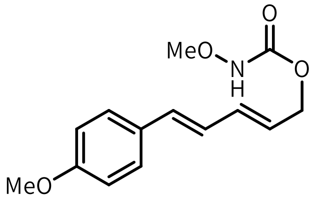

1c

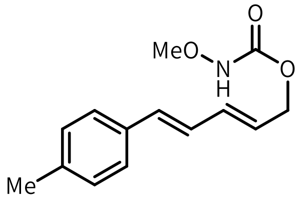

1d

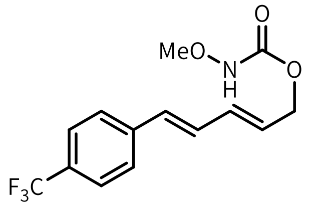

1e

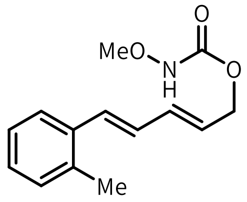

1f

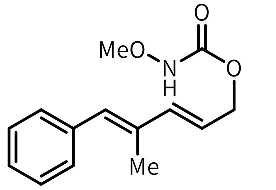

1g

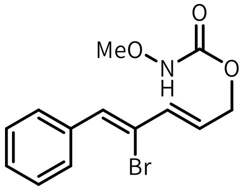

1h

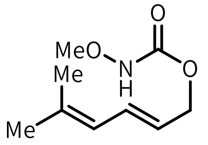

1i

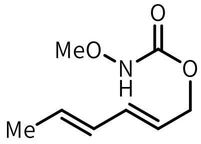

1j

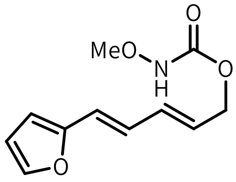

1k

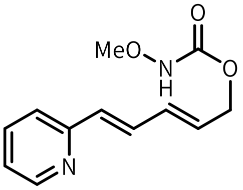

1l

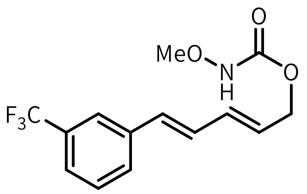

1m

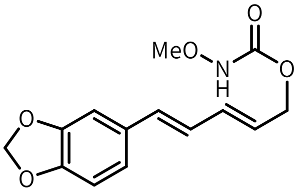

1n

Preparation of 2,4-dien-1-yl *N*-methoxycarbamates

Synthesis from cinnamaldehyde derivatives

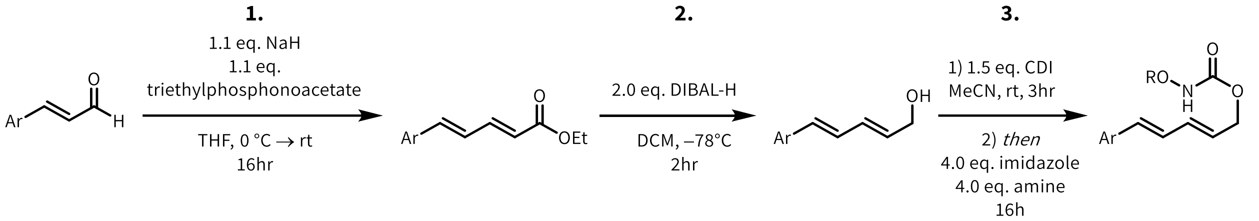

Standard Procedure:
1. Modified from a literature procedure.[1] To a suspension of NaH (60% in mineral oil, 1.1 equiv) in THF (0.2M) at 0 °C was added dropwise triethyl phosphonoacetate (1.1 eq.) and the reaction mixture was stirred for 30 min. A solution of aldehyde in THF (0.5M) was then added. The reaction mixture was allowed to warm to rt and was stirred overnight. The reaction was quenched with sat. aqueous NH_4Cl, the aqueous layer was extracted with EtOAc (2x) and the organic layer was washed with brine, dried over Na_2SO_4 and concentrated under reduced pressure to afford α,β-unsaturated ester **2.** Modified from a literature procedure.[1] Substituted ester was transferred to a 3 neck rbf, sealed, then purged and backfilled with argon 3 times. The solids were dissolved in dry DCM (0.2M) and cooled to –78 °C, followed by dropwise addition of DIBAL-H (2.2 equiv., 1M in PhMe) over 10 min. The reaction was stirred at –78 °C for 1h. Upon completion the reaction was quenched with 10% aq. NaOH (5 mL/mmol cinnamate ester). The biphasic mixture was stirred while warming to rt over 1h, then further at rt over another 1h as the emulsion subsides. The layers were separated, and the aq layer was extracted with DCM twice (5mL/mmol cinnamate ester). The organic layer was washed with brine, dried over Na_2SO_4, then concentrated in vacuo affording the crude cinnamyl alcohol which was used without further purification.
3. Modified from a literature procedure.[2] Substituted alcohol was taken up in MeCN (0.2M) in a rbf. CDI (1.5 equiv.) was added in one portion at rt. Upon consumption of the alcohol by TLC (1-3hr), methoxyamine HCl (4 equiv.) and imidazole (4 equiv.) were added, and the mixture was allowed to stir overnight (16h). The aqueous layer was extracted 3 times with EtOAc (5 mL/mmol alcohol). The organic layer was washed with brine, dried over Na_2SO_4 and concentrated in vacuo. The crude residue was purified by flash chromatography on SiO_2 eluting with EtOAc/hexanes mixture unless otherwise noted.

(2*E*,4*E*)-5-(4-fluorophenyl)penta-2,4-dien-1-yl methoxycarbamate (1a)

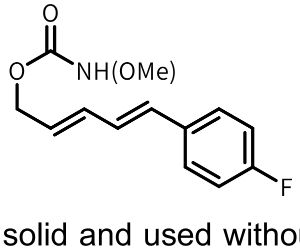

Prepared according to standard procedure: **1)** (*E*)-3-(4-fluorophenyl)acrylaldehyde (2.25 g, 15.0 mmol, 1.0 equiv.) THF (30.0 mL, 0.5M), NaH (0.72 g, 18.0 mmol, 1.2 equiv.), Triethylphosphonoacetate (3.6 mL, 18.0 mmol, 1,2 equiv.). **2)** DIBAL-H (1M in PhMe) (30.0 mL, 30.0 mmol, 2.0 equiv.), DCM (75.0 mL, 0.2M). Product with minor impurities was obtained as a solid and used without further purification. **3)** MeCN (75.0 mL, 0.2M), CDI (3.65 g, 22.5 mmol, 1.5 equiv.), imidazole (4.08 g, 60.0 mmol, 4.0 equiv.), methoxyamine HCl (5.01 g, 60.0 mmol, 4.0 equiv.). Crude material was purified by flash column chromatography

(SiO_2) using a gradient of 30% --> 50% EtOAc in hexanes to afford pure product as a white solid (2.36 g, 9.4 mmol, 63% over three steps).

^{1}H NMR (400 MHz, $CDCl_3$) δ 7.45 (s, 1H), 7.40 – 7.31 (m, 2H), 7.08 – 6.95 (m, 2H), 6.67 (dd, *J* = 15.6, 10.3 Hz, 1H), 6.55 (d, *J* = 15.6 Hz, 1H), 5.87 (dt, *J* = 15.1, 6.6 Hz, 1H), 4.74 (dd, *J* = 6.6, 1.3 Hz, 2H), 3.75 (s, 3H).

^{13}C{^{1}H} NMR (101 MHz, $CDCl_3$) δ 163.8, 157.5, 135.0, 133.0, 128.2, 127.5, 126.6, 115.9, 115.7, 66.2, 64.9.

^{19}F NMR (376 MHz, $CDCl_3$) δ -113.88.

HRMS (ESI) m/z calculated for $C_{13}H_{14}FNO_3$ $[M+H]^+$: 252.1031. Found: 252.1030.

mp. range: 35–36 °C.

(2*E*,4*E*)-5-phenylpenta-2,4-dien-1-yl methoxycarbamate (1b)

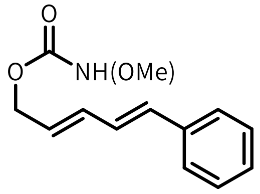

Prepared according to standard procedure: **1)** (*E*)-cinnamaldehyde (1.59 g, 12.0 mmol, 1.0 equiv.) THF (24.0 mL, 0.5M), NaH (0.58 g, 14.4 mmol, 1.2 equiv.), Triethylphosphonoacetate (2.9 mL, 14.4 mmol, 1.2 equiv.). **2)** DIBAL-H (1M in PhMe) (24.0 mL, 24.0 mmol, 2.0 equiv.), DCM (60.0 mL, 0.2M). Product with minor impurities was obtained as a solid and used without further purification. **3)** MeCN (60.0 mL, 0.2M), CDI (2.92 g, 18.0 mmol, 1.5 equiv.), imidazole (3.27 g, 48.0 mmol, 4.0 equiv.), methoxyamine HCl (4.01 g, 48.0 mmol, 4.0 equiv.). Crude material was purified by flash column chromatography (SiO_2) using a gradient of 30% --> 50% EtOAc in hexanes to afford pure product as a white solid (1.21 g, 5.2 mmol, 43% over three steps).

^{1}H NMR (400 MHz, $CDCl_3$) δ 7.59 (s, 1H), 7.49 – 7.38 (m, 2H), 7.38 – 7.33 (m, 2H), 7.29 (d, *J* = 7.7 Hz, 1H), 6.81 (dd, *J* = 15.5, 10.4 Hz, 1H), 6.63 (d, *J* = 15.7 Hz, 1H), 6.51 (dd, *J* = 15.2, 10.4 Hz, 1H), 5.92 (dt, *J* = 15.0, 6.6 Hz, 1H), 4.78 (d, *J* = 6.6 Hz, 2H), 3.79 (s, 3H).

^{13}C{^{1}H} NMR (101 MHz, $CDCl_3$) δ 157.5, 136.9, 135.1, 134.2, 128.8, 128.0, 127.7, 126.6, 66.2, 64.8.

HRMS (ESI) m/z calculated for $C_{13}H_{15}NO_3$ $[M+H]^+$: 234.1125. Found: 234.1120.

mp. range: 55–56 °C.

(2*E*,4*E*)-5-(4-methoxyphenyl)penta-2,4-dien-1-yl methoxycarbamate (1c)

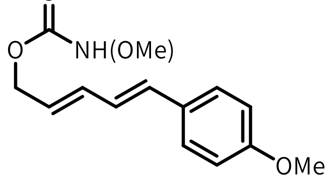

Prepared according to standard procedure: **1)** (*E*)-3-(4-methoxyphenyl)acrylaldehyde (1.30 g, 8.0 mmol, 1.0 equiv.) THF (16.0 mL, 0.5M), NaH (0.38 g, 9.6 mmol, 1.2 equiv.), Triethylphosphonoacetate (1.9 mL, 9.6 mmol, 1,2 equiv.). **2)** DIBAL-H (1M in PhMe) (16.0 mL, 16.0 mmol, 2.0 equiv.), DCM (40.0 mL, 0.2M). Product with minor impurities was obtained as

a solid and used without further purification. **3)** MeCN (40.0 mL, 0.2M), CDI (1.95 g, 12.0 mmol, 1.5 equiv.), imidazole (2.18 g mg, 32.0 mmol, 4.0 equiv.), methoxyamine HCl (2.67 g, 32.0 mmol, 4.0 equiv.). Crude material was purified by flash column chromatography (SiO_2) using a gradient of 30% --> 50% EtOAc in hexanes to afford pure product as a white solid (0.57 g, 2.2 mmol, 27% over three steps).

^{1}H NMR (400 MHz, $CDCl_3$) δ 7.44 (s, 1H), 7.37 – 7.27 (m, 2H), 6.90 – 6.81 (m, 2H), 6.64 (dd, *J* = 15.5, 10.2 Hz, 1H), 6.54 (d, *J* = 15.6 Hz, 1H), 6.45 (dd, *J* = 15.2, 10.2 Hz, 1H), 5.83 (dt, *J* = 15.0, 6.7 Hz, 1H), 4.73 (dd, *J* = 6.7, 1.2 Hz, 2H), 3.81 (s, 3H), 3.77 (s, 1H).

^{13}C{^{1}H} NMR (101 MHz, $CDCl_3$) δ 159.6, 157.5, 135.7, 133.9, 129.8, 127.9, 125.7, 125.3, 114.3, 66.4, 64.9, 55.4.

HRMS (ESI) m/z calculated for $C_{14}H_{17}NO_4$ $[M+H]^+$: 264.1231. Found: 264.1230.

mp. range: 86–87 °C.

(2*E*,4*E*)-5-(4-methylphenyl)penta-2,4-dien-1-yl methoxycarbamate (1d)

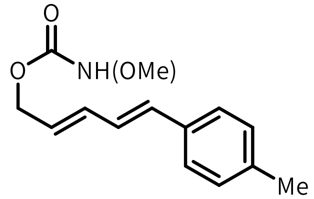

Prepared according to standard procedure: **1)** (*E*)-3-(4-methylphenyl)acrylaldehyde (1.00 g, 6.84 mmol, 1.0 equiv.) THF (13.68 mL, 0.5M), NaH (0.33 g, 8.21 mmol, 1.2 equiv.), Triethylphosphonoacetate (1.6 mL, 8.21 mmol, 1,2 equiv.). **2)** DIBAL-H (1M in PhMe) (13.68 mL, 13.68 mmol, 2.0 equiv.), DCM (34.2 mL, 0.2M). Product with minor impurities was obtained as a solid and used without further purification. **3)** MeCN (34.2 mL, 0.2M), CDI (1.66 g, 10.3 mmol, 1.5 equiv.), imidazole (1.86 g, 27.36 mmol, 4.0 equiv.), methoxyamine HCl (2.29 g, 27.36 mmol, 4.0 equiv.). Crude material was purified by flash column chromatography (SiO_2) using a gradient of 30% --> 50% EtOAc in hexanes to afford pure product as a white solid (0.49 g, 2.0 mmol, 29% over three steps).

^{1}H NMR (400 MHz, $CDCl_3$) δ 7.83 (s, 1H), 7.29 (d, *J* = 8.1 Hz, 2H), 7.12 (d, *J* = 7.9 Hz, 2H), 6.72 (dd, *J* = 15.6, 10.5 Hz, 1H), 6.56 (d, *J* = 15.6 Hz, 1H), 6.45 (ddt, *J* = 15.2, 10.3, 1.4 Hz, 1H), 5.86 (dt, *J* = 15.1, 6.6 Hz, 1H), 4.74 (dd, *J* = 6.7, 1.3 Hz, 2H), 3.75 (s, 3H), 2.34 (s, 3H).

^{13}C{^{1}H} NMR (101 MHz, $CDCl_3$) δ 157.5, 137.8, 135.3, 134.1, 134.1, 129.4, 126.6, 126.5, 125.9, 66.2, 64.6, 21.3.

HRMS (ESI) m/z calculated for $C_{14}H_{17}NO_3$ $[M+H]^+$: 248.1282. Found: 248.1280.

mp. range: 33–34 °C.

(2*E*,4*E*)-5-(4-(trifluoromethyl)phenyl)penta-2,4-dien-1-yl methoxycarbamate (1e)

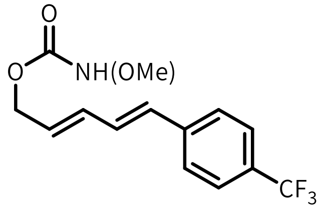

Prepared according to standard procedure: **1)** (*E*)-3-(4-(trifluoromethyl)phenyl)acrylaldehyde (3.00 g, 15.0 mmol, 1.0 equiv.) THF (30.0 mL, 0.5M), NaH (0.72 g, 18.0 mmol, 1.2 equiv.), Triethylphosphonoacetate (3.6 mL, 18.0 mmol, 1,2 equiv.). **2)** DIBAL-H (1M in PhMe) (30.0 mL, 30.0 mmol, 2.0 equiv.), DCM (75.0 mL, 0.2M). Product with minor impurities was obtained as a solid and used without further purification. **3)** MeCN (75.0 mL, 0.2M), CDI (3.65 g, 22.5 mmol, 1.5 equiv.), imidazole (4.08 g, 60.0 mmol, 4.0 equiv.), methoxyamine HCl (5.01 g, 60.0 mmol, 4.0 equiv.). Crude material was purified by flash column chromatography (SiO_2) using a gradient of 30% --> 50% EtOAc in hexanes to afford pure product as a white solid (2.934 g, 9.75 mmol, 65% over three steps).

^{1}H NMR (400 MHz, CDCl3) δ 7.56 (d, J = 8.3 Hz, 2H), 7.47 (d, J = 8.4 Hz, 2H), 6.83 (dd, J = 15.7, 10.5 Hz, 1H), 6.60 (d, J = 15.7 Hz, 1H), 6.53 – 6.41 (m, 1H), 5.95 (dt, J = 15.2, 6.4 Hz, 1H), 4.79 – 4.71 (m, 2H), 3.75 (s, 3H).

^{13}C{^{1}H} NMR (101 MHz, $CDCl_3$) δ 157.2, 140.2, 134.1, 132.3, 129.9, 128.3, 126.5, 125.6, 125.5, 77.3, 77.0, 76.7, 65.7, 64.7.

^{19}F NMR (376 MHz, $CDCl_3$) δ -62.43.

HRMS (ESI) m/z calculated for $C_{14}H_{14}F_3NO_3$ $[M+H]^+$: 302.1004. Found: 302.1001.

mp. range: 46–47 °C.

(2*E*,4*E*)-5-(o-tolyl)penta-2,4-dien-1-yl methoxycarbamate (1f)

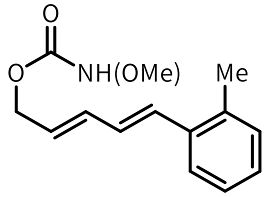

Prepared according to standard procedure: **1)** (*E*)-3-(2-methylphenyl)acrylaldehyde (1.75 g, 12.0 mmol, 1.0 equiv.) THF (24.0 mL, 0.5M), NaH (0.57 g, 14.4 mmol, 1.2 equiv.), Triethylphosphonoacetate (2.9 mL, 14.4 mmol, 1,2 equiv.). **2)** DIBAL-H (1M in PhMe) (24.00 mL, 24.00 mmol, 2.0 equiv.), DCM (60 mL, 0.2M). Product with minor impurities was obtained as a clear oil used without further purification. **3)** MeCN (60 mL, 0.2M), CDI (2.91 g, 18 mmol, 1.5 equiv.), imidazole (3.26 g, 48.00 mmol, 4.0 equiv.), methoxyamine HCl (3.98 g, 48.00 mmol, 4.0 equiv.). Crude material was purified by flash column chromatography (SiO_2) using a gradient of 30% --> 50% EtOAc in hexanes to afford pure product as a white solid (1.98 g, 8.04 mmol, 67% over three steps).

^{1}H NMR (500 MHz, $CDCl_3$) δ 7.49 – 7.44 (m, 1H), 7.38 (s, 1H), 7.15 (d, *J* = 9.4 Hz, 2H), 6.81 (d, *J* = 15.4 Hz, 1H), 6.66 (dd, *J* = 15.5, 10.4 Hz, 1H), 6.50 (dd, *J* = 15.2, 10.4 Hz, 1H), 5.87 (dt, *J* = 14.3, 6.6 Hz, 1H), 4.73 (d, *J* = 6.6 Hz, 2H), 3.74 (s, 3H), 2.34 (s, 3H).

^{13}C{^{1}H} NMR (101 MHz, $CDCl_3$) δ 123.9, 123.9, 123.7, 120.7, 119.7, 118.2, 117.5, 116.3, 116.2, 115.5, 68.2, 67.1, 31.1.

HRMS (ESI) m/z calculated for $C_{14}H_{17}NO_3$ $[M+H]^+$: 248.1282. Found: 248.1271.

mp. range: 29–30 °C.

(*2E,4E*)-4-methyl-5-phenylpenta-2,4-dien-1-yl methoxycarbamate (1g)

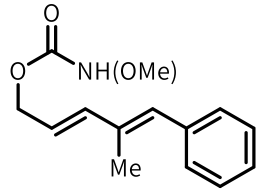

Prepared according to standard procedure: **1)** (*E*)-2-methyl-3-phenylacrylaldehyde (1.46 g, 10.0 mmol, 1.0 equiv.) THF (20.0 mL, 0.5M), NaH (0.48 g, 12.0 mmol, 1.2 equiv.), Triethylphosphonoacetate (2.4 mL, 12.0 mmol, 1,2 equiv.). **2)** DIBAL-H (1M in PhMe) (20.0 mL, 20.0 mmol, 2.0 equiv.), DCM (50.0 mL, 0.2M). Product with minor impurities was obtained as a solid and used without further purification. **3)** MeCN (50.0 mL, 0.2M), CDI (2.43 g, 15.0 mmol, 1.5 equiv.), imidazole (2.72 g, 40.0 mmol, 4.0 equiv.), methoxyamine HCl (3.34 g, 40.0 mmol, 4.0 equiv.). Crude material was purified by flash column chromatography (SiO_2) using a gradient of 30% --> 50% EtOAc in hexanes to afford pure product as a white solid (1.178 g, 4.8 mmol, 48% over three steps).

^{1}H NMR (400 MHz, $CDCl_3$) δ 7.72 – 7.51 (m, 1H), 7.39 – 7.32 (m, 2H), 7.32 – 7.27 (m, 2H), 6.57 (s, 1H), 6.55 – 6.51 (m, 1H), 5.86 (dt, *J* = 15.6, 6.6 Hz, 1H), 4.78 (dd, *J* = 6.6, 1.2 Hz, 2H), 3.76 (s, 3H), 2.01 (d, *J* = 1.3 Hz, 3H).

^{13}C{^{1}H} NMR (101 MHz, $CDCl_3$) δ 157.6, 140.2, 137.5, 134.7, 133.0, 129.3, 128.3, 127.0, 122.0, 66.7, 64.8, 13.9.

HRMS (ESI) m/z calculated for $C_{14}H_{17}NO_3$ $[M+H]^+$: 248.1282. Found: 248.1280.

mp. range: 30–31 °C.

(2*E*,4*E*)-4-bromo-5-phenylpenta-2,4-dien-1-yl methoxycarbamate (1h)

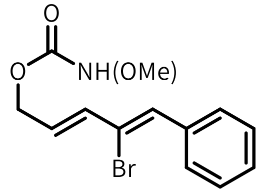

Prepared according to standard procedure: **1)** (*E*)-2-bromo-3-phenylacrylaldehyde (1.27 g, 6.0 mmol, 1.0 equiv.) THF (12.0 mL, 0.5M), NaH (0.29 g, 7.2 mmol, 1.2 equiv.), Triethylphosphonoacetate (1.4 mL, 7.2 mmol, 1,2 equiv.). **2)** DIBAL-H (1M in PhMe) (12.0 mL, 12.0 mmol, 2.0 equiv.), DCM (30.0 mL, 0.2M). Product with minor impurities was obtained as a solid and used without further purification. **3)** MeCN (30.0 mL, 0.2M), CDI (1.46 g, 9.0 mmol, 1.5 equiv.), imidazole (1.63 g, 24.0 mmol, 4.0 equiv.), methoxyamine HCl (2.0 g, 24.0 mmol, 4.0 equiv.). Crude material was purified by flash column chromatography (SiO_2) using a gradient of 30% --> 50% EtOAc in hexanes to afford pure product as a white solid (0.532 g, 1.7 mmol, 28% over three steps).

^{1}H NMR (500 MHz, $CDCl_3$) δ 7.68 (dd, *J* = 7.5, 2.7 Hz, 2H), 7.43 (s, 1H), 7.38 (td, *J* = 7.6, 2.5 Hz, 2H), 7.32 (dd, *J* = 8.5, 6.0 Hz, 1H), 7.01 (d, *J* = 2.5 Hz, 1H), 6.50 (d, *J* = 14.7 Hz, 1H), 6.28 (ddd, *J* = 14.6, 7.4, 5.0 Hz, 1H), 4.84 (d, *J* = 6.0 Hz, 2H), 3.77 (d, *J* = 2.5 Hz, 3H).

^{13}C{^{1}H} NMR (126 MHz, $CDCl_3$) δ 157.3, 135.5, 134.6, 133.4, 129.7, 128.6, 128.4, 128.3, 121.9, 65.2, 64.9.

HRMS (ESI) m/z calculated for $C_{13}H_{14}BrNO_3$ $[M+H]^+$: 312.0230. Found: 312.0262.

mp. range: 32–33 °C.

(*E*)-5-methylhexa-2,4-dien-1-yl methoxycarbamate (1i)

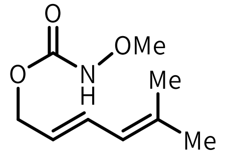

Prepared according to standard procedure, starting from (*E*)-5-methylhexa-2,4-dien-1-ol (1.00 g, 8.9 mmol, 1.0 equiv.): **3)** MeCN (45.0 mL, 0.2M), CDI (2.16 g, 13.4 mmol, 1.5 equiv.), imidazole (2.4 g, 35.6 mmol, 4.0 equiv.), methoxyamine HCl (2.97 g, 35.6 mmol, 4.0 equiv.). Crude material was purified by flash column chromatography (SiO_2) using a gradient of 30% --> 50% EtOAc in hexanes to afford pure product as a clear oil (0.70 g, 3.8 mmol, 42%).

^{1}H NMR (400 MHz, $CDCl_3$) δ 7.40 (s, 1H), 6.52 (dd, *J* = 15.0, 11.0 Hz, 1H), 5.83 (d, *J* = 11.0 Hz, 1H), 5.62 (dt, *J* = 14.4, 6.8 Hz, 1H), 4.68 (d, *J* = 6.8 Hz, 2H), 3.73 (d, *J* = 0.8 Hz, 3H), 1.77 (d, *J* = 8.1 Hz, 6H).

^{13}C{^{1}H} NMR (101 MHz, $CDCl_3$) δ 157.6, 138.1, 132.1, 124.0, 123.1, 66.8, 64.8, 26.2, 18.5.

HRMS (ESI) m/z calculated for $C_9H_{15}NO_3$ $[M+H]^+$: 186.1125. Found: 186.1130.

(2*E*,4*E*)-hexa-2,4-dien-1-yl methoxycarbamate (1j)

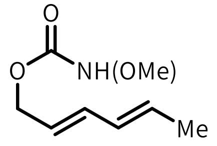

Prepared according to standard procedure, starting from (2*E*,4*E*)-hexa-2,4-dien-1-ol (1.00 g, 10.2 mmol, 1.0 equiv.): **3)** MeCN (51.0 mL, 0.2M), CDI (2.48 g, 15.3 mmol, 1.5 equiv.), imidazole (2.78 g, 40.8 mmol, 4.0 equiv.), methoxyamine HCl (3.63 g, 40.8 mmol, 4.0 equiv.). Crude material was purified by flash column chromatography (SiO_2) using a gradient of 30% --> 50% EtOAc in hexanes to afford pure product as a clear oil (1.49 g, 8.7 mmol, 85%).

^{1}H NMR (400 MHz, $CDCl_3$) δ 7.39 (s, 1H), 6.27 (dd, *J* = 15.2, 10.4 Hz, 1H), 6.05 (ddd, *J* = 15.2, 10.3, 1.8 Hz, 1H), 5.76 (dq, *J* = 13.8, 6.7 Hz, 1H), 5.63 (dt, *J* = 14.5, 6.7 Hz, 1H), 4.65 (d, *J* = 6.7 Hz, 2H), 3.73 (s, 3H), 1.76 (dd, *J* = 6.7, 1.6 Hz, 3H).

^{13}C{^{1}H} NMR (101 MHz, $CDCl_3$) δ 153.9, 135.6, 131.9, 130.5, 123.4, 66.5, 64.8, 18.3.

HRMS (ESI) m/z calculated for $C_8H_{13}NO_3$ $[M+Na]^+$: 194.0778. Found: 194.0770.

(2*E*,4*E*)-5-(furan-2-yl)penta-2,4-dien-1-yl methoxycarbamate (1k)

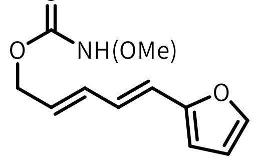

Prepared according to standard procedure: (*E*)-3-(2-furyl)acrolein (0.61 g, 5.0 mmol, 1.0 equiv.) THF (10.0 mL, 0.5M), NaH (0.24 g, 6.0 mmol, 1.2 equiv.), Triethyl 2-phosphonopropionate (1.3 mL, 6.0 mmol, 1.2 equiv.). **2)** DIBAL-H (1M in PhMe) (10.0 mL, 10.0 mmol, 2.0 equiv.), DCM (25.0 mL, 0.2M). Product with minor impurities was obtained as a solid and used without further purification. **3)** MeCN (25.0 mL, 0.2M), CDI (1.22 g, 7.5 mmol, 1.5 equiv.), imidazole (1.36 g, 20.0 mmol, 4.0

equiv.), methoxyamine HCl (1.67 g, 20.0 mmol, 4.0 equiv.). Crude material was purified by flash column chromatography (SiO_2) using a gradient of 30% --> 50% EtOAc in hexanes to afford pure product as a clear oil (0.55 g, 2.5 mmol, 50% over three steps).

1H NMR (400 MHz, $CDCl_3$) δ 7.64 (s, 1H), 7.35 (d, *J* = 1.8 Hz, 1H), 6.66 (dd, *J* = 15.6, 10.7 Hz, 1H), 6.44 – 6.30 (m, 3H), 6.28 (d, *J* = 3.4 Hz, 1H), 5.85 (dt, *J* = 14.9, 6.6 Hz, 1H), 4.71 (dd, *J* = 6.6, 1.2 Hz, 2H), 3.73 (s, 3H).

$^{13}C\{^1H\}$ NMR (101 MHz, $CDCl_3$) δ 157.5, 152.8, 142.5, 134.7, 126.6, 126.2, 121.6, 111.8, 109.2, 66.1, 64.8.

HRMS (ESI) m/z calculated for $C_{11}H_{13}NO_4$ $[M+H]^+$: 224.0918. Found: 224.0920.

(2*E*,4*E*)-5-(pyridin-2-yl)penta-2,4-dien-1-yl methoxycarbamate (1l)

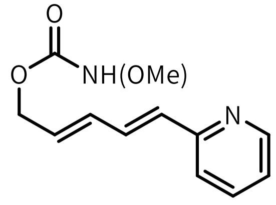

Prepared according to standard procedure: **1)** (*E*)-3-(pyridin-2-yl)acrylaldehyde (1.59 g, 12.0 mmol, 1.0 equiv.) THF (24.0 mL, 0.5M), NaH (0.57 g, 14.4 mmol, 1.2 equiv.), Triethylphosphonoacetate (2.9 mL, 14.4 mmol, 1,2 equiv.). **2)** DIBAL-H (1M in PhMe) (24.0 mL, 24.0 mmol, 2.0 equiv.), DCM (60 mL, 0.2M). Product with minor impurities was obtained as a yellow oil used without further purification. **3)** MeCN (60 mL, 0.2M), CDI (2.91 g, 18 mmol, 1.5 equiv.), imidazole (3.26 g, 48.0 mmol, 4.0 equiv.), methoxyamine HCl (3.98 g, 48.0 mmol, 4.0 equiv.). Crude material was purified by flash column chromatography (SiO_2) using a gradient of 30% --> 50% EtOAc in hexanes to afford pure product as a yellow oil (0.85 g, 3.64 mmol, 30% over three steps).

^1H NMR (400 MHz, $CDCl_3$) δ 8.54 (ddd, *J* = 4.8, 1.8, 1.0 Hz, 1H), 7.60 (tt, *J* = 7.7, 1.7 Hz, 1H), 7.31 – 7.17 (m, 2H), 7.15 – 7.06 (m, 1H), 6.63 (d, *J* = 15.5 Hz, 1H), 6.49 (dd, *J* = 15.2, 10.9 Hz, 1H), 5.98 (dt, *J* = 14.9, 6.3 Hz, 1H), 4.74 (dt, *J* = 6.5, 1.5 Hz, 2H), 3.77 – 3.70 (m, 3H).

$^{13}C\{^1H\}$ NMR (101 MHz, $CDCl_3$) δ 157.1, 155.0, 149.5, 136.3, 133.7, 132.9, 131.4, 129.0, 122.1, 121.9, 65.6, 64.5.

HRMS (ESI) *m/z* calculated for $C_{12}H_{14}N_2O_3$ $[M+H]^+$: 235.1080. Found: 235.1079.

(*2E,4E*)-5-(3-(trifluoromethyl)phenyl)penta-2,4-dien-1-yl methoxycarbamate (1m)

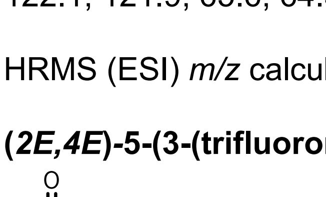

Prepared according to standard procedure: **1)** (*E*)-3-(3-(trifluoromethyl)phenyl)acrylaldehyde (3.00 g, 15.0 mmol, 1.0 equiv.) THF (30.0 mL, 0.5M), NaH (0.72 g, 18.0 mmol, 1.2 equiv.), Triethylphosphonoacetate (3.6 mL, 18.0 mmol, 1,2 equiv.). **2)** DIBAL-H (1M in PhMe) (30.0 mL, 30.0 mmol, 2.0 equiv.), DCM (75.0 mL, 0.2M). Product with minor impurities was obtained as a solid and used without further purification. **3)** MeCN (75.0 mL, 0.2M), CDI (3.65 g, 22.5 mmol, 1.5 equiv.), imidazole (4.08 g, 60.0 mmol, 4.0 equiv.), methoxyamine HCl (5.01 g, 60.0 mmol, 4.0 equiv.). Crude material was purified by flash column

chromatography (SiO_2) using a gradient of 30% --> 50% EtOAc in hexanes to afford pure product as a white solid (2.756 g, 9.14 mmol, 61% over three steps).

^{1}H NMR (400 MHz, $CDCl_3$) δ 7.64 – 7.38 (m, 5H), 6.87 – 6.75 (m, 1H), 6.59 (dd, *J* = 15.7, 2.0 Hz, 1H), 6.52 – 6.41 (m, 1H), 5.94 (dt, *J* = 15.3, 6.4 Hz, 1H), 4.75 (dd, *J* = 6.4, 1.3 Hz, 2H), 3.75 (d, *J* = 1.0 Hz, 3H).

^{13}C{^{1}H} NMR (101 MHz, $CDCl_3$) δ 157.4, 137.7, 134.3, 132.4, 129.6, 129.5, 129.2, 128.1, 125.5, 124.4, 123.2, 122.8, 65.9, 64.9.

^{19}F NMR (376 MHz, $CDCl_3$) δ -63.01.

HRMS (ESI) m/z calculated for $C_{14}H_{14}F_3NO_3$ $[M+H]^+$: 302.0999. Found: 302.1000.

mp. range: 43–44 °C.

(2E,4E)-5-(benzo[d][1,3]dioxol-5-yl)penta-2,4-dien-1-yl methoxycarbamate (1n)

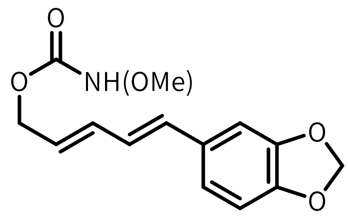

Prepared according to standard procedure: **1)** (E)-3-(benzo[d][1,3]dioxol-5-yl)acrylaldehyde (2.64 g, 15.0 mmol, 1.0 equiv.) THF (30.0 mL, 0.5M), NaH (0.72 g, 18.0 mmol, 1.2 equiv.), Triethylphosphonoacetate (3.6 mL, 18.0 mmol, 1,2 equiv.). **2)** DIBAL-H (1M in PhMe) (30.0 mL, 30.0 mmol, 2.0 equiv.), DCM (75.0 mL, 0.2M). Product with minor impurities was obtained as a solid and used without further purification. **3)** MeCN (75.0 mL, 0.2M), CDI (3.65 g, 22.5 mmol, 1.5 equiv.), imidazole (4.08 g, 60.0 mmol, 4.0 equiv.), methoxyamine HCl (5.01 g, 60.0 mmol, 4.0 equiv.). Crude material was purified by flash column chromatography (SiO_2) using a gradient of 30% --> 50% EtOAc in hexanes to afford pure product as a white solid (2.409 g, 8.7mmol, 58% over three steps).

^{1}H NMR (400 MHz, CHLOROFORM-*D*) δ 7.37 (s, 1H), 6.94 (t, *J* = 1.2 Hz, 1H), 6.89 – 6.79 (m, 1H), 6.76 (d, *J* = 8.0 Hz, 1H), 6.60 (dd, *J* = 15.5, 10.2 Hz, 1H), 6.55 – 6.38 (m, 2H), 5.96 (d, *J* = 0.9 Hz, 2H), 5.84 (dt, *J* = 14.3, 6.7 Hz, 1H), 4.73 (d, *J* = 6.6 Hz, 2H), 3.75 (d, *J* = 0.9 Hz, 3H).

^{13}C{^{1}H} NMR (101 MHz, CHLOROFORM-*D*) δ 157.5, 148.2, 147.6, 135.3, 131.4, 126.0, 125.9, 125.7, 121.7, 108.5, 105.6, 101.3, 77.5, 77.2, 76.8, 66.3, 64.8.

HRMS (ESI) m/z calculated for $C_{14}H_{15}NO_5$ $[M+H]^+$: 278.1028. Found: 278.1021.

mp. range: 89–90 °C.

Reaction Investigations

Copper Source Studies

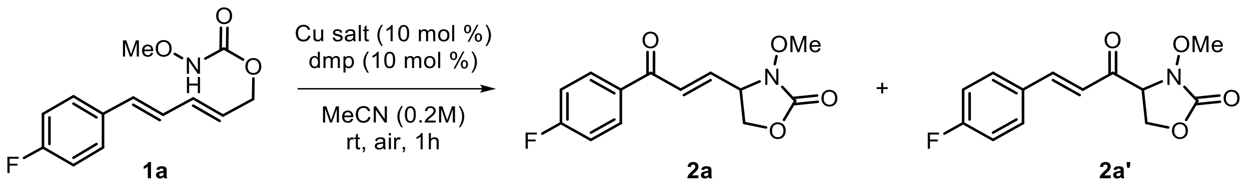

Table S1. Copper source studies.

Entry	Copper Source	Yield 2a	Yield 2a’	Aldehyde
**1**	CuCl	52	6	3
**2**	$CuCl_2$	29	9	6
**3**	CuOAc	6	16	7
**4**	$Cu(OAc)_2$	6	40	24
**5**	Cu(OTf)•PhMe	21	28	29
**6**	$Cu(OTf)_2$	15	25	28
**7**	CuI	23	23	13
**8**	CuI_2	47	12	13
**9**	CuBr	46	6	3
**10**	$CuBr_2$	9	29	8
**11**	CuTC	45	13	11
**12**	$Cu(eh)_2$	4	44	21

General Procedure for copper source investigation:
Copper salt stock solution: Copper salt (0.25 mmol, 1.0 equiv.) was weighed into a 20 mL scintillation vial in a glovebox. The vial was sealed, removed from the glovebox and the solids were taken up in MeCN (3.1 mL, 4.0mM) and stirred until homogenous (15 min).
Ligand stock solution: dmp (52.1 mg, 0.25 mmol, 1.0 equiv.) was weighed into a 20 mL scintillation vial in a glovebox. The vial was sealed, removed from the glovebox and the solids were taken up in MeCN (3.1 mL, 4.0mM) and stirred until homogenous (15 min).
Reaction: 0.125 mL of the copper salt stock solution, and 0.125 mL ligand stock solution (containing dmp (1.0 mg, 5.0 µmol, 0.1 equiv.)) was transferred to an uncapped, flame-dried 1 dram vial equipped with a stir bar. (2*E*,4*E*)-5-(4-fluorophenyl)penta-2,4-dien-1-yl methoxycarbamate (**1a**) (12.6 mg, 0.050 mmol, 1.0 equiv.) was added in one portion and the reaction mixture was stirred at 500 rpm for 1h in the uncapped vial. 20.0 µL of the reaction mixture was filtered through a ~2 cm pad of SiO_2 in a Pasteur pipette and eluting with ~1 mL EtO_2. The crude residue was analyzed by gas chromatography using anisole (2.5 µL, 0.023 mmol, 0.46 equiv.) as an internal standard.

Entry 1: CuCl General procedure for catalyst investigations was followed. 0.125 mL catalyst stock solution (containing CuCl (0.6 mg, 5.0 µmol, 0.10 equiv.), 0.125 mL ligand stock solution (containing dmp (1.0 mg, 5.0 µmol, 0.1 equiv.)), *(2E,4E)*-5-(4-fluorophenyl)penta-2,4-dien-1-yl methoxycarbamate (**1a**) (12.6 mg, 0.050 mmol, 1.0

equiv.). Product yield was analyzed by gas chromatography using anisole as an internal standard.

<u>Run Number</u>: (yield of **2a** in mmol, yield of **2a** percent) (yield of **2a’** in mmol, yield of **2a’** percent)

<u>Run 1</u>: (0.027 mmol, 54%) (0.0029 mmol, 6%)
<u>Run 2</u>: (0.0245 mmol, 49%) (0.003 mmol, 6%)
Average: 58% yield (90:10)

Entry 2: $CuCl_2$ General procedure for catalyst investigations was followed. 0.125 mL catalyst stock solution (containing $CuCl_2$ (0.7 mg, 5.0 μmol, 0.10 equiv.), 0.125 mL ligand stock solution (containing dmp (1.0 mg, 5.0 μmol, 0.1 equiv.)), *(2E,4E)*-5-(4-fluorophenyl)penta-2,4-dien-1-yl methoxycarbamate (**1a**) (12.6 mg, 0.050 mmol, 1.0 equiv.). Product yield was analyzed by gas chromatography using anisole as an internal standard.

<u>Run Number</u>: (yield of **2a** in mmol, yield of **2a** percent) (yield of **2a’** in mmol, yield of **2a’** percent)

<u>Run 1</u>: (0.0198 mmol, 39.6%) (0.00325 mmol, 6.5%)
<u>Run 2</u>: (0.0189 mmol, 37.8%) (0.006 mmol, 12.0%)
Average: 48% yield (81:19)

Entry 3: CuOAc General procedure for catalyst investigations was followed. 0.125 mL catalyst stock solution (containing CuOAc (0.6 mg, 5.0 μmol, 0.10 equiv.), 0.125 mL ligand stock solution (containing dmp (1.0 mg, 5.0 μmol, 0.1 equiv.)), *(2E,4E)*-5-(4-fluorophenyl)penta-2,4-dien-1-yl methoxycarbamate (**1a**) (12.6 mg, 0.050 mmol, 1.0 equiv.). Product yield was analyzed by gas chromatography using anisole as an internal standard.

<u>Run Number</u>: (yield of **2a** in mmol, yield of **2a** percent) (yield of **2a’** in mmol, yield of **2a’** percent)

<u>Run 1</u>: (0.004 mmol, 8%) (0.0103 mmol, 20.6%)
<u>Run 2</u>: (0.0015 mmol, 3%) (0.005 mmol, 10%)
Average: 21% yield (26:74)

Entry 4: $Cu(OAc)_2$ General procedure for catalyst investigations was followed. 0.125 mL catalyst stock solution (containing $Cu(OAc)_2$ (0.9 mg, 5.0 μmol, 0.10 equiv.), 0.125 mL ligand stock solution (containing dmp (1.0 mg, 5.0 μmol, 0.1 equiv.)), *(2E,4E)*-5-(4-fluorophenyl)penta-2,4-dien-1-yl methoxycarbamate (**1a**) (12.6 mg, 0.050 mmol, 1.0 equiv.). Product yield was analyzed by gas chromatography using anisole as an internal standard.

Run Number: (yield of **2a** in mmol, yield of **2a** percent) (yield of **2a'** in mmol, yield of **2a'** percent)

Run 1: (0.005 mmol, 5%) (0.0209 mmol, 41.7%)
Run 2: (0.0039 mmol, 7.8%) (0.0196 mmol, 39.2%)
Average: 47% yield (14:86)

Entry 5: Cu(OTf)•PhMe General procedure for catalyst investigations was followed. 0.125 mL catalyst stock solution (containing Cu(OTf)-PhMe (2.6 mg, 5.0 μmol, 0.10 equiv.), 0.125 mL ligand stock solution (containing dmp (1.0 mg, 5.0 μmol, 0.1 equiv.)), *(2E,4E)*-5-(4-fluorophenyl)penta-2,4-dien-1-yl methoxycarbamate (**1a**) (12.6 mg, 0.050 mmol, 1.0 equiv.). Product yield was analyzed by gas chromatography using anisole as an internal standard.

Run Number: (yield of **2a** in mmol, yield of **2a** percent) (yield of **2a'** in mmol, yield of **2a'** percent)

Run 1: (0.0105 mmol, 21%) (0.0135 mmol, 27%)
Run 2: (0.0105 mmol, 21%) (0.014 mmol, 28%)
Average: 49% yield (43:57)

Entry 6: $Cu(OTf)_2$ General procedure for catalyst investigations was followed. 0.125 mL catalyst stock solution (containing $Cu(OTf)_2$ (1.8 mg, 5.0 μmol, 0.10 equiv.), 0.125 mL ligand stock solution (containing dmp (1.0 mg, 5.0 μmol, 0.1 equiv.)), *(2E,4E)*-5-(4-fluorophenyl)penta-2,4-dien-1-yl methoxycarbamate (**1a**) (12.6 mg, 0.050 mmol, 1.0 equiv.). Product yield was analyzed by gas chromatography using anisole as an internal standard.

Run Number: (yield of **2a** in mmol, yield of **2a** percent) (yield of **2a'** in mmol, yield of **2a'** percent)

Run 1: (0.0065 mmol, 13%) (0.0113 mmol, 22.5%)
Run 2: (0.008 mmol, 16%) (0.0135 mmol, 27%)
Average: 39% yield (37:63)

Entry 7: CuI General procedure for catalyst investigations was followed. 0.125 mL catalyst stock solution (containing CuI (1.0 mg, 5.0 μmol, 0.10 equiv.), 0.125 mL ligand stock solution (containing dmp (1.0 mg, 5.0 μmol, 0.1 equiv.)), *(2E,4E)*-5-(4-fluorophenyl)penta-2,4-dien-1-yl methoxycarbamate (**1a**) (12.6 mg, 0.050 mmol, 1.0 equiv.). Product yield was analyzed by gas chromatography using anisole as an internal standard.

Run Number: (yield of **2a** in mmol, yield of **2a** percent) (yield of **2a'** in mmol, yield of **2a'** percent)

Run 1: (0.01 mmol, 20%) (0.0115 mmol, 23%)

Run 2: (0.0125 mmol, 25%) (0.011 mmol, 22%)
Average: 45% yield (50:50)

Entry 8: CuI_2 General procedure for catalyst investigations was followed. 0.125 mL catalyst stock solution (containing CuI_2 (1.6 mg, 5.0 μmol, 0.10 equiv.), 0.125 mL ligand stock solution (containing dmp (1.0 mg, 5.0 μmol, 0.1 equiv.)), *(2E,4E)*-5-(4-fluorophenyl)penta-2,4-dien-1-yl methoxycarbamate (**1a**) (12.6 mg, 0.050 mmol, 1.0 equiv.). Product yield was analyzed by gas chromatography using anisole as an internal standard.

Run Number: (yield of **2a** in mmol, yield of **2a** percent) (yield of **2a'** in mmol, yield of **2a'** percent)

Run 1: (0.022 mmol, 44%) (0.005 mmol, 10%)
Run 2: (0.0245 mmol, 49%) (0.0065 mmol, 13%)
Average: 58% yield (80:20)

Entry 9: CuBr General procedure for catalyst investigations was followed. 0.125 mL catalyst stock solution (containing CuBr (0.7 mg, 5.0 μmol, 0.10 equiv.), 0.125 mL ligand stock solution (containing dmp (1.0 mg, 5.0 μmol, 0.1 equiv.)), *(2E,4E)*-5-(4-fluorophenyl)penta-2,4-dien-1-yl methoxycarbamate (**1a**) (12.6 mg, 0.050 mmol, 1.0 equiv.). Product yield was analyzed by gas chromatography using anisole as an internal standard.

Run Number: (yield of **2a** in mmol, yield of **2a** percent) (yield of **2a'** in mmol, yield of **2a'** percent)

Run 1: (0.021 mmol, 42%) (0.00215 mmol, 4.3%)
Run 2: (0.025 mmol, 50%) (0.004 mmol, 8%)
Average: 52% yield (88:12)

Entry 10: $CuBr_2$ General procedure for catalyst investigations was followed. 0.125 mL catalyst stock solution (containing $CuBr_2$ (1.1 mg, 5.0 μmol, 0.10 equiv.), 0.125 mL ligand stock solution (containing dmp (1.0 mg, 5.0 μmol, 0.1 equiv.)), *(2E,4E)*-5-(4-fluorophenyl)penta-2,4-dien-1-yl methoxycarbamate (**1a**) (12.6 mg, 0.050 mmol, 1.0 equiv.). Product yield was analyzed by gas chromatography using anisole as an internal standard.

Run Number: (yield of **2a** in mmol, yield of **2a** percent) (yield of **2a'** in mmol, yield of **2a'** percent)

Run 1: (0.00465 mmol, 9.3%) (0.0157 mmol, 31.3%)
Run 2: (0.004 mmol, 8%) (0.0135 mmol, 27%)
Average: 38% yield (23:77)

Entry 11: CuTC General procedure for catalyst investigations was followed. 0.125 mL catalyst stock solution (containing CuTC (1.0 mg, 5.0 μmol, 0.10 equiv.), 0.125 mL ligand stock solution (containing dmp (1.0 mg, 5.0 μmol, 0.1 equiv.)), *(2E,4E)*-5-(4-fluorophenyl)penta-2,4-dien-1-yl methoxycarbamate (**1a**) (12.6 mg, 0.050 mmol, 1.0 equiv.). Product yield was analyzed by gas chromatography using anisole as an internal standard.

Run Number: (yield of **2a** in mmol, yield of **2a** percent) (yield of **2a'** in mmol, yield of **2a'** percent)

Run 1: (0.0235 mmol, 47%) (0.0075 mmol, 15%)
Run 2: (0.021 mmol, 42%) (0.005 mmol, 10%)
Average: 57% yield (78:22)

Entry 12: Cu(eh)$_2$ General procedure for catalyst investigations was followed. 0.125 mL catalyst stock solution (containing Cu(eh)$_2$ (1.8 mg, 5.0 μmol, 0.10 equiv.), 0.125 mL ligand stock solution (containing dmp (1.0 mg, 5.0 μmol, 0.1 equiv.)), *(2E,4E)*-5-(4-fluorophenyl)penta-2,4-dien-1-yl methoxycarbamate (**1a**) (12.6 mg, 0.050 mmol, 1.0 equiv.). Product yield was analyzed by gas chromatography using anisole as an internal standard.

Run Number: (yield of **2a** in mmol, yield of **2a** percent) (yield of **2a'** in mmol, yield of **2a'** percent)

Run 1: (0.0001 mmol, 2%) (0.023 mmol, 46%)
Run 2: (0.0025 mmol, 5%) (0.0205 mmol, 41%)
Average: 47% yield (7:93)

Ligand Studies

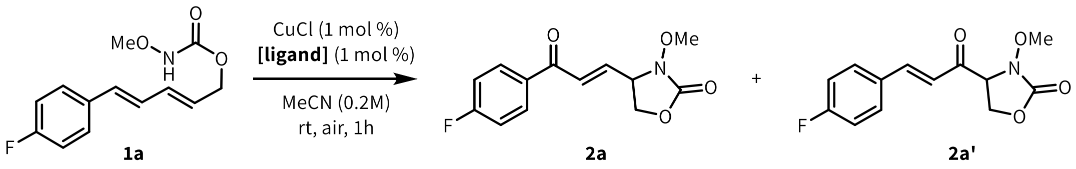

Table S2. Ligand studies.

Entry	ligand	Yield 2a	Yield 2a'	Aldehyde
1	none	18	8	11
2	dmp	47	6	3
3	4,7-dimethyl-2,9-phenanthroline	20	7	6
4	bipyridine	25	3	4
5	6,6′-dimethyl-2,2′-bipyridine	46	7	3

6	4,4′-dimethyl-2,2′-bipyridine	39	8	11

General procedure for ligand studies using CuCl:
CuCl stock solution: Copper chloride (5.0 mg, 50.0 μmol, 1.0 equiv.) was weighed into a 20 mL volumetric flask in a glovebox. The vial was sealed, removed from the glovebox and the solids were taken up in MeCN (12.5 mL, 4.0mM) and stirred until homogenous (15 min).
Ligand stock solution: Ligand (50.0 μmol, 1.0 equiv.) was weighed into a 20 mL volumetric flask in a glovebox. The vial was sealed, removed from the glovebox and the solids were taken up in MeCN (12.5 mL, 4.0mM) and stirred until homogenous (15 min).
Reaction: 0.125 mL of the catalyst stock solution (containing CuCl (0.05 mg, 0.500 μmol, 0.01 equiv.)) and 0.125 mL ligand stock solution (containing 0.500 μmol, 0.01 equiv.) was transferred to an uncapped, flame-dried 1 dram vial equipped with a stir bar. *(2E,4E)*-5-(4-fluorophenyl)penta-2,4-dien-1-yl methoxycarbamate (**1a**) (12.6 mg, 0.050 mmol, 1.0 equiv.) was added in one portion and the reaction mixture was stirred at 500 rpm for 1h in the uncapped vial. 20.0 μL of the reaction mixture was filtered through a ~2 cm pad of SiO_2 in a Pasteur pipette and eluting with ~1 mL EtO_2. The crude residue was analyzed by gas chromatography using anisole (2.5 μL, 0.023 mmol, 0.46 equiv.) as an internal standard.

Entry 1: no ligand General procedure for ligand studies was followed. 0.125 mL of the catalyst stock solution (containing CuCl (0.05 mg, 0.500 μmol, 0.01 equiv.)) 0.125 mL ligand stock solution (containing MeCN blank), *(2E,4E)*-5-(4-fluorophenyl)penta-2,4-dien-1-yl methoxycarbamate (**1a**) (12.6 mg, 0.050 mmol, 1.0 equiv.). Product yield was analyzed by gas chromatography using anisole as an internal standard.

Run Number: (yield of **2a** in mmol, yield of **2a** percent) (yield of **2a’** in mmol, yield of **2a’** percent)

Run 1: (0.0105 mmol, 21%) (0.0039 mmol, 8%)
Run 2: (0.0079 mmol, 16%) (0.0045 mmol, 9%)
Average: 26% yield (69:31)

Entry 2: dmp General procedure for ligand studies was followed. 0.125 mL of the catalyst stock solution (containing CuCl (0.05 mg, 0.500 μmol, 0.01 equiv.)) 0.125 mL ligand stock solution (containing dmp (0.10 mg, 0.500 μmol, 0.01 equiv.)), *(2E,4E)*-5-(4-fluorophenyl)penta-2,4-dien-1-yl methoxycarbamate (**1a**) (12.6 mg, 0.050 mmol, 1.0 equiv.). Product yield was analyzed by gas chromatography using anisole as an internal standard.

Run Number: (yield of **2a** in mmol, yield of **2a** percent) (yield of **2a’** in mmol, yield of **2a’** percent)

Run 1: (0.0225 mmol, 45%) (0.0028 mmol, 6%)

<u>Run 2</u>: (0.0246 mmol, 49%) (0.0028 mmol, 6%)
Average: 53% yield (89:11)

Entry 3: 4,7-dimethyl-1,10-phenanthroline General procedure for ligand studies was followed. 0.125 mL of the catalyst stock solution (containing CuCl (0.05 mg, 0.500 µmol, 0.01 equiv.)) 0.125 mL ligand stock solution (containing dmp (0.10 mg, 0.500 µmol, 0.01 equiv.)), *(2E,4E)*-5-(4-fluorophenyl)penta-2,4-dien-1-yl methoxycarbamate (**1a**) (12.6 mg, 0.050 mmol, 1.0 equiv.). Product yield was analyzed by gas chromatography using anisole as an internal standard.

<u>Run Number:</u> (yield of **2a** in mmol, yield of **2a** percent) (yield of **2a’** in mmol, yield of **2a’** percent)

<u>Run 1</u>: (0.0106 mmol, 21%) (0.00315 mmol, 6%)
<u>Run 2</u>: (0.009 mmol, 18%) (0.004 mmol, 8%)
Average: 27% yield (73:27)

Entry 4: 2,2’-bipyridine General procedure for ligand studies was followed. 0.125 mL of the catalyst stock solution (containing CuCl (0.05 mg, 0.500 µmol, 0.01 equiv.)) 0.125 mL ligand stock solution (containing bpy (0.08 mg, 0.500 µmol, 0.01 equiv.)), *(2E,4E)*-5-(4-fluorophenyl)penta-2,4-dien-1-yl methoxycarbamate (**1a**) (12.6 mg, 0.050 mmol, 1.0 equiv.). Product yield was analyzed by gas chromatography using anisole as an internal standard.

<u>Run Number:</u> (yield of **2a** in mmol, yield of **2a** percent) (yield of **2a’** in mmol, yield of **2a’** percent)

<u>Run 1</u>: (0.0121 mmol, 24%) (0.002 mmol, 4%)
<u>Run 2</u>: (0.0125 mmol, 25%) (0.0012 mmol, 2%)
Average: 28% yield (88:12)

Entry 5: 6,6’-dimethyl-2,2’-bipyridine General procedure for ligand studies was followed. 0.125 mL of the catalyst stock solution (containing CuCl (0.05 mg, 0.500 µmol, 0.01 equiv.)) 0.125 mL ligand stock solution (containing bpy (0.09 mg, 0.500 µmol, 0.01 equiv.)), *(2E,4E)*-5-(4-fluorophenyl)penta-2,4-dien-1-yl methoxycarbamate (**1a**) (12.6 mg, 0.050 mmol, 1.0 equiv.). Product yield was analyzed by gas chromatography using anisole as an internal standard.

<u>Run Number:</u> (yield of **2a** in mmol, yield of **2a** percent) (yield of **2a’** in mmol, yield of **2a’** percent)

<u>Run 1</u>: (0.02245 mmol, 45%) (0.0045 mmol, 9%)
<u>Run 2</u>: (0.024 mmol, 48%) (0.002 mmol, 4%)
Average: 53% yield (88:12)

Entry 6: 4,4'-dimethyl-2,2'-bipyridine General procedure for ligand studies was followed. 0.125 mL of the catalyst stock solution (containing CuCl (0.05 mg, 0.500 µmol, 0.01 equiv.)) 0.125 mL ligand stock solution (containing bpy (0.09 mg, 0.500 µmol, 0.01 equiv.)), *(2E,4E)*-5-(4-fluorophenyl)penta-2,4-dien-1-yl methoxycarbamate (**1a**) (12.6 mg, 0.050 mmol, 1.0 equiv.). Product yield was analyzed by gas chromatography using anisole as an internal standard.

<u>Run Number:</u> (yield of **2a** in mmol, yield of **2a** percent) (yield of **2a'** in mmol, yield of **2a'** percent)

<u>Run 1</u>: (0.0197 mmol, 39%) (0.0044 mmol, 9%)
<u>Run 2</u>: (0.019 mmol, 38%) (0.0035 mmol, 7%)
Average: 47% yield (83:17)

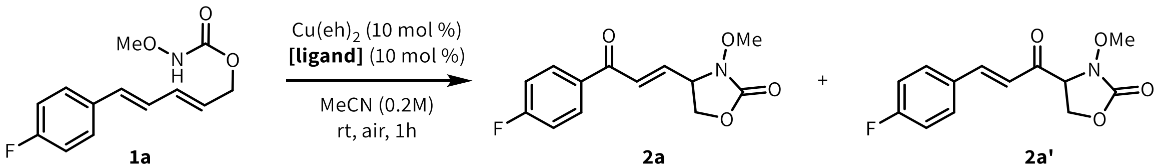

Table S3. Ligand studies with $Cu(eh)_2$.

Entry	Ligand	Yield 2a	Yield 2a'	Aldehyde
1	none	1	3	14
2	dmp	6	40	24
3	4,7-dimethyl-2,9-phenanthroline	1	2	3
4	bipyridine	1	4	5
5	6,6′-dimethyl-2,2′-bipyridine	5	34	25
6	4,4′-dimethyl-2,2′-bipyridine	1	2	3

General procedure for ligand studies using $Cu(eh)_2$:
<u>$Cu(eh)_2$ stock solution:</u> Copper 2-ethylhexanoate (175 mg, 0.500 mmol, 1.0 equiv.) was weighed into a 20 mL volumetric flask in a glovebox. The vial was sealed, removed from the glovebox and the solids were taken up in MeCN (12.5 mL, 0.04 M) and stirred until homogenous (15 min).
<u>Ligand stock solution:</u> Ligand (0.500 mmol, 1.0 equiv.) was weighed into a 20 mL volumetric flask in a glovebox. The vial was sealed, removed from the glovebox and the solids were taken up in MeCN (12.5 mL, 0.04 M) and stirred until homogenous (15 min).
<u>Reaction:</u> 0.125 mL of the catalyst stock solution (containing $Cu(eh)_2$ (1.8 mg, 5.0 µmol, 0.1 equiv.)) and 0.125 mL ligand stock solution (containing 5.0 µmol, 0.1 equiv.) was transferred to an uncapped, flame-dried 1 dram vial equipped with a stir bar. *(2E,4E)*-5-(4-fluorophenyl)penta-2,4-dien-1-yl methoxycarbamate (**1a**) (12.6 mg, 0.050 mmol, 1.0 equiv.) was added in one portion and the reaction mixture was stirred at 500 rpm for 1h in the uncapped vial. 20.0 µL of the reaction mixture was filtered through a ~2 cm pad of

SiO_2 in a Pasteur pipette and eluting with ~1 mL EtO_2. The crude residue was analyzed by gas chromatography using anisole (2.5 µL, 0.023 mmol, 0.46 equiv.) as an internal standard.

Entry 1: no ligand General procedure for ligand studies was followed. 0.125 mL of the catalyst stock solution (containing $Cu(eh)_2$ (1.8 mg, 5.0 µmol, 0.1 equiv.)) 0.125 mL ligand stock solution (containing MeCN blank), *(2E,4E)*-5-(4-fluorophenyl)penta-2,4-dien-1-yl methoxycarbamate (**1a**) (12.6 mg, 0.050 mmol, 1.0 equiv.). Product yield was analyzed by gas chromatography using anisole as an internal standard.

Run Number: (yield of **2a** in mmol, yield of **2a** percent) (yield of **2a'** in mmol, yield of **2a'** percent)

Run 1: (0.0005 mmol, 1%) (0.002 mmol, 4%)
Run 2: (*ND, ND*) (0.001 mmol, 2%)
Average: 4% yield (14:86)

Entry 2: dmp General procedure for ligand studies was followed. 0.125 mL of the catalyst stock solution (containing $Cu(eh)_2$ (1.8 mg, 5.0 µmol, 0.1 equiv.)) 0.125 mL ligand stock solution (containing dmp (1.0 mg, 5.0 µmol, 0.1 equiv.)), *(2E,4E)*-5-(4-fluorophenyl)penta-2,4-dien-1-yl methoxycarbamate (**1a**) (12.6 mg, 0.050 mmol, 1.0 equiv.). Product yield was analyzed by gas chromatography using anisole as an internal standard.

Run Number: (yield of **2a** in mmol, yield of **2a** percent) (yield of **2a'** in mmol, yield of **2a'** percent)

Run 1: (0.0039 mmol, 8%) (0.0196 mmol, 39%)
Run 2: (0.025 mmol, 5%) (0.0209 mmol, 42%)
Average: 47% yield (14:86)

Entry 3: 4,7-dimethyl-1,10-phenanthroline General procedure for ligand studies was followed. 0.125 mL of the catalyst stock solution (containing $Cu(eh)_2$ (1.8 mg, 5.0 µmol, 0.1 equiv.)) 0.125 mL ligand stock solution (containing 4,7-dimethyl-1,10-phenanthroline (1.0 mg, 5.0 µmol, 0.1 equiv.)), *(2E,4E)*-5-(4-fluorophenyl)penta-2,4-dien-1-yl methoxycarbamate (**1a**) (12.6 mg, 0.050 mmol, 1.0 equiv.). Product yield was analyzed by gas chromatography using anisole as an internal standard.

Run Number: (yield of **2a** in mmol, yield of **2a** percent) (yield of **2a'** in mmol, yield of **2a'** percent)

Run 1: (*ND, ND*) (0.0005 mmol, 1%)
Run 2: (0.005 mmol, 1%) (0.0015 mmol, 3%)
Average: 3% yield (20:80)

Entry 4: 2,2'-bipyridine General procedure for ligand studies was followed. 0.125 mL of the catalyst stock solution (containing $Cu(eh)_2$ (1.8 mg, 5.0 µmol, 0.1 equiv.)) 0.125 mL

ligand stock solution (containing bpy (0.8 mg, 5.0 μmol, 0.1 equiv.)), *(2E,4E)*-5-(4-fluorophenyl)penta-2,4-dien-1-yl methoxycarbamate (**1a**) (12.6 mg, 0.050 mmol, 1.0 equiv.). Product yield was analyzed by gas chromatography using anisole as an internal standard.

Run Number: (yield of **2a** in mmol, yield of **2a** percent) (yield of **2a'** in mmol, yield of **2a'** percent)

Run 1: (0.0005 mmol, 1%) (0.002 mmol, 4%)
Run 2: (0.0005 mmol, 1%) (0.0015mmol, 3%)
Average: 5% yield (22:78)

Entry 5: 6,6'-dimethyl-2,2'-bipyridine General procedure for ligand studies was followed. 0.125 mL of the catalyst stock solution (containing $Cu(eh)_2$ (1.8 mg, 5.0 μmol, 0.1 equiv.)) 0.125 mL ligand stock solution (containing 6,6-dimethyl-2,2-bipyridine (0.9 mg, 5.0 μmol, 0.1 equiv.)), *(2E,4E)*-5-(4-fluorophenyl)penta-2,4-dien-1-yl methoxycarbamate (**1a**) (12.6 mg, 0.050 mmol, 1.0 equiv.). Product yield was analyzed by gas chromatography using anisole as an internal standard.

Run Number: (yield of **2a** in mmol, yield of **2a** percent) (yield of **2a'** in mmol, yield of **2a'** percent)

Run 1: (0.0015 mmol, 3%) (0.017 mmol, 34%)
Run 2: (0.003 mmol, 6%) (0.017 mmol, 34%)
Average: 39% yield (12:88)

Entry 5: 4,4'-dimethyl-2,2'-bipyridine General procedure for ligand studies was followed. 0.125 mL of the catalyst stock solution (containing $Cu(eh)_2$ (1.8 mg, 5.0 μmol, 0.1 equiv.)) 0.125 mL ligand stock solution (containing 4,4-dimethyl-2,2-bipyridine (0.9 mg, 5.0 μmol, 0.1 equiv.)), *(2E,4E)*-5-(4-fluorophenyl)penta-2,4-dien-1-yl methoxycarbamate (**1a**) (12.6 mg, 0.050 mmol, 1.0 equiv.). Product yield was analyzed by gas chromatography using anisole as an internal standard.

Run Number: (yield of **2a** in mmol, yield of **2a** percent) (yield of **2a'** in mmol, yield of **2a'** percent)

Run 1: (*ND, ND*) (0.0005 mmol, 1%)
Run 2: (0.0005 mmol, 1%) (0.001 mmol, 2%)
Average: 2% yield (25:75)

Base and Solvent Screening

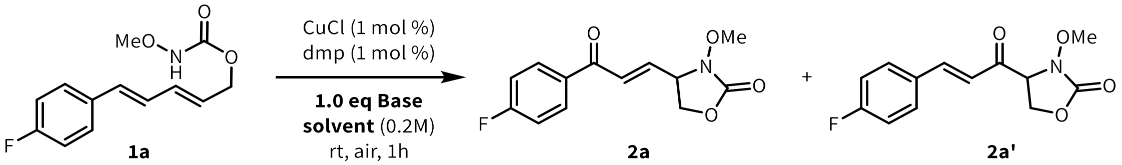

Table S4. Base and solvent screening.

Entry	Additive	Solvent	Yield 2a	Yield 2a'	Aldehyde
		**Base Screening**			
**1**	none	MeCN	47	6	6
**2**	Na_2CO_3	MeCN	45	8	5
**3**	K_2CO_3	MeCN	43	8	5
**4**	Cs_2CO_3	MeCN	15	*ND*	5
**5**	KOAc	MeCN	51	10	7
**6**	DTBP	MeCN	45	8	4
		**Solvent Screening**			
**7**	none	MeCN	47	6	6
**8**	none	DMF	49	5	3.5
**9**	none	DCM	40	5	4.5
**10**	none	EtOAc	4	25	17.5
**11**	none	PhMe	<1	9	13.5
**12**	none	H_2O	*ND*	*ND*	*ND*

General procedure for additive studies with CuCl:
Catalyst stock solution: CuCl (2.5 mg, 0.025 mmol, 1.0 equiv.) and dmp (5.2 mg, 0.025 mmol, 1.0 equiv.) was weighed into a 20 mL scintillation vial in a glovebox. The vial was sealed, removed from the glovebox and the solids were taken up in MeCN (6.25 mL, 4.0mM) and stirred until homogenous (15 min).
Reaction: 0.25 mL of the catalyst stock solution (containing CuCl (0.05 mg, 0.500 μmol, 0.010 equiv.) and dmp (0.10 mg, 0.500 μmol, 0.01 equiv.) was transferred to an uncapped, flame-dried 1 dram vial equipped with a stir bar. *(2E,4E)*-5-(4-fluorophenyl)penta-2,4-dien-1-yl methoxycarbamate (**1a**) (12.6 mg, 0.050 mmol, 1.0 equiv.), and the additive being screened (0.050 mmol, 1.0 equiv.), were added in one portion and the reaction mixture was stirred at 500 rpm for 1h in the uncapped vial. 20.0 μL of the reaction mixture was filtered through a ~2 cm pad of SiO_2 in a Pasteur pipette and eluting with ~1 mL EtO_2. The crude residue was analyzed by gas chromatography using anisole (2.5 μL, 0.023 mmol, 0.46 equiv.) as an internal standard.

Entry 1: No Additive General procedure for additive studies was followed. 0.25 mL of the catalyst stock solution (containing CuCl (0.05 mg, 0.500 μmol, 0.010 equiv.) and dmp (0.10 mg, 0.500 μmol, 0.01 equiv.)) was transferred to an uncapped, flame-dried 1 dram vial equipped with a stir bar. *(2E,4E)*-5-(4-fluorophenyl)penta-2,4-dien-1-yl methoxycarbamate (**1a**) (12.6 mg, 0.050 mmol, 1.0 equiv.) was used. Product yield was analyzed by gas chromatography using anisole as an internal standard.

Run Number: (yield of **2a** in mmol, yield of **2a** percent) (yield of **2a'** in mmol, yield of **2a'** percent)

Run 1: (0.0225 mmol, 45%) (0.003 mmol, 6%)
Run 2: (0.0245 mmol, 49%) (0.003 mmol, 6%)
Average: 47%, 6%

Entry 2: Na_2CO_3 General procedure for additive studies was followed. 0.25 mL of the catalyst stock solution (containing CuCl (0.05 mg, 0.500 μmol, 0.010 equiv.) and dmp (0.10 mg, 0.500 μmol, 0.01 equiv.)) was transferred to an uncapped, flame-dried 1 dram vial equipped with a stir bar. *(2E,4E)*-5-(4-fluorophenyl)penta-2,4-dien-1-yl methoxycarbamate (**1a**) (12.6 mg, 0.050 mmol, 1.0 equiv.), Na_2CO_3 (5.3 mg, 0.05 mmol, 1.0 equiv.). Product yield was analyzed by gas chromatography using anisole as an internal standard.

Run Number: (yield of **2a** in mmol, yield of **2a** percent) (yield of **2a'** in mmol, yield of **2a'** percent)

Run 1: (0.0235 mmol, 47%) (0.0055 mmol, 11%)
Run 2: (0.0210 mmol, 42%) (0.0025 mmol, 5%)
Average: 45%, 8%

Entry 3: K_2CO_3 General procedure for additive studies was followed. 0.25 mL of the catalyst stock solution (containing CuCl (0.05 mg, 0.500 μmol, 0.010 equiv.) and dmp (0.10 mg, 0.500 μmol, 0.01 equiv.)) was transferred to an uncapped, flame-dried 1 dram vial equipped with a stir bar. *(2E,4E)*-5-(4-fluorophenyl)penta-2,4-dien-1-yl methoxycarbamate (**1a**) (12.6 mg, 0.050 mmol, 1.0 equiv.), K_2CO_3 (6.9 mg, 0.05 mmol, 1.0 equiv.). Product yield was analyzed by gas chromatography using anisole as an internal standard.

Run Number: (yield of **2a** in mmol, yield of **2a** percent) (yield of **2a'** in mmol, yield of **2a'** percent)

Run 1: (0.021 mmol, 42%) (0.0050 mmol, 10%)
Run 2: (0.022 mmol, 44%) (0.0025 mmol, 5%)
Average: 43%, 8%

Entry 4: Cs_2CO_3 General procedure for additive studies was followed. 0.25 mL of the catalyst stock solution (containing CuCl (0.05 mg, 0.500 μmol, 0.010 equiv.) and dmp (0.10 mg, 0.500 μmol, 0.01 equiv.)) was transferred to an uncapped, flame-dried 1 dram vial equipped with a stir bar. *(2E,4E)*-5-(4-fluorophenyl)penta-2,4-dien-1-yl methoxycarbamate (**1a**) (12.6 mg, 0.050 mmol, 1.0 equiv.), Cs_2CO_3 (16.3 mg, 0.05 mmol, 1.0 equiv.). Product yield was analyzed by gas chromatography using anisole as an internal standard.

Run Number: (yield of **2a** in mmol, yield of **2a** percent) (yield of **2a'** in mmol, yield of **2a'** percent)

Run 1: (0.007 mmol, 14%) (ND)
Run 2: (0.008 mmol, 16%) (ND)
Average: 15%, *ND*

Entry 5: KOAc General procedure for additive studies was followed. 0.25 mL of the catalyst stock solution (containing CuCl (0.05 mg, 0.500 μmol, 0.010 equiv.) and dmp (0.10 mg, 0.500 μmol, 0.01 equiv.)) was transferred to an uncapped, flame-dried 1 dram vial equipped with a stir bar. *(2E,4E)*-5-(4-fluorophenyl)penta-2,4-dien-1-yl methoxycarbamate (**1a**) (12.6 mg, 0.050 mmol, 1.0 equiv.), KOAc (4.9 mg, 0.05 mmol, 1.0 equiv.). Product yield was analyzed by gas chromatography using anisole as an internal standard.

Run Number: (yield of **2a** in mmol, yield of **2a** percent) (yield of **2a'** in mmol, yield of **2a'** percent)

Run 1: (0.026 mmol, 52%) (0.0045 mmol, 9%)
Run 2: (0.025 mmol, 50%) (0.0055 mmol, 11%)
Average: 51%, 10%

Entry 6: DTBP General procedure for additive studies was followed. 0.25 mL of the catalyst stock solution (containing CuCl (0.05 mg, 0.500 μmol, 0.010 equiv.) and dmp (0.10 mg, 0.500 μmol, 0.01 equiv.)) was transferred to an uncapped, flame-dried 1 dram vial equipped with a stir bar. *(2E,4E)*-5-(4-fluorophenyl)penta-2,4-dien-1-yl methoxycarbamate (**1a**) (12.6 mg, 0.050 mmol, 1.0 equiv.), DTBP (7.3 mg, 0.05 mmol, 1.0 equiv.). Product yield was analyzed by gas chromatography using anisole as an internal standard.

Run Number: (yield of **2a** in mmol, yield of **2a** percent) (yield of **2a'** in mmol, yield of **2a'** percent)

Run 1: (0.0225 mmol, 45%) (0.004 mmol, 8%)
Run 2: (0.0225 mmol, 45%) (0.0035 mmol, 7%)
Average: 45%, 8%

General procedure for solvent optimization studies:
Catalyst stock solution: CuCl (2.5 mg, 0.025 mmol, 1.0 equiv.) and dmp (5.2 mg, 0.025 mmol, 1.0 equiv.) was weighed into a 20 mL scintillation vial in a glovebox. The vial was sealed, removed from the glovebox and the solids were taken up in the desired solvent (6.25 mL, 4.0mM) and stirred until homogenous (15 min).
Reaction: 0.25 mL of the catalyst stock solution (containing CuCl (0.05 mg, 0.500 μmol, 0.010 equiv.) and dmp (0.10 mg, 0.500 μmol, 0.01 equiv.) was transferred to an uncapped, flame-dried 1 dram vial equipped with a stir bar. *(2E,4E)*-5-(4-fluorophenyl)penta-2,4-dien-1-yl methoxycarbamate (**1a**) (12.6 mg, 0.050 mmol, 1.0

equiv.) was added in one portion and the reaction mixture was stirred at 500 rpm for 1h in the uncapped vial. 20.0 µL of the reaction mixture was filtered through a ~2 cm pad of SiO_2 in a Pasteur pipette and eluting with ~1 mL EtO_2. The crude residue was analyzed by gas chromatography using anisole (2.5 µL, 0.023 mmol, 0.46 equiv.) as an internal standard.

Entry 7: MeCN General procedure for additive studies was followed. 0.25 mL of the catalyst stock solution (containing CuCl (0.05 mg, 0.500 µmol, 0.010 equiv.) and dmp (0.10 mg, 0.500 µmol, 0.01 equiv.) was transferred to an uncapped, flame-dried 1 dram vial equipped with a stir bar. *(2E,4E)*-5-(4-fluorophenyl)penta-2,4-dien-1-yl methoxycarbamate (**1a**) (12.6 mg, 0.050 mmol, 1.0 equiv.). Product yield was analyzed by gas chromatography using anisole as an internal standard.

Run Number: (yield of **2a** in mmol, yield of **2a** percent) (yield of **2a'** in mmol, yield of **2a'** percent)

Run 1: (0.0225 mmol, 45%) (0.003 mmol, 6%)
Run 2: (0.0245 mmol, 49%) (0.003 mmol, 6%)
Average: 47%, 6%

Entry 8: DMF General procedure for additive studies was followed. 0.25 mL of the catalyst stock solution (containing CuCl (0.05 mg, 0.500 µmol, 0.010 equiv.) and dmp (0.10 mg, 0.500 µmol, 0.01 equiv.) was transferred to an uncapped, flame-dried 1 dram vial equipped with a stir bar. *(2E,4E)*-5-(4-fluorophenyl)penta-2,4-dien-1-yl methoxycarbamate (**1a**) (12.6 mg, 0.050 mmol, 1.0 equiv.). Product yield was analyzed by gas chromatography using anisole as an internal standard.

Run Number: (yield of **2a** in mmol, yield of **2a** percent) (yield of **2a'** in mmol, yield of **2a'** percent)

Run 1: (0.0245 mmol, 49%) (0.0025 mmol, 5%)
Run 2: (0.024 mmol, 48%) (0.002 mmol, 4%)
Average: 49%, 5%

Entry 9: DCM General procedure for additive studies was followed. 0.25 mL of the catalyst stock solution (containing CuCl (0.050 mg, 0.500 µmol, 0.010 equiv.) and dmp (0.10 mg, 0.500 µmol, 0.01 equiv.) was transferred to an uncapped, flame-dried 1 dram vial equipped with a stir bar. *(2E,4E)*-5-(4-fluorophenyl)penta-2,4-dien-1-yl methoxycarbamate (**1a**) (12.6 mg, 0.050 mmol, 1.0 equiv.). Product yield was analyzed by gas chromatography using anisole as an internal standard.

Run Number: (yield of **2a** in mmol, yield of **2a** percent) (yield of **2a'** in mmol, yield of **2a'** percent)

Run 1: (0.021 mmol, 42%) (0.002 mmol, 4%)
Run 2: (0.0185 mmol, 37%) (0.0025 mmol, 5%)

Average: 40%, 5%

Entry 10: EtOAc General procedure for additive studies was followed. 0.25 mL of the catalyst stock solution (containing CuCl (0.05 mg, 0.500 µmol, 0.010 equiv.) and dmp (0.10 mg, 0.500 µmol, 0.01 equiv.) was transferred to an uncapped, flame-dried 1 dram vial equipped with a stir bar. *(2E,4E)*-5-(4-fluorophenyl)penta-2,4-dien-1-yl methoxycarbamate (**1a**) (12.6 mg, 0.050 mmol, 1.0 equiv.). Product yield was analyzed by gas chromatography using anisole as an internal standard.

Run Number: (yield of **2a** in mmol, yield of **2a** percent) (yield of **2a'** in mmol, yield of **2a'** percent)

Run 1: (0.0015 mmol, 3%) (0.0125 mmol, 25%)
Run 2: (0.002 mmol, 4%) (0.0120 mmol, 24%)
Average: 4%, 25%

Entry 11: PhMe General procedure for additive studies was followed. 0.25 mL of the catalyst stock solution (containing CuCl (0.05 mg, 0.500 µmol, 0.010 equiv.) and dmp (0.10 mg, 0.500 µmol, 0.01 equiv.) was transferred to an uncapped, flame-dried 1 dram vial equipped with a stir bar. *(2E,4E)*-5-(4-fluorophenyl)penta-2,4-dien-1-yl methoxycarbamate (**1a**) (12.6 mg, 0.050 mmol, 1.0 equiv.). Product yield was analyzed by gas chromatography using anisole as an internal standard.

Run Number: (yield of **2a** in mmol, yield of **2a** percent) (yield of **2a'** in mmol, yield of **2a'** percent)

Run 1: (<0.001 mmol, <1%) (0.0035 mmol, 7%)
Run 2: (<0.001 mmol, <1%) (0.0055 mmol, 11%)
Average: <1%, 9%

Entry 12: H_2O General procedure for additive studies was followed. 0.25 mL of the catalyst stock solution (containing CuCl (0.05 mg, 0.500 µmol, 0.010 equiv.) and dmp (0.10 mg, 0.500 µmol, 0.01 equiv.) was transferred to an uncapped, flame-dried 1 dram vial equipped with a stir bar. *(2E,4E)*-5-(4-fluorophenyl)penta-2,4-dien-1-yl methoxycarbamate (**1a**) (12.6 mg, 0.050 mmol, 1.0 equiv.). Product yield was analyzed by gas chromatography using anisole as an internal standard.

Run Number: (yield of **2a** in mmol, yield of **2a** percent) (yield of **2a'** in mmol, yield of **2a'** percent)

Run 1: *(ND) (ND)*
Run 2: *(ND) (ND)*
Average: *ND, ND*

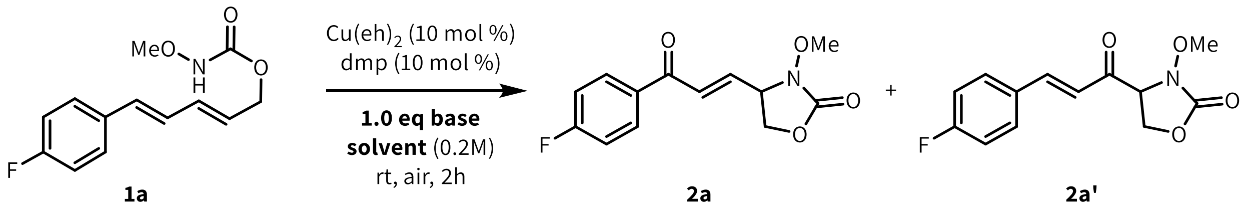

Table S5. Base and solvent screening.

Entry	Additive	Solvent	Yield 2a	Yield 2a’	Aldehyde
		**Base Screening**			
**1**	none	MeCN	12.5	37	22
**2**	Na_2CO_3	MeCN	17	39.5	22
**3**	K_2CO_3	MeCN	11	22	25
**4**	Cs_2CO_3	MeCN	19	ND	5
**5**	KOAc	MeCN	18.5	23	24
**6**	DTBP	MeCN	13	26	24
		**Solvent Screening**			
**7**	none	MeCN	13.5	37	21
**8**	none	DMF	11	39	22
**9**	none	DCM	7.5	11	30
**10**	none	EtOAc	9	38.5	9
**11**	none	PhMe	2	12	24
**12**	none	H_2O	ND	ND	34

General procedure for additive studies with $Cu(eh)_2$:

Catalyst stock solution: $Cu(eh)_2$ (175 mg, 0.500 mmol, 1.0 equiv.) and dmp (104 mg, 0.500 mmol, 1.0 equiv.) were weighed into a scintillation vial in a glovebox. The vial was sealed was removed from the glovebox and the solids were taken up in MeCN (12.5 mL, 40.0 mM) and stirred at rt for 15 min as the vial is exposed to air.

Starting material stock solution: *(2E,4E)*-5-(4-fluorophenyl)penta-2,4-dien-1-yl methoxycarbamate (**1a**) (302 mg, 1.20 mmol, 1.0 equiv.) were taken up in MeCN (3.0 mL, 0.4 M) and stirred until homogenous.

Reaction: 0.125 mL of the catalyst stock solution (containing $Cu(eh)_2$ (1.8 mg, 5.0 µmol, 0.10 equiv.), and dmp (1.0 mg, 5.0 µmol, 0.1 equiv.) was transferred to an uncapped, flame-dried 1 dram vial equipped with a stir bar. 0.125 mL of the starting material stock solution (containing 12.6 mg, 0.050 mmol, 1.0 equiv. *(2E,4E)*-5-(4-fluorophenyl)penta-2,4-dien-1-yl methoxycarbamate (**1a**) and the additive being screened (0.050 mmol, 1.0 equiv.), were added and the reaction mixture was stirred at 500 rpm for 2h in the uncapped vial. 20.0 µL of the reaction mixture was filtered through a ~2 cm pad of SiO_2 in a Pasteur pipette and eluting with ~1 mL EtO_2. The crude residue was analyzed by gas chromatography using anisole (2.5 µL, 0.023 mmol, 0.46 equiv.) as an internal standard.

Entry 1: No Additive General procedure for additive studies was followed. 0.125 mL of the catalyst stock solution (containing Cu(eh)$_2$ (1.8 mg, 5.0 μmol, 0.10 equiv.), and dmp (1.0 mg, 5.0 μmol, 0.1 equiv.) was transferred to an uncapped, flame-dried 1 dram vial equipped with a stir bar. 0.125 mL of the starting material stock solution (containing 12.6 mg, 0.050 mmol, 1.0 equiv. *(2E,4E)*-5-(4-fluorophenyl)penta-2,4-dien-1-yl methoxycarbamate (**1a**) was added. Product yield was analyzed by gas chromatography using anisole as an internal standard.

Run Number: (yield of **2a** in mmol, yield of **2a** percent) (yield of **2a'** in mmol, yield of **2a'** percent)

Run 1: (0.006 mmol, 12%) (0.019 mmol, 38%)
Run 2: (0.0065mmol, 13%) (0.018 mmol, 36%)
Average: 12.5%, 37%

Entry 2: Na_2CO_3 General procedure for additive studies was followed. 0.125 mL of the catalyst stock solution (containing Cu(eh)$_2$ (1.8 mg, 5.0 μmol, 0.10 equiv.), and dmp (1.0 mg, 5.0 μmol, 0.1 equiv.) was transferred to an uncapped, flame-dried 1 dram vial equipped with a stir bar. 0.125 mL of the starting material stock solution (containing 12.6 mg, 0.050 mmol, 1.0 equiv. *(2E,4E)*-5-(4-fluorophenyl)penta-2,4-dien-1-yl methoxycarbamate (**1a**) was added. Product yield was analyzed by gas chromatography using anisole as an internal standard.

Run Number: (yield of **2a** in mmol, yield of **2a** percent) (yield of **2a'** in mmol, yield of **2a'** percent)

Run 1: (0.009 mmol, 18%) (0.02 mmol, 40%)
Run 2: (0.0075 mmol, 15%) (0.0195 mmol, 39%)
Average: 17%, 39.5%

Entry 3: K_2CO_3 General procedure for additive studies was followed. 0.125 mL of the catalyst stock solution (containing Cu(eh)$_2$ (1.8 mg, 5.0 μmol, 0.10 equiv.), and dmp (1.0 mg, 5.0 μmol, 0.1 equiv.) was transferred to an uncapped, flame-dried 1 dram vial equipped with a stir bar. 0.125 mL of the starting material stock solution (containing 12.6 mg, 0.050 mmol, 1.0 equiv. *(2E,4E)*-5-(4-fluorophenyl)penta-2,4-dien-1-yl methoxycarbamate (**1a**) was added. Product yield was analyzed by gas chromatography using anisole as an internal standard.

Run Number: (yield of **2a** in mmol, yield of **2a** percent) (yield of **2a'** in mmol, yield of **2a'** percent)

Run 1: (0.0055 mmol, 11%) (0.00105 mmol, 21%)
Run 2: (0.0055 mmol, 11%) (0.00115 mmol, 23%)
Average: 11%, 22%

Entry 4: Cs_2CO_3 General procedure for additive studies was followed. 0.125 mL of the catalyst stock solution (containing Cu(eh)$_2$ (1.8 mg, 5.0 μmol, 0.10 equiv.), and dmp (1.0

mg, 5.0 µmol, 0.1 equiv.) was transferred to an uncapped, flame-dried 1 dram vial equipped with a stir bar. 0.125 mL of the starting material stock solution (containing 12.6 mg, 0.050 mmol, 1.0 equiv. *(2E,4E)*-5-(4-fluorophenyl)penta-2,4-dien-1-yl methoxycarbamate (**1a**) was added. Product yield was analyzed by gas chromatography using anisole as an internal standard.

Run Number: (yield of **2a** in mmol, yield of **2a** percent) (yield of **2a'** in mmol, yield of **2a'** percent)

Run 1: (0.0105 mmol, 21%) (ND)
Run 2: (0.008 mmol, 16%) (ND)
Average: 19%, *ND*

Entry 5: KOAc General procedure for additive studies was followed. 0.125 mL of the catalyst stock solution (containing $Cu(eh)_2$ (1.8 mg, 5.0 µmol, 0.10 equiv.), and dmp (1.0 mg, 5.0 µmol, 0.1 equiv.) was transferred to an uncapped, flame-dried 1 dram vial equipped with a stir bar. 0.125 mL of the starting material stock solution (containing 12.6 mg, 0.050 mmol, 1.0 equiv. *(2E,4E)*-5-(4-fluorophenyl)penta-2,4-dien-1-yl methoxycarbamate (**1a**) was added. Product yield was analyzed by gas chromatography using anisole as an internal standard.

Run Number: (yield of **2a** in mmol, yield of **2a** percent) (yield of **2a'** in mmol, yield of **2a'** percent)

Run 1: (0.009 mmol, 18%) (0.0105 mmol, 21%)
Run 2: (0.0095 mmol, 19%) (0.0125 mmol, 25%)
Average: 18.5%, 23%

Entry 6: DTBP General procedure for additive studies was followed. 0.125 mL of the catalyst stock solution (containing $Cu(eh)_2$ (1.8 mg, 5.0 µmol, 0.10 equiv.), and dmp (1.0 mg, 5.0 µmol, 0.1 equiv.) was transferred to an uncapped, flame-dried 1 dram vial equipped with a stir bar. 0.125 mL of the starting material stock solution (containing 12.6 mg, 0.050 mmol, 1.0 equiv. *(2E,4E)*-5-(4-fluorophenyl)penta-2,4-dien-1-yl methoxycarbamate (**1a**) was added. Product yield was analyzed by gas chromatography using anisole as an internal standard.

Run Number: (yield of **2a** in mmol, yield of **2a** percent) (yield of **2a'** in mmol, yield of **2a'** percent)

Run 1: (0.0075 mmol, 15%) (0.013 mmol, 26%)
Run 2: (0.005 mmol, 10%) (0.0125 mmol, 25%)
Average: 13%, 26%

General procedure for solvent optimization studies:

Catalyst stock solution: Cu(eh)$_2$ (175 mg, 0.500 mmol, 1.0 equiv.) and dmp (104 mg, 0.500 mmol, 1.0 equiv.) were weighed into a 25 mL volumetric flask in a glovebox. The vial was sealed was removed from the glovebox and the solids were taken up in the solvent to be screened (25 mL, 2.0 mM) and stirred at rt for 15 min as the vial is exposed to air.

Starting material stock solution: *(2E,4E)*-5-(4-fluorophenyl)penta-2,4-dien-1-yl methoxycarbamate (**1a**) (302 mg, 1.20 mmol, 1.0 equiv.) were taken up in MeCN (3.0 mL, 0.4 M) and stirred until homogenous.

Reaction: 0.25 mL of the catalyst stock solution (containing Cu(eh)$_2$ (1.8 mg, 5.0 μmol, 0.10 equiv.), and dmp (1.0 mg, 5.0 μmol, 0.1 equiv.) was transferred to an uncapped, flame-dried 1 dram vial equipped with a stir bar. 0.125 mL of the starting material stock solution (containing 12.6 mg, 0.050 mmol, 1.0 equiv. *(2E,4E)*-5-(4-fluorophenyl)penta-2,4-dien-1-yl methoxycarbamate (**1a**)) and the additive being screened (0.05 mmol, 1.0 equiv.), were added sequentially and the reaction mixture was stirred at 500 rpm for 2h in the uncapped vial. 20.0 μL of the reaction mixture was filtered through a ~2 cm pad of SiO_2 in a Pasteur pipette and eluting with ~1 mL EtO_2. The crude residue was analyzed by gas chromatography using anisole (2.5 μL, 0.023 mmol, 0.46 equiv.) as an internal standard.

Entry 7: MeCN General procedure for solvent optimization screening was followed. 0.125 mL of the catalyst stock solution (containing Cu(eh)$_2$ (1.8 mg, 5.0 μmol, 0.10 equiv.), and dmp (1.0 mg, 5.0 μmol, 0.1 equiv.) was transferred to an uncapped, flame-dried 1 dram vial equipped with a stir bar. 0.125 mL of the starting material stock solution (containing 12.6 mg, 0.050 mmol, 1.0 equiv. (*2E,4E*)-5-(4-fluorophenyl)penta-2,4-dien-1-yl methoxycarbamate (**1a**) was added. Product yield was analyzed by gas chromatography using anisole as an internal standard.

Run Number: (yield of **2a** in mmol, yield of **2a** percent) (yield of **2a’** in mmol, yield of **2a’** percent)

Run 1: (0.007 mmol, 14%) (0.019 mmol, 38%)
Run 2: (0.0065mmol, 13%) (0.018 mmol, 36%)
Average: 13.5%, 37%

Entry 8: DMF General procedure for solvent optimization screening was followed. 0.125 mL of the catalyst stock solution (containing Cu(eh)$_2$ (1.8 mg, 5.0 μmol, 0.10 equiv.), and dmp (1.0 mg, 5.0 μmol, 0.1 equiv.) was transferred to an uncapped, flame-dried 1 dram vial equipped with a stir bar. 0.125 mL of the starting material stock solution (containing 12.6 mg, 0.050 mmol, 1.0 equiv. *(2E,4E)*-5-(4-fluorophenyl)penta-2,4-dien-1-yl methoxycarbamate (**1a**) was added. Product yield was analyzed by gas chromatography using anisole as an internal standard.

Run Number: (yield of **2a** in mmol, yield of **2a** percent) (yield of **2a’** in mmol, yield of **2a’** percent)

Run 1: (0.005 mmol, 10%) (0.0185 mmol, 37%)
Run 2: (0.006 mmol, 12%) (0.0205 mmol, 41%)
Average: 11%, 39%

Entry 9: DCM General procedure for solvent optimization screening was followed. 0.125 mL of the catalyst stock solution (containing $Cu(eh)_2$ (1.8 mg, 5.0 µmol, 0.10 equiv.), and dmp (1.0 mg, 5.0 µmol, 0.1 equiv.) was transferred to an uncapped, flame-dried 1 dram vial equipped with a stir bar. 0.125 mL of the starting material stock solution (containing 12.6 mg, 0.050 mmol, 1.0 equiv. *(2E,4E)*-5-(4-fluorophenyl)penta-2,4-dien-1-yl methoxycarbamate (**1a**) was added. Product yield was analyzed by gas chromatography using anisole as an internal standard.

Run Number: (yield of **2a** in mmol, yield of **2a** percent) (yield of **2a'** in mmol, yield of **2a'** percent)

Run 1: (0.0025 mmol, 5%) (0.0045 mmol, 9%)
Run 2: (0.005 mmol, 10%) (0.0065 mmol, 13%)
Average: 7.5%, 11%

Entry 10: EtOAc General procedure for solvent optimization screening was followed. 0.125 mL of the catalyst stock solution (containing $Cu(eh)_2$ (1.8 mg, 5.0 µmol, 0.10 equiv.), and dmp (1.0 mg, 5.0 µmol, 0.1 equiv.) was transferred to an uncapped, flame-dried 1 dram vial equipped with a stir bar. 0.125 mL of the starting material stock solution (containing 12.6 mg, 0.050 mmol, 1.0 equiv. *(2E,4E)*-5-(4-fluorophenyl)penta-2,4-dien-1-yl methoxycarbamate (**1a**) was added. Product yield was analyzed by gas chromatography using anisole as an internal standard.

Run Number: (yield of **2a** in mmol, yield of **2a** percent) (yield of **2a'** in mmol, yield of **2a'** percent)

Run 1: (0.0045 mmol, 9%) (0.0195 mmol, 39%)
Run 2: (0.0045 mmol, 9%) (0.019 mmol, 38%)
Average: 9%, 38.5%

Entry 11: PhMe General procedure for solvent optimization screening was followed. 0.125 mL of the catalyst stock solution (containing $Cu(eh)_2$ (1.8 mg, 5.0 µmol, 0.10 equiv.), and dmp (1.0 mg, 5.0 µmol, 0.1 equiv.) was transferred to an uncapped, flame-dried 1 dram vial equipped with a stir bar. 0.125 mL of the starting material stock solution (containing 12.6 mg, 0.050 mmol, 1.0 equiv. *(2E,4E)*-5-(4-fluorophenyl)penta-2,4-dien-1-yl methoxycarbamate (**1a**) was added. Product yield was analyzed by gas chromatography using anisole as an internal standard.

Run Number: (yield of **2a** in mmol, yield of **2a** percent) (yield of **2a'** in mmol, yield of **2a'** percent)

Run 1: (0.001 mmol, 2%) (0.006 mmol, 12%)
Run 2: (0.001 mmol, 2%) (0.0055 mmol, 11%)
Average: 2%, 12%

Entry 12: H_2O General procedure for solvent optimization screening was followed. 0.125 mL of the catalyst stock solution (containing Cu(eh)$_2$ (1.8 mg, 5.0 μmol, 0.10 equiv.), and dmp (1.0 mg, 5.0 μmol, 0.1 equiv.) was transferred to an uncapped, flame-dried 1 dram vial equipped with a stir bar. 0.125 mL of the starting material stock solution (containing 12.6 mg, 0.050 mmol, 1.0 equiv. *(2E,4E)*-5-(4-fluorophenyl)penta-2,4-dien-1-yl methoxycarbamate (**1a**) was added. Product yield was analyzed by gas chromatography using anisole as an internal standard.

Run Number: (yield of **2a** in mmol, yield of **2a** percent) (yield of **2a'** in mmol, yield of **2a'** percent)

Run 1: *(ND) (ND)*
Run 2: *(ND) (ND)*
Average: *ND, ND*

Counterion Control Experiments

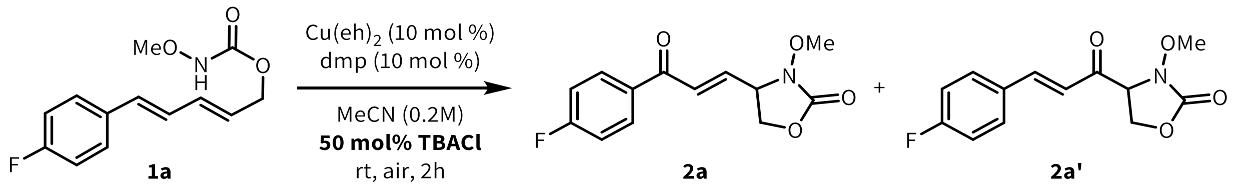

Catalyst stock solution: Cu(eh)$_2$ (175 mg, 0.500 mmol, 1.0 equiv.) and dmp (104 mg, 0.500 mmol, 1.0 equiv.) were weighed into a 25 mL volumetric flask in a glovebox. The vial was sealed was removed from the glovebox and the solids were taken up in MeCN (25 mL, 2.0 mM) and stirred at rt for 15 min as the vial is exposed to air.
Reaction: 1.5 mL of the catalyst stock solution (containing Cu(eh)$_2$ (10.5 mg, 0.030 mmol, 0.10 equiv.) and dmp (6.2 mg, 0.030 mmol, 0.10 equiv.) was transferred to an uncapped, flame-dried 20 mL scintillation vial equipped with a stir bar. Tetrabutylammonium chloride (TBACl) (42.7 mg, 0.150 mmol, 0.5 equiv.), *(2E,4E)-5-(*4-fluorophenyl)penta-2,4-dien-1-yl methoxycarbamate (**1a**) (75.4 mg, 0.300 mmol) were added sequentially and the reaction mixture was stirred at 500 rpm for 2h in the uncapped vial. The reaction was quenched by filtering through a ~2 cm pad of SiO_2 in a Pasteur pipette and eluting with ~10 mL EtOAc. The filtrate was concentrated in vacuo and the crude residue was purified by flash chromatography on SiO_2 using 30% EtOAc in hexanes, unless otherwise noted.

Run: (**2a**), ***(2a')***
Run 1: (45.7 mg, 0.172 mmol, 57%), *(ND)*
Run 2: (46.0 mg, 0.1.73 mmol, 58%), *(ND)*
Run 3 (qNMR): (44.5 mg 0.168 mmol, 56%), *(ND)*

Average: 57% yield (>99:1)

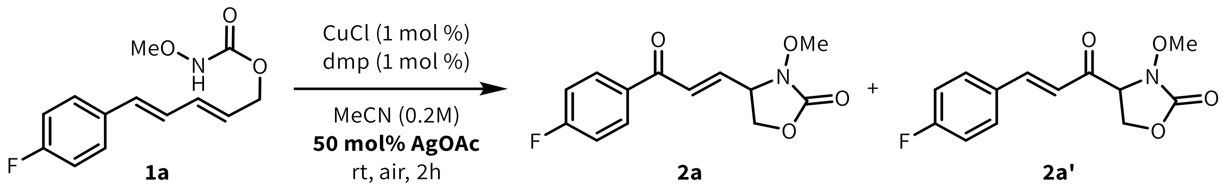

General Procedure B: Catalyst stock solution: CuCl (5.0 mg, 0.050 mmol, 1.0 equiv.) and dmp (10.4 mg, 0.050 mmol, 1.0 equiv.) were weighed into a 25 mL volumetric flask in a glovebox. The vial was sealed was removed from the glovebox and the solids were taken up in MeCN (25 mL, 2.0 mM) and stirred at rt for 15 min as the vial is exposed to air.
Reaction: 2.5 mL of the catalyst stock solution (containing CuCl (0.5 mg, 5.0 µmol, 0.010 equiv.) and dmp (1.04 mg, 5.0 µmol, 0.01 equiv.) was transferred to an uncapped, flame-dried 20 mL scintillation vial equipped with a stir bar. *(2E,4E)*-5-(4-fluorophenyl)penta-2,4-dien-1-yl methoxycarbamate (**1a**) (125.6 mg, 0.500 mmol) and silver acetate (41.7 mg, 0.250 mmol, 0.5 equiv.) were added sequentially and the reaction mixture was stirred at 500 rpm for 1h in the uncapped vial. The reaction was quenched by filtering through a ~2 cm pad of SiO_2 in a Pasteur pipette and eluting with ~10 mL EtOAc. The filtrate was concentrated in vacuo and the crude residue was purified by flash chromatography on SiO_2 using 30% EtOAc in hexanes, unless otherwise noted.

Run: (**1a**), (**2a**), *(2a')*
Run 1: (28.1 mg, 0.11 mmol, 23%), (6.7 mg, 0.025 mmol, 5%), *(28.7 mg, 0.108 mmol, 22%)*
Run 2: (28.3 mg, 0.11 mmol, 23%), (6.0 mg, 0.023 mmol, 5%), *(28.3 mg, 0.107 mmol, 21%)*
Run 3 (qNMR): (26.4 mg, 0.105 mmol, 21%) (6.6 mg, 0.025 mmol, 5%), *(29.2 mg, 0.11 mmol, 22%)*
Average: 27% yield (19:81)

Reaction Kinetics Experiments

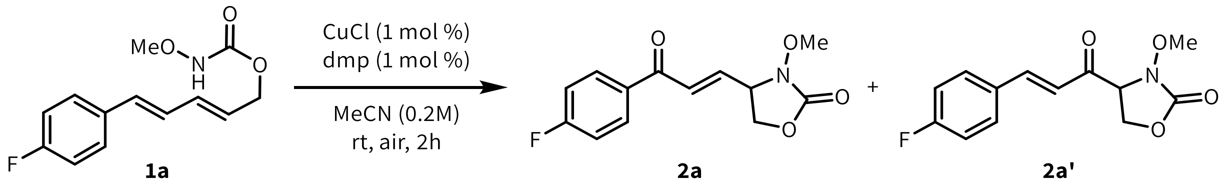

Catalyst stock solution: CuCl (5.0 mg, 0.05 mmol, 1.0 equiv.) and dmp (10.4 mg, 0.05 mmol, 1.0 equiv.) were weighed into a 25 mL volumetric flask in a glovebox. The vial was sealed was removed from the glovebox and the solids were taken up in MeCN (25 mL, 2.0 mM) and stirred at rt for 15 min as the vial is exposed to air.

Reaction: 1.25 mL of the catalyst stock solution (containing CuCl (0.25 mg, 0.0025 mmol, 0.01 equiv.) and dmp (0.5 mg, 0.0025 mmol, 0.01 equiv.)) was transferred to an uncapped, flame-dried 20 mL scintillation vial equipped with a stir bar. *(2E,4E)-5-(*4-fluorophenyl)penta-2,4-dien-1-yl methoxycarbamate (**1a**) (62.8 mg, 0.25 mmol, 1.0 equiv.) was added and the reaction mixture was stirred at 500 rpm for 1h in the uncapped vial. The reaction was monitored by quantitative ^{19}F NMR using trifluorotoluene (6 μL, 0.0489 mmol, 0.20 equiv.) as an internal standard using ACETONITRILE-D3 as a solvent and a relaxation delay of 5.00 seconds

Table S6. Time point data for CuCl kinetic experiments.

Time (min)	2a (%)	2a' (%)	3a (%)	1a (%)
**0**	0	0	0	98
**1**	2	0	0	55
**3**	6	1	2	43
**6**	13	4	4	39
**9**	18	4	4	27
**12**	25	3	3	22
**15**	28	4	3	16
**20**	38	6	4	12
**30**	50	6	3	5
**60**	50	6	3	5

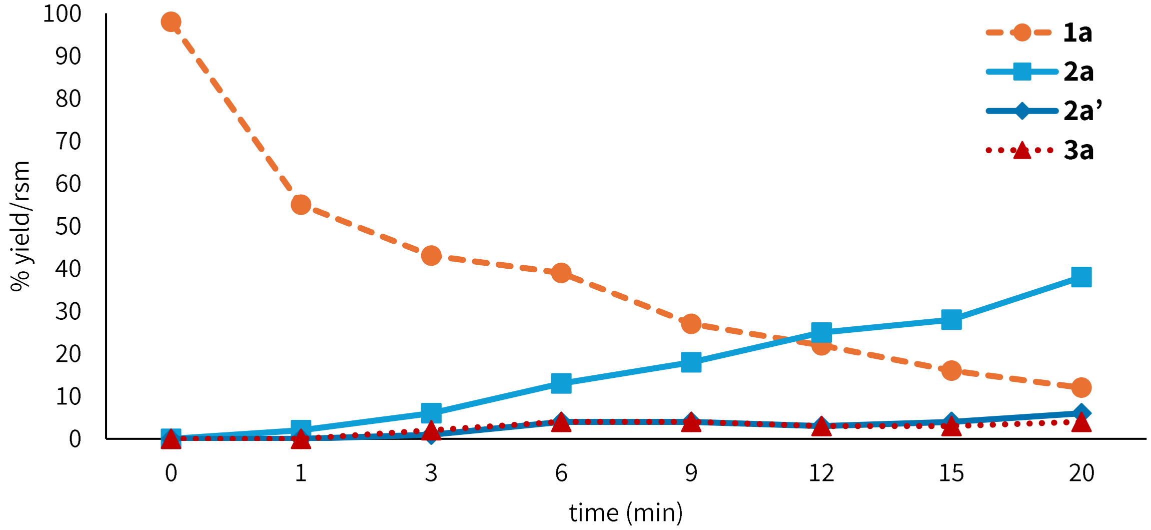

Figure S1. Reaction timecourse under CuCl conditions.

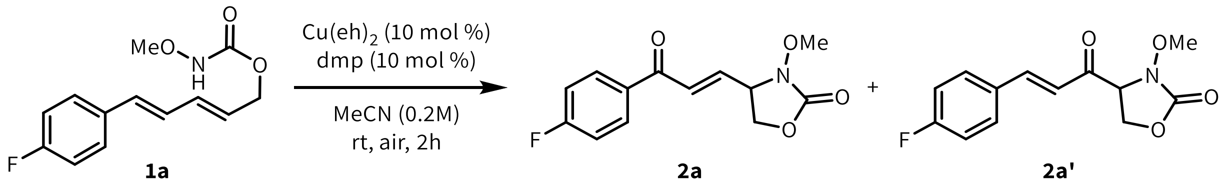

<u>Catalyst stock solution:</u> Cu(eh)$_2$ (175 mg, 0.500 mmol, 1.0 equiv.) and dmp (104 mg, 0.500 mmol, 1.0 equiv.) were weighed into a 25 mL volumetric flask in a glovebox. The vial was sealed was removed from the glovebox and the solids were taken up in MeCN (25 mL, 2.0 mM) and stirred at rt for 15 min as the vial is exposed to air.
<u>Reaction:</u> 1.25 mL of the catalyst stock solution (containing Cu(eh)$_2$ (8.8 mg, 0.025 mmol, 0.10 equiv.) and dmp (5.2 mg, 0.025 mmol, 0.10 equiv.)) was transferred to an uncapped, flame-dried 20 mL scintillation vial equipped with a stir bar. *(2E,4E)-5-*(4-fluorophenyl)penta-2,4-dien-1-yl methoxycarbamate (**1a**) (62.8 mg, 0.25 mmol, 1.0 equiv.) was added and the reaction mixture was stirred at 500 rpm for 1h in the uncapped vial. The reaction was monitored by quantitative ^{19}F NMR using trifluorotoluene (6 μL, 0.0489 mmol, 0.20 equiv.) as an internal standard using ACETONITRILE-D3 as a solvent and a relaxation delay of 5.00 seconds

Table S7. Time point data for Cu(eh)$_2$ kinetic experiments.

Time (min)	1a	2a'	2a	3a
0	95	0	0	0
1	88	0	0	7
3	77	5	2	10
6	54	16	5	15
9	33	27	8	20
12	21	33	9	24
15	9	34	9	25
30	9	44	9	27
60	10	44	10	26

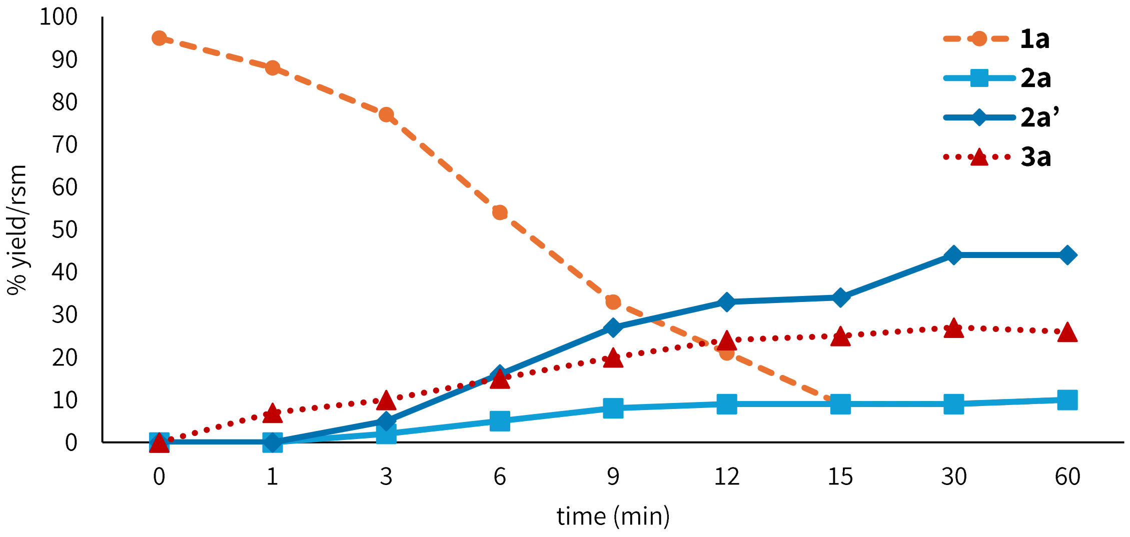

Figure S2. Reaction timecourse under Cu(eh)$_2$ conditions.

Product Interconversion Experiments

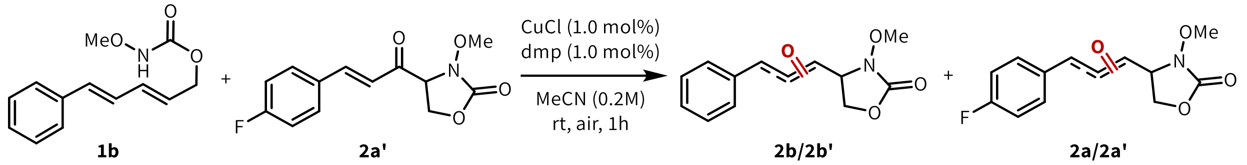

Catalyst stock solution: CuCl (4.95 mg, 0.05 mmol, 1.0 equiv.) and dmp (10.4 mg, 0.05 mmol, 1.0 equiv.) were weighed into a 25 mL volumetric flask in a glovebox. The vial was sealed was removed from the glovebox and the solids were taken up in MeCN (25 mL, 2.0 mM) and stirred at rt for 15 min as the vial is exposed to air.
Reaction: 0.50 mL of the catalyst stock solution (containing CuCl (0.1 mg, 0.001 mmol, 0.010 equiv.) and dmp (0.21 mg, 0.001 mmol, 0.010 equiv.) was transferred to an uncapped, flame-dried 20 mL scintillation vial equipped with a stir bar. *(2E,4E)-5-*phenylpenta-2,4-dien-1-yl methoxycarbamate (**1b**) (23.3 mg, 0.100 mmol, 1.0 equiv.) and (*E*)-4-(3-(4-fluorophenyl)acryloyl)-3-methoxyoxazolidin-2-one (**2a'**) (13.3 mg, 0.050 mmol, 0.5 equiv.) were added sequentially and the reaction mixture was stirred at 500 rpm for 2h in the uncapped vial. The reaction was monitored by quantitative NMR using 1,3,5-trimethoxybenzene as an internal standard.

Run: (**2a**), *(**2a'**)*
Run 1: *ND,* (13.3 mg, 0.05 mmol, 99%)
Run 2: *ND,* (13.3 mg, 0.05 mmol, 99%)
Average: 99% recovery 2a'

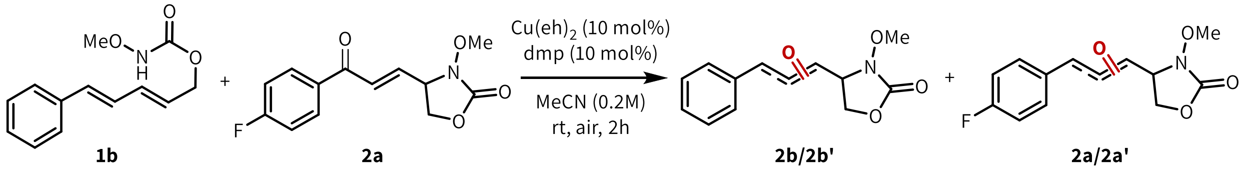

Catalyst stock solution: $Cu(eh)_2$ (175 mg, 0.500 mmol, 1.0 equiv.) and dmp (104 mg, 0.500 mmol, 1.0 equiv.) were weighed into a 25 mL volumetric flask in a glovebox. The vial was sealed was removed from the glovebox and the solids were taken up in MeCN (25 mL, 2.0 mM) and stirred at rt for 15 min as the vial is exposed to air.
Reaction: 0.50 mL of the catalyst stock solution (containing $Cu(eh)_2$ (8.8 mg, 0.010 mmol, 0.10 equiv.) and dmp (5.2 mg, 0.010 mmol, 0.10 equiv.) was transferred to an uncapped, flame-dried 20 mL scintillation vial equipped with a stir bar. *(2E,4E)-5-*phenylpenta-2,4-dien-1-yl methoxycarbamate (**1b**) (23.3 mg, 0.100 mmol, 1.0 equiv.) and (*E*)-4-(3-(4-fluorophenyl)-3-oxoprop-1-en-1-yl)-3-methoxyoxazolidin-2-one (**2a**) (13.3 mg, 0.050 mmol, 0.5 equiv.) were added sequentially and the reaction mixture was stirred at 500 rpm for 2h in the uncapped vial. The reaction was monitored by quantitative NMR using 1,3,5-trimethoxybenzene as an internal standard.

Run: (**2a**), ***(2a')***
Run 1: (13.3 mg, 0.05 mmol, 99%), *ND*
Run 2: (13.3 mg, 0.05 mmol, 99%), *ND*
Average: 99% recovery 2a

Preparation of Precatalysts

Cu(dmp)Cl precatalyst (PC1)

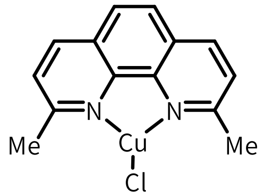

CuCl (49.5 mg, 0.500 mmol, 1.0 equiv.) and dmp (104 mg, 0.500 mmol, 1.0 equiv.) were weighed into a 25 mL volumetric flask in a glovebox with a stir bar. The flask was sealed was removed from the glovebox and the solids were taken up in MeCN (25 mL, 0.2M) and stirred at rt for 60 min as the flask is exposed to air. The S60 flask was left to slowly evaporate to afford precatalyst 1 as red rectangular crystals (154 mg, 0.500 mmol, >99% yield). Spectral data agreed with that reported in literature.[3]

^{1}H NMR (400 MHz, CD_3CN) δ 8.57 (d, *J* = 8.4 Hz, 2H), 8.09 (s, 2H), 7.81 (d, *J* = 8.3 Hz, 2H), 2.40 (s, 6H).

HRMS (ESI) *m/z* calculated for $C_{14}H_{12}ClCuN_2$ $[M+Na]^+$: 328.9877. Found: 328.9880.

Cu(dmp)(eh)$_2$ precatalyst (PC2)

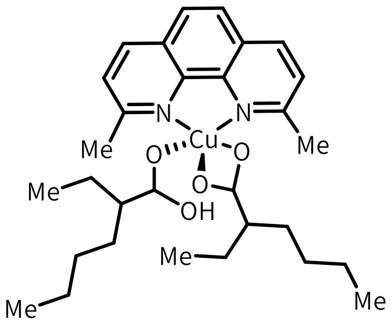

Cu(eh)$_2$ (175 mg, 0.500 mmol, 1.0 equiv.) and dmp (104 mg, 0.500 mmol, 1.0 equiv.) were weighed into a 25 mL volumetric flask in a glovebox with a stir bar. The flask was sealed was removed from the glovebox and the solids were taken up in MeCN (25 mL, 0.2M) and stirred at rt for 60 min as the flask is exposed to air. The flask was left to slowly evaporate to afford precatalyst 2 as green needle crystals (279 mg, 0.500 mmol, >99% yield).
Structural confirmation of **PC2** was obtained by X-ray crystallography.

Table S8. Crystal data and structure refinement for parcm03 (**PC2**).

Identification code	parcm03	
Empirical formula	C30 H42 Cu N2 O4	
Formula weight	558.19	
Temperature	100.00(10) K	
Wavelength	1.54184 Å	
Crystal system	orthorhombic	
Space group	$P2_12_12_1$	
Unit cell dimensions	*a* = 9.76430(10) Å	α = 90°
	b = 21.5508(2) Å	β = 90°
	c = 27.5722(3) Å	γ = 90°

Volume	5801.98(10) Å^3
Z	8
Density (calculated)	1.278 Mg/m^3
Absorption coefficient	1.345 mm^{-1}
F(000)	2376
Crystal color, morphology	green, needle
Crystal size	0.273 x 0.058 x 0.023 mm^3
Theta range for data collection	2.602 to 80.340°
Index ranges	$-12 \le h \le 12$, $-27 \le k \le 20$, $-35 \le l \le 35$
Reflections collected	70225
Independent reflections	12423 [*R*(int) = 0.0461]
Observed reflections	11033
Completeness to theta = 74.504°	99.9%
Absorption correction	Multi-scan
Max. and min. transmission	1.00000 and 0.71703
Refinement method	Full-matrix least-squares on F^2
Data / restraints / parameters	12423 / 493 / 955
Goodness-of-fit on F^2	1.021
Final *R* indices [*I*>2sigma(*I*)]	*R*1 = 0.0472, *wR*2 = 0.1243
R indices (all data)	*R*1 = 0.0546, *wR*2 = 0.1302
Absolute structure parameter	0.50(3)
Largest diff. peak and hole	0.436 and -0.381 e.Å^{-3}

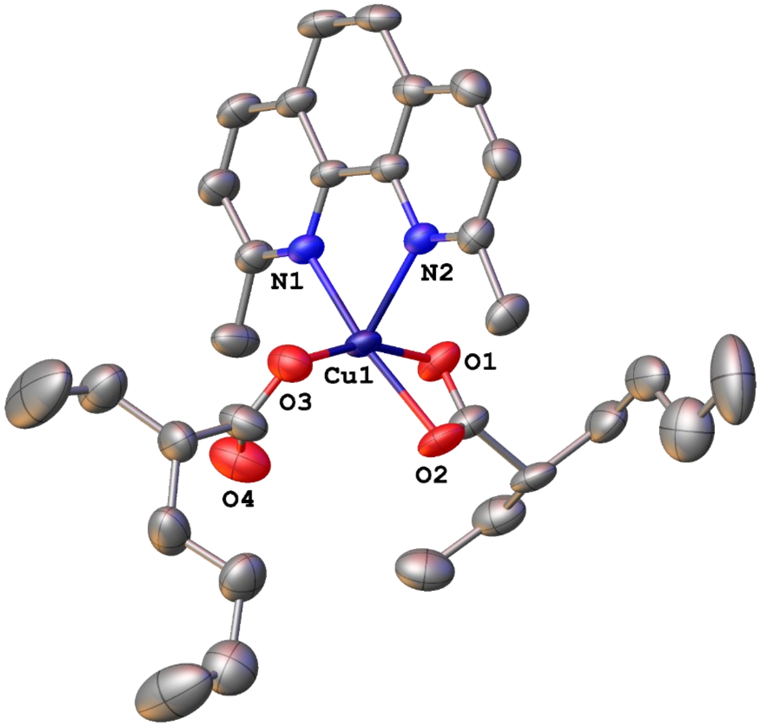

PC2 Structure. Ellipsoids are drawn at the 50 % probability level.

Data collection

A crystal (0.273 x 0.058 x 0.023 mm^3) was placed onto a nylon loop and mounted on a Rigaku XtaLAB Synergy-S Dualflex diffractometer equipped with a HyPix-6000HE HPC area detector for data collection at 100.00(10) K. A preliminary set of cell constants and an orientation matrix were calculated from a small sampling of reflections.[5] A short pre-experiment was run, from which an optimal data collection strategy was determined. The full data collection was carried out using a PhotonJet (Cu) X-ray source with frame times of 4.50 and 18.00 seconds and a detector distance of 34.0 mm. Series of frames were collected in 0.50º steps in *w* at different 2*q*, *k*, and *f* settings. After the intensity data were corrected for absorption, the final cell constants were calculated from the xyz centroids of 32219 strong reflections from the actual data collection after integration.[5]

Structure solution and refinement

The structure was solved using SHELXT[6] and refined using SHELXL.[7] The space group $P2_12_12_1$ was determined based on systematic absences. Most or all non-hydrogen atoms were assigned from the solution. Refinement proceeded in an iterative fashion, with each stage including full-matrix least squares cycles, followed by a difference Fourier synthesis, which located any remaining electron density. All non-hydrogen atoms were refined with anisotropic displacement parameters. All hydrogen atoms were placed in ideal positions and refined as riding atoms with relative isotropic displacement parameters. The final full matrix least squares refinement converged to $R1 = 0.0472$ (F^2, $I > 2s(I)$) and $wR2 = 0.1302$ (F^2, all data).

Structure description

The structure is the one suggested. The asymmetric unit contains two molecules in general positions that appeared to be aligned by offset parallel pi-pi interactions. The -CHEtBu parts of carboxylate ligands O1/O2, O3/O4, and O5/O6 are modeled as disordered over two positions each: 0.70:0.30, 0.65:0.35, and 0.61:0.39, respectively. Carboxylate ligand O7/O8/C53-C60 is modeled as disordered over two positions (0.56:0.44).

Structure manipulation and figure generation were performed using Olex2.[8] Unless noted otherwise all structural diagrams containing anisotropic displacement ellipsoids are drawn at the 50 % probability level.

Data collection, structure solution, and structure refinement were conducted at the X-ray Crystallographic Facility, B04 Hutchison Hall, Department of Chemistry, University of Rochester. The instrument was purchased with funding from NSF MRI program grant CHE-1725028.

UV/Vis Spectra of Reaction Components and Kinetic Data

A 2.0 mM solution of CuCl complex was made by dissolving CuCl (5.0 mg, 0.050 mmol, 1.0 equiv.) and dmp (10.4 mg, 0.050 mmol, 1.0 equiv) in 25.0 mL of dry MeCN into a volumetric flask. A 2.0 mM solution of cinnamyl *N*-methoxy carbamate **1a** was made by

dissolving *(2E,4E)-5-(*4-fluorophenyl)penta-2,4-dien-1-yl methoxycarbamate (**1a**) (5.0 mg, 0.020 mmol, 1.0 equiv.) in 10.0 mL of dry MeCN into a volumetric flask. Samples were made for UV/Vis spectroscopy by dissolving 1.0 mL of each stock solution up to 3.0 mL in a cuvette for 0.667 mM solutions. The spectra were obtained between 200-800 nm, at a scan rate of 480 nm/min over the course of 20 min.

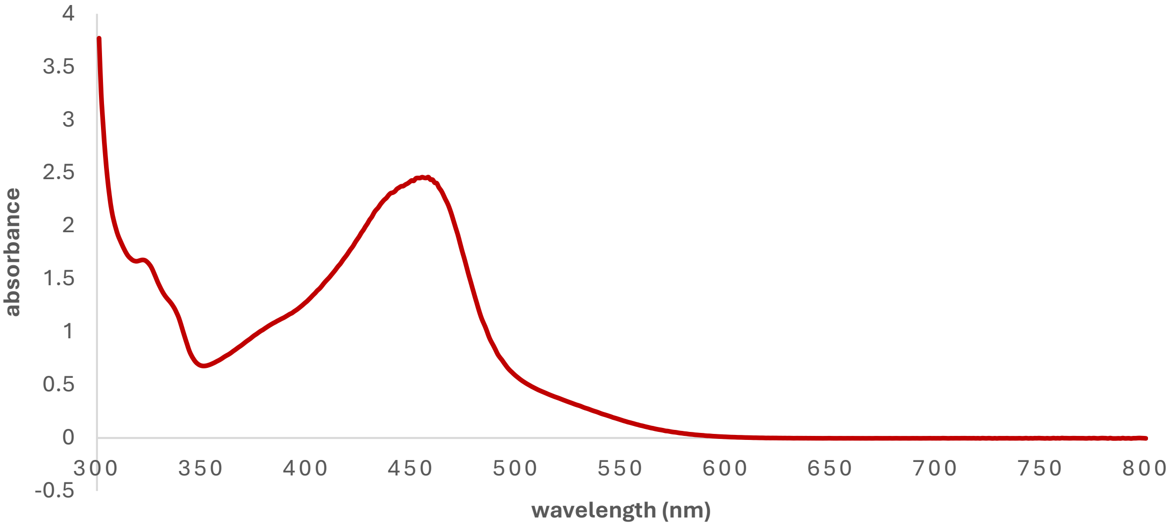

Figure S3. UV-Vis spectrum of CuCl + dmp + **1a**.

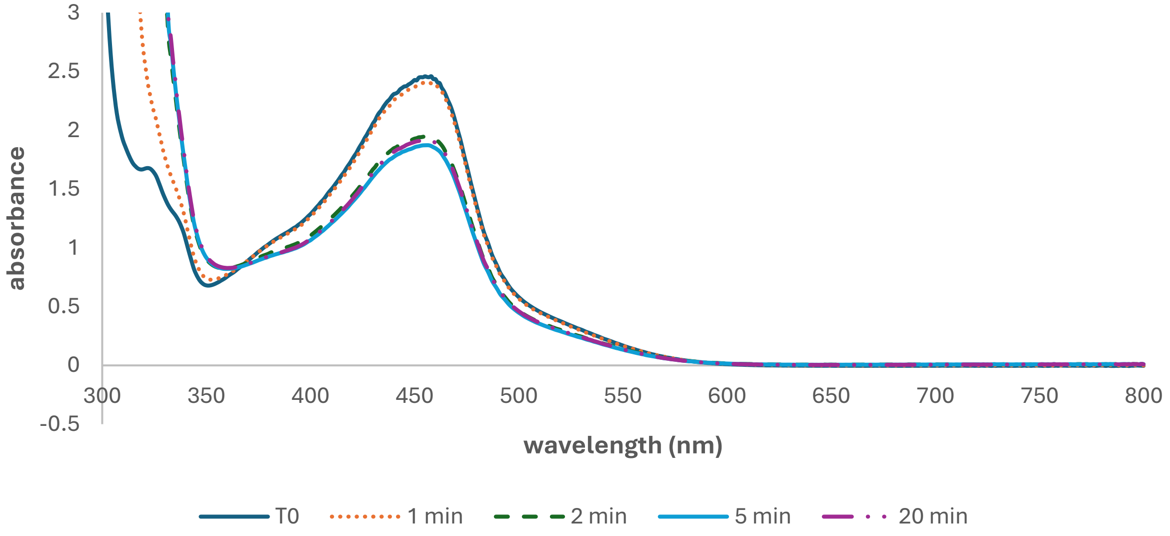

Figure S4. UV-Vis spectrum of above reaction components over time.

A 2.0 mM solution of Cu(eh)$_2$ complex was made by dissolving Cu(eh)$_2$ (7.0 mg, 0.020 mmol, 1.0 equiv.) and dmp (4.2 mg, 0.020 mmol, 1.0 equiv) in 10.0 mL of dry MeCN into a volumetric flask. A 2.0 mM solution of cinnamyl N-methoxy carbamate **1a** was made by dissolving *(2E,4E)-5-(*4-fluorophenyl)penta-2,4-dien-1-yl methoxycarbamate (**1a**) (5.0 mg, 0.020 mmol, 1.0 equiv.) in 10.0 mL of dry MeCN into a volumetric flask. Samples were made for UV/Vis spectroscopy by dissolving 1.0 mL of each stock solution up to 3.0

mL in a cuvette for 0.667 mM solutions. The spectra were obtained between 200-800 nm, at a scan rate of 480 nm/min over the course of 20 min.

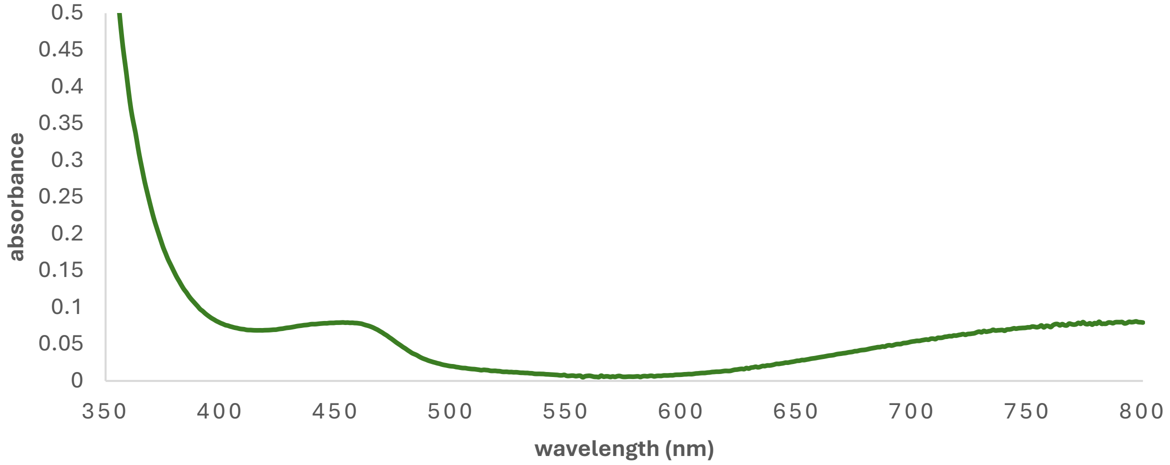

Figure S5. UV-Vis spectrum of $Cu(eh)_2$ + dmp + **1a**.

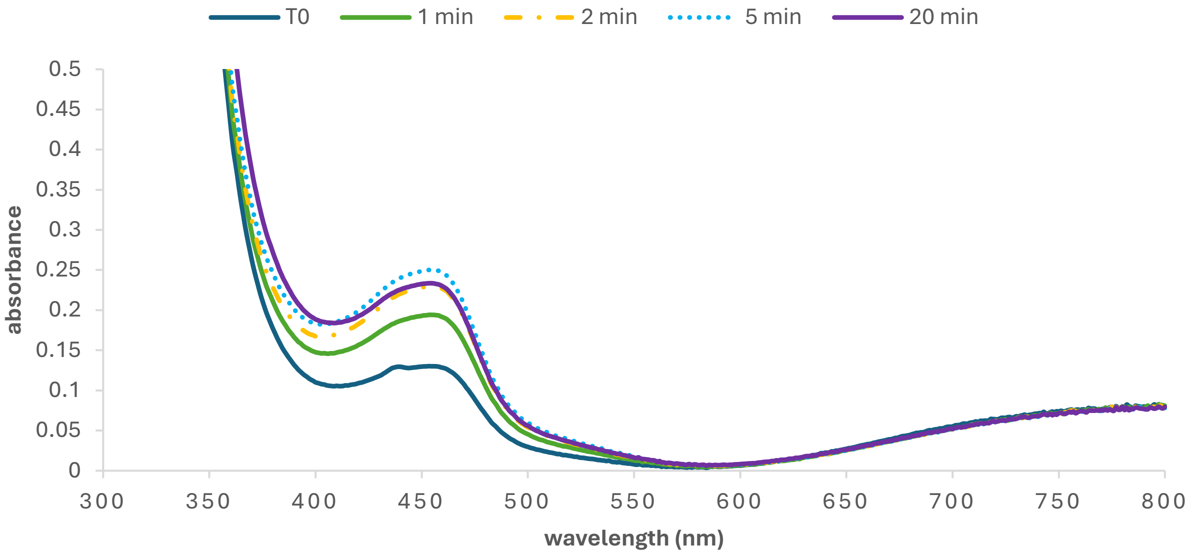

Figure S6. UV-Vis spectrum of above reaction components over time.

Substrate Scope

Reaction Conditions A

<u>Catalyst stock solution:</u> CuCl (5.0 mg, 0.05 mmol, 1.0 equiv.) and dmp (10.4 mg, 0.05 mmol, 1.0 equiv.) were weighed into a 25 mL volumetric flask in a glovebox. The vial was sealed was removed from the glovebox and the solids were taken up in MeCN (25 mL, 2.0 mM) and stirred at rt for 15 min as the vial is exposed to air.
<u>Reaction:</u> 2.5 mL of the catalyst stock solution (containing CuCl (0.5 mg, 5.0 µmol, 0.010 equiv.) and dmp (1.0 mg, 5.0 µmol, 0.01 equiv.)) was transferred to an uncapped, flame-dried 20 mL scintillation vial equipped with a stir bar. 2,4-dien-1-yl *N*-methoxycarbamate (0.500 mmol, 1.0 equiv.) **1** was added in one portion and the reaction mixture was stirred at 500 rpm for 1h in the uncapped vial. The reaction was quenched by filtering through a ~2 cm pad of SiO_2 in a Pasteur pipette and eluting with ~10 mL EtOAc. The filtrate was concentrated in vacuo and the crude residue was purified by flash chromatography on SiO_2 using 30% EtOAc in hexanes, unless otherwise noted.

(*E*)-4-(3-(4-fluorophenyl)-3-oxoprop-1-en-1-yl)-3-methoxyoxazolidin-2-one (2a)

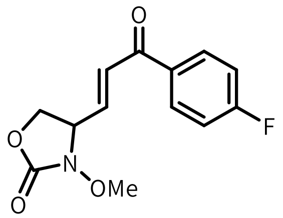

Prepared according to general procedure A: 2.5 mL catalyst stock solution (0.5 mg, 5.0 µmol, 0.010 equiv. CuCl, 1.0 mg, 5.0 µmol, 0.010 equiv. dmp), *(2E,4E)*-5-(4-fluorophenyl)penta-2,4-dien-1-yl methoxycarbamate (**1a**) (125.6 mg, 0.500 mmol) were used. Crude material was purified by flash column chromatography (SiO_2) using a gradient of 30% EtOAc in hexanes to afford pure product as a white solid.

<u>Run:</u> (2a), *(2a')*
<u>Run 1:</u> (70.3 mg, 0.265 mmol, 53%), (*8.5 mg, 0.032 mmol, 6.4%*)
<u>Run 2:</u> (64.9 mg, 0.245 mmol, 49%)
<u>Run 3:</u> (63.8 mg, 0.241 mmol, 48%)
Average: 50% yield
Combined: 56% yield (89:11)

^{1}H NMR (500 MHz, $CDCl_3$) δ 8.02 – 7.94 (m, 2H), 7.22 (d, *J* = 15.4 Hz, 1H), 7.20 – 7.12 (m, 2H), 6.90 (dd, *J* = 15.3, 8.1 Hz, 1H), 4.58 (q, *J* = 8.3 Hz, 1H), 4.44 (t, *J* = 8.4 Hz, 1H), 4.06 (t, *J* = 8.8 Hz, 1H), 3.80 (s, 3H).

^{13}C{^{1}H} NMR (126 MHz, $CDCl_3$) δ 188.0, 167.8, 165.7, 159.1, 140.8, 132.1, 130.9, 116.9, 65.8, 65.2, 61.5.

^{19}F NMR (376 MHz, C_6D_6) δ -104.13.

HRMS (ESI) *m/z* calculated for $C_{13}H_{12}FNO_4$ $[M+H]^+$: 266.0824. Found: 266.0820.

mp. range: 88–89 °C.

(*E*)-3-methoxy-4-(3-oxo-3-phenylprop-1-en-1-yl)oxazolidin-2-one (2b)

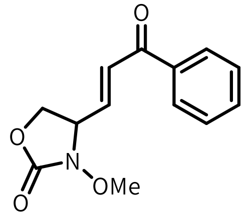

Prepared according to general procedure A: 2.5 mL catalyst stock solution (0.5 mg, 5.0 µmol, 0.010 equiv. CuCl, 1.0 mg, 5.0 µmol, 0.010 equiv. dmp), (2*E*,4*E*)-5-phenylpenta-2,4-dien-1-yl methoxycarbamate (**1b**) (116.6 mg, 0.500 mmol) were used. Crude material was purified by flash column chromatography (SiO_2) using a gradient of 30% EtOAc in hexanes to afford pure product as a white solid.

Run: (2b), *(2b')*
Run 1: (50.4 mg, 0.204 mmol, 41%), (*19.8 mg, 0.08 mmol, 16%*)
Run 2: (55.4 mg, 0.224 mmol, 45%), *(0.067 mmol, 13%)*
Run 3: (54.5 mg, 0.220 mmol, 44%), *(0.066 mmol, 13%)*
Average: 43% yield
Combined: 57% yield (77:23)

^{1}H NMR (500 MHz, $CDCl_3$) δ 8.02 – 7.94 (m, 2H), 7.22 (d, *J* = 15.4 Hz, 1H), 7.20 – 7.12 (m, 2H), 6.90 (dd, *J* = 15.3, 8.1 Hz, 1H), 4.58 (q, *J* = 8.3 Hz, 1H), 4.44 (t, *J* = 8.4 Hz, 1H), 4.06 (t, *J* = 8.8 Hz, 1H), 3.80 (s, 3H).

^{13}C{^{1}H} NMR (126 MHz, $CDCl_3$) δ 188.0, 167.8, 165.7, 159.1, 140.8, 132.1, 130.9, 116.9, 65.8, 65.2, 61.5.

HRMS (ESI) *m/z* calculated for $C_{13}H_{13}NO_4$ $[M+H]^+$: 248.0918. Found: 248.0920.

mp. range: 60–61 °C.

(*E*)-3-methoxy-4-(3-(4-methoxyphenyl)-3-oxoprop-1-en-1-yl)oxazolidin-2-one (2c)

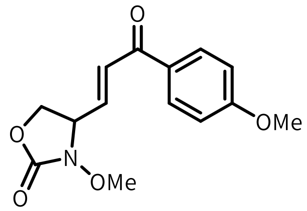

Prepared according to general procedure A: 2.5 mL catalyst stock solution (0.5 mg, 5.0 µmol, 0.010 equiv. CuCl, 1.0 mg, 5.0 µmol, 0.010 equiv. dmp), *(2E,4E)-5-(*4-methoxyphenyl)penta-2,4-dien-1-yl methoxycarbamate (**1c**) (131.6 mg, 0.500 mmol) were used. Crude material was purified by flash column chromatography (SiO_2) using a gradient of 30% EtOAc in hexanes to afford pure product as a white solid.

Run: (2c), *(2c')*
Run 1: (73.5 mg, 0.265 mmol, 53%), (*11.1 mg, 0.040 mmol, 8.0%*)
Run 2: (66.5 mg, 0.240 mmol, 48%)
Run 3: (69.9 mg, 0.252 mmol, 50%)
Average: 50% yield
Combined: 58% yield (86:14)

^{1}H NMR (400 MHz, $CDCl_3$) δ 8.01 – 7.90 (m, 2H), 7.29 – 7.21 (m, 1H), 7.02 – 6.93 (m, 2H), 6.88 (dd, *J* = 15.3, 8.1 Hz, 1H), 4.55 (p, *J* = 8.1 Hz, 1H), 4.43 (t, *J* = 8.3 Hz, 1H), 4.15 – 4.02 (m, 1H), 3.88 (s, 3H), 3.82 (s, 3H).

$^{13}C\{^1H\}$ NMR (101 MHz, $CDCl_3$) δ 187.2, 164.2, 158.6, 139.0, 131.2, 130.7, 129.8, 114.2, 65.2, 64.7, 61.1, 55.7.

HRMS (ESI) *m/z* calculated for $C_{14}H_{15}NO_5$ $[M+H]^+$: 278.1023. Found: 278.1020.

mp. range: 56–57 °C.

(*E*)-3-methoxy-4-(3-oxo-3-(p-tolyl)prop-1-en-1-yl)oxazolidin-2-one (2d)

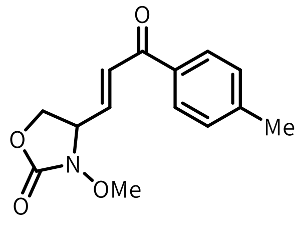

Prepared according to general procedure A: 2.5 mL catalyst stock solution (0.5 mg, 5.0 μmol, 0.010 equiv. CuCl, 1.0 mg, 5.0 μmol, 0.010 equiv. dmp)*,* (2*E*,4*E*)-5-(p-tolyl)penta-2,4-dien-1-yl methoxycarbamate (**1d**) (123.6 mg, 0.500 mmol) were used. Crude material was purified by flash column chromatography (SiO_2) using a gradient of 30% EtOAc in hexanes to afford pure product as a white solid.

Run: (2d), *(2d')*
Run 1: (54.6 mg, 0.209 mmol, 42%), (*7.1 mg, 0.027 mmol, 5.4%*)
Run 2: (60.1 mg, 0.230 mmol, 46%)
Run 3: (58.9 mg, 0.225 mmol, 45%)
Average: 44% yield
Combined: 50% yield (89:11)

^1H NMR (400 MHz, $CDCl_3$) δ 7.89 (d, *J* = 7.9 Hz, 2H), 7.35 – 7.28 (m, 3H), 6.91 (dd, *J* = 15.3, 8.0 Hz, 1H), 4.61 (q, *J* = 8.3 Hz, 1H), 4.46 (t, *J* = 8.3 Hz, 1H), 4.09 (t, *J* = 8.9 Hz, 1H), 3.84 (s, 3H), 2.45 (s, 3H).
$^{13}C\{^1H\}$ NMR (101 MHz, $CDCl_3$) δ 188.5, 158.5, 144.7, 139.4, 134.3, 130.8, 129.6, 128.9, 65.2, 64.6, 60.9, 21.8.

HRMS (ESI) *m/z* calculated for $C_{14}H_{15}NO_4$ $[M+H]^+$: 262.1074. Found: 262.1080.

mp. range: 59–60 °C.

(*E*)-3-methoxy-4-(3-oxo-3-(4-(trifluoromethyl)phenyl)prop-1-en-1-yl)oxazolidin-2-one (2e)

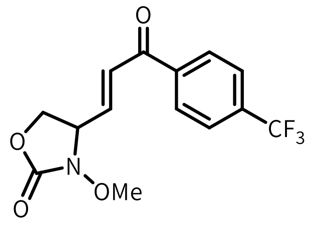

Prepared according to general procedure A: 2.5 mL catalyst stock solution (0.5 mg, 5.0 μmol, 0.010 equiv. CuCl, 1.0 mg, 5.0 μmol, 0.010 equiv. dmp)*,* (2*E*,4*E*)-5-(4-(trifluoromethyl)phenyl) penta-2,4-dien-1-yl methoxycarbamate (**1e**) (150.6 mg, 0.500 mmol) were used. Crude material was purified by flash column chromatography (SiO_2) using a gradient of 30% EtOAc in hexanes to afford pure product as a white solid.

Run: (2e), *(2e')*
Run 1: (16.1 mg, 0.05 mmol, 10%) *(32.7 mg, 0.103 mmol, 21%)*
Run 2: (17.3 mg, 0.055 mmol, 11%)

Run 3: (16.2 mg, 0.05 mmol, 10%)
Average: 10% yield
Combined: 31% yield (33:67)

^{1}H NMR (400 MHz, CHLOROFORM-*D*) δ 8.04 (d, *J* = 8.1 Hz, 2H), 7.75 (d, *J* = 8.2 Hz, 2H), 7.27 – 7.16 (m, 1H), 7.08 – 6.89 (m, 1H), 4.69 – 4.51 (m, 1H), 4.45 (td, *J* = 8.4, 1.2 Hz, 1H), 4.07 (td, *J* = 8.8, 1.1 Hz, 1H), 3.81 (t, *J* = 0.8 Hz, 3H).

^{13}C{^{1}H} NMR (101 MHz, CHLOROFORM-*D*) δ 188.1, 158.4, 144.8, 141.3, 139.6, 135.0, 134.7, 130.1, 129.1, 126.1, 126.0, 65.1, 64.6, 60.8.

^{19}F NMR (376 MHz, $CDCl_3$) δ -63.05.

HRMS (ESI) *m/z* calculated for $C_{14}H_{12}F_3NO_4$ $[M+H]^+$: 316.0794. Found: 316.0792.

mp. range: 48–49 °C.

(*E*)-3-methoxy-4-(3-oxo-3-(*o*-tolyl)prop-1-en-1-yl)oxazolidin-2-one (2f)

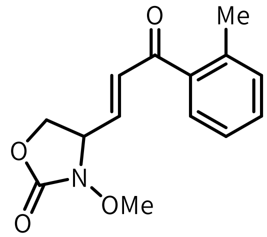

Prepared according to general procedure A: 2.5 mL catalyst stock solution (0.5 mg, 5.0 μmol, 0.010 equiv. CuCl, 1.0 mg, 5.0 μmol, 0.010 equiv. dmp), (2*E*,4*E*)-5-(*o*-tolyl)penta-2,4-dien-1-yl methoxycarbamate (**1f**) (123.6 mg, 0.5 mmol) were used. Crude material was purified by flash column chromatography (SiO_2) using a gradient of 30% EtOAc in hexanes to afford pure product as a clear oil.

Run: (2f), *(2f')*
Run 1: (60.0 mg, 0.230 mmol, 46%), (*27.4 mg, 0.105 mmol, 21%*)
Run 2: (56.1 mg, 0.215 mmol, 43%), (*0.115 mmol, 23%*)
Run 3: (54.8 mg, 0.210 mmol, 42%), *(0.102 mmol, 20%)*
Average: 44% yield
Combined: 65% yield (67:33)

^{1}H NMR (400 MHz, $CDCl_3$) δ 7.53 – 7.47 (m, 1H), 7.41 (t, *J* = 7.4 Hz, 1H), 7.32 – 7.24 (m, 2H), 6.90 (dd, *J* = 15.6, 1.8 Hz, 1H), 6.67 (ddd, *J* = 15.8, 7.9, 1.8 Hz, 1H), 4.54 (q, *J* = 8.3 Hz, 1H), 4.47 – 4.38 (m, 1H), 4.04 (td, *J* = 8.8, 1.8 Hz, 1H), 3.83 (d, *J* = 1.7 Hz, 3H), 2.46 (d, *J* = 1.8 Hz, 3H).

^{13}C{^{1}H} NMR (101 MHz, $CDCl_3$) δ 194.2, 158.8, 140.7, 138.5, 137.7, 135.1, 132.3, 132.0, 129.1, 126.1, 65.5, 65.0, 61.2, 21.1.

HRMS (ESI) *m/z* calculated for $C_{14}H_{15}NO_4$ $[M+H]^+$: 262.1074. Found: 262.1069.

(*E*)-3-methoxy-4-(2-methyl-3-oxo-3-phenylprop-1-en-1-yl)oxazolidin-2-one (2g)

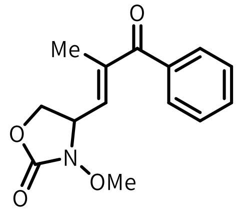

Prepared according to general procedure A: 2.5 mL catalyst stock solution (0.5 mg, 5.0 μmol, 0.010 equiv. CuCl, 1.0 mg, 5.0 μmol, 0.010 equiv. dmp), (2*E*,4*E*)-4-methyl-5-phenylpenta-2,4-dien-1-yl methoxycarbamate (**1g**) (123.6 mg, 0.500 mmol) were used. Crude material was purified by flash column chromatography (SiO_2) using a gradient of 30% EtOAc in hexanes to afford pure product as a white solid.

Run: (2g), *(2g')*
Run 1: (65.7 mg, 0.251 mmol, 50%), *0%*
Run 2: (58.5 mg, 0.224 mmol, 45%), *0%*
Run 3: (60.0 mg, 0.230 mmol, 46%), *0%*
Average: 47% yield
Combined: 47% yield (>99:1)

^{1}H NMR (400 MHz, $CDCl_3$) δ 7.71 (dt, *J* = 7.1, 1.4 Hz, 2H), 7.62 – 7.53 (m, 1H), 7.46 (t, *J* = 7.6 Hz, 2H), 6.11 (dq, *J* = 8.7, 1.5 Hz, 1H), 4.85 (q, *J* = 8.5 Hz, 1H), 4.43 (t, *J* = 8.3 Hz, 1H), 3.94 (t, *J* = 8.8 Hz, 1H), 3.83 (s, 3H), 2.08 (d, *J* = 1.5 Hz, 3H).

^{13}C{^{1}H} NMR (101 MHz, $CDCl_3$) δ 197.6, 158.6, 142.9, 137.0, 135.0, 132.8, 129.6, 128.6, 65.1, 64.6, 57.4, 13.7.

HRMS (ESI) *m/z* calculated for $C_{14}H_{15}NO_4$ $[M+H]^+$: 262.1074. Found: 262.1070.

mp. range: 84–85 °C.

(*Z*)-4-(2-bromo-3-oxo-3-phenylprop-1-en-1-yl)-3-methoxyoxazolidin-2-one (2h)

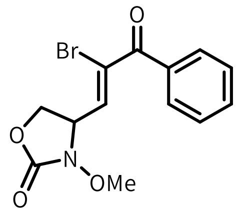

Prepared according to general procedure A: 2.5 mL catalyst stock solution (0.5 mg, 5.0 μmol, 0.010 equiv. CuCl, 1.0 mg, 5.0 μmol, 0.010 equiv. dmp), (2*E*,4*Z*)-4-bromo-5-phenylpenta-2,4-dien-1-yl methoxycarbamate (**1h**) (155.6 mg, 0.500 mmol) were used. Crude material was purified by flash column chromatography (SiO_2) using a gradient of 30% EtOAc in hexanes to afford pure product as a white solid.

Run: (2h), *(2h')*
Run 1: (85.5 mg, 0.262 mmol, 52%), *0%*
Run 2: (83.8 mg, 0.257 mmol, 51%), *0%*
Run 3: (82.2 mg, 0.252 mmol, 50%), *0%*
Average: 51% yield
Combined: 51% yield (>99:1)

^{1}H NMR (400 MHz, $CDCl_3$) δ 7.81 – 7.74 (m, 2H), 7.67 – 7.57 (m, 1H), 7.49 (t, *J* = 7.7 Hz, 3H), 6.82 (d, *J* = 7.4 Hz, 1H), 5.06 (q, *J* = 7.4 Hz, 1H), 4.61 – 4.53 (m, 1H), 4.09 – 4.05 (m, 1H), 3.85 (s, 3H).

$^{13}C\{^1H\}$ NMR (101 MHz, $CDCl_3$) δ 189.2, 158.0, 138.2, 134.9, 133.8, 131.0, 129.9, 129.4, 128.9, 64.4, 59.9.

HRMS (ESI) *m/z* calculated for $C_{13}H_{12}BrNO_4$ $[M+H]^+$: 326.0023. Found: 326.0020.

mp. range: 29–30 °C.

(*E*)-5-methylhexa-2,4-dien-1-yl methoxycarbamate (1i)

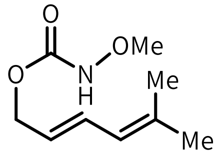

Prepared according to general procedure A: 2.5 mL catalyst stock solution (0.5 mg, 5.0 µmol, 0.010 equiv. CuCl, 1.0 mg, 5.0 µmol, 0.010 equiv. dmp), (*E*)-5-methylhexa-2,4-dien-1-yl methoxycarbamate (**1i**) (92.6 mg, 0.500 mmol) were used.

Run: (2i), *(2i')*
Run 1: (0 mg, 0.0 mmol, 0%), *(0 mg, 0.0 mmol, 0%)*
Run 2: (0 mg, 0.0 mmol, 0%), *(0 mg, 0.0 mmol, 0%)*
Run 3: (0 mg, 0.0 mmol, 0%), *(0 mg, 0.0 mmol, 0%)*
Average: 0% yield
Combined: 0% yield (n/a)

(*E*)-3-methoxy-4-(3-oxobut-1-en-1-yl)oxazolidin-2-one (2j)

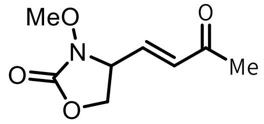

Prepared according to general procedure A: 2.5 mL catalyst stock solution (0.5 mg, 5.0 µmol, 0.010 equiv. CuCl, 1.0 mg, 5.0 µmol, 0.010 equiv. dmp), (2*E*,4*E*)-hexa-2,4-dien-1-yl methoxycarbamate (**1j**) (85.6 mg, 0.500 mmol) were used. Crude material was purified by flash column chromatography (SiO_2) using a gradient of 30% EtOAc in hexanes to afford pure product as a white solid.

Run: (2j), *(2j')*
Run 1: (51.5 mg, 0.278 mmol, 56%), *0%*
Run 2: (49.6 mg, 0.268 mmol, 54%), *0%*
Run 3: (53.0 mg, 0.286 mmol, 57%), *0%*
Average: 55% yield
Combined: 55% yield (>99:1)

^1H NMR (400 MHz, $CDCl_3$) δ 6.66 (dd, *J* = 15.9, 7.6 Hz, 1H), 6.38 (d, *J* = 15.9 Hz, 1H), 4.52 – 4.38 (m, 2H), 4.02 (t, *J* = 8.1 Hz, 1H), 3.79 (s, 3H), 2.31 (s, 3H).

$^{13}C\{^1H\}$ NMR (101 MHz, $CDCl_3$) δ 197.0, 158.4, 138.7, 135.3, 65.1, 64.5, 60.5, 28.0.

HRMS (ESI) *m/z* calculated for $C_8H_{11}NO_4$ $[M+H]^+$: 186.0761. Found: 186.0760

mp. range: 39–40 °C.

(*E*)-4-(3-(furan-2-yl)-3-oxoprop-1-en-1-yl)-3-methoxyoxazolidin-2-one (2k)

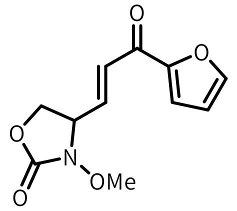

Prepared according to general procedure A: 2.5 mL catalyst stock solution (0.50 mg, 5.0 μmol, 0.010 equiv. CuCl, 1.04 mg, 5.0 μmol, 0.010 equiv. dmp, (2*E*,4*E*)-5-(furan-2-yl)penta-2,4-dien-1-yl methoxycarbamate (**1k**) (111.6 mg, 0.500 mmol) were used. Crude material was purified by flash column chromatography (SiO_2) using a gradient of 30% EtOAc in hexanes to afford pure product as a white solid.

Run: (2k), *(2k')*
Run 1: 0%, *(63.3 mg, 0.239 mmol, 48%)*
Run 2: 0%, *(58.5 mg, 0.221 mmol, 44%)*
Run 3: 0%, *(62.4 mg, 0.265 mmol, 53%)*
Average 1,4-aminooygenation: 0% yield
Combined: 48% yield (<1:99)

(*E*)-3-methoxy-4-(3-oxo-3-(pyridin-2-yl)prop-1-en-1-yl)oxazolidin-2-one (2l)

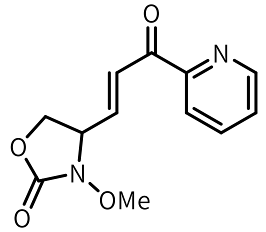

Prepared according to general procedure A: 2.5 mL catalyst stock solution (0.5 mg, 5.0 μmol, 0.010 equiv. CuCl, 1.0 mg, 5.0 μmol, 0.010 equiv. dmp), *(2E,4E)*-5-(pyridin-2-yl)penta-2,4-dien-1-yl methoxycarbamate (**1l**) (117.1 mg, 0.500 mmol) were used. Crude material was purified by flash column chromatography (SiO_2) using a gradient of 50% EtOAc in hexanes to afford pure product as a yellow solid.

Run: (2l), *(2l')*
Run 1: (15.5 mg, 0.063 mmol, 13%), (*46.5 mg, 0.187 mmol, 38%*)
Run 2: (17.1 mg, 0.068 mmol, 14%)
Run 3: (18.7 mg, 0.075 mmol, 15%)
Average: 14% yield
Combined: 52% yield (26:74)

^{1}H NMR (500 MHz, $CDCl_3$) δ 8.71 (d, *J* = 4.8 Hz, 1H), 8.14 (d, *J* = 7.9 Hz, 1H), 7.97 (d, *J* = 15.8 Hz, 1H), 7.88 (t, *J* = 7.6 Hz, 1H), 7.51 (dd, *J* = 7.5, 4.8 Hz, 1H), 7.04 (dd, *J* = 15.7, 8.2 Hz, 1H), 4.60 (q, *J* = 8.4 Hz, 1H), 4.43 (t, *J* = 8.4 Hz, 1H), 4.14 – 4.00 (m, 1H), 3.82 (s, 3H).

^{13}C{^{1}H} NMR (101 MHz, $CDCl_3$) δ 188.4, 153.2, 149.2, 140.0, 137.3, 129.7, 127.6, 123.2, 65.1, 64.7, 61.2.

HRMS (ESI) *m/z* calculated for $C_{12}H_{12}N_2O_4$ $[M+H]^+$: 249.0880. Found: 249.0862.

mp. range: 94–95 °C.

(*E*)-3-methoxy-4-(3-oxo-3-(3-(trifluoromethyl)phenyl)prop-1-en-1-yl)oxazolidin-2-one (2m)

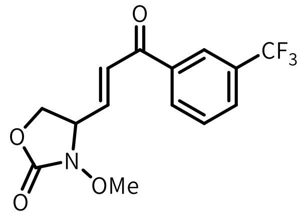

Prepared according to general procedure A: 2.5 mL catalyst stock solution (0.5 mg, 5.0 µmol, 0.010 equiv. CuCl, 1.0 mg, 5.0 µmol, 0.010 equiv. dmp), (2*E*,4*E*)-5-(3-(trifluoromethyl)phenyl) penta-2,4-dien-1-yl methoxycarbamate (**1e**) (150.6 mg, 0.500 mmol) were used. Crude material was purified by flash column chromatography (SiO_2) using a gradient of 30% EtOAc in hexanes to afford pure product as a white solid.

Run: (2m), *(2m')*
Run 1: (80.6 mg, 0.26 mmol, 51%) *(11.0 mg, 0.035 mmol, 7%)*
Run 2: (80.7 mg, 0.26 mmol, 51%)
Run 3: (74.5 mg, 0.24 mmol, 47%)
Average: 50% yield
Combined: 57% yield (88:12)

^{1}H NMR (500 MHz, $CDCl_3$) δ 8.18 (s, 1H), 8.14 (d, 1H), 7.89 – 7.77 (d, 1H), 7.65 (q, *J* = 6.7 Hz, 1H), 7.24 (d, *J* = 15.4 Hz, 1H), 7.06 – 6.91 (m, 1H), 4.61 (q, *J* = 8.3 Hz, 1H), 4.45 (t, *J* = 8.4 Hz, 1H), 4.07 (t, *J* = 8.9 Hz, 1H), 3.81 (s, 3H).

^{13}C{^{1}H} NMR (126 MHz, $CDCl_3$) δ 187.7, 158.4, 141.3, 137.4, 131.9, 131.7, 131.4, 130.0, 130.0, 129.7, 125.5, 65.0, 64.6, 60.8.

^{19}F NMR (376 MHz, $CDCl_3$) δ -63.00.

HRMS (ESI) *m/z* calculated for $C_{14}H_{12}F_3NO_4$ $[M+H]^+$: 316.0792. Found: 316.079.

mp. range: 37–38 °C.

(E)-4-(3-(benzo[d][1,3]dioxol-5-yl)-3-oxoprop-1-en-1-yl)-3-methoxyoxazolidin-2-one (2n)

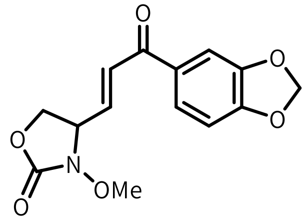

Prepared according to general procedure A: 2.5 mL catalyst stock solution (0.5 mg, 5.0 µmol, 0.010 equiv. CuCl, 1.0 mg, 5.0 µmol, 0.010 equiv. dmp), (2E,4E)-5-(benzo[d][1,3]dioxol-5-yl)penta-2,4-dien-1-yl methoxycarbamate (1n) (138.4 mg, 0.500 mmol) were used. Crude material was purified by flash column chromatography (SiO_2) using a gradient of 30% EtOAc in hexanes to afford pure product as a white solid.

Run: (2n), *(2n')*
Run 1: (62.6 mg, 0.215 mmol, 43%) *(11.8 mg, 0.041 mmol, 8%)*
Run 2: (61.1 mg, 0.21 mmol, 42%)
Run 3: (64.0 mg, 0.22 mmol, 44%)
Average: 43% yield
Combined: 51% yield (84:16)

^{1}H NMR (400 MHz, CHLOROFORM-*D*) δ 7.56 (dd, *J* = 8.1, 1.8 Hz, 1H), 7.45 (d, *J* = 1.8 Hz, 1H), 7.19 (d, *J* = 15.3 Hz, 1H), 6.92 – 6.75 (m, 2H), 6.06 (t, *J* = 1.2 Hz, 2H), 4.54 (q, *J* = 8.3 Hz, 1H), 4.42 (t, *J* = 8.3 Hz, 1H), 4.07 (q, *J* = 10.0 Hz, 1H), 3.81 (t, *J* = 1.2 Hz, 3H).

$^{13}C\{^{1}H\}$ NMR (101 MHz, CHLOROFORM-*D*) δ 186.8, 158.5, 152.6, 148.7, 139.2, 131.7, 130.5, 125.4, 108.4, 108.2, 102.2, 65.2, 64.7, 61.0.

HRMS (ESI) *m/z* calculated for $C_{14}H_{13}NO_6$ $[M+H]^+$: 292.0821. Found: 292.0823.

mp. range: 102–103 °C.

Reaction Conditions B

General Procedure B:
Catalyst stock solution: $Cu(eh)_2$ (175 mg, 0.500 mmol, 1.0 equiv.) and dmp (**3a**) (104 mg, 0.500 mmol, 1.0 equiv.) were weighed into a 25 mL volumetric flask in a glovebox. The vial was sealed was removed from the glovebox and the solids were taken up in MeCN (25 mL, 2.0 mM) and stirred at rt for 15 min as the vial is exposed to air.
Reaction: 1.5 mL of the catalyst stock solution (containing $Cu(eh)_2$ (10.5 mg, 0.030 mmol, 0.10 equiv.) and dmp (**3a**) (6.2 mg, 0.030 mmol, 0.10 equiv.) was transferred to an uncapped, flame-dried 20 mL scintillation vial equipped with a stir bar. 2,4-dien-1-yl *N*-methoxycarbamate (0.300 mmol, 1.0 equiv.) was added in one portion and the reaction mixture was stirred at 500 rpm for 2h in the uncapped vial. The reaction was quenched by filtering through a ~2 cm pad of SiO_2 in a Pasteur pipette and eluting with ~10 mL EtOAc. The filtrate was concentrated in vacuo and the crude residue was purified by flash chromatography on SiO_2 using 30% EtOAc in hexanes, unless otherwise noted.

(*E*)-4-(3-(4-fluorophenyl)acryloyl)-3-methoxyoxazolidin-2-one (2a')

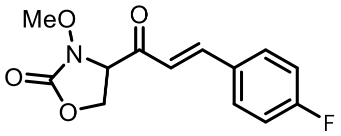

Prepared according to general procedure B: 1.5 mL catalyst stock solution (10.5 mg, 0.030 mmol, 0.10 equiv. $Cu(eh)_2$, 6.2 mg, 0.030 mol, 0.10 equiv. dmp), *(2E,4E)-5-(*4-fluorophenyl)penta-2,4-dien-1-yl methoxycarbamate (**1a**) (75.4 mg, 0.300 mmol) were used. Crude material was purified by flash column chromatography (SiO_2) using a gradient of 30% EtOAc in hexanes to afford pure product as a white solid.

Run: (2a'), *(qNMR 2a), (qNMR cinnamaldehyde)*
Run 1: (35.8 mg, 0.135 mmol, 45%), (*0.0375 mmol, 13%*), *(0.0386 mmol, 13%)*
Run 2: (34.4 mg, 0.130 mmol, 43%), *(0.046 mmol, 15%), (0.0392 mmol, 13%)*
Run 3: (32.1 mg, 0.121 mmol, 40%), *(0.0378 mmol, 13%), (0.036 mmol, 12%)*
Average 1,2-aminooygenation: 43% yield
Combined: 56% yield (61:*20:19*)

^{1}H NMR (400 MHz, $CDCl_3$) δ 7.77 (d, *J* = 15.9 Hz, 1H), 7.61 (ddd, *J* = 10.0, 5.3, 2.6 Hz, 2H), 7.20 – 7.08 (m, 2H), 6.92 (d, *J* = 15.9 Hz, 1H), 4.73 (t, *J* = 8.5 Hz, 1H), 4.53 (t, *J* = 8.8 Hz, 1H), 4.25 (t, *J* = 8.6 Hz, 1H), 3.90 (s, 3H).

$^{13}C\{^1H\}$ NMR (101 MHz, $CDCl_3$) δ 193.0, 166.1, 163.6, 158.4, 145.6, 131.0, 120.7, 116.5, 64.6, 64.2, 63.1.

^{19}F NMR (376 MHz, $CDCl_3$) δ -106.95.

HRMS (ESI) *m/z* calculated for $C_{13}H_{12}FNO_4$ $[M+H]^+$: 266.0824. Found: 266.0820.

mp. range: 50–51 °C.

(*E*)-4-cinnamoyl-3-methoxyoxazolidin-2-one (2b')

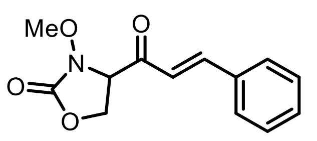

Prepared according to general procedure B: 1.5 mL catalyst stock solution (10.5 mg, 0.030 mmol, 0.10 equiv. $Cu(eh)_2$, 6.2 mg, 0.030 mol, 0.10 equiv. dmp), (2*E*,4*E*)-5-phenylpenta-2,4-dien-1-yl methoxycarbamate (**1b**) (70.0 mg, 0.300 mmol) were used. Crude material was purified by flash column chromatography (SiO_2) using a gradient of 30% EtOAc in hexanes to afford pure product as a white solid.

Run: (2b'), *(qNMR 2b), (qNMR cinnamaldehyde)*
Run 1: (25.2 mg, 0.102 mmol, 34%), (*0.0496 mmol, 17%*), *(0.0496 mmol, 17%)*
Run 2: (27.1 mg, 0.110 mmol, 37%), *(0.0404 mmol, 13%), (0.0541 mmol, 18%)*
Run 3: (24.7 mg, 0.100 mmol, 33%), *(0.049 mmol, 16%), (0.0542 mmol, 18%)*
Average 1,2-aminooygenation: 35% yield
Combined: 50% yield (51:*22:26*)

^1H NMR (400 MHz, $CDCl_3$) δ 7.81 (d, J = 16.0 Hz, 1H), 7.65 – 7.58 (m, 2H), 7.47 – 7.39 (m, 3H), 6.99 (d, J = 16.0 Hz, 1H), 4.76 (t, J = 8.6 Hz, 1H), 4.54 (t, J = 8.8 Hz, 1H), 4.26 (t, J = 8.7 Hz, 1H), 3.90 (s, 3H).

$^{13}C\{^1H\}$ NMR (101 MHz, $CDCl_3$) δ 193.1, 158.4, 147.0, 133.7, 131.9, 129.3, 129.0, 121.1, 64.6, 64.3, 63.1.

HRMS (ESI) *m/z* calculated for $C_{13}H_{13}NO_4$ $[M+H]^+$: 248.0920. Found: 248.0920.

mp. range: 39–40 °C.

(*E*)-3-methoxy-4-(3-(4-methoxyphenyl)acryloyl)oxazolidin-2-one (2c')

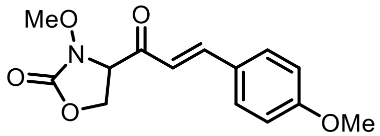

Prepared according to general procedure B: 1.5 mL catalyst stock solution (10.5 mg, 0.030 mmol, 0.10 equiv. $Cu(eh)_2$, 6.2 mg, 0.030 mol, 0.10 equiv. dmp), (2*E*,4*E*)-5-(4-methoxyphenyl)penta-2,4-dien-1-yl methoxycarbamate (**1c**) (79.0 mg, 0.300 mmol) were used. Crude material was purified by flash column chromatography (SiO_2) using a gradient of 30% EtOAc in hexanes to afford pure product as a white solid.

Run: (2c'), *(qNMR 2c), (qNMR cinnamaldehyde)*

Run 1: (25.8 mg, 0.0931 mmol, 31%), (*0.0095 mmol, 3%*), *(0.033 mmol, 11%)*
Run 2: (24.6 mg, 0.089 mmol, 30%), *(0.0098 mmol, 3%), (0.037 mmol, 12%)*
Run 3: (28.0 mg, 0.10 mmol, 34%), *(0.007 mmol, 2%), (0.037 mmol, 12%)*
Average 1,2-aminooygenation: 32% yield
Combined: 34% yield (67:*7:26*)

^{1}H NMR (400 MHz, $CDCl_3$) δ 7.77 (d, *J* = 15.6 Hz, 1H), 7.63 – 7.53 (m, 2H), 7.01 – 6.89 (m, 2H), 6.89 (d, 1H), 4.73 (dd, *J* = 8.6, 1.4 Hz, 1H), 4.52 (dd, *J* = 8.8, 1.5 Hz, 1H), 4.24 (dd, *J* = 8.7, 1.5 Hz, 1H), 3.89 (s, 3H), 3.87 (s, 3H).

$^{13}C\{^1H\}$ NMR (101 MHz, $CDCl_3$) δ 192.9, 162.8, 158.5, 146.7, 131.0, 126.5, 118.7, 114.8, 64.6, 64.2, 63.2, 55.6.

HRMS (ESI) *m/z* calculated for $C_{14}H_{15}NO_5$ $[M+H]^+$: 278.1020. Found: 278.1020.

mp. range: 47–48 °C.

(*E*)-3-methoxy-4-(3-(p-tolyl)acryloyl)oxazolidin-2-one (2d')

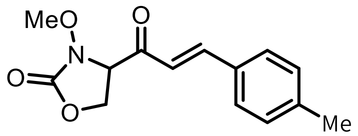

Prepared according to general procedure B: 1.5 mL catalyst stock solution (10.5 mg, 0.030 mmol, 0.10 equiv. $Cu(eh)_2$, 6.2 mg, 0.030 mol, 0.10 equiv. dmp)*, (2E,4E)-5-(*4-methylphenyl)penta-2,4-dien-1-yl methoxycarbamate (**1d**) (74.2 mg, 0.300 mmol) were used. Crude material was purified by flash column chromatography (SiO_2) using a gradient of 30% EtOAc in hexanes to afford pure product as a white solid.

Run: (2d'), *(qNMR 2d), (qNMR cinnamaldehyde)*
Run 1: (33.0 mg, 0.126 mmol, 42%), (*0.0320 mmol, 11%*), *(0.0162 mmol, 5%)*
Run 2: (33.3 mg, 0.127 mmol, 43%), *(0.0262 mmol, 9%), (0.0104 mmol, 4%)*
Run 3: (36.9 mg, 0.141 mmol, 47%), *(0.0325 mmol, 11%), (0.0166 mmol, 6%)*
Average 1,2-aminooygenation: 44% yield
Combined: 54% yield (75:*17:8*)

^{1}H NMR (400 MHz, $CDCl_3$) δ 7.78 (d, *J* = 15.9 Hz, 1H), 7.51 (d, *J* = 7.9 Hz, 2H), 7.24 (d, *J* = 7.6 Hz, 2H), 6.95 (d, *J* = 15.8 Hz, 1H), 4.74 (t, *J* = 8.7 Hz, 1H), 4.53 (td, *J* = 9.3, 1.8 Hz, 1H), 4.25 (td, *J* = 10.2, 2.8 Hz, 1H), 3.90 (d, *J* = 1.4 Hz, 3H), 2.40 (s, 3H).

$^{13}C\{^1H\}$ NMR (101 MHz, $CDCl_3$) δ 193.0, 158.5, 147.1, 142.7, 131.1, 130.1, 129.1, 120.1, 64.7, 64.3, 63.1, 21.8.

HRMS (ESI) *m/z* calculated for $C_{14}H_{15}NO_4$ $[M+H]^+$: 262.1074. Found: 262.1070.

mp. range: 37–38 °C.

(*E*)-3-methoxy-4-(3-(4-(trifluoromethyl)phenyl)acryloyl)oxazolidin-2-one (2e')

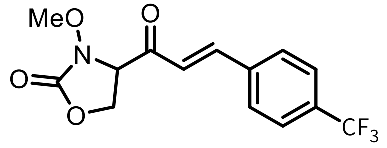

Prepared according to general procedure B: 1.5 mL catalyst stock solution (10.5 mg, 0.030 mmol, 0.10 equiv. $Cu(eh)_2$, 6.2 mg, 0.030 mol, 0.10 equiv. dmp), *(2E,4E)*-5-(4-(trifluoromethyl)phenyl)penta-2,4-dien-1-yl methoxycarbamate (**1e**) (90.4 mg, 0.300 mmol) were used. Crude material was purified by flash column chromatography (SiO_2) using a gradient of 30% EtOAc in hexanes to afford pure product as a white solid.

Run: (2e'), *(qNMR 2e), (qNMR cinnamaldehyde)*
Run 1: (14.8 mg, 0.047 mmol, 16%), (*0.039 mmol, 13%*), *(0.0131 mmol, 4%)*
Run 2: (15.1 mg, 0.048 mmol, 16%), *(0.041 mmol, 14%), (0.0133 mmol, 4%)*
Run 3: (14.2 mg, 0.458 mmol, 15%), *(0.042 mmol, 14%), (0.0116 mmol, 4%)*
Average 1,2-aminooygenation: 16% yield
Combined: 29% yield (47:*41:11*)

^{1}H NMR (400 MHz, CHLOROFORM-*D*) δ 8.06 (t, *J* = 10.1 Hz, 1H), 7.84 – 7.76 (m, 2H), 7.69 (s, 3H), 7.05 (dd, *J* = 15.9, 1.7 Hz, 1H), 4.77 – 4.68 (m, 1H), 4.63 – 4.49 (m, 1H), 3.92 – 3.82 (m, 3H).

^{13}C{^{1}H} NMR (101 MHz, CHLOROFORM-*D*) δ 193.08, 171.34, 158.28, 144.89, 129.46, 129.10, 129.05, 126.52, 126.28, 122.99, 64.71, 64.25, 62.91.

^{19}F NMR (376 MHz, $CDCl_3$) δ -62.91.

HRMS (ESI) *m/z* calculated for $C_{14}H_{12}F_3NO_4$ $[M+H]^+$: 316.0794. Found: 316.0790.

mp. range: 45–46 °C.

(*E*)-3-methoxy-4-(3-(*o*-tolyl)acryloyl)oxazolidin-2-one (2f')

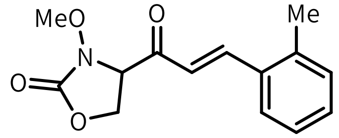

Prepared according to general procedure B: 1.5 mL catalyst stock solution (10.5 mg, 0.030 mmol, 0.10 equiv. $Cu(eh)_2$, 6.2 mg, 0.030 mol, 0.10 equiv. dmp), (2*E*,4*E*)-5-(*o*-tolyl)penta-2,4-dien-1-yl methoxycarbamate (**1f**) (74.2 mg, 0.300 mmol) were used. Crude material was purified by flash column chromatography (SiO_2) using a gradient of 30% EtOAc in hexanes to afford pure product as a clear oil.

Run: (2f'), *(qNMR 2f), (qNMR cinnamaldehyde)*
Run 1: (31.0 mg, 0.119 mmol, 40%), (*0.066 mmol, 22%*), *(0.0161 mmol, 5%)*
Run 2: (31.0 mg, 0.119 mmol, 40%), *(0.066 mmol, 22%), (0.0105 mmol, 4%)*
Run 3: (32.4 mg, 0.124 mmol, 41%), *(0.058 mmol, 20%), (0.0166 mmol, 6%)*
Average 1,2-aminooygenation: 41% yield
Combined: 62% yield (60:*32:7*)

^{1}H NMR (400 MHz, $CDCl_3$) δ 8.14 (d, *J* = 15.8 Hz, 1H), 7.64 (d, *J* = 7.9 Hz, 1H), 7.35 (t, *J* = 7.5 Hz, 1H), 7.24 (d, *J* = 8.0 Hz, 2H), 6.97 – 6.88 (m, 1H), 4.77 – 4.68 (m, 1H), 4.57 – 4.46 (m, 1H), 4.28 (td, *J* = 8.6, 1.4 Hz, 1H), 3.91 (d, *J* = 1.4 Hz, 3H), 2.48 (s, 3H).

$^{13}C\{^{1}H\}$ NMR (101 MHz, $CDCl_3$) δ 192.8, 144.2, 138.9, 132.3, 131.3, 131.1, 126.5, 126.4, 121.4, 64.5, 64.0, 62.7, 19.6.

HRMS (ESI) *m/z* calculated for $C_{14}H_{15}NO_4$ $[M+H]^+$: 262.1074. Found: 262.1068.

(*E*)-3-methoxy-4-(2-methyl-3-phenylacryloyl)oxazolidin-2-one (2g')

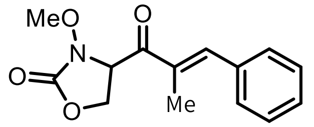

Prepared according to general procedure B: 2.0 mL catalyst stock solution (17.5 mg, 0.05 mmol, 0.10 equiv. $Cu(eh)_2$, 10.4 mg, 0.05 mol, 0.10 equiv. dmp), (2*E*,4*E*)-4-methyl-5-phenylpenta-2,4-dien-1-yl methoxycarbamate (**1g**) (111.6 mg, 0.500 mmol) were used. Crude material was purified by flash column chromatography (SiO_2) using a gradient of 30% EtOAc in hexanes to afford pure product as a white solid.

<u>Run:</u> (2g'), *(2g)*
<u>Run 1:</u> 0%, *(74.0 mg, 0.283 mmol, 57%)*
<u>Run 2:</u> 0%, *(74.0 mg, 0.283 mmol, 57%)*
<u>Run 3:</u> 0%, *(68.6 mg, 0.263 mmol, 53%)*
Average 1,2-aminooygenation: ND
Combined: 55% yield (<1:99)

(*E*)-3-methoxy-4-(2-bromo-3-phenylacryloyl)oxazolidin-2-one (2h')

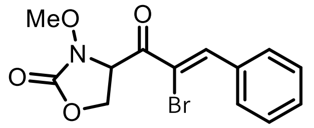

Prepared according to general procedure B: 2.0 mL catalyst stock solution (17.5 mg, 0.05 mmol, 0.10 equiv. $Cu(eh)_2$, 10.4 mg, 0.05 mol, 0.10 equiv. dmp), *(2E,4Z)*-4-bromo-5-phenylpenta-2,4-dien-1-yl methoxycarbamate (**1h**) (156.1 mg, 0.500 mmol) were used. Crude material was purified by flash column chromatography (SiO_2) using a gradient of 30% EtOAc in hexanes to afford pure product as a white solid.

<u>Run:</u> (2h'), *(2h)*
<u>Run 1:</u> 0%, *(94.6 mg, 0.290 mmol, 58%)*
<u>Run 2:</u> 0%, *(100.3 mg, 0.308 mmol, 62%)*
<u>Run 3:</u> 0%, *(99.4 mg, 0.305 mmol, 61%)*
Average 1,2-aminooygenation: 0%
Combined: 60% yield (<1:99)

(*E*)-5-methylhexa-2,4-dien-1-yl methoxycarbamate (1i)

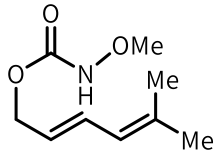

Prepared according to general procedure B: 2.0 mL catalyst stock solution (17.5 mg, 0.05 mmol, 0.10 equiv. $Cu(eh)_2$, 10.4 mg, 0.05 mol, 0.10 equiv. dmp), (*E*)-5-methylhexa-2,4-dien-1-yl methoxycarbamate (**1i**) (92.6 mg, 0.500 mmol) were used.

Run: (2i), *(2i')*
Run 1: (0 mg, 0.0 mmol, 0%), *(0 mg, 0.0 mmol, 0%)*
Run 2: (0 mg, 0.0 mmol, 0%), *(0 mg, 0.0 mmol, 0%)*
Run 3: (0 mg, 0.0 mmol, 0%), *(0 mg, 0.0 mmol, 0%)*
Average 1,2-aminooxygenation: 0% yield
Combined: 0% yield (n/a)

(E)-4-(but-2-enoyl)-3-methoxyoxazolidin-2-one (2j')

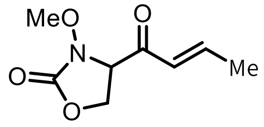

Prepared according to general procedure B: 2.0 mL catalyst stock solution (17.5 mg, 0.05 mmol, 0.10 equiv. $Cu(eh)_2$, 10.4 mg, 0.05 mol, 0.10 equiv. dmp), *(2E,4E)*-hexa-2,4-dien-1-yl methoxycarbamate (**1j**) (85.6 mg, 0.500 mmol) were used. Crude material was purified by flash column chromatography (SiO_2) using a gradient of 30% EtOAc in hexanes to afford pure product as a white solid.

Run: (2j'), *(2j)*
Run 1: 0%, *(34.7 mg, 0.187 mmol, 37%)*
Run 2: 0%, *(29.9 mg, 0.161 mmol, 32%)*
Run 3: 0%, *(32.2 mg, 0.174 mmol, 35%)*
Average 1,2-aminooygenation: 0%
Combined: 35% yield (<1:99)

(*E*)-4-(3-(furan-2-yl)acryloyl)-3-methoxyoxazolidin-2-one (2k')

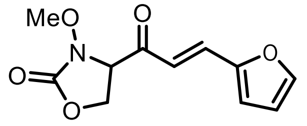

Prepared according to general procedure B: 2.0 mL catalyst stock solution (17.5 mg, 0.05 mmol, 0.10 equiv. $Cu(eh)_2$, 10.4 mg, 0.05 mol, 0.10 equiv. dmp), *(2E,4E)*-5-(furan-2-yl)penta-2,4-dien-1-yl methoxycarbamate (**1k**) (111.6 mg, 0.500 mmol) were used. Crude material was purified by flash column chromatography (SiO_2) using a gradient of 30% EtOAc in hexanes to afford pure product as a white solid.

Run: (2k'), *(qNMR 2k), (qNMR cinnamaldehyde)*
Run 1: (69.0 mg, 0.291 mmol, 58%), *0%, (0.045 mmol, 9%)*
Run 2: (69.1 mg, 0.291 mmol, 58%), *0%, ND*
Run 3: (62.9 mg, 0.265 mmol, 53%), *0%, ND*
Average 1,2-aminooygenation: 56% yield
Combined: 56% yield (86:0:14)

^{1}H NMR (400 MHz, $CDCl_3$) δ 7.60 – 7.51 (m, 2H), 6.90 – 6.79 (m, 2H), 6.54 (dd, *J* = 3.5, 1.8 Hz, 1H), 4.67 (t, *J* = 8.7 Hz, 1H), 4.51 (t, *J* = 8.8 Hz, 1H), 4.22 (t, *J* = 8.7 Hz, 1H), 3.89 (s, 3H).

$^{13}C\{^1H\}$ NMR (101 MHz, $CDCl_3$) δ 192.8, 158.5, 150.8, 146.3, 132.2, 118.8, 118.0, 113.3, 65.0, 64.3, 63.1.

HRMS (ESI) *m/z* calculated for $C_{11}H_{11}NO_5$ $[M+H]^+$: 238.0710. Found: 238.0710.

mp. range: 32–33 °C.

(*E*)-3-methoxy-4-(3-(pyridin-2-yl)acryloyl)oxazolidin-2-one (2l')

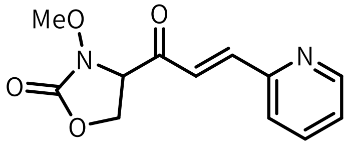

Prepared according to general procedure B: 1.5 mL catalyst stock solution (10.5 mg, 0.030 mmol, 0.10 equiv. $Cu(eh)_2$, 6.2 mg, 0.030 mol, 0.10 equiv. dmp), *(2E,4E)*-5-(pyridin-2-yl)penta-2,4-dien-1-yl methoxycarbamate (**1l**) (70.2 mg, 0.300 mmol) were used. Crude material was purified by flash column chromatography (SiO_2) using a gradient of 50% EtOAc in hexanes to afford pure product as a yellow oil.

Run: (2l'), *(qNMR 2l), (qNMR cinnamaldehyde)*
Run 1: (22.1 mg, 0.089 mmol, 30%), (*0.066 mmol, 22%*), *(0.0161 mmol, 5%)*
Run 2: (22.9 mg, 0.092 mmol, 31%), *(0.075 mmol, 25%), (0.0105 mmol, 4%)*
Run 3: (22.2 mg, 0.089 mmol, 30%), *(0.078 mmol, 26%), (0.0160 mmol, 5%)*
Average 1,2-aminooygenation: 30% yield
Combined: 62% yield (50:*40:8*)

^1H NMR (500 MHz, $CDCl_3$) δ 8.67 (d, *J* = 4.8 Hz, 1H), 7.79 – 7.72 (m, 2H), 7.53 – 7.45 (m, 2H), 7.33 (dd, *J* = 7.7, 4.7 Hz, 1H), 4.75 (q, *J* = 8.6 Hz, 1H), 4.52 (dt, *J* = 20.2, 10.4 Hz, 1H), 4.24 – 4.12 (m, 1H), 3.90 (s, 3H).

$^{13}C\{^1H\}$ NMR (101 MHz, $CDCl_3$) δ 193.7, 152.4, 150.9, 145.2, 137.6, 126.4, 125.8, 124.8, 65.5, 64.7, 63.1.

HRMS (ESI) *m/z* calculated for $C_{12}H_{12}N_2O_4$ $[M+H]^+$: 249.0880. Found: 249.0861.

(*E*)-3-methoxy-4-(3-(3-(trifluoromethyl)phenyl)acryloyl)oxazolidin-2-one (2m')

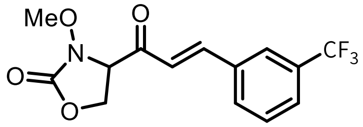

Prepared according to general procedure B: 1.5 mL catalyst stock solution (10.5 mg, 0.030 mmol, 0.10 equiv. $Cu(eh)_2$, 6.2 mg, 0.030 mol, 0.10 equiv. dmp), *(2E,4E)*-5-(3-(trifluoromethyl)phenyl)penta-2,4-dien-1-yl methoxycarbamate (**1m**) (90.4 mg, 0.300 mmol) were used. Crude material was purified by flash column chromatography (SiO_2) using a gradient of 30% EtOAc in hexanes to afford pure product as a white solid.

Run: (2m'), *(qNMR 2m), (qNMR cinnamaldehyde)*
Run 1: (20.7 mg, 0.075 mmol, 25%), (*0.101 mmol, 34%*), *(0.0131 mmol, 4%)*
Run 2: (24.8 mg, 0.089 mmol, 30%), *(0.100 mmol, 33%), (0.0134 mmol, 4%)*
Run 3: (23.8 mg, 0.858 mmol, 29%%), *(0.091 mmol, 30%), (0.0116 mmol, 4%)*

Average 1,2-aminooygenation: 28% yield
Combined: 60% yield (44:*50:6*)

^{1}H NMR (400 MHz, $CDCl_3$) δ 7.91 – 7.76k (m, 3H), 7.69 (d, *J* = 6.7 Hz, 1H), 7.58 (t, *J* = 7.8 Hz, 1H), 7.05 (d, *J* = 16.0 Hz, 1H), 4.76 (t, *J* = 8.6 Hz, 1H), 4.55 (t, *J* = 8.8 Hz, 1H), 4.27 (t, *J* = 8.6 Hz, 1H), 3.91 (s, 3H).

$^{13}C\{^{1}H\}$ NMR (101 MHz, $CDCl_3$) δ 192.9, 158.3, 145.0, 134.6, 131.9, 130.0, 128.1, 125.5, 125.5, 122.5, 64.7, 64.3, 62.9.

^{19}F NMR (376 MHz, $CDCl_3$) δ -62.83.

HRMS (ESI) *m/z* calculated for $C_{14}H_{12}F_3NO_4$ $[M+H]^+$: 316.0792. Found: 316.0790.

mp. range: 41–42 °C.

(*E*)-4-(3-(benzo[d][1,3]dioxol-5-yl)acryloyl)-3-methoxyoxazolidin-2-one (2n')

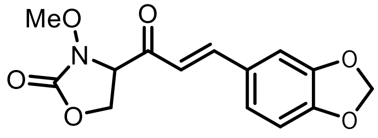

Prepared according to general procedure B: 1.5 mL catalyst stock solution (10.5 mg, 0.030 mmol, 0.10 equiv. $Cu(eh)_2$, 6.2 mg, 0.030 mol, 0.10 equiv. dmp), (2E,4E)-5-(benzo[d][1,3]dioxol-5-yl)penta-2,4-dien-1-yl methoxycarbamate (**1n**) (90.4 mg, 0.300 mmol) were used. Crude material was purified by flash column chromatography (SiO_2) using a gradient of 30% EtOAc in hexanes to afford pure product as a white solid.

<u>Run:</u> (2n'), *(qNMR 2e), (qNMR cinnamaldehyde)*
<u>Run 1:</u> (21.8 mg, 0.075 mmol, 25%), (*0.087 mmol, 29%*), *(0.0131 mmol, 4%)*
<u>Run 2:</u> (22.1 mg, 0.078 mmol, 26%), *(0.085 mmol, 28%), (0.0134 mmol, 4%)*
<u>Run 3:</u> (23.8 mg, 0.075 mmol, 25%), *(0.084 mmol, 28%), (0.0116 mmol, 4%)*
Average 1,2-aminooygenation: 25% yield
Combined: 60% yield (44:*49:7*)

^{1}H NMR (400 MHz, $CDCl_3$) δ 7.70 (d, *J* = 15.8 Hz, 1H), 7.10 (d, *J* = 7.0 Hz, 2H), 6.89 – 6.77 (m, 2H), 6.03 (q, *J* = 1.1 Hz, 2H), 4.70 (t, *J* = 8.7 Hz, 1H), 4.55 – 4.43 (m, 1H), 4.27 – 4.18 (m, 1H), 3.88 (s, *J* = 1.0 Hz, 3H).

$^{13}C\{^{1}H\}$ NMR (101 MHz, $CDCl_3$) δ 192.8, 158.5, 151.1, 148.7, 146.7, 128.2, 126.5, 118.9, 108.9, 106.8, 102.0, 64.7, 64.2, 63.1.

HRMS (ESI) *m/z* calculated for $C_{14}H_{13}NO_6$ $[M+H]^+$: 292.0821. Found: 292.0820

mp. range: 57–58 °C.

Scaled Reaction

General Procedure A: Catalyst stock solution: CuCl (5.0 mg, 0.050 mmol, 1.0 equiv.) and dmp (10.4 mg, 0.050 mmol, 1.0 equiv.) were weighed into a 25 mL volumetric flask in a glovebox. The vial was sealed was removed from the glovebox and the solids were taken up in MeCN (25 mL, 2.0 mM) and stirred at rt for 15 min as the vial is exposed to air.

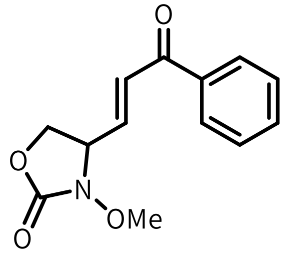

Reaction: 7.5 mL of the catalyst stock solution (containing CuCl (1.48 mg, 0.015 mmol, 0.010 equiv.) and dmp (3.12 mg, 0.015 mmol, 0.010 equiv.) was transferred to an 100 mL beaker equipped with a stir bar. *(2E,4E)*-5-phenylpenta-2,4-dien-1-yl methoxycarbamate (**1b**) (349.9 mg, 1.5 mmol, 1.0 equiv.) was added in one portion and the reaction mixture was stirred at 500 rpm for 1h in the beaker. The reaction was quenched by filtering through a ~2 cm pad of SiO_2 in a Pasteur pipette and eluting with ~10 mL EtOAc. The filtrate was concentrated in vacuo and the crude residue was purified by flash chromatography on SiO_2 using 30% EtOAc in hexanes.

Run: (mg **2b**, mmol **2b**, yield **2b**), *(qNMR* **2b'***)*
Run 1: (164.0 mg, 0.66 mmol, 44%), (0.18 mmol, 12%)
Combined: 56% yield (79:21)

General Procedure B: Catalyst stock solution: $Cu(eh)_2$ (175 mg, 0.500 mmol, 1.0 equiv.) and dmp (104 mg, 0.500 mmol, 1.0 equiv.) were weighed into a 25 mL volumetric flask in a glovebox. The vial was sealed was removed from the glovebox and the solids were taken up in MeCN (25 mL, 2.0 mM) and stirred at rt for 15 min as the vial is exposed to air.

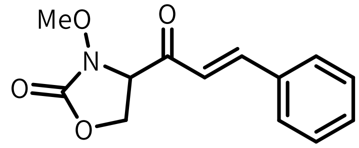

Reaction: 7.5 mL of the catalyst stock solution (containing $Cu(eh)_2$ (52.5 mg, 0.15 mmol, 0.10 equiv.) and dmp (31.2 mg, 0.15 mmol, 0.10 equiv.) was transferred to an 100 mL beaker equipped with a stir bar. *(2E,4E)*-5-phenylpenta-2,4-dien-1-yl methoxycarbamate (**1b**) (349.9 mg, 1.5 mmol, 1.0 equiv.) was added in one portion and the reaction mixture was stirred at 500 rpm for 2h in the beaker. The reaction was quenched by filtering through a ~2 cm pad of SiO_2 in a Pasteur pipette and eluting with ~10 mL EtOAc. The filtrate was concentrated in vacuo and the crude residue was purified by flash chromatography on SiO_2 using 30% EtOAc in hexanes.

Run: (mg **2b'**, mmol **2b'**, yield **2b'**), *(qNMR* **2b***)*
Run 1: (131.0 mg, 0.53 mmol, 35%), (0.24 mmol, 16%)
Combined: 51% yield 68:32)

Methodology Limitations

Substrates bearing coordinating amine functionality, pyridine rings, tertiary alkyl substrates, and *tert*-butyl protecting group did not produce any desired aminooxygenation products under either set of standard reaction conditions, affording quantitative recovery of starting material.

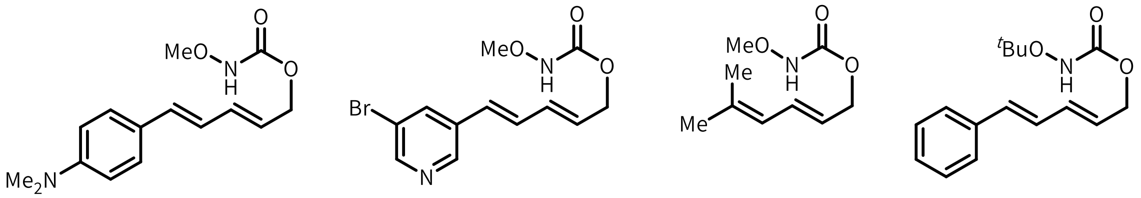

References Cited

[1] Einaru, S.; Shitamichi, K.; Nagano, T.; Matsumoto, A.; Asano, K.; Matsubara, S. trans-Cyclooctenes as Halolactonization Catalysts. *Angew. Chem. Int. Ed.* **2018**, *57*, 13863-13867.

[2] Shen, K.; Wang, Q. Copper-Catalyzed Alkene Aminoazidation as a Rapid Entry to 1,2-Diamines and Installation of an Azide Reporter onto Azahetereocycles. *J. Am. Chem. Soc.* **2017**, *139*, 13110-13116.

[3] McNichol, C. P.; DeCicco, E. M.; Canfield, A. M.; Carstairs, D. P.; Paradine, S. M. Copper-Catalyzed Aerobic Aminooxygenation of Cinnamyl N-Alkoxycarbamates via Substrate-Promoted Catalyst Activation. *ACS Catal.* **2023**, *13* (10), 6568–6573.

[5] *CrysAlisPro*, version 171.43.136a; Rigaku Corporation: Oxford, UK, 2024.

[6] Sheldrick, G. M. *SHELXT*, version 2018/2; *Acta. Crystallogr.* **2015**, *A71*, 3-8.

[7] Sheldrick, G. M. *SHELXL*, version 2019/3; *Acta. Crystallogr.* **2015**, *C71*, 3-8.

[8] Dolomanov, O. V.; Bourhis, L. J.; Gildea, R. J.; Howard, J. A. K.; Puschmann, H. *Olex2*, version 1.5; *J. Appl. Cryst.* **2009**, *42*, 339-341.

1H NMR, 13C NMR, and 19F NMR Spectra for New Compounds

(2E,4E)-5-(4-fluorophenyl)penta-2,4-dien-1-yl methoxycarbamate (1a)

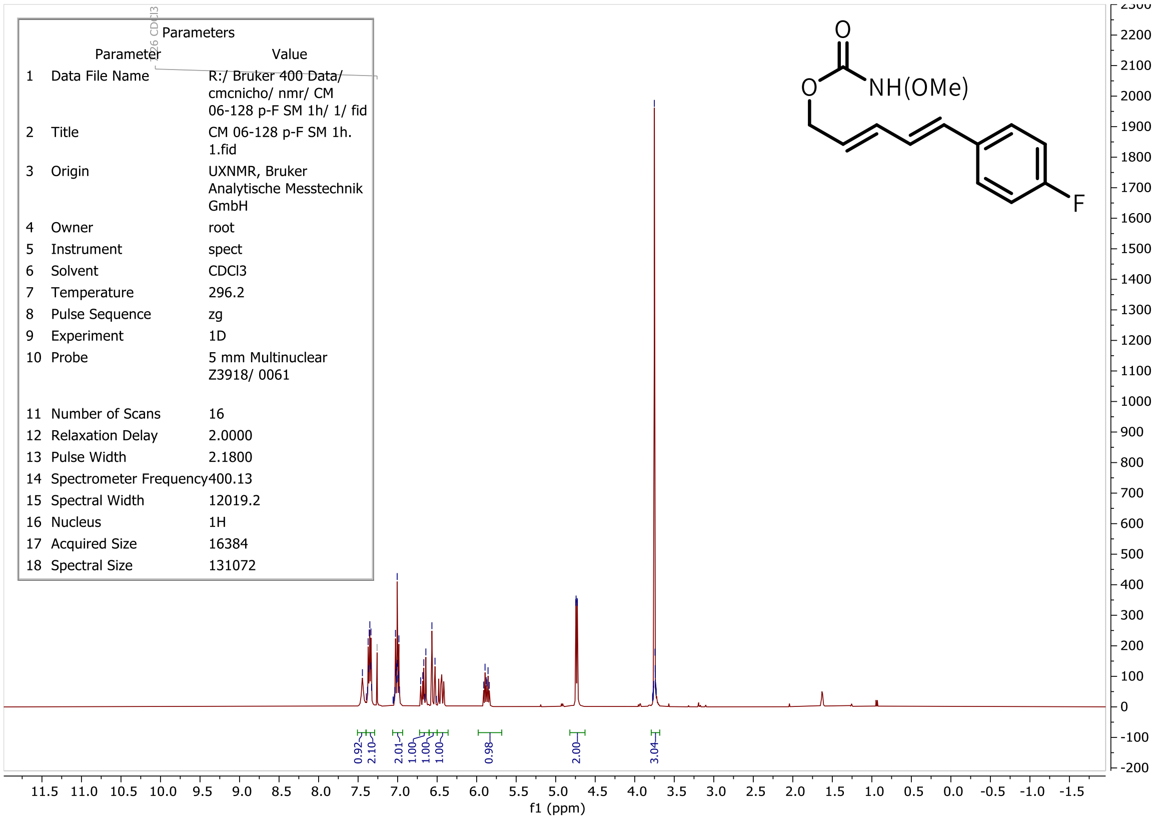

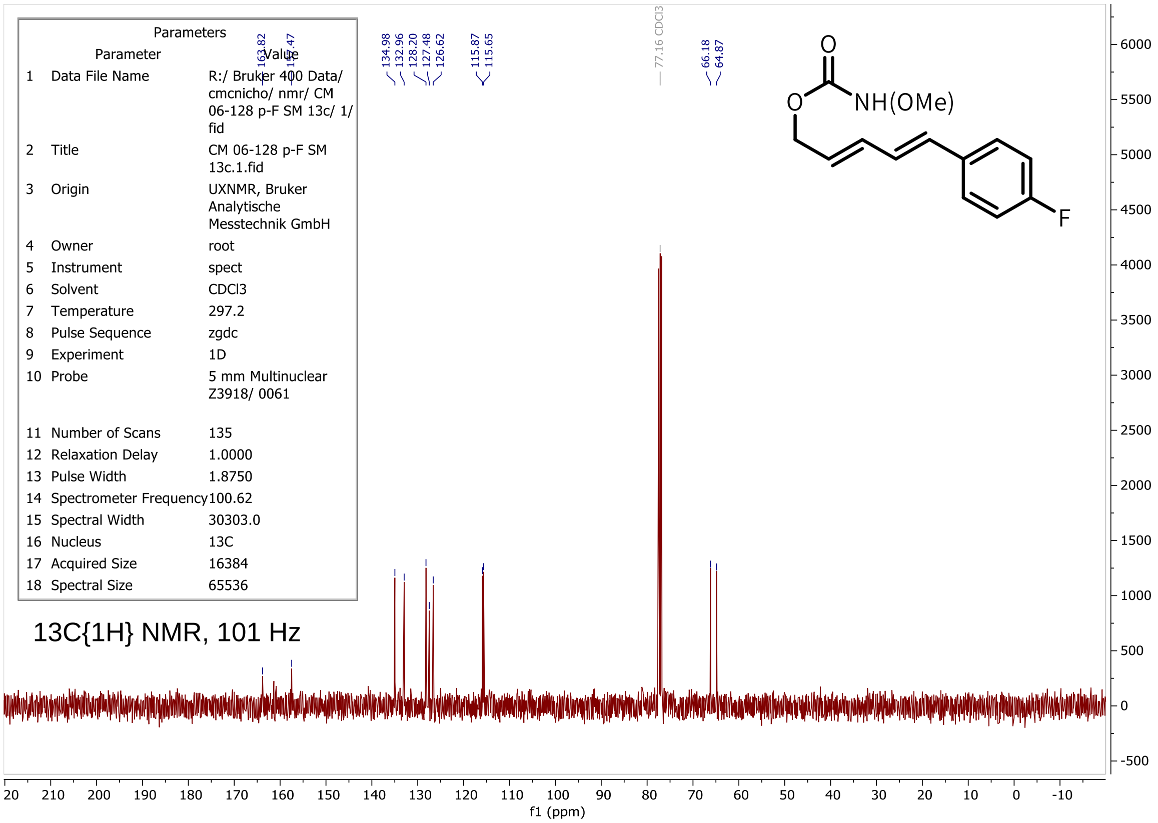

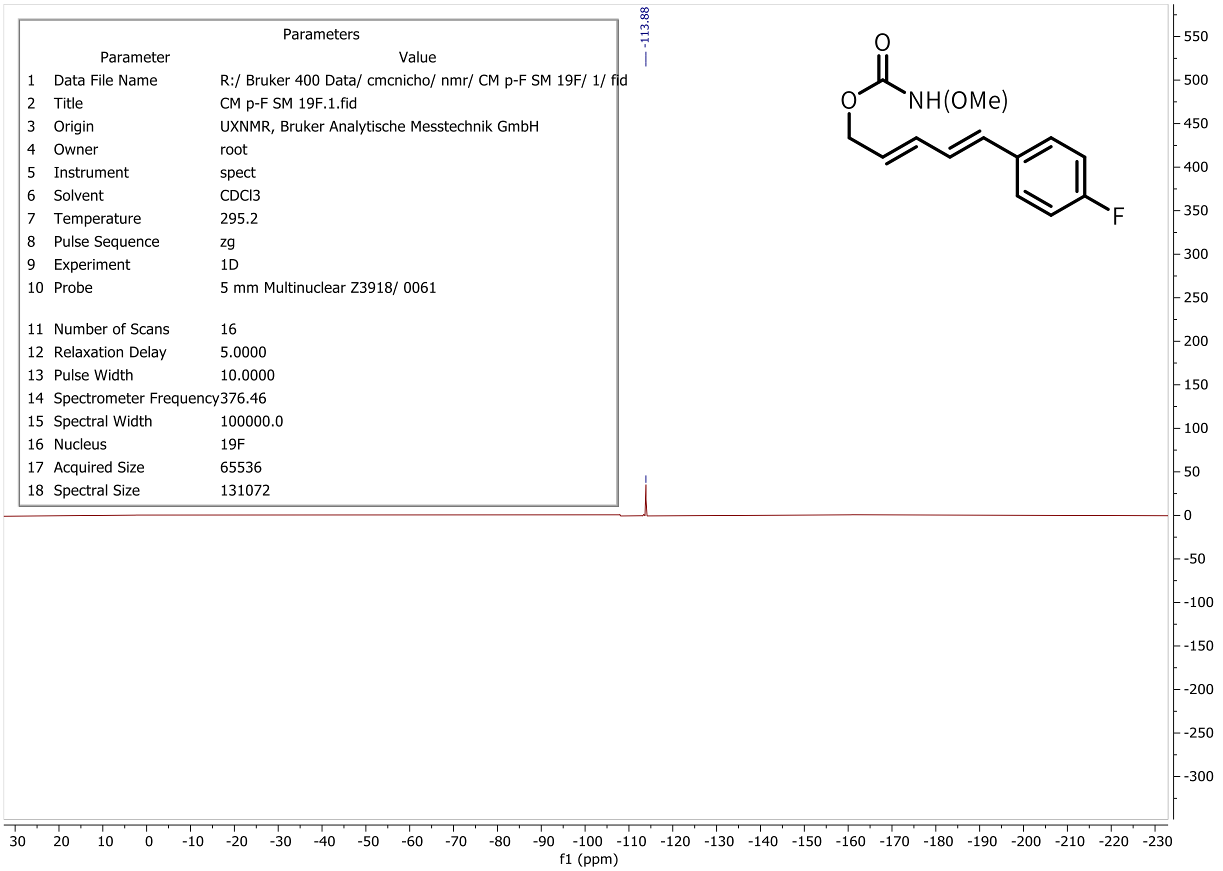
-113.88
Parameters
Parameter Value
1 Data File Name R:/ Bruker 400 Data/ cmcnicho/ nmr/ CM p-F SM 19F/ 1/ fid
2 Title CM p-F SM 19F.1.fid
3 Origin UXNMR, Bruker Analytische Messtechnik GmbH
4 Owner root
5 Instrument spect
6 Solvent CDCl3
7 Temperature 295.2
8 Pulse Sequence zg
9 Experiment 1D
10 Probe 5 mm Multinuclear Z3918/ 0061
11 Number of Scans 16
12 Relaxation Delay 5.0000
13 Pulse Width 10.0000
14 Spectrometer Frequency 376.46
15 Spectral Width 100000.0
16 Nucleus 19F
17 Acquired Size 65536
18 Spectral Size 131072
O
O
NH(OMe)
F
f1 (ppm)

(2E,4E)-5-phenylpenta-2,4-dien-1-yl methoxycarbamate (1b)

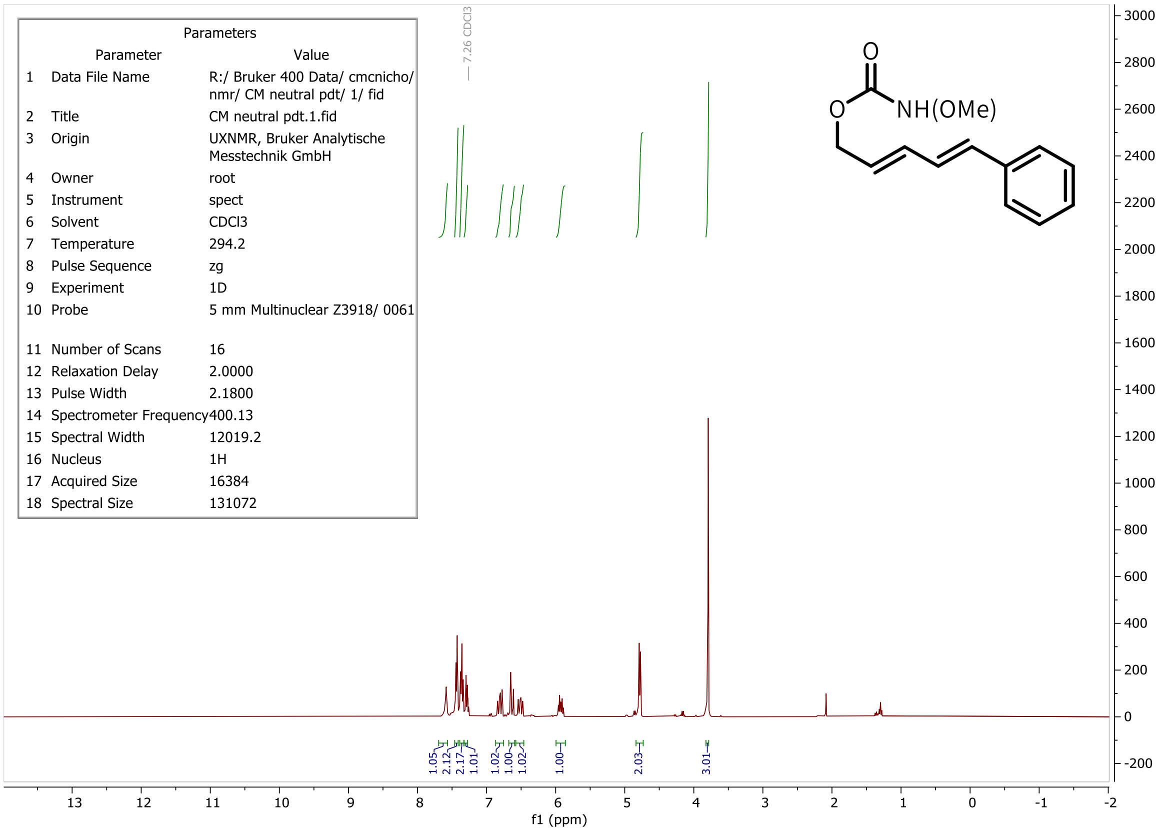

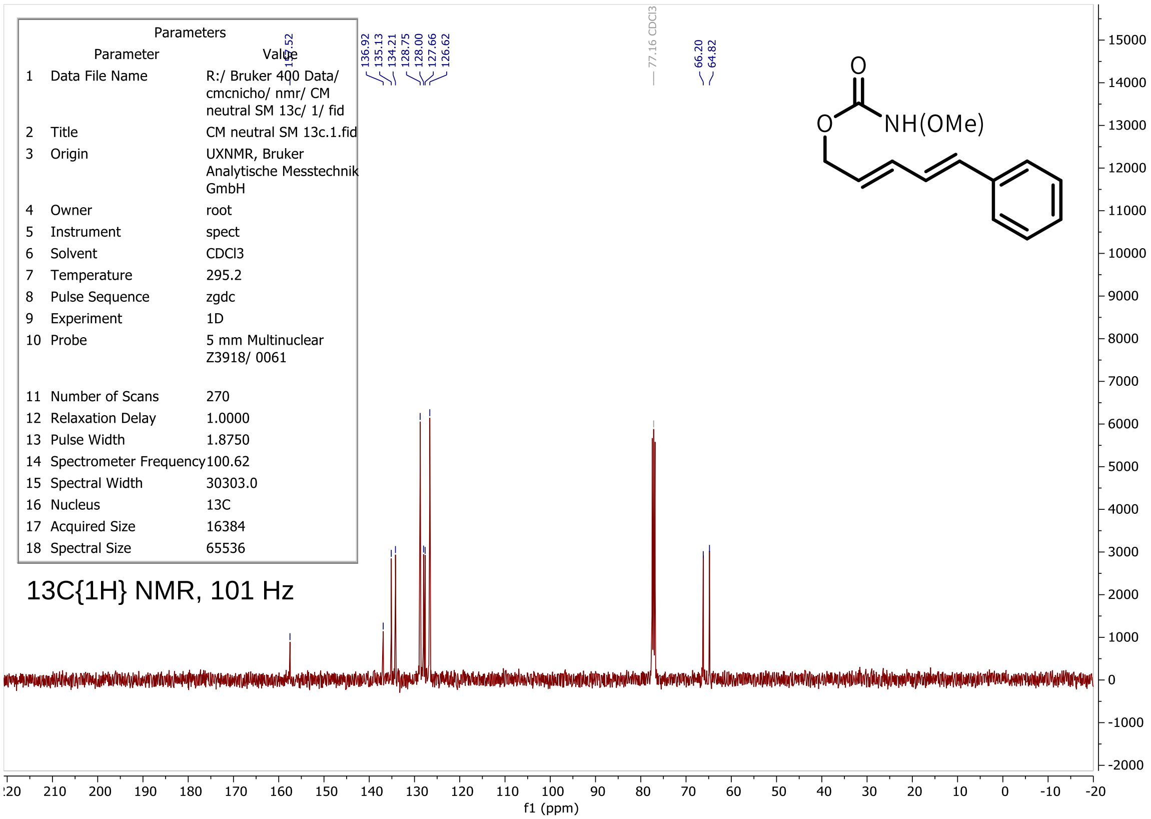

(2E,4E)-5-(4-methoxyphenyl)penta-2,4-dien-1-yl methoxycarbamate (1c)

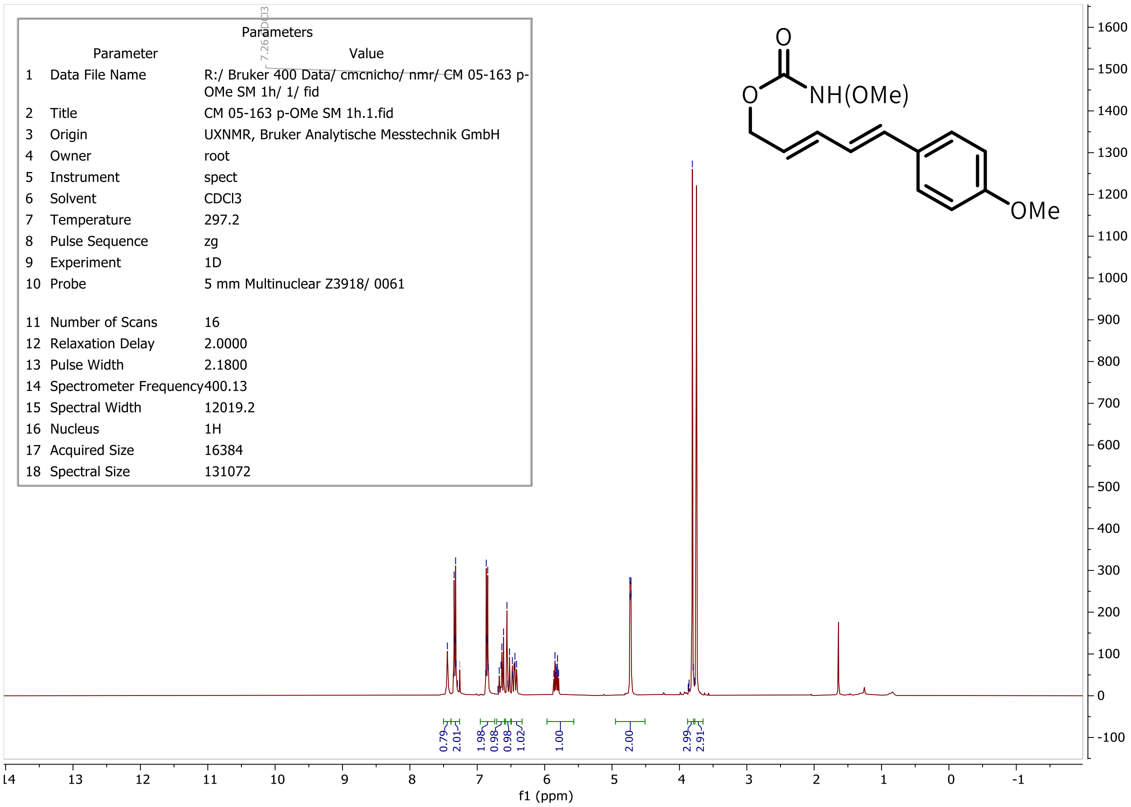

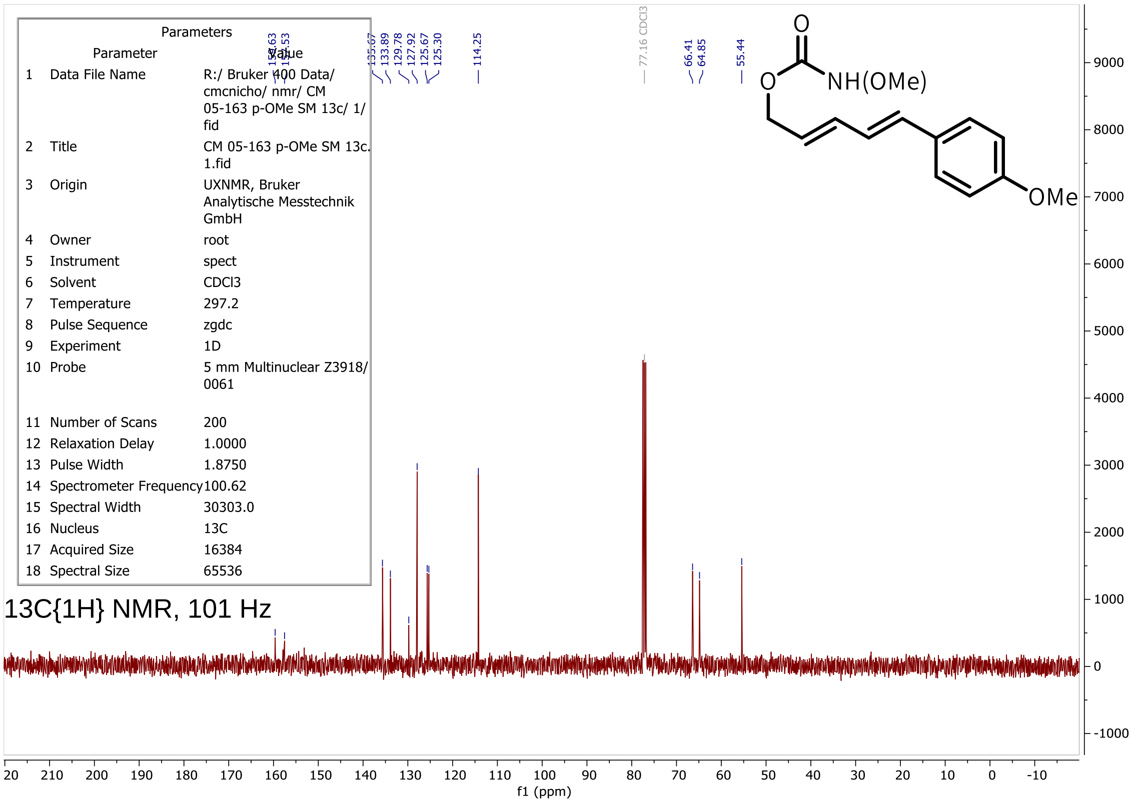

(2E,4E)-5-(4-methylphenyl)penta-2,4-dien-1-yl methoxycarbamate (1d)

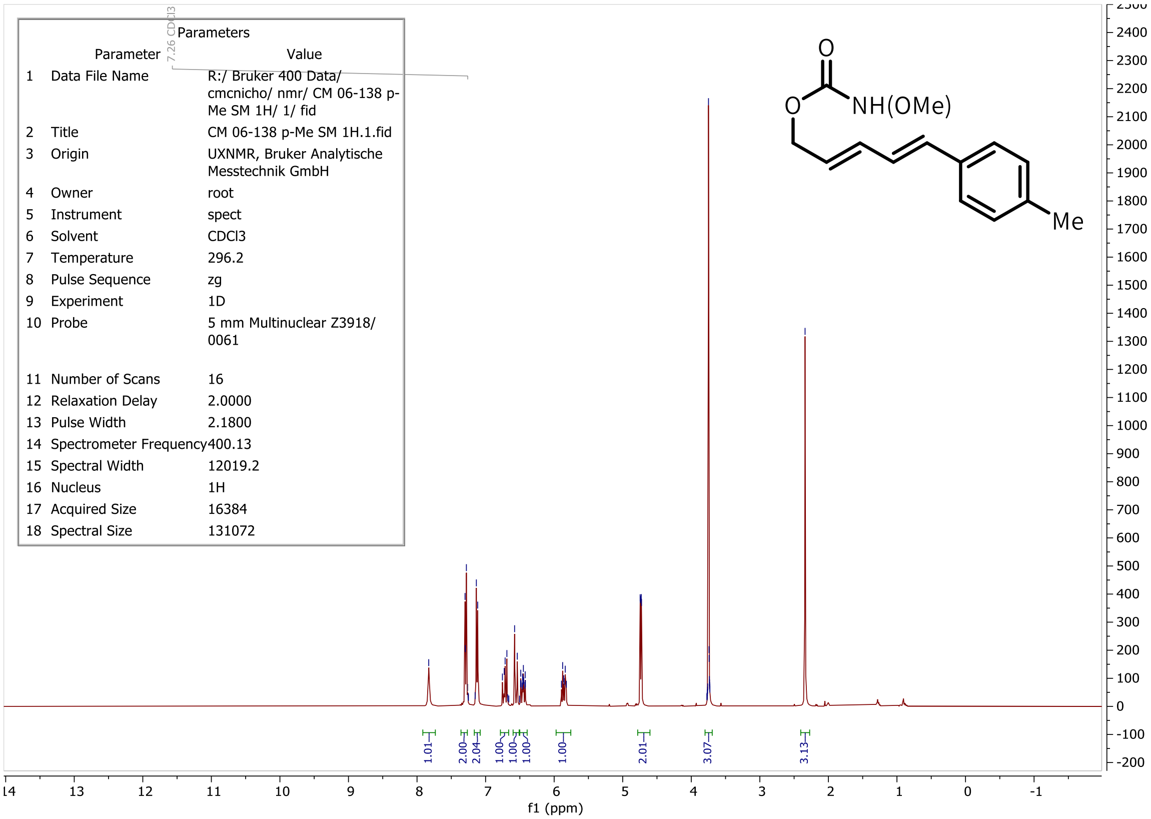

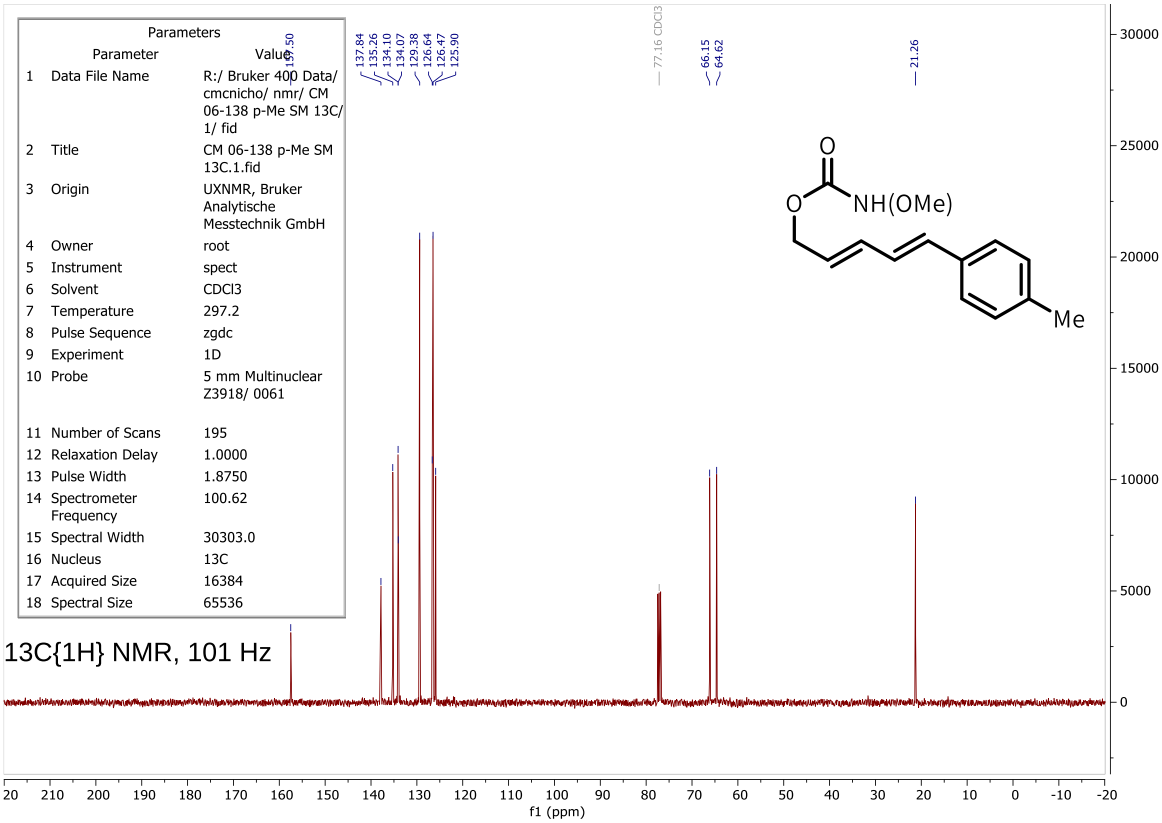

(2E,4E)-5-(4-(trifluoromethyl)phenyl)penta-2,4-dien-1-yl methoxycarbamate (1e)

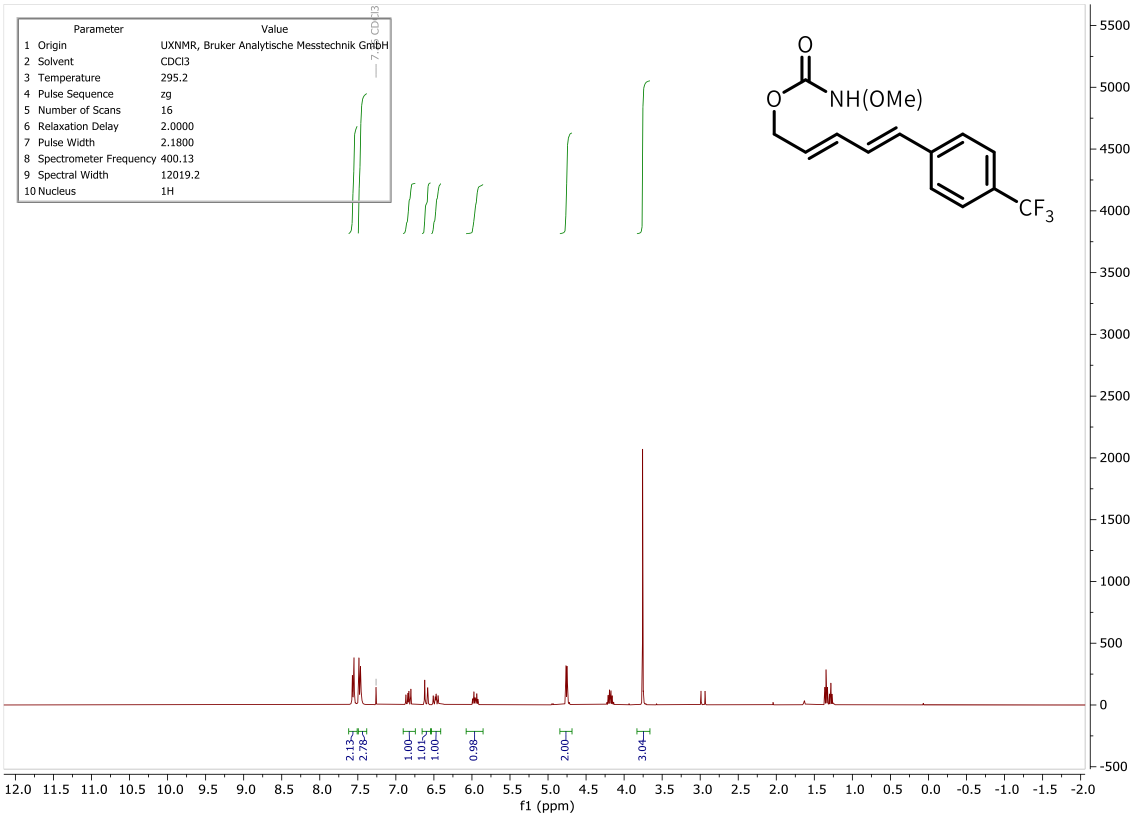

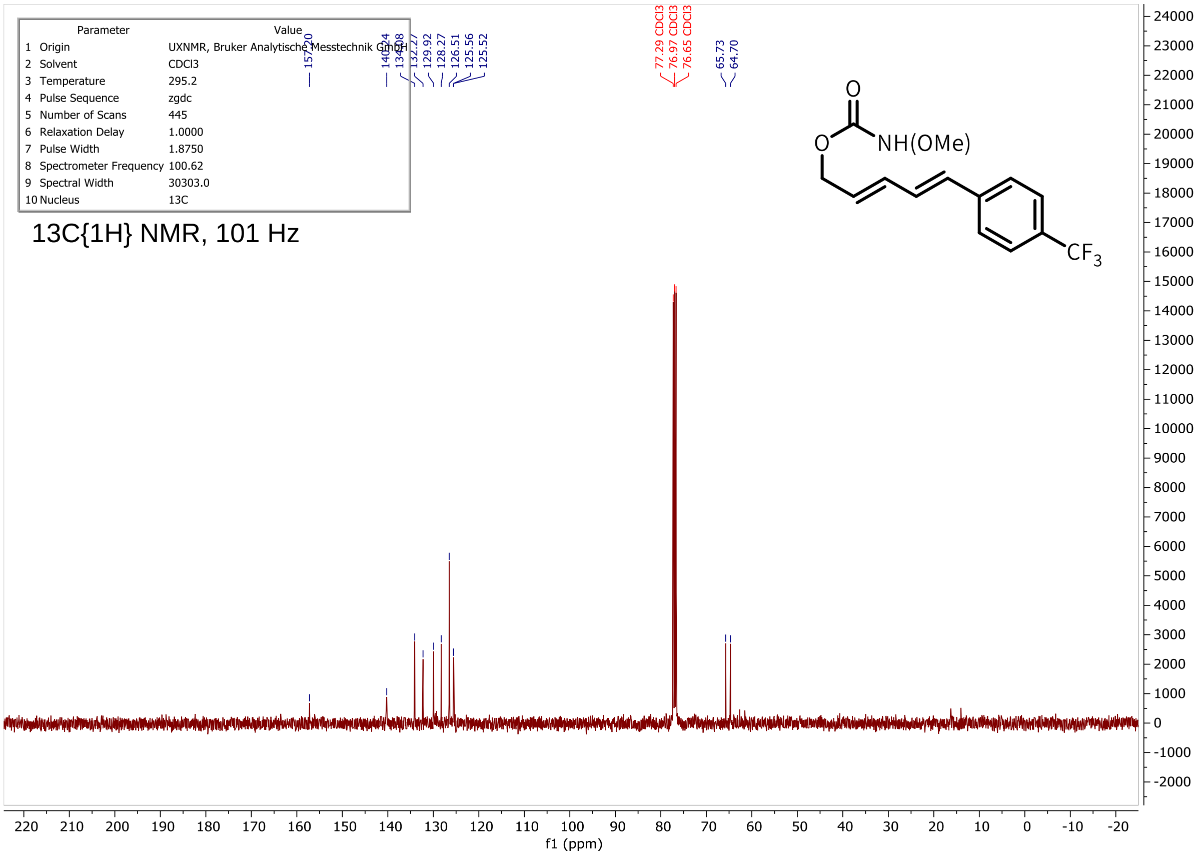

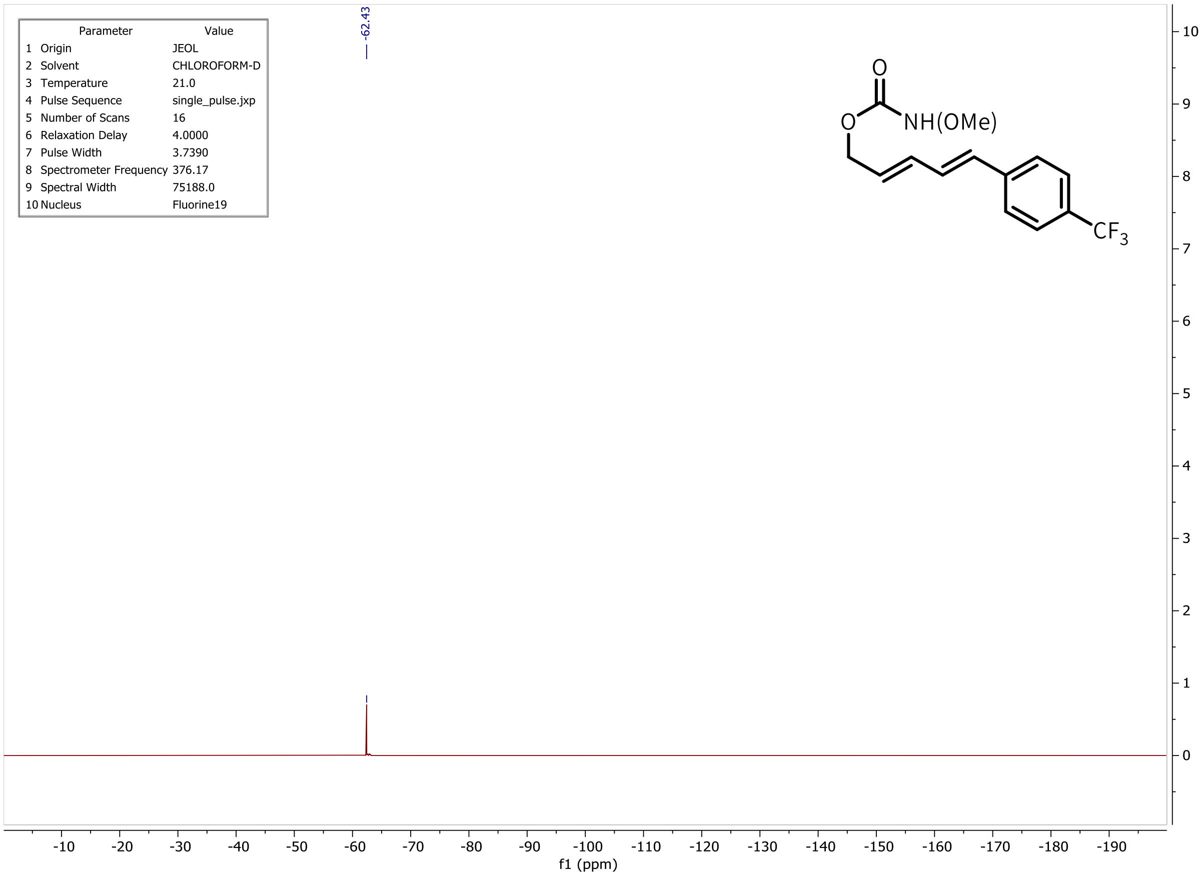

Parameter Value
1 Origin JEOL
2 Solvent CHLOROFORM-D
3 Temperature 21.0
4 Pulse Sequence single_pulse.jxp
5 Number of Scans 16
6 Relaxation Delay 4.0000
7 Pulse Width 3.7390
8 Spectrometer Frequency 376.17
9 Spectral Width 75188.0
10 Nucleus Fluorine19
-62.43
O
O
NH(OMe)
CF3
f1 (ppm)

(2E,4E)-5-(o-tolyl)penta-2,4-dien-1-yl methoxycarbamate (1f)

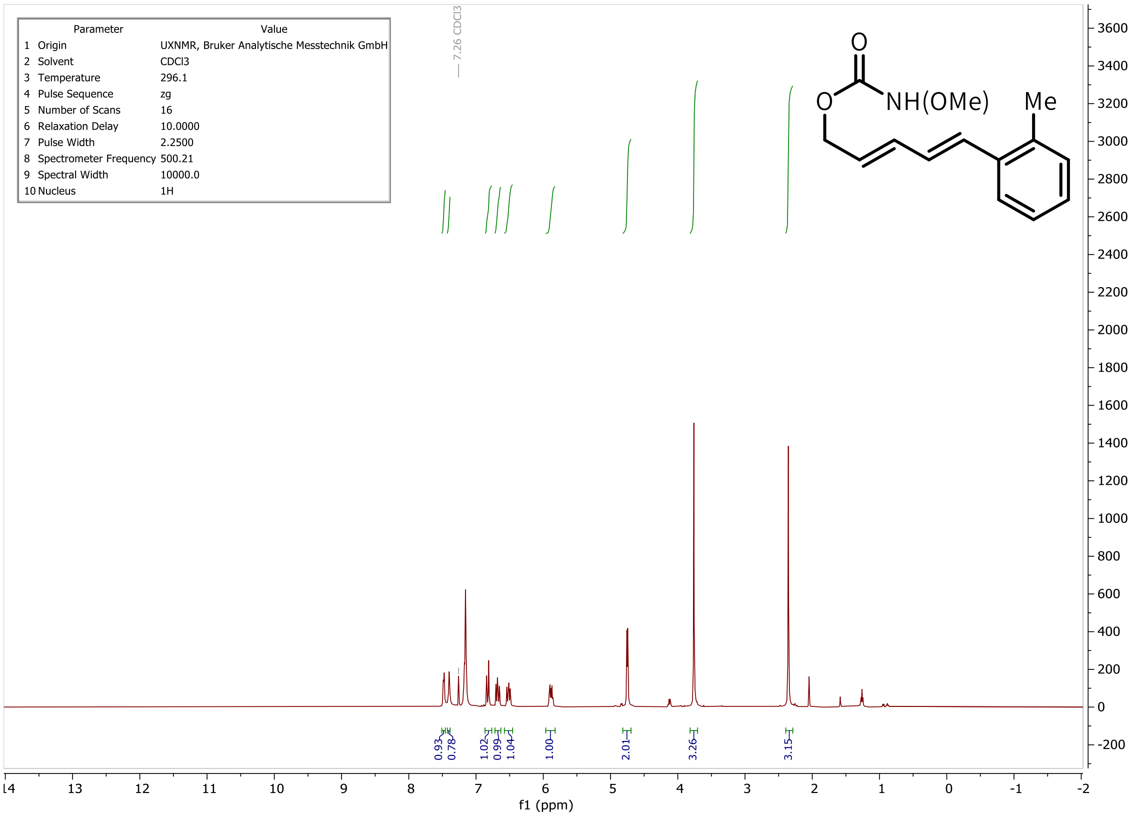

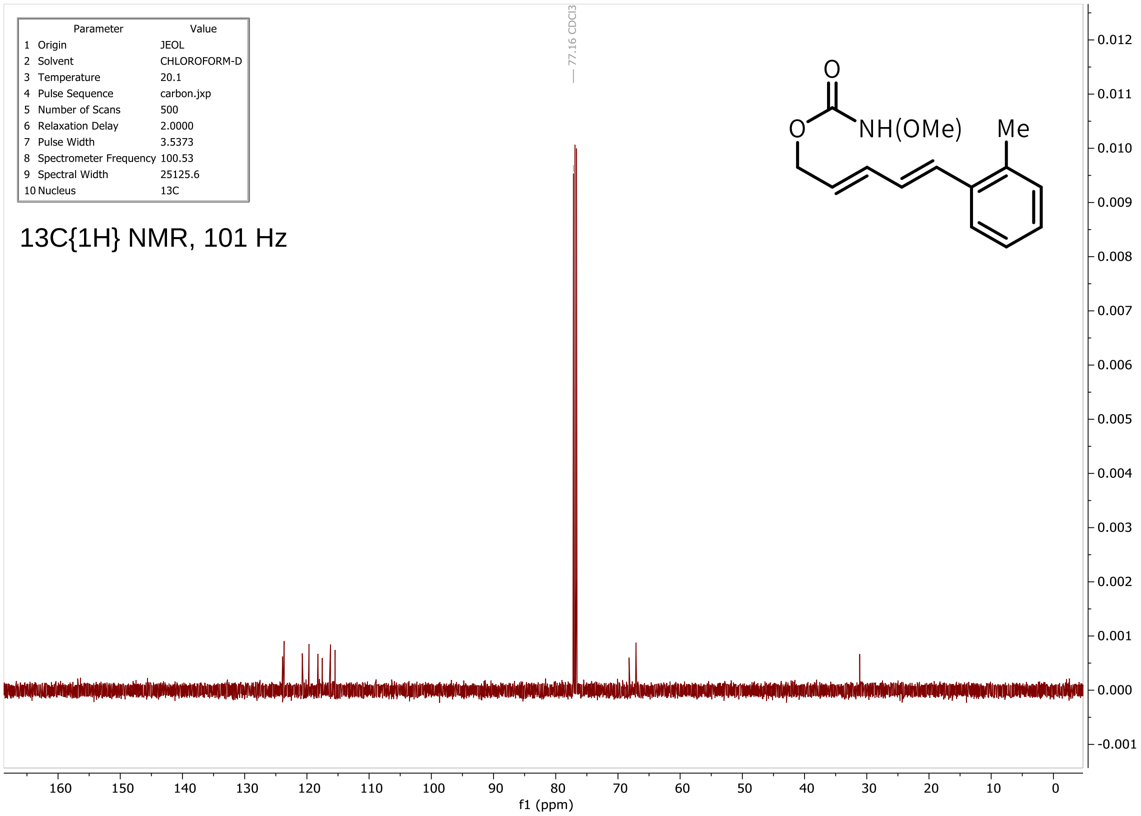

(2E,4E)-4-methyl-5-phenylpenta-2,4-dien-1-yl methoxycarbamate (1g)

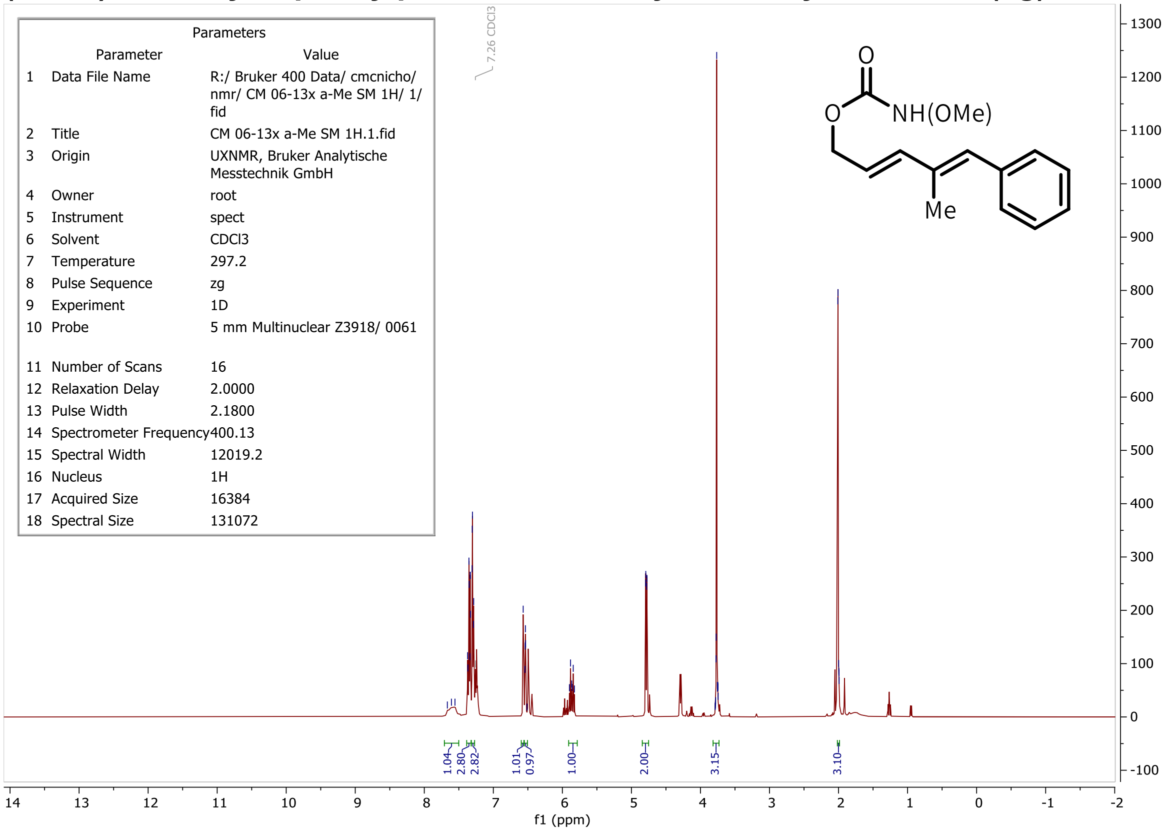

Parameters

	Parameter	Value
1	Data File Name	R:/ Bruker 400 Data/ cmcnicho/ nmr/ CM 06-13x a-Me SM 1H/ 1/ fid
2	Title	CM 06-13x a-Me SM 1H.1.fid
3	Origin	UXNMR, Bruker Analytische Messtechnik GmbH
4	Owner	root
5	Instrument	spect
6	Solvent	CDCl3
7	Temperature	297.2
8	Pulse Sequence	zg
9	Experiment	1D
10	Probe	5 mm Multinuclear Z3918/ 0061
11	Number of Scans	16
12	Relaxation Delay	2.0000
13	Pulse Width	2.1800
14	Spectrometer Frequency	400.13
15	Spectral Width	12019.2
16	Nucleus	1H
17	Acquired Size	16384
18	Spectral Size	131072

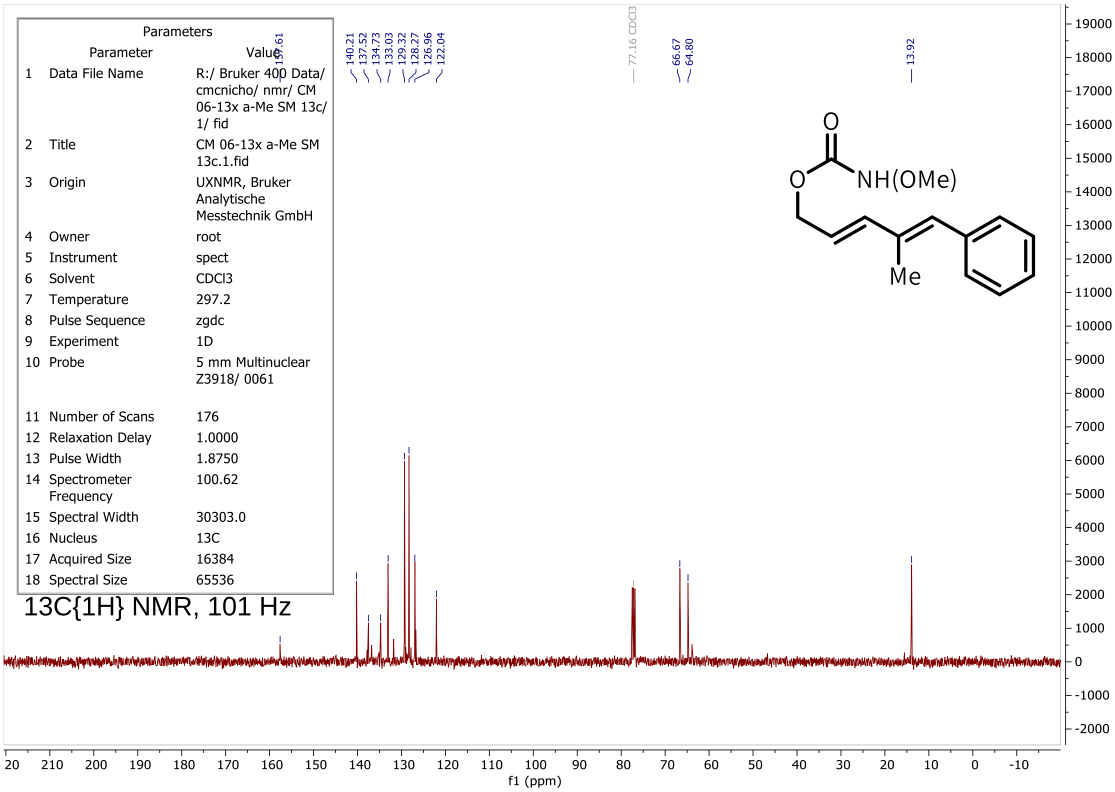

Parameters

	Parameter	Value
1	Data File Name	R:/ Bruker 400 Data/ cmcnicho/ nmr/ CM 06-13x a-Me SM 13c/ 1/ fid
2	Title	CM 06-13x a-Me SM 13c.1.fid
3	Origin	UXNMR, Bruker Analytische Messtechnik GmbH
4	Owner	root
5	Instrument	spect
6	Solvent	CDCl3
7	Temperature	297.2
8	Pulse Sequence	zgdc
9	Experiment	1D
10	Probe	5 mm Multinuclear Z3918/ 0061
11	Number of Scans	176
12	Relaxation Delay	1.0000
13	Pulse Width	1.8750
14	Spectrometer Frequency	100.62
15	Spectral Width	30303.0
16	Nucleus	13C
17	Acquired Size	16384
18	Spectral Size	65536

(2E,4E)-4-bromo-5-phenylpenta-2,4-dien-1-yl methoxycarbamate (1h)

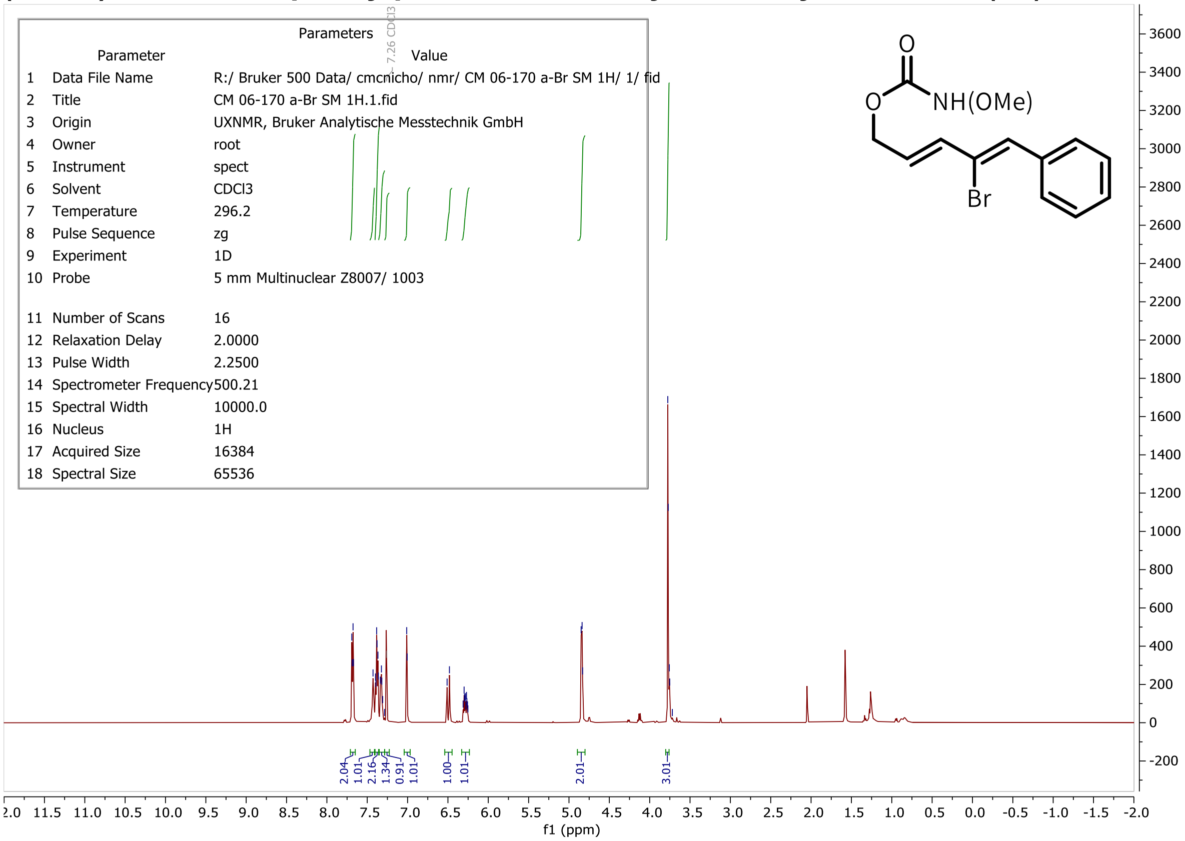

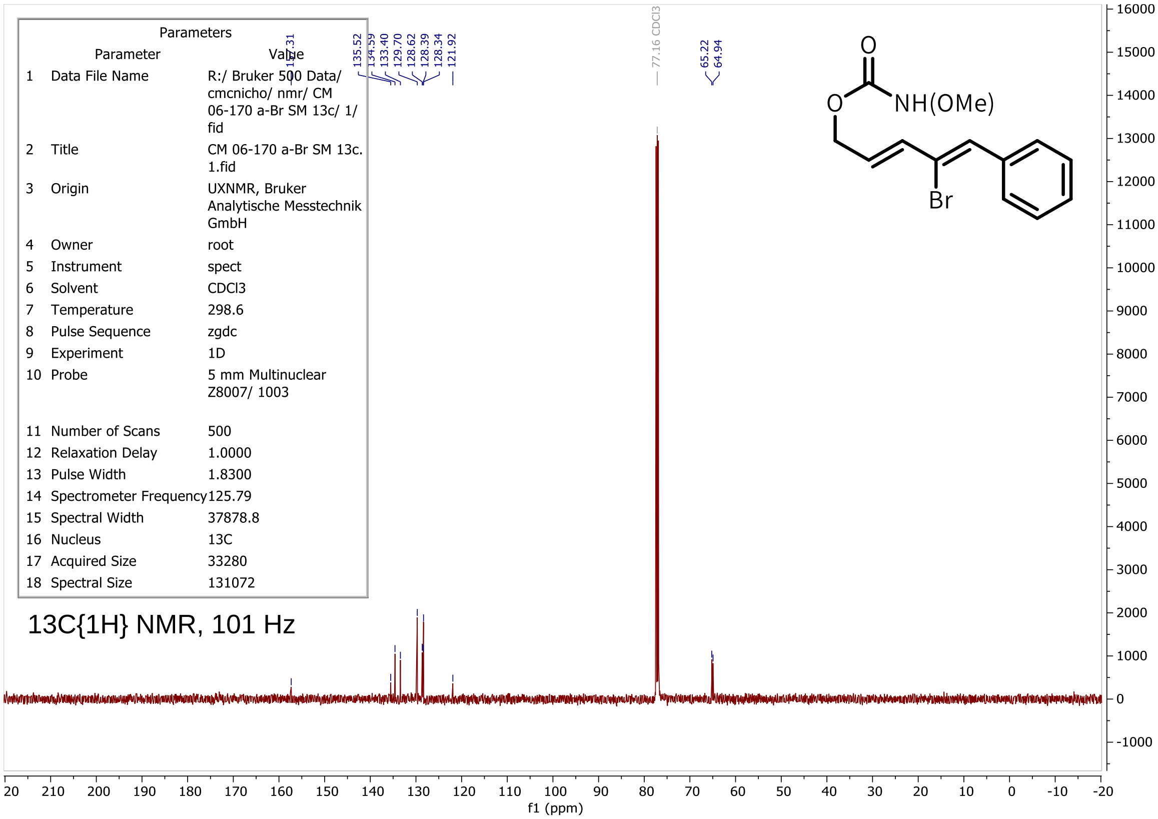

(E)-5-methylhexa-2,4-dien-1-yl methoxycarbamate (1i)

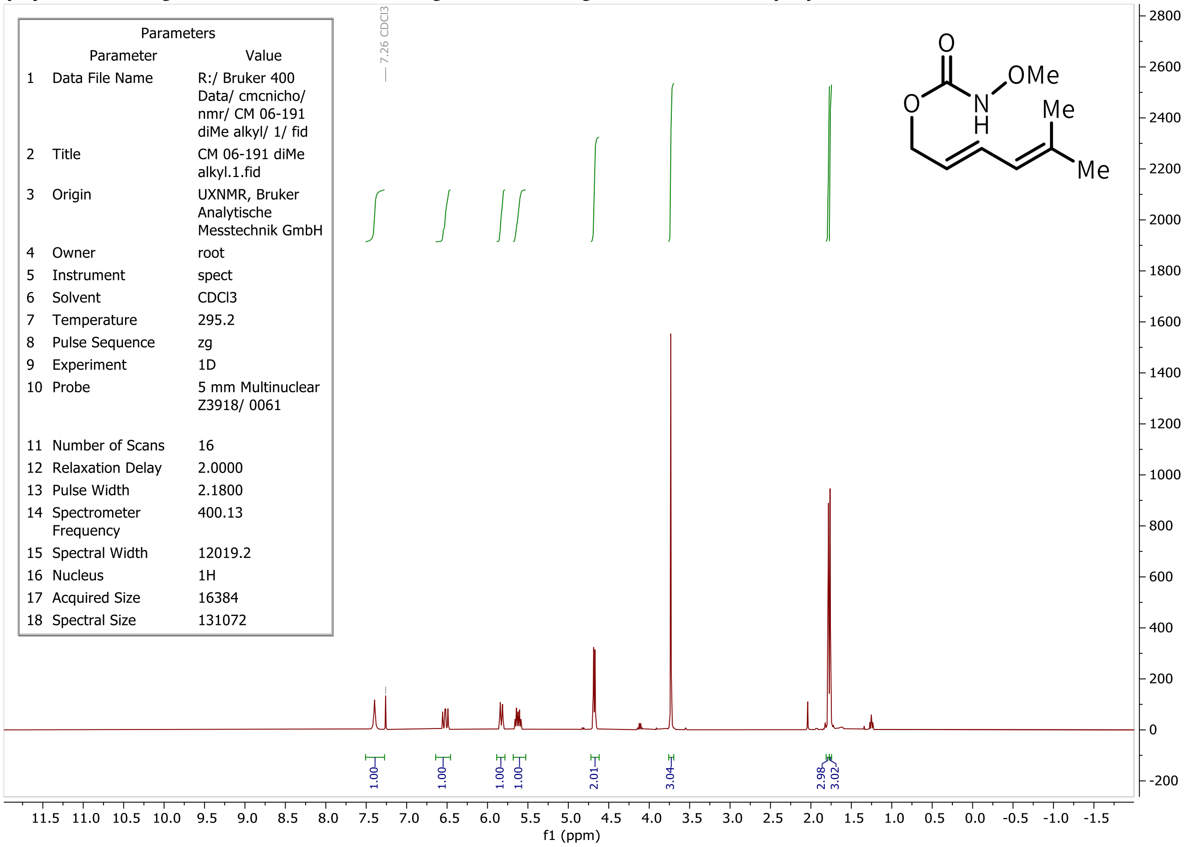

Parameters

	Parameter	Value
1	Data File Name	R:/ Bruker 400 Data/ cmcnicho/ nmr/ CM 06-191 diMe alkyl/ 1/ fid
2	Title	CM 06-191 diMe alkyl.1.fid
3	Origin	UXNMR, Bruker Analytische Messtechnik GmbH
4	Owner	root
5	Instrument	spect
6	Solvent	CDCl3
7	Temperature	295.2
8	Pulse Sequence	zg
9	Experiment	1D
10	Probe	5 mm Multinuclear Z3918/ 0061
11	Number of Scans	16
12	Relaxation Delay	2.0000
13	Pulse Width	2.1800
14	Spectrometer Frequency	400.13
15	Spectral Width	12019.2
16	Nucleus	1H
17	Acquired Size	16384
18	Spectral Size	131072

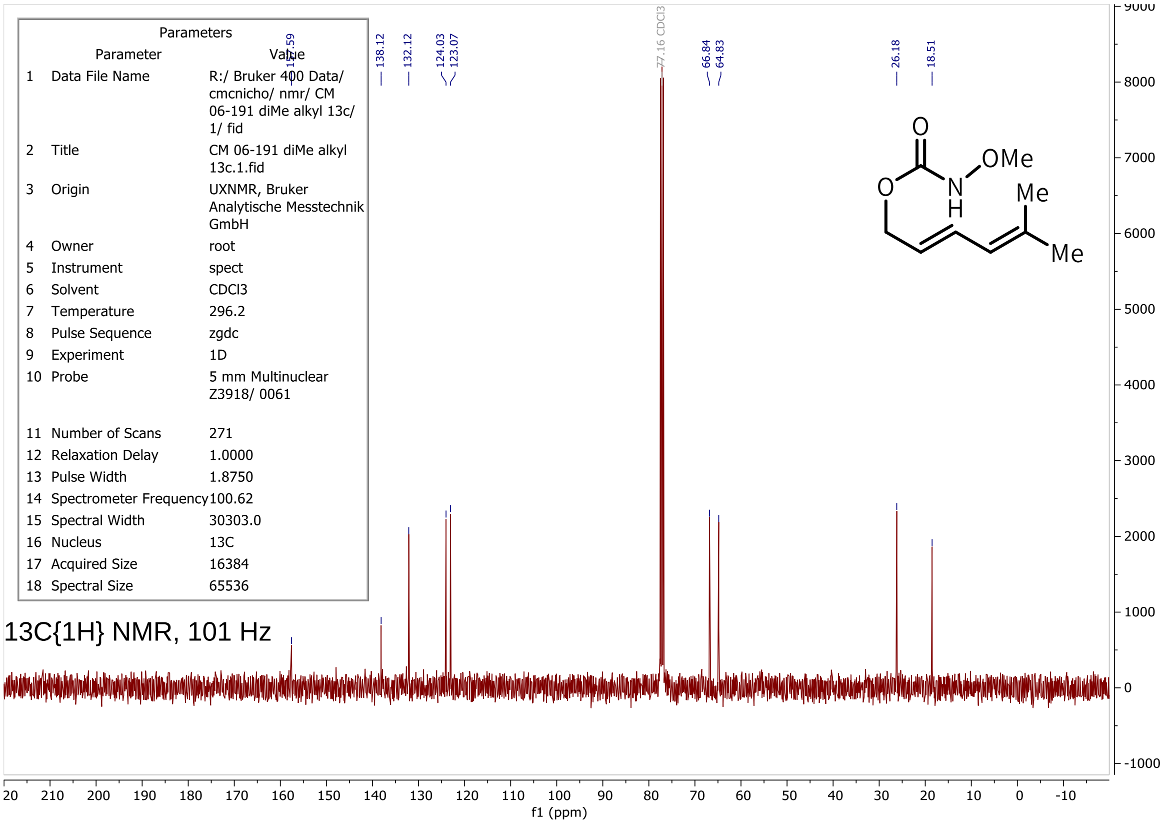

Parameters

	Parameter	Value
1	Data File Name	R:/ Bruker 400 Data/ cmcnicho/ nmr/ CM 06-191 diMe alkyl 13c/ 1/ fid
2	Title	CM 06-191 diMe alkyl 13c.1.fid
3	Origin	UXNMR, Bruker Analytische Messtechnik GmbH
4	Owner	root
5	Instrument	spect
6	Solvent	CDCl3
7	Temperature	296.2
8	Pulse Sequence	zgdc
9	Experiment	1D
10	Probe	5 mm Multinuclear Z3918/ 0061
11	Number of Scans	271
12	Relaxation Delay	1.0000
13	Pulse Width	1.8750
14	Spectrometer Frequency	100.62
15	Spectral Width	30303.0
16	Nucleus	13C
17	Acquired Size	16384
18	Spectral Size	65536

(2*E*,4*E*)-hexa-2,4-dien-1-yl methoxycarbamate (1j)

	Parameter	Value
1	Data File Name	R:/ Bruker 400 Data/ cmcnicho/ nmr/ CM 06-186 alkyl diene/ 1/ fid
2	Title	CM 06-186 alkyl diene.1.fid
3	Origin	UXNMR, Bruker Analytische Messtechnik GmbH
4	Owner	root
5	Instrument	spect
6	Solvent	CDCl3
7	Temperature	295.2
8	Pulse Sequence	zg
9	Experiment	1D
10	Probe	5 mm Multinuclear Z3918/ 0061
11	Number of Scans	16
12	Relaxation Delay	2.0000
13	Pulse Width	2.1800
14	Spectrometer Frequency	400.13
15	Spectral Width	12019.2
16	Nucleus	1H
17	Acquired Size	16384
18	Spectral Size	131072

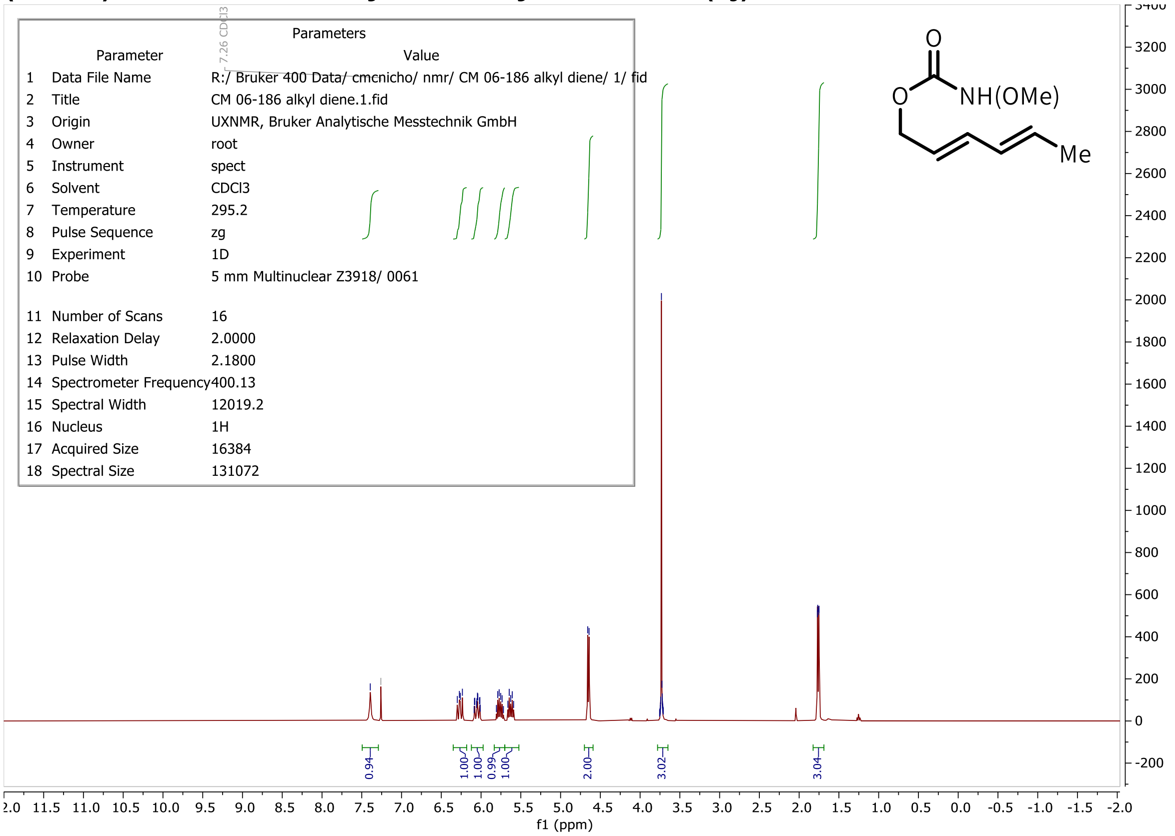

	Parameter	Value
1	Data File Name	R:/ Bruker 400 Data/ cmcnicho/ nmr/ CM 06-186 alkyl diene 13c/ 1/ fid
2	Title	CM 06-186 alkyl diene 13c.1.fid
3	Origin	UXNMR, Bruker Analytische Messtechnik GmbH
4	Owner	root
5	Instrument	spect
6	Solvent	CDCl3
7	Temperature	296.2
8	Pulse Sequence	zgdc
9	Experiment	1D
10	Probe	5 mm Multinuclear Z3918/ 0061
11	Number of Scans	300
12	Relaxation Delay	1.0000
13	Pulse Width	1.8750
14	Spectrometer Frequency	100.62
15	Spectral Width	30303.0
16	Nucleus	13C
17	Acquired Size	16384
18	Spectral Size	65536

13C{1H} NMR, 101 Hz

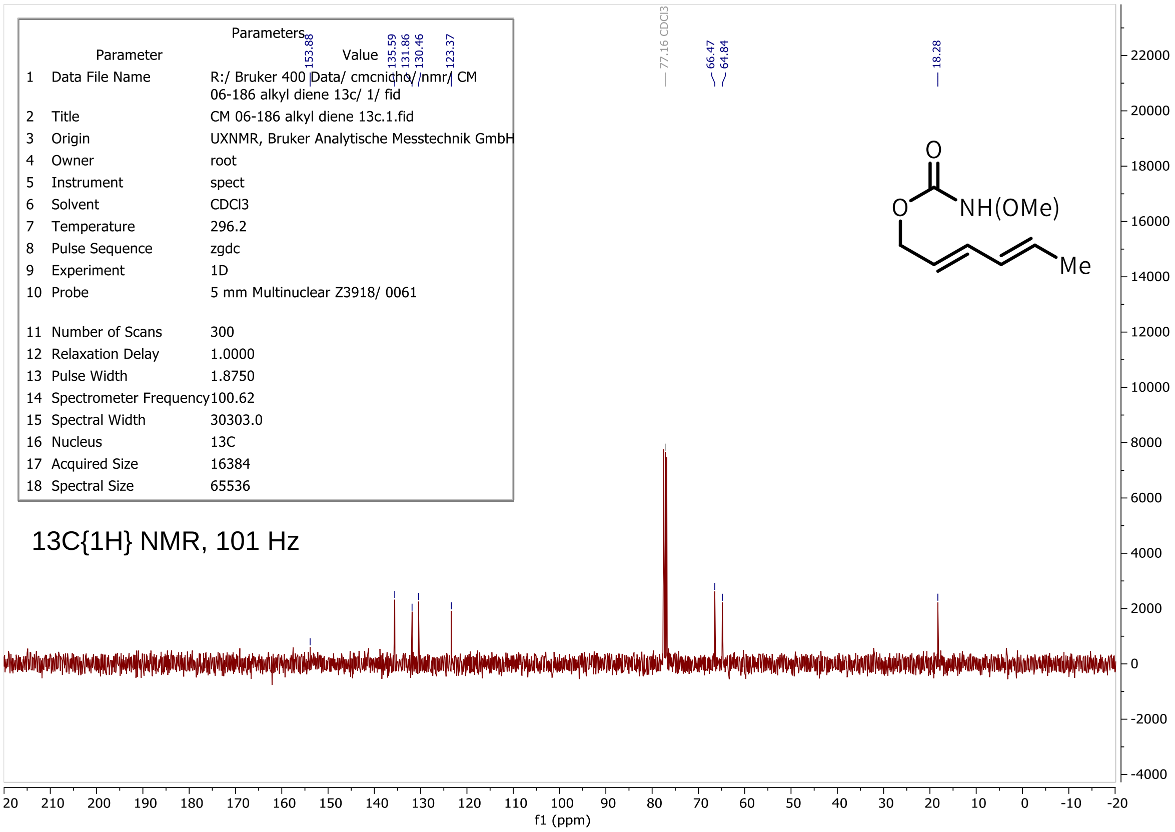

(2*E*,4*E*)-5-(furan-2-yl)penta-2,4-dien-1-yl methoxycarbamate (1k)

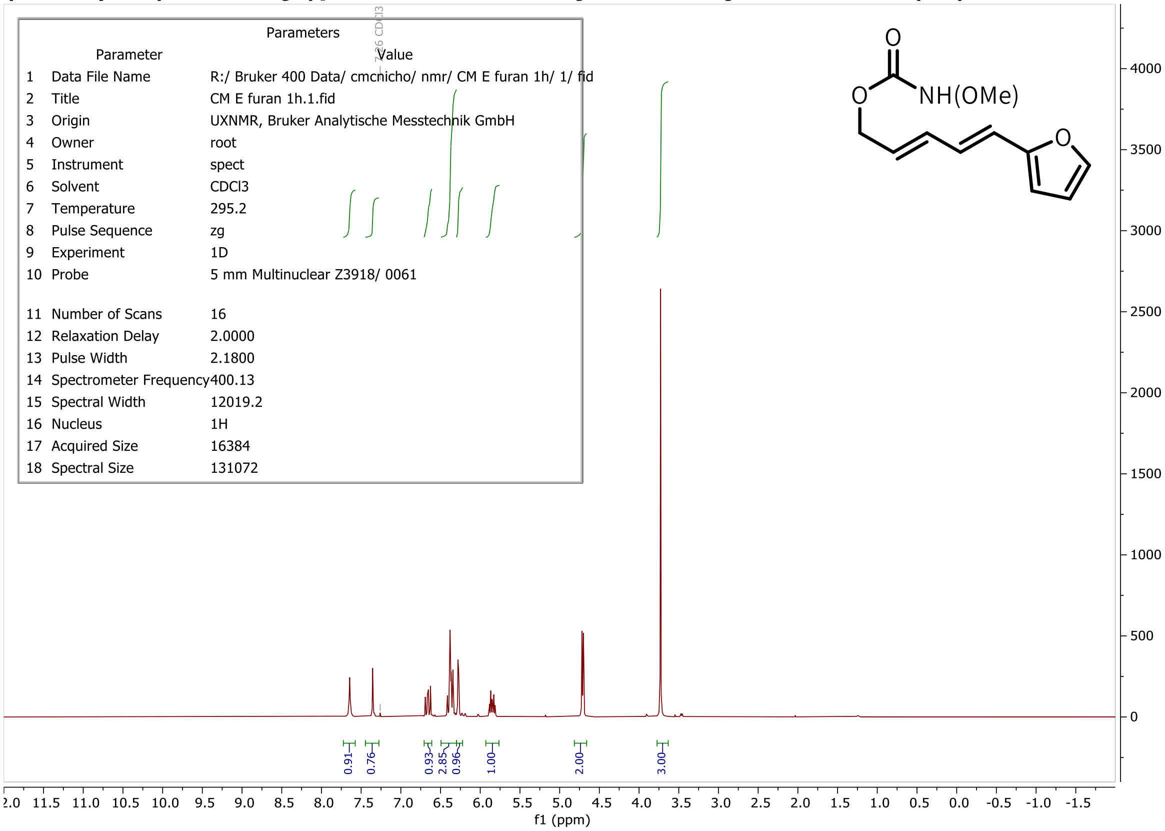

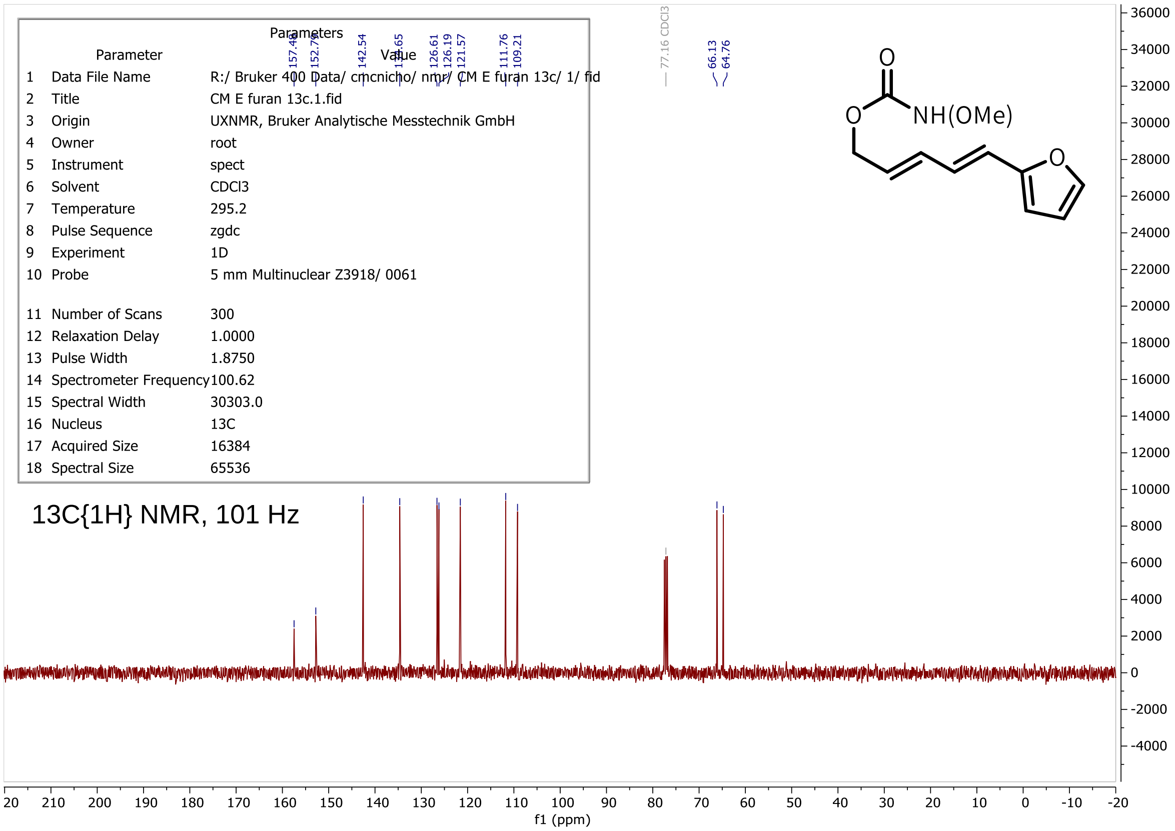

(2*E*,4*E*)-5-(pyridin-2-yl)penta-2,4-dien-1-yl methoxycarbamate (1l)

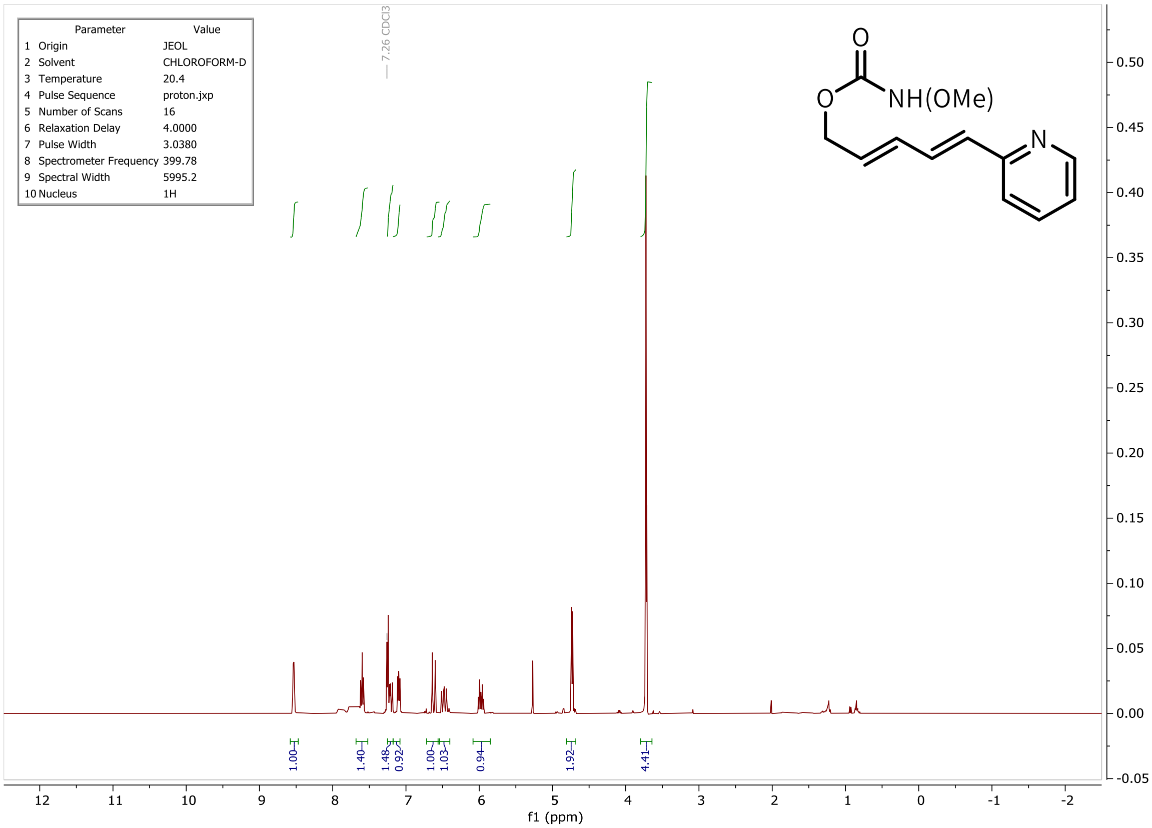

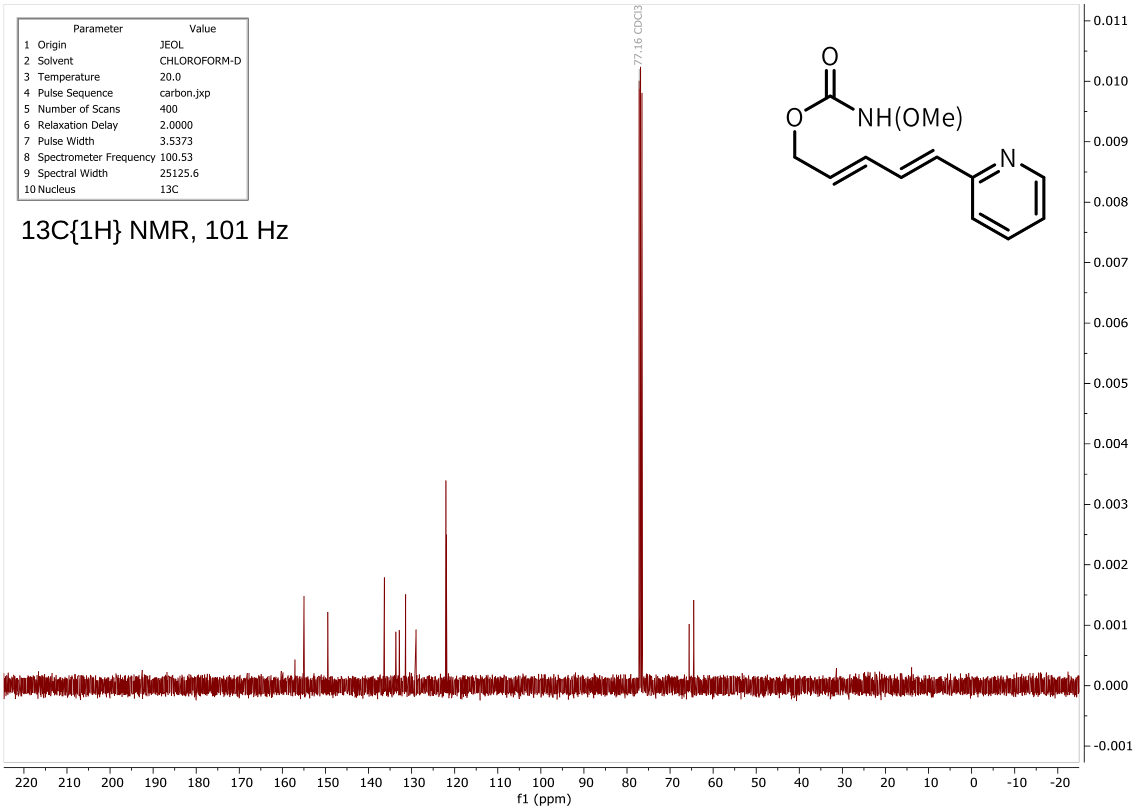

(2E,4E)-5-(3-(trifluoromethyl)phenyl)penta-2,4-dien-1-yl methoxycarbamate (1m)

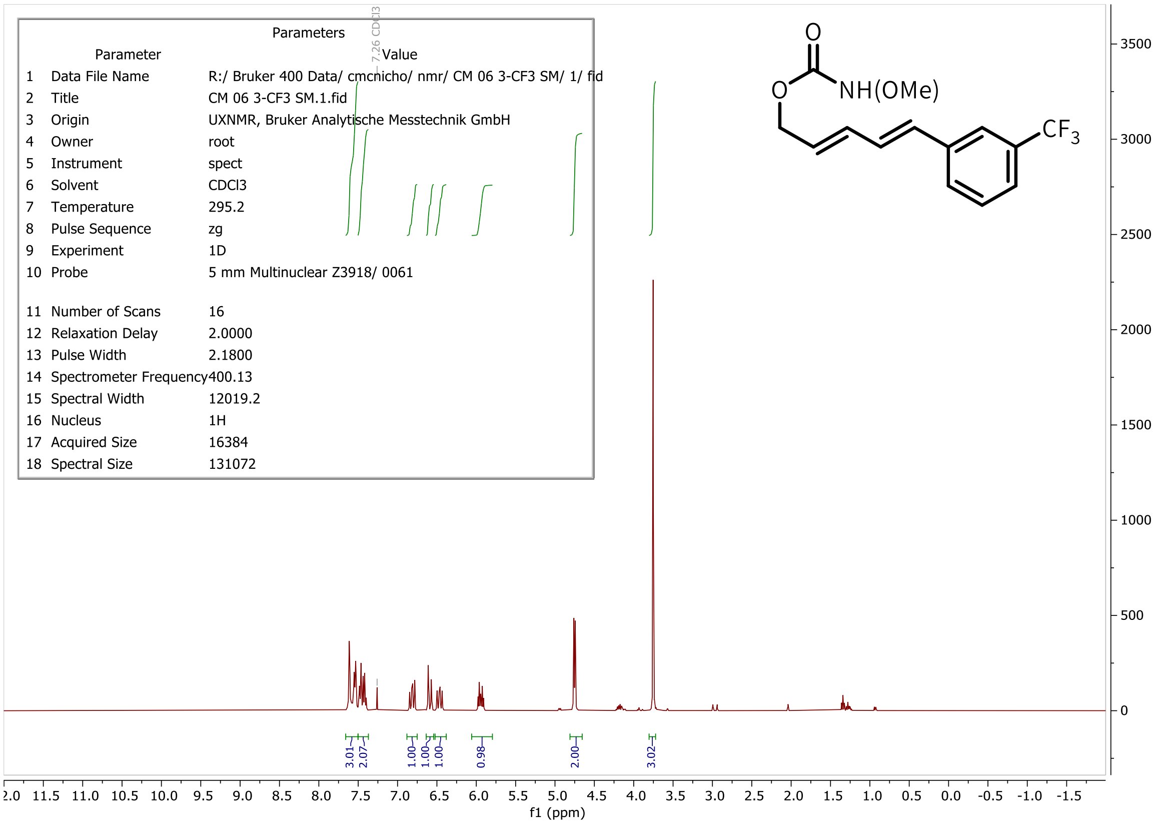

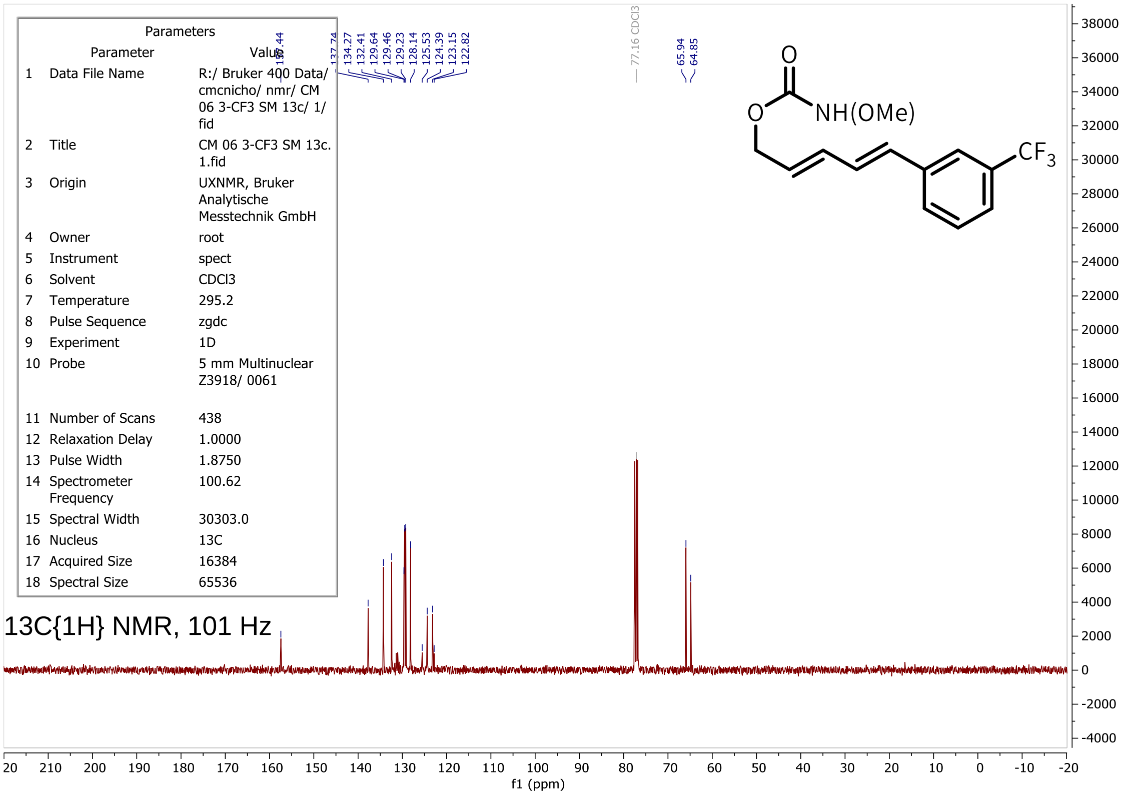

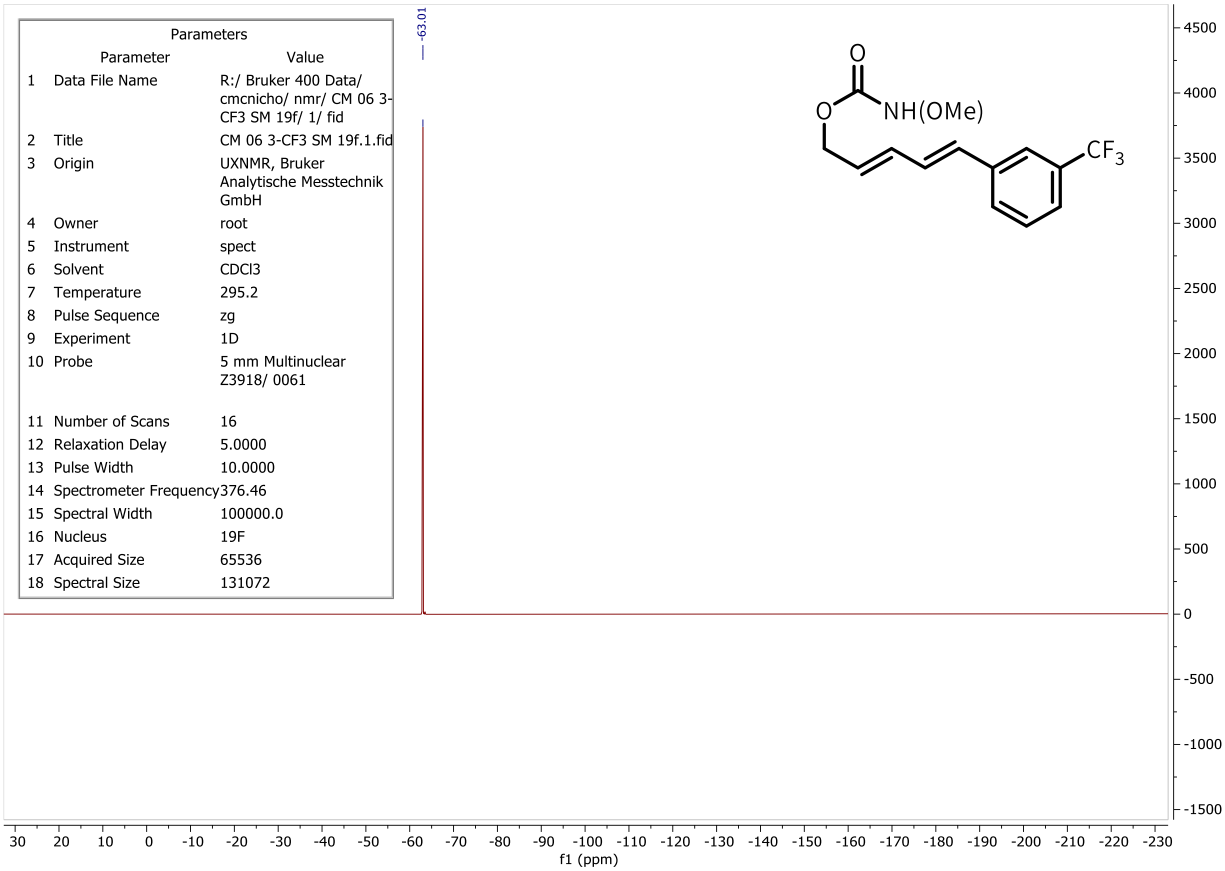
Parameters
Parameter Value
1 Data File Name R:/ Bruker 400 Data/ cmcnicho/ nmr/ CM 06 3-CF3 SM 19f/ 1/ fid
2 Title CM 06 3-CF3 SM 19f.1.fid
3 Origin UXNMR, Bruker Analytische Messtechnik GmbH
4 Owner root
5 Instrument spect
6 Solvent CDCl3
7 Temperature 295.2
8 Pulse Sequence zg
9 Experiment 1D
10 Probe 5 mm Multinuclear Z3918/ 0061
11 Number of Scans 16
12 Relaxation Delay 5.0000
13 Pulse Width 10.0000
14 Spectrometer Frequency 376.46
15 Spectral Width 100000.0
16 Nucleus 19F
17 Acquired Size 65536
18 Spectral Size 131072
-63.01
O
O
NH(OMe)
CF3
f1 (ppm)

(2E,4E)-5-(benzo[d][1,3]dioxol-5-yl)penta-2,4-dien-1-yl methoxycarbamate (1n)

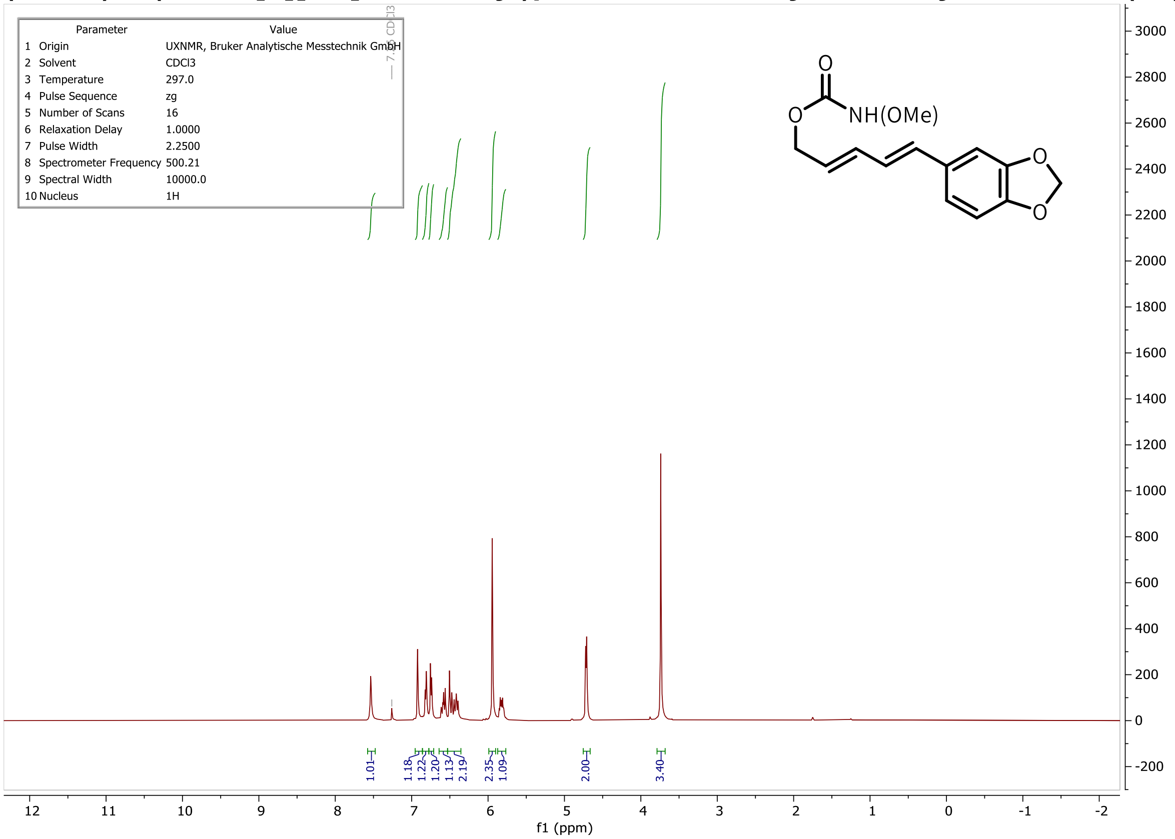

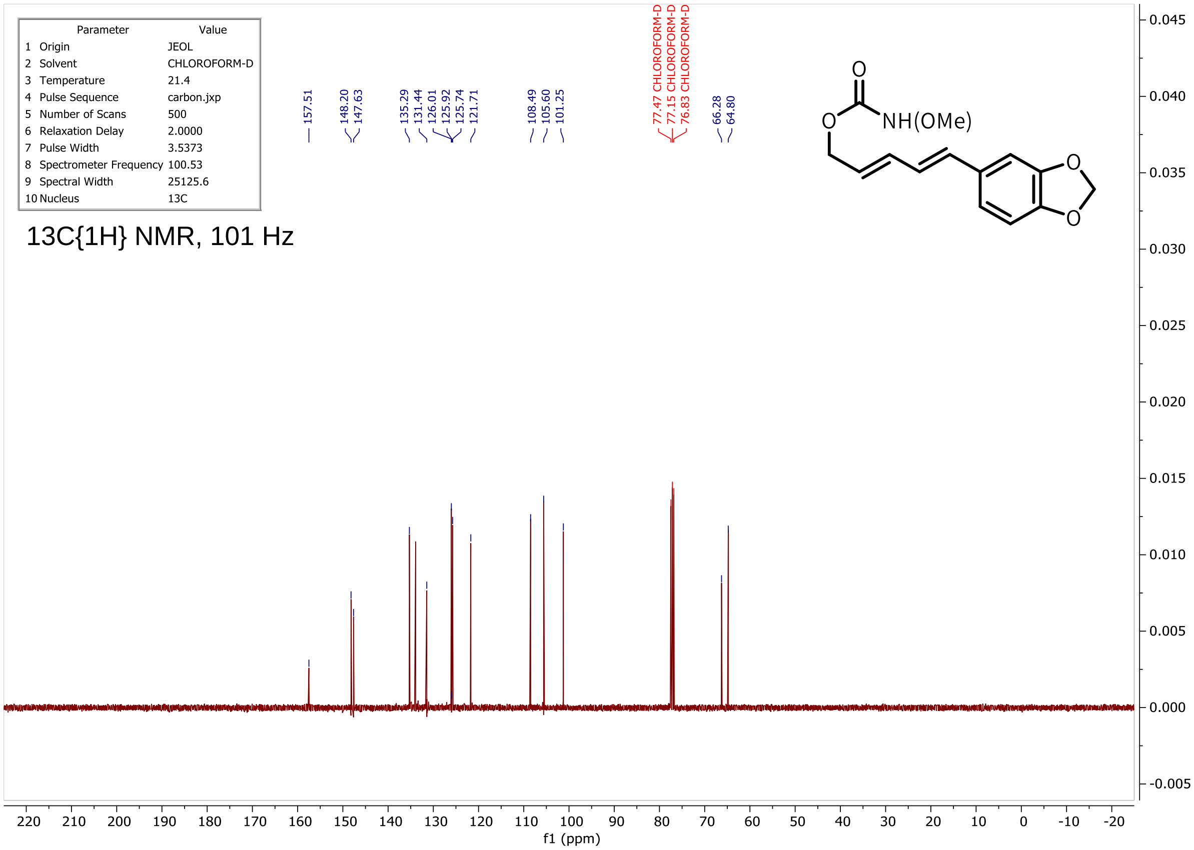

(*E*)-4-(3-(4-fluorophenyl)-3-oxoprop-1-en-1-yl)-3-methoxyoxazolidin-2-one (2a)

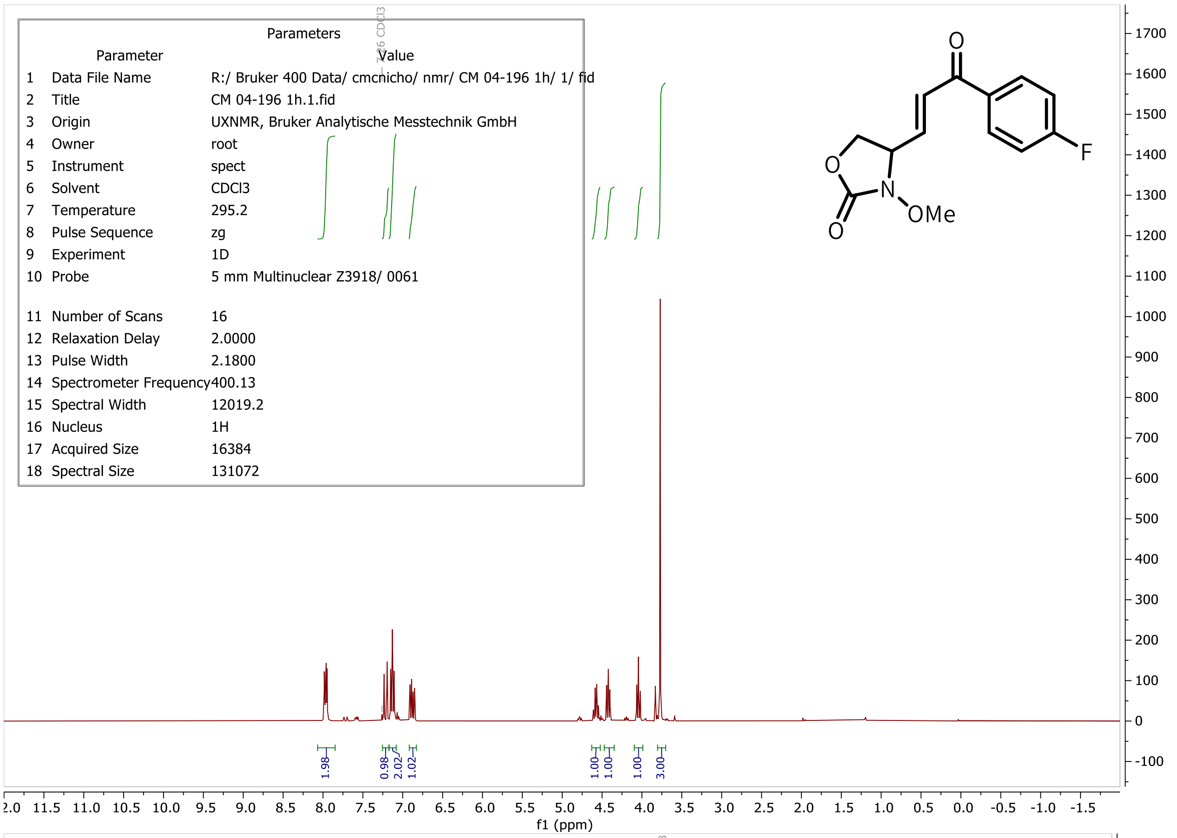

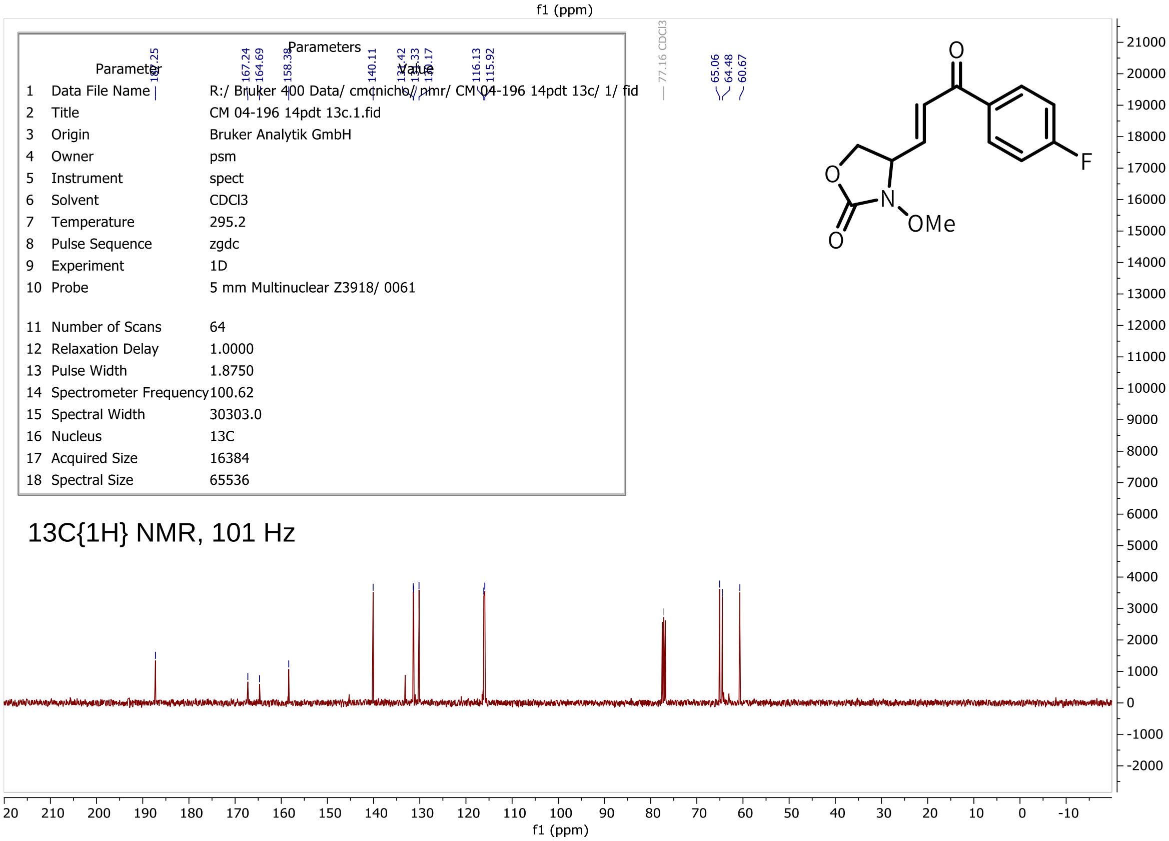

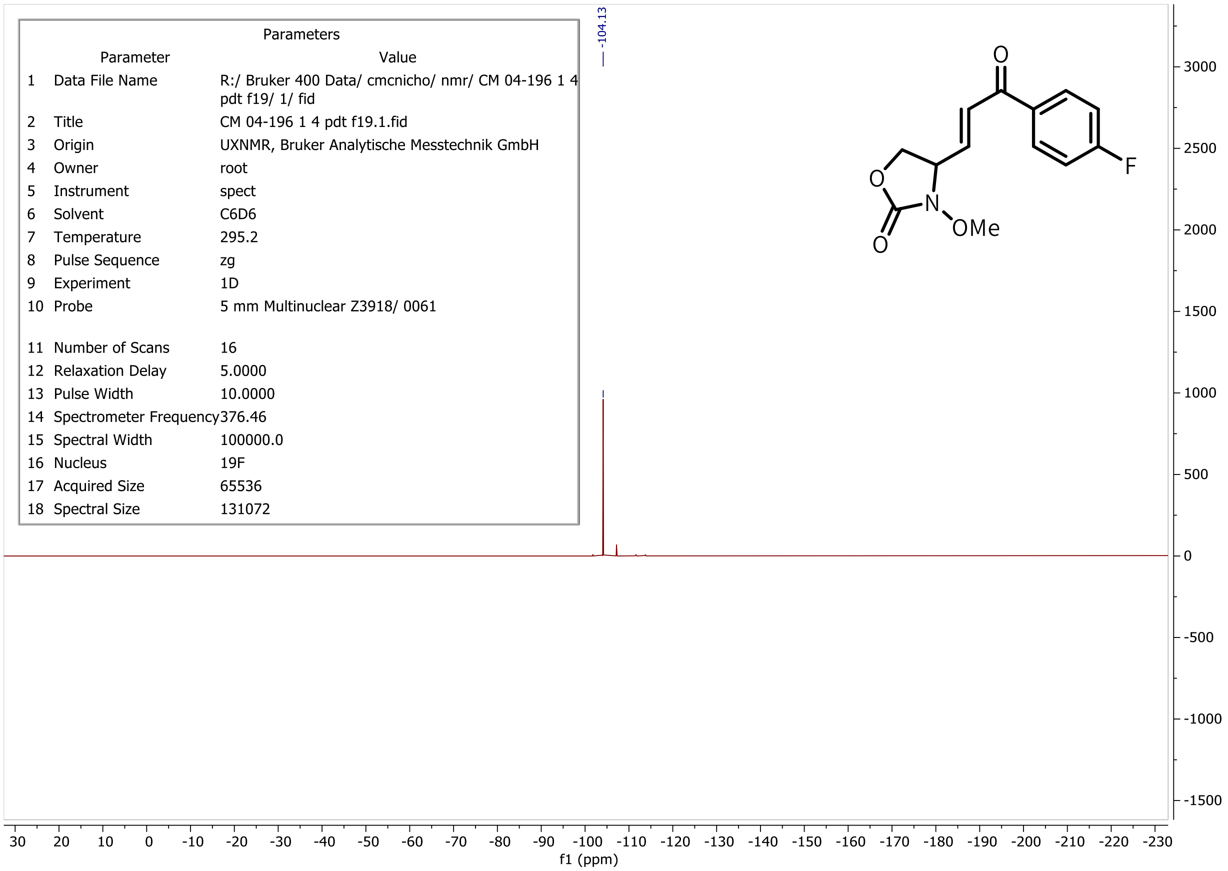

	Parameter	Value
1	Data File Name	R:/ Bruker 400 Data/ cmcnicho/ nmr/ CM 04-196 1 4 pdt f19/ 1/ fid
2	Title	CM 04-196 1 4 pdt f19.1.fid
3	Origin	UXNMR, Bruker Analytische Messtechnik GmbH
4	Owner	root
5	Instrument	spect
6	Solvent	C6D6
7	Temperature	295.2
8	Pulse Sequence	zg
9	Experiment	1D
10	Probe	5 mm Multinuclear Z3918/ 0061
11	Number of Scans	16
12	Relaxation Delay	5.0000
13	Pulse Width	10.0000
14	Spectrometer Frequency	376.46
15	Spectral Width	100000.0
16	Nucleus	19F
17	Acquired Size	65536
18	Spectral Size	131072

(*E*)-4-(3-(4-fluorophenyl)acryloyl)-3-methoxyoxazolidin-2-one (2a')

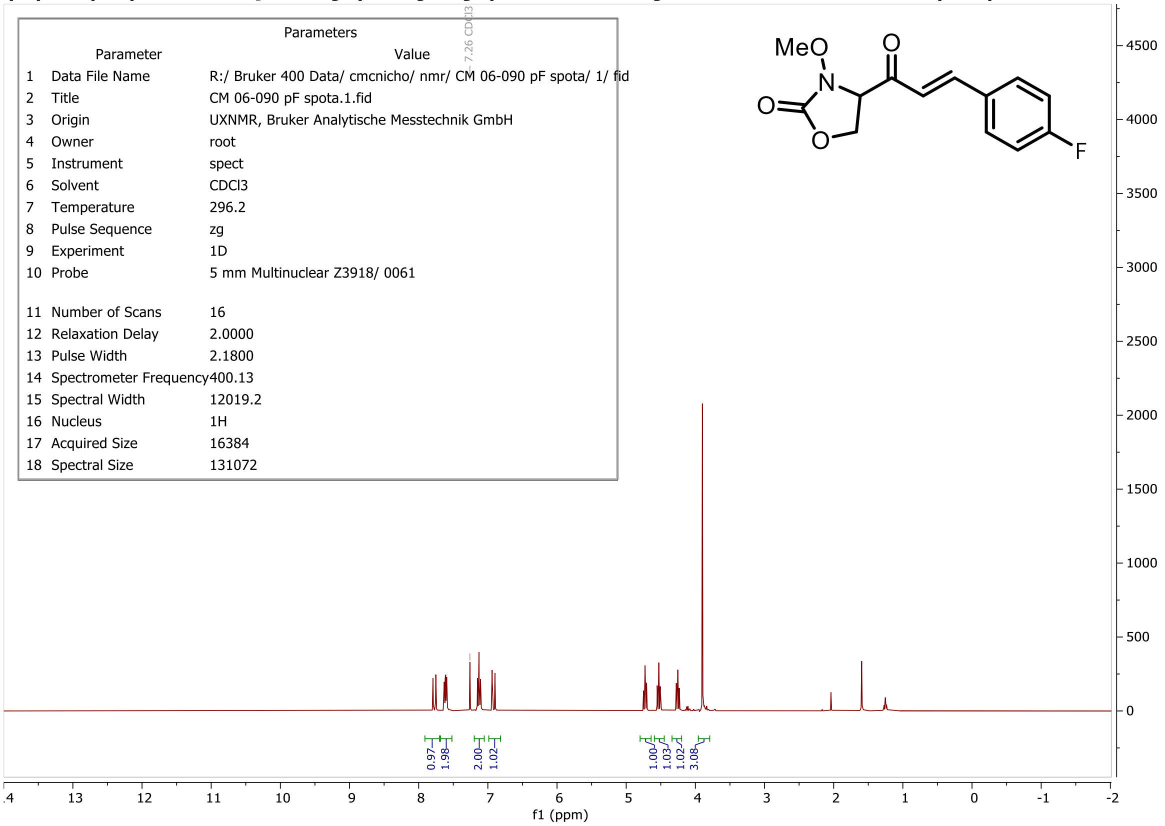

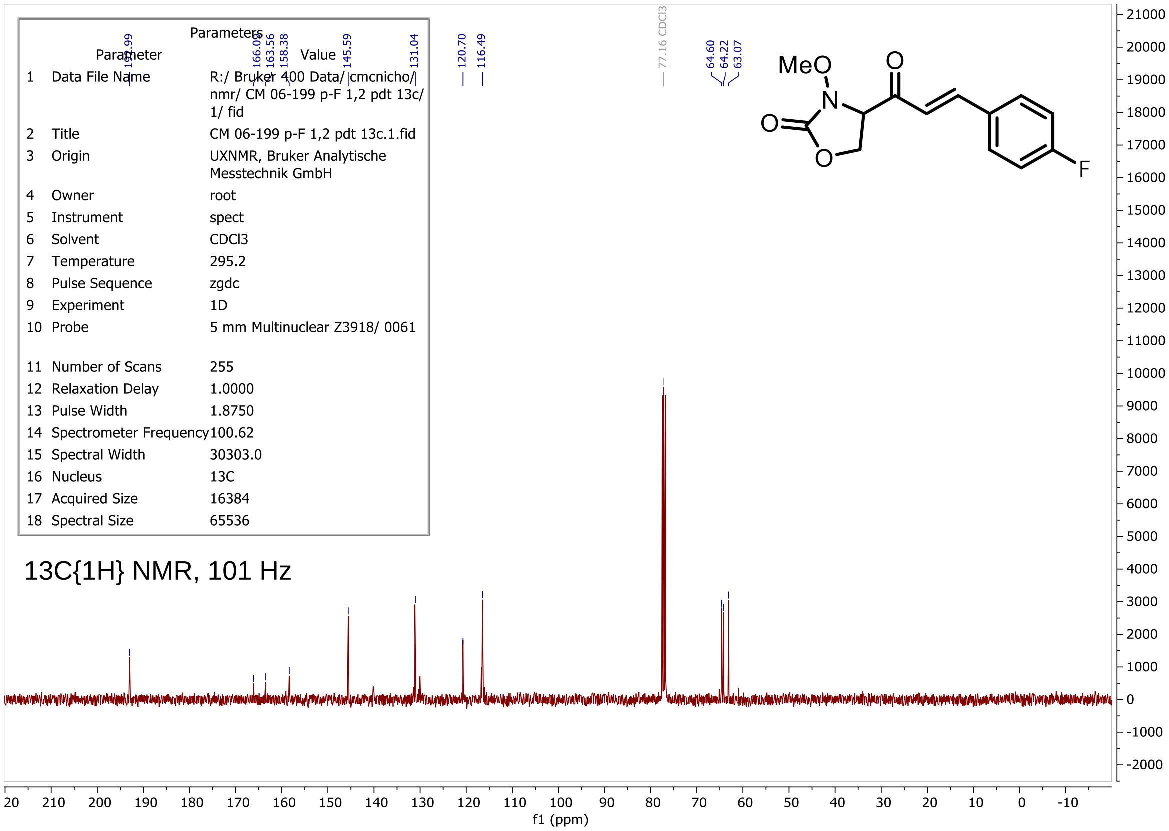

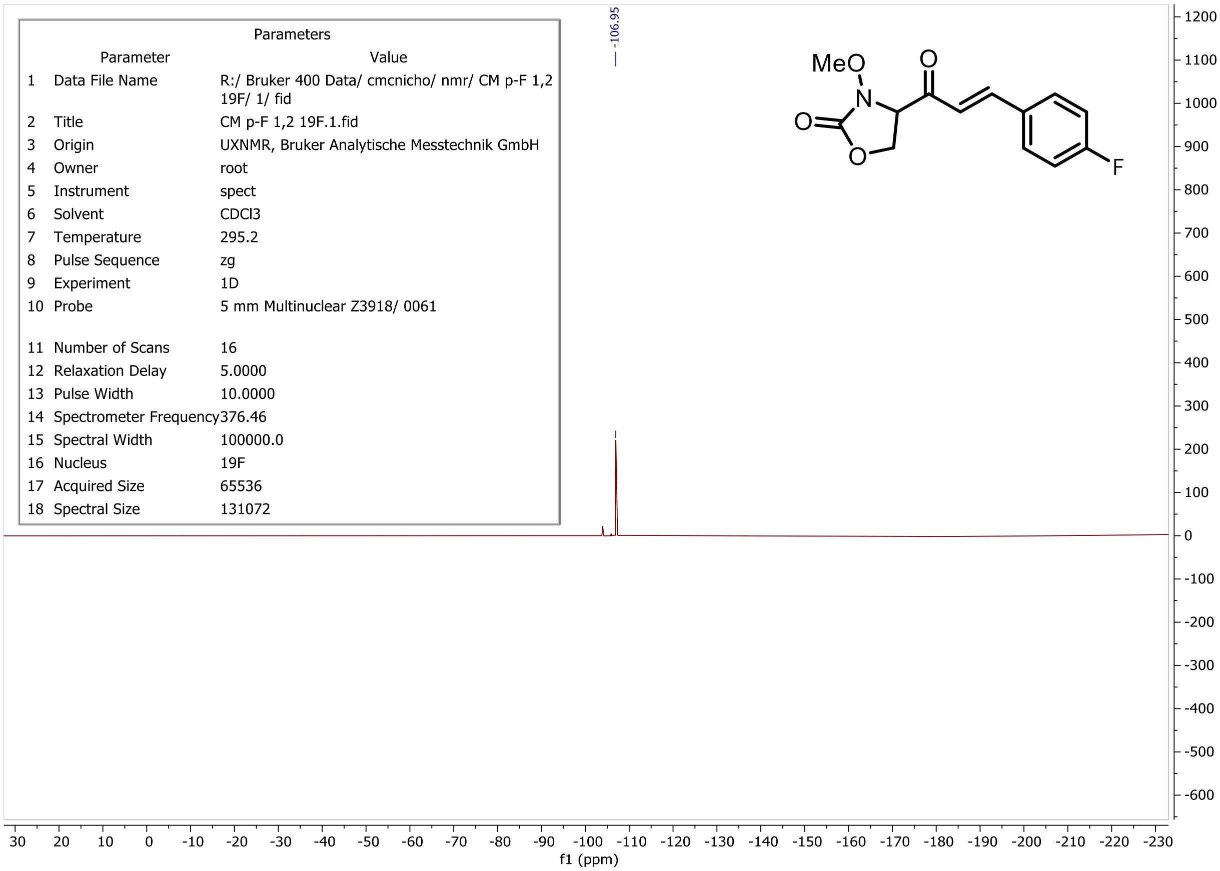

Parameters

	Parameter	Value
1	Data File Name	R:/ Bruker 400 Data/ cmcnicho/ nmr/ CM p-F 1,2 19F/ 1/ fid
2	Title	CM p-F 1,2 19F.1.fid
3	Origin	UXNMR, Bruker Analytische Messtechnik GmbH
4	Owner	root
5	Instrument	spect
6	Solvent	CDCl3
7	Temperature	295.2
8	Pulse Sequence	zg
9	Experiment	1D
10	Probe	5 mm Multinuclear Z3918/ 0061
11	Number of Scans	16
12	Relaxation Delay	5.0000
13	Pulse Width	10.0000
14	Spectrometer Frequency	376.46
15	Spectral Width	100000.0
16	Nucleus	19F
17	Acquired Size	65536
18	Spectral Size	131072

(*E*)-3-methoxy-4-(3-oxo-3-phenylprop-1-en-1-yl)oxazolidin-2-one (2b)

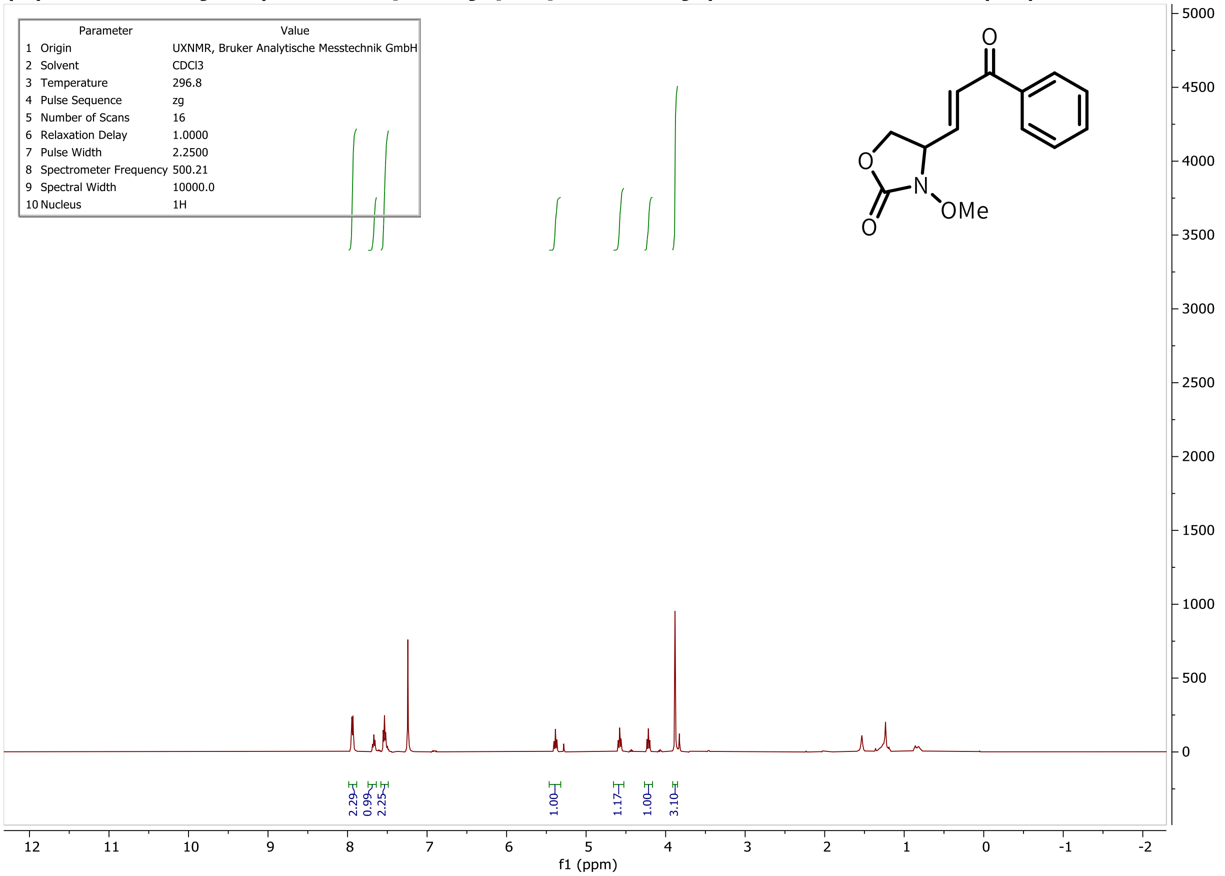

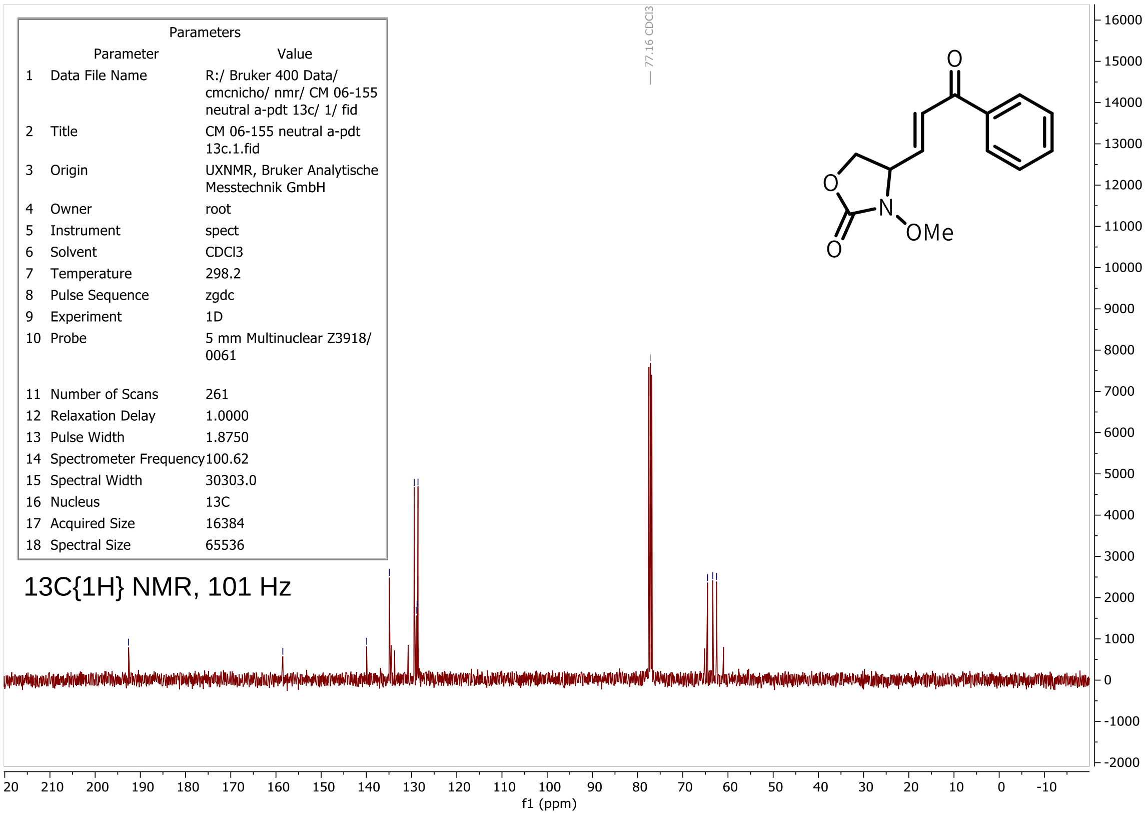

(*E*)-4-cinnamoyl-3-methoxyoxazolidin-2-one (2b')

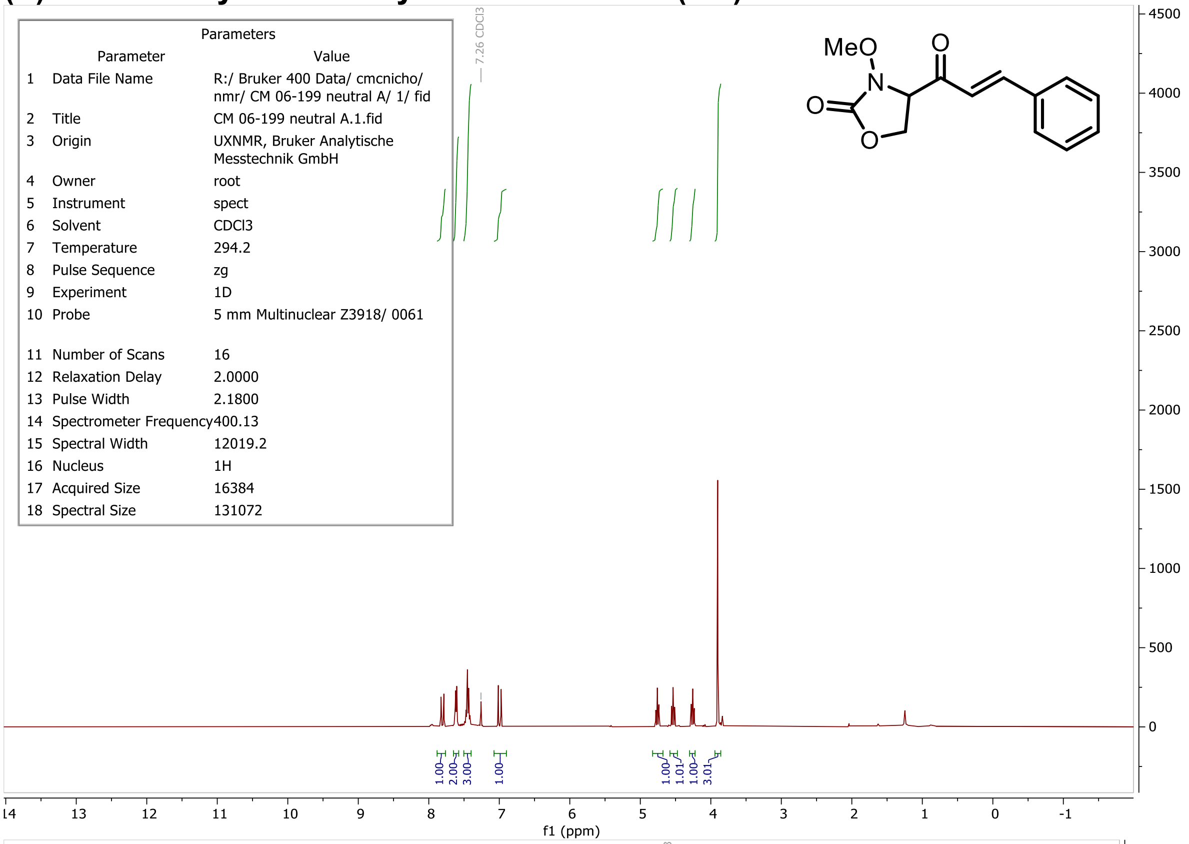

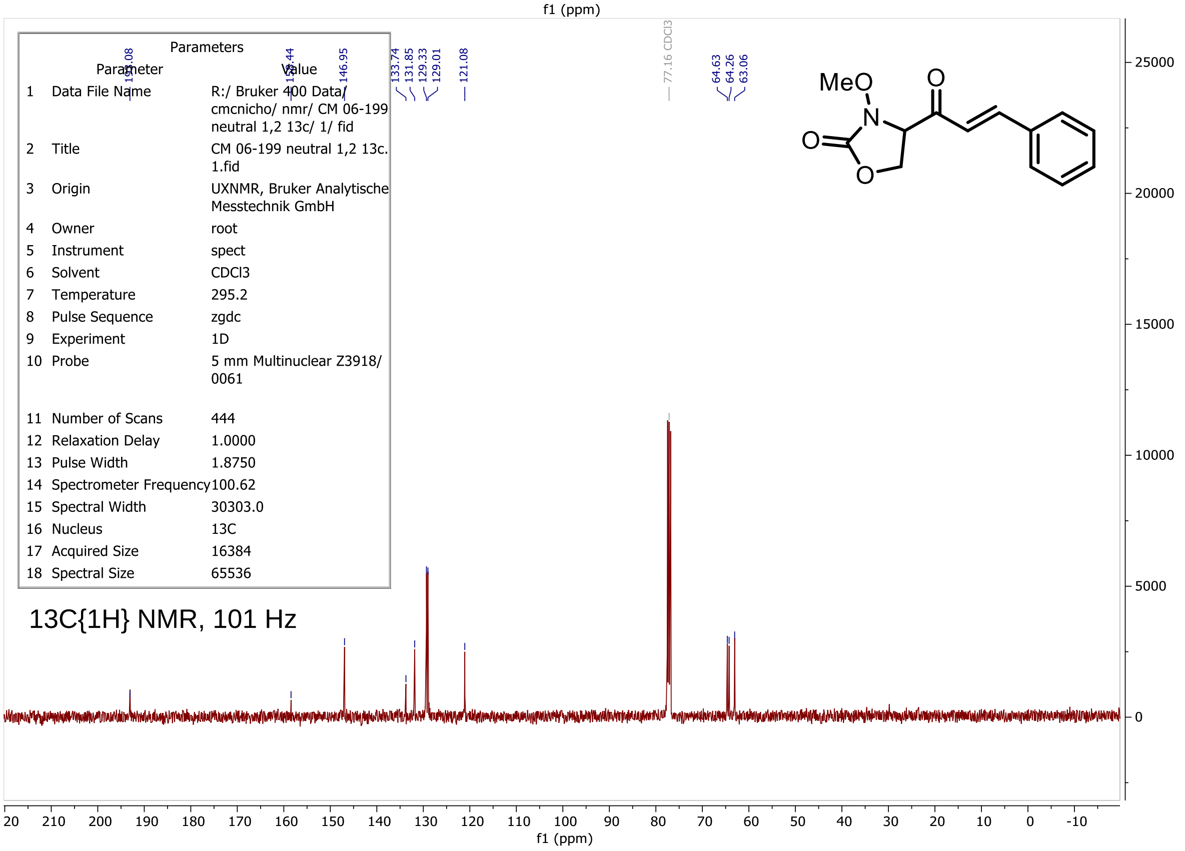

(*E*)-3-methoxy-4-(3-(4-methoxyphenyl)-3-oxoprop-1-en-1-yl)oxazolidin-2-one (2c)

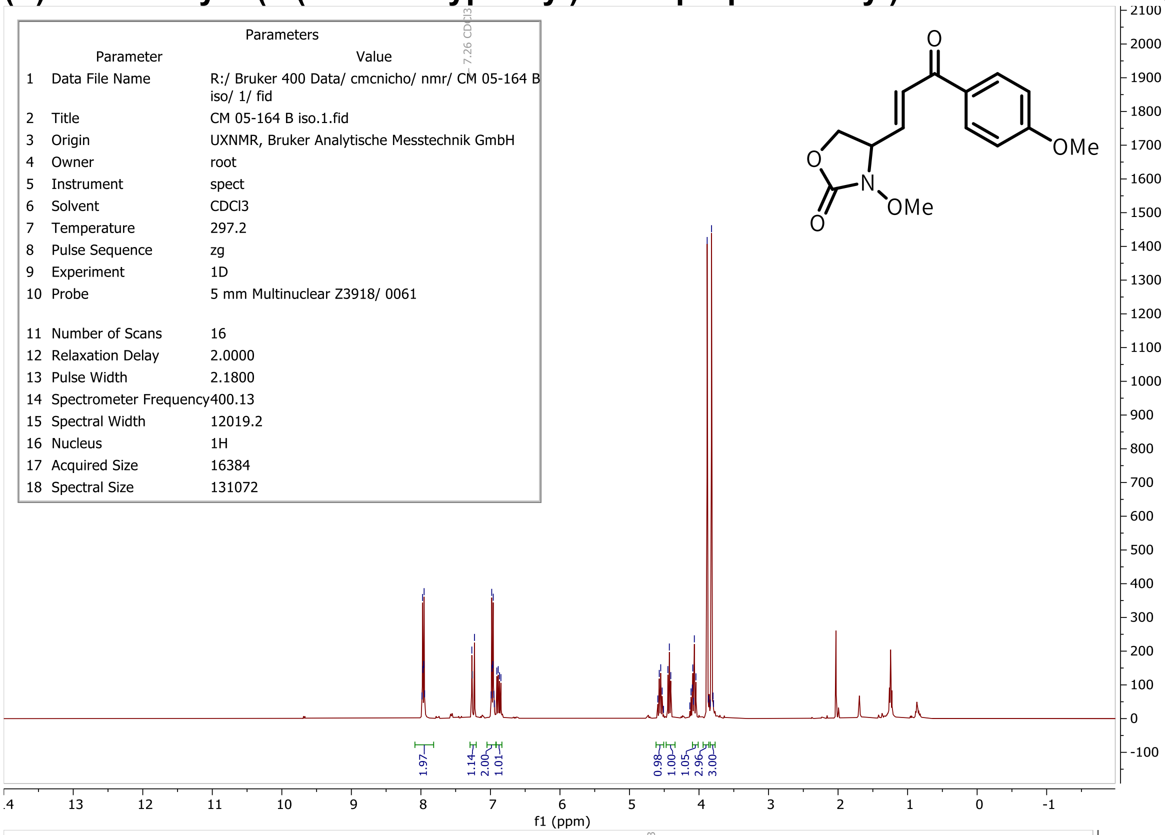

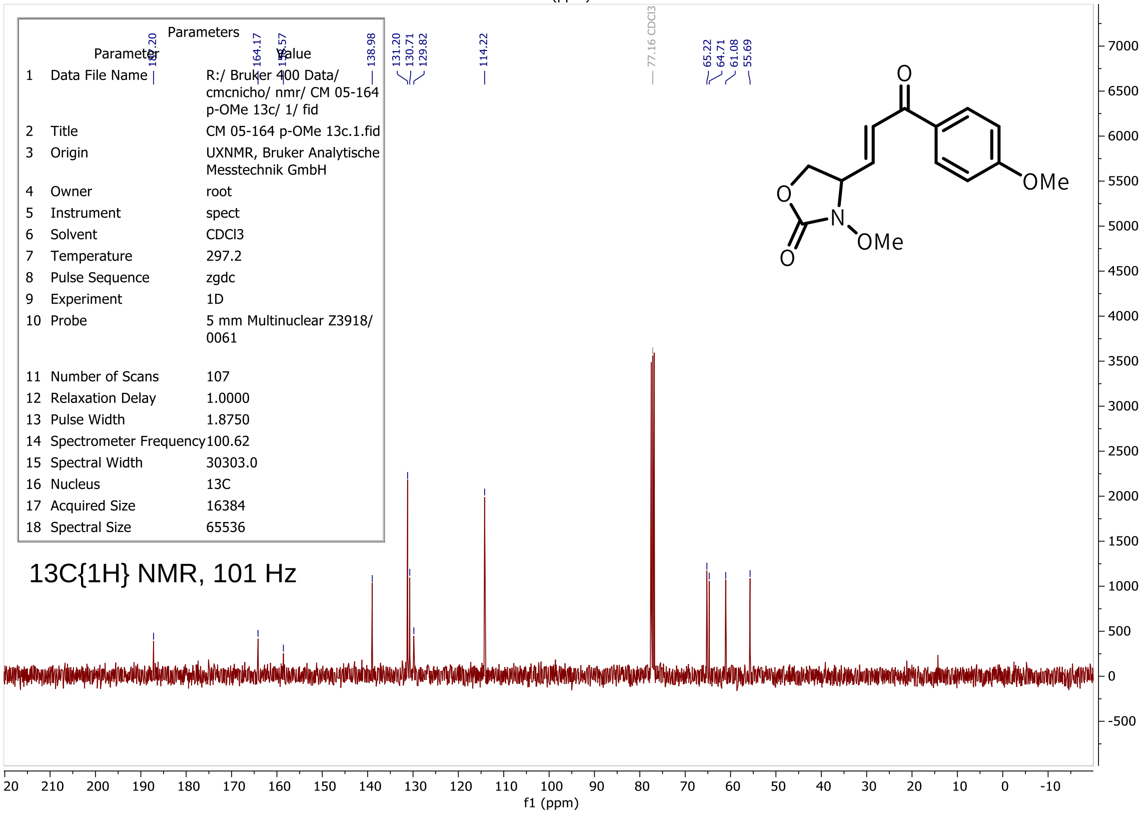

(*E*)-3-methoxy-4-(3-(4-methoxyphenyl)acryloyl)oxazolidin-2-one (2c')

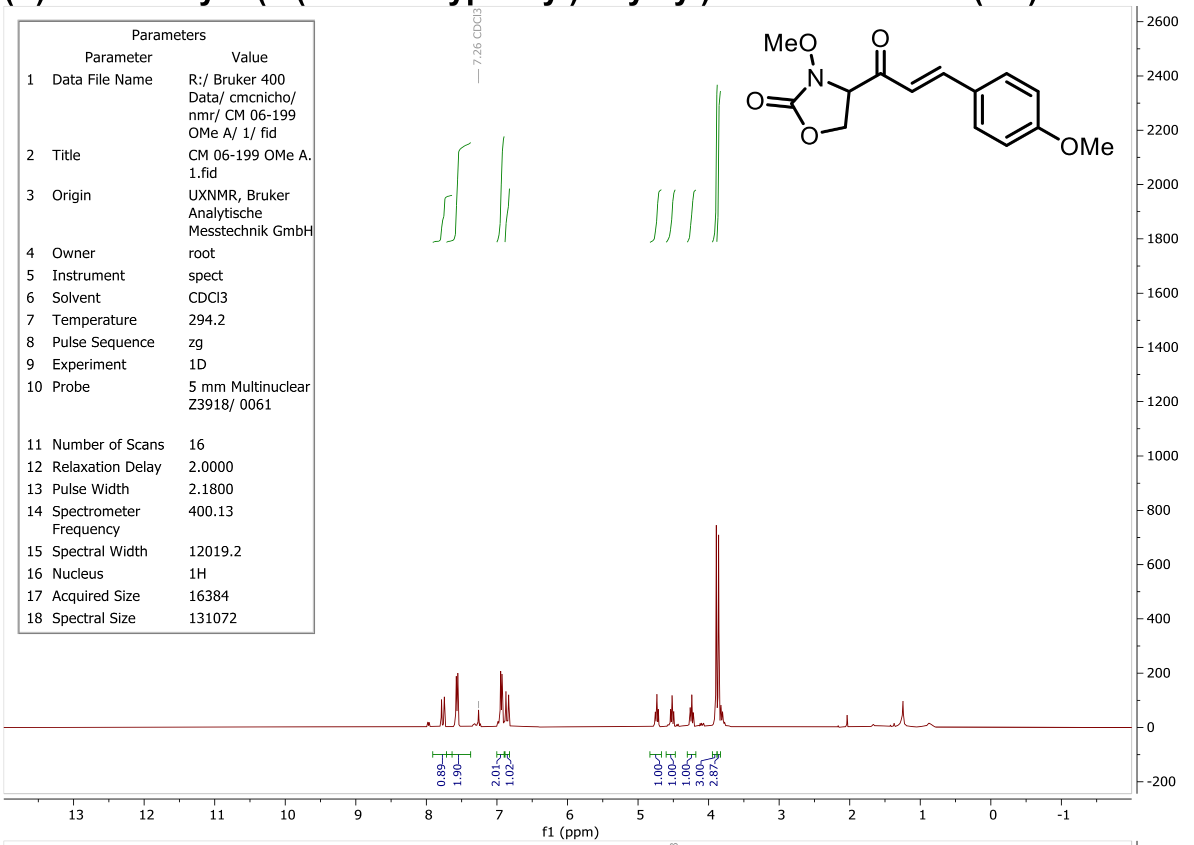

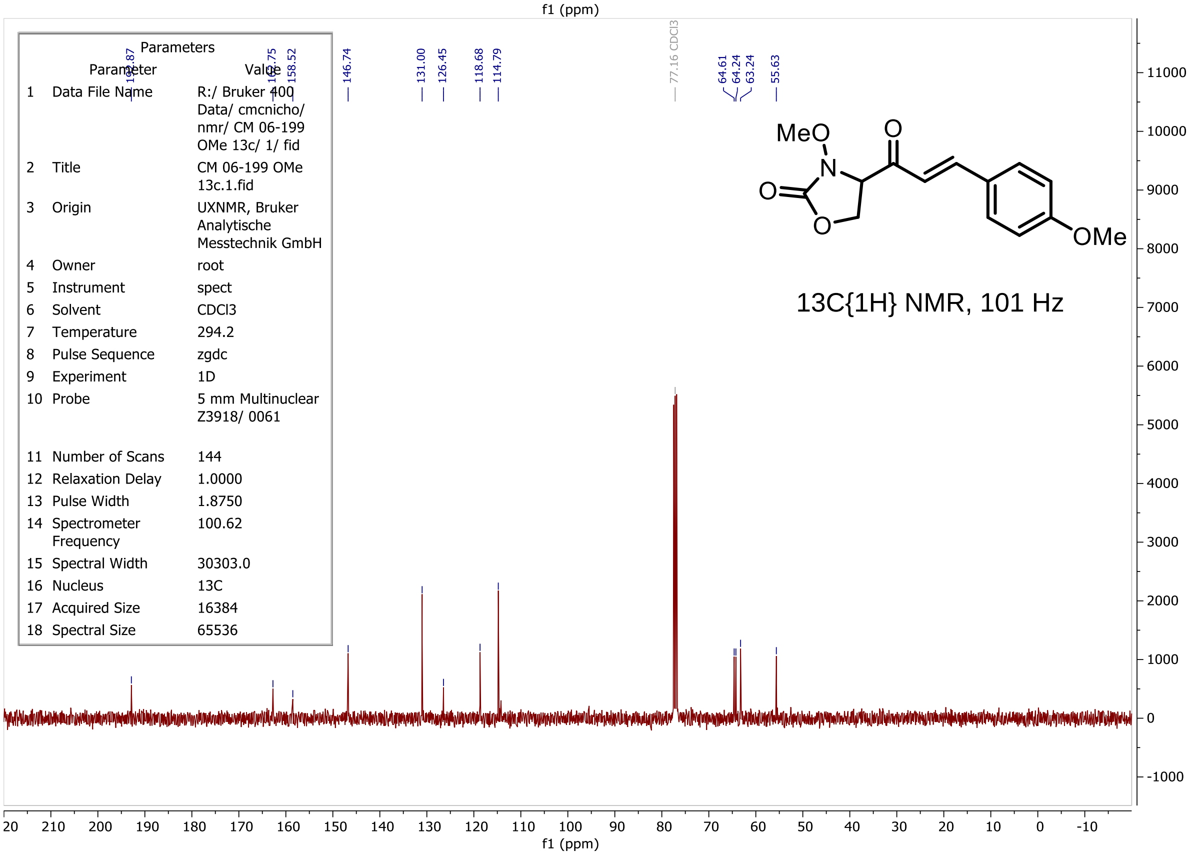

(*E*)-3-methoxy-4-(3-oxo-3-(p-tolyl)prop-1-en-1-yl)oxazolidin-2-one (2d)

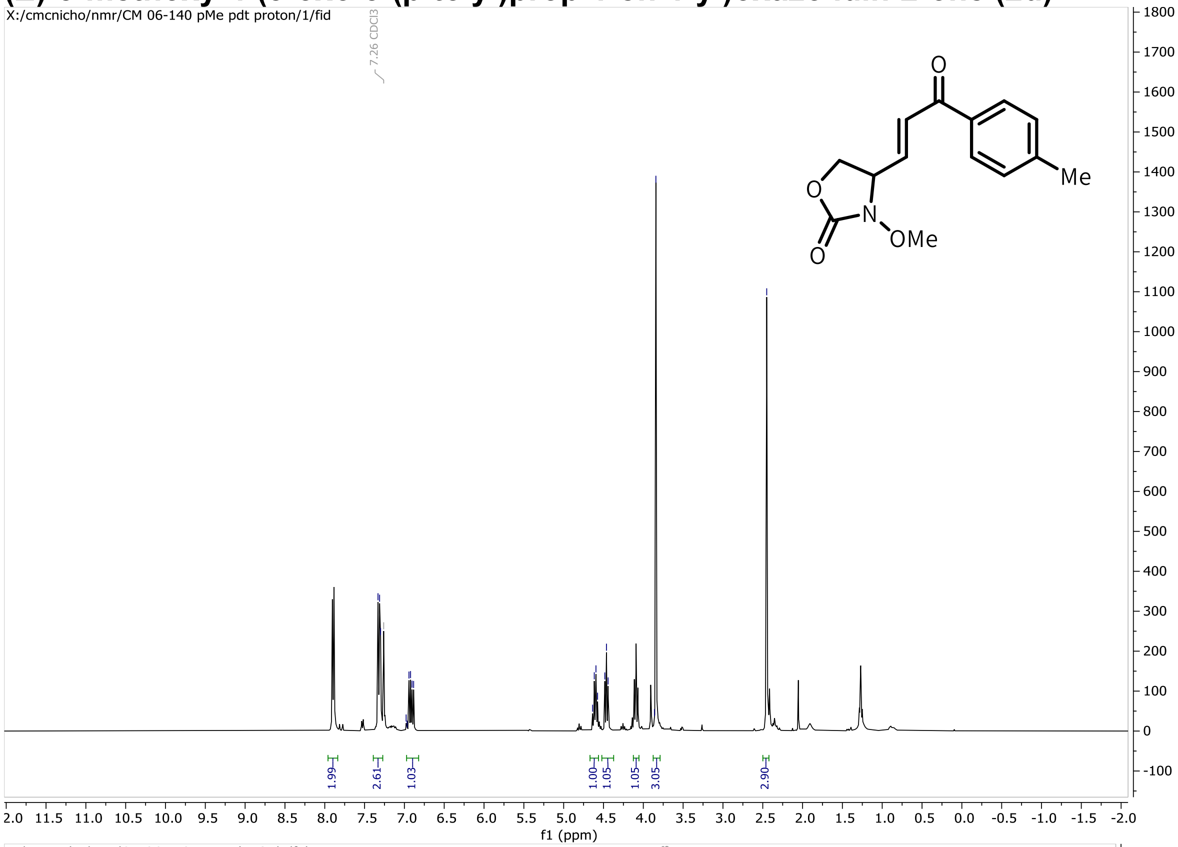

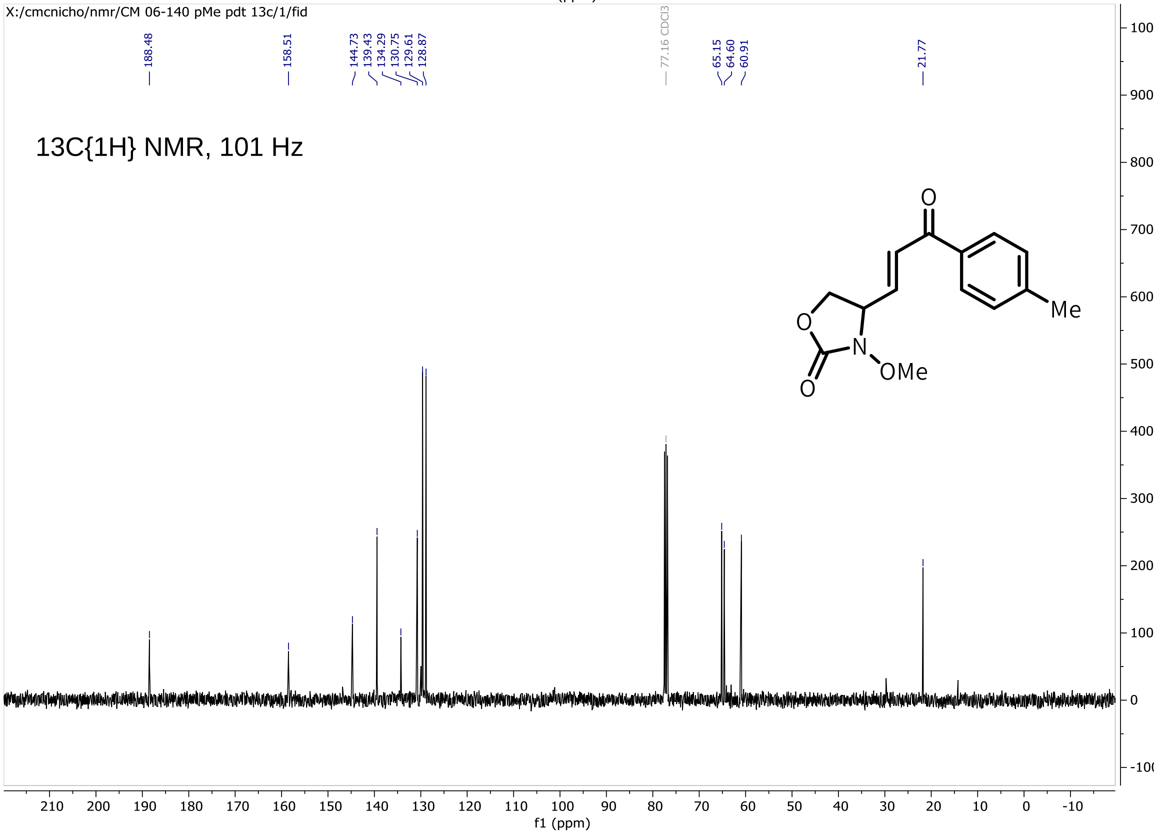

(*E*)-3-methoxy-4-(3-(p-tolyl)acryloyl)oxazolidin-2-one (2d')

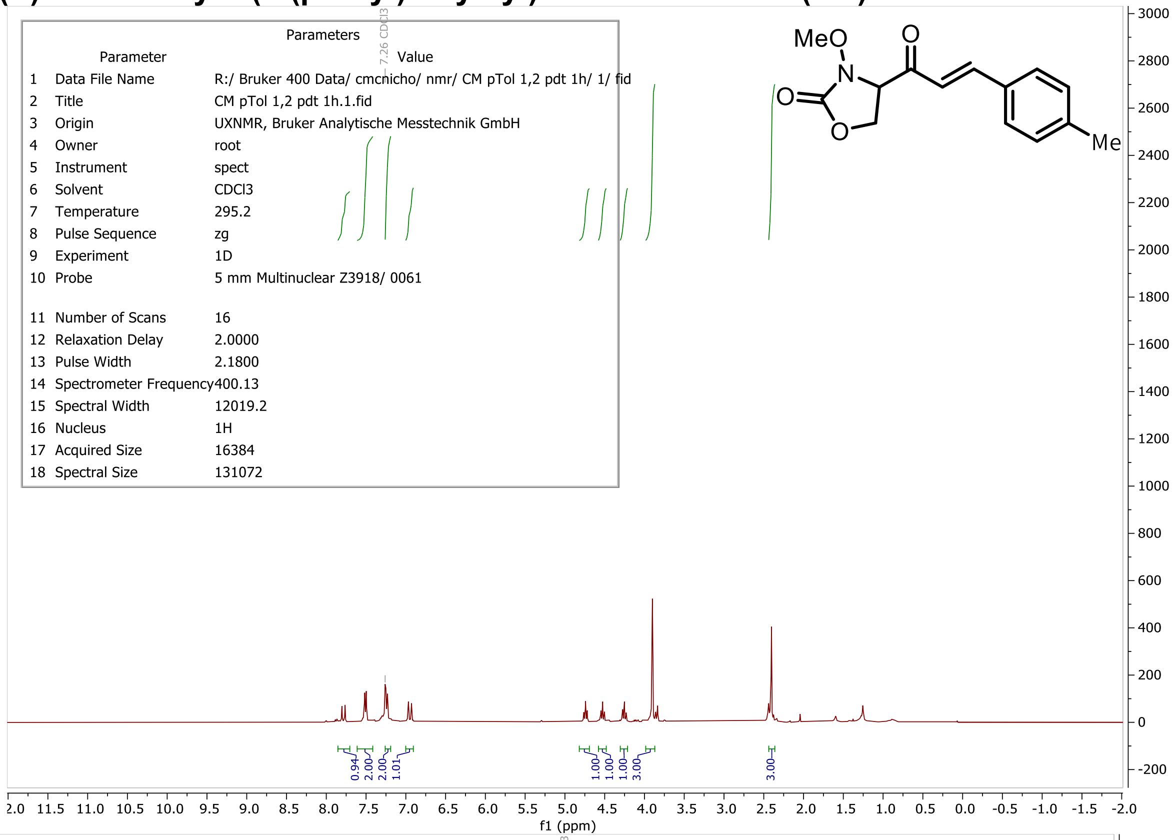

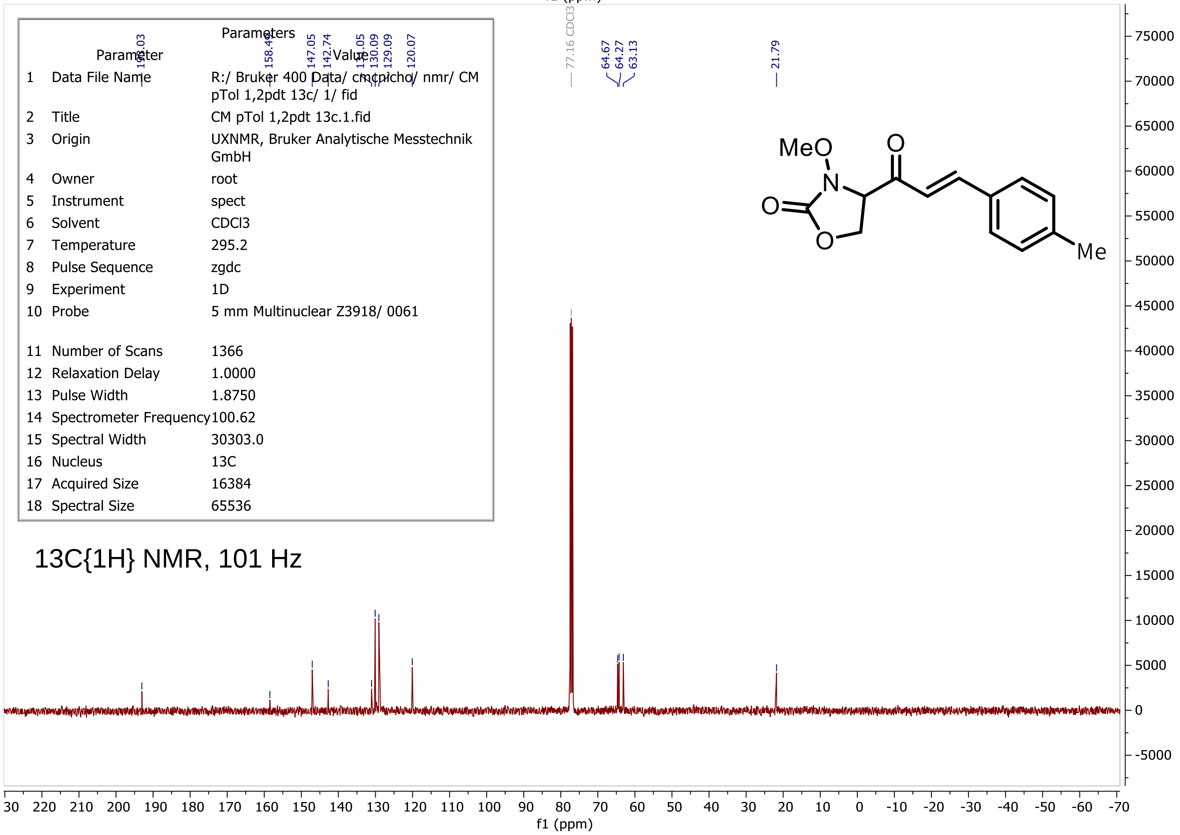

(*E*)-3-methoxy-4-(3-oxo-3-(4-(trifluoromethyl)phenyl)prop-1-en-1-yl)oxazolidin-2-one (2e)

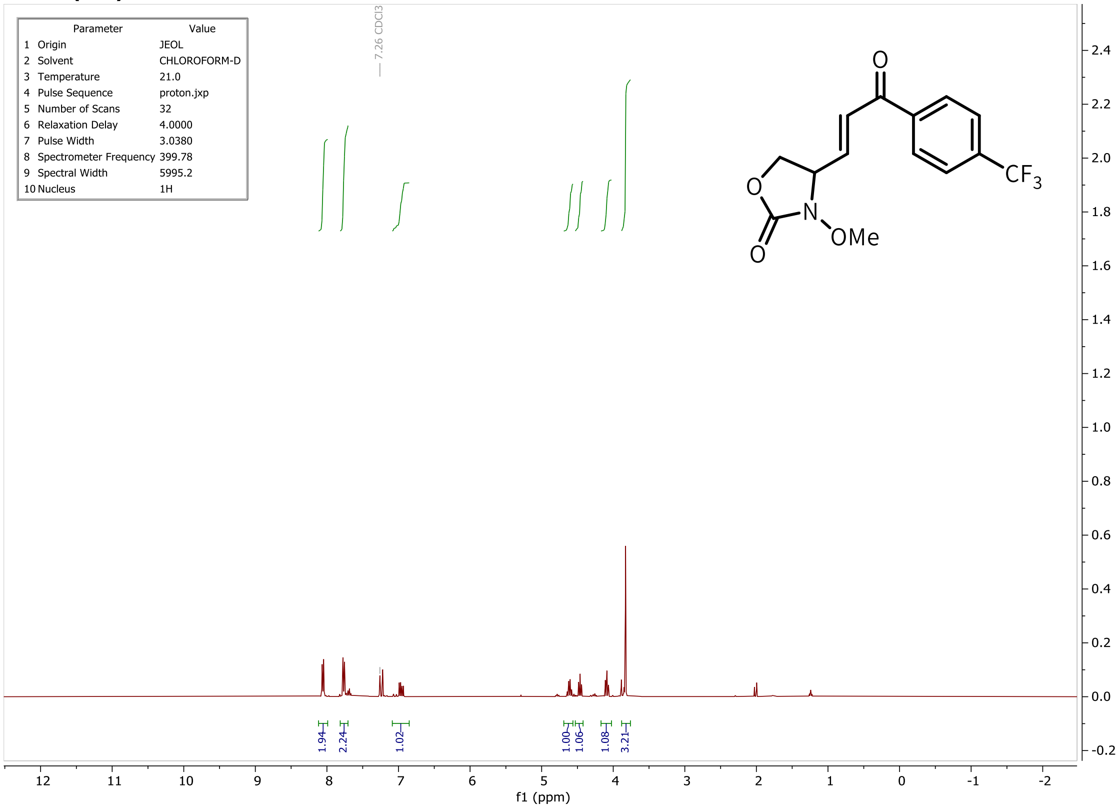

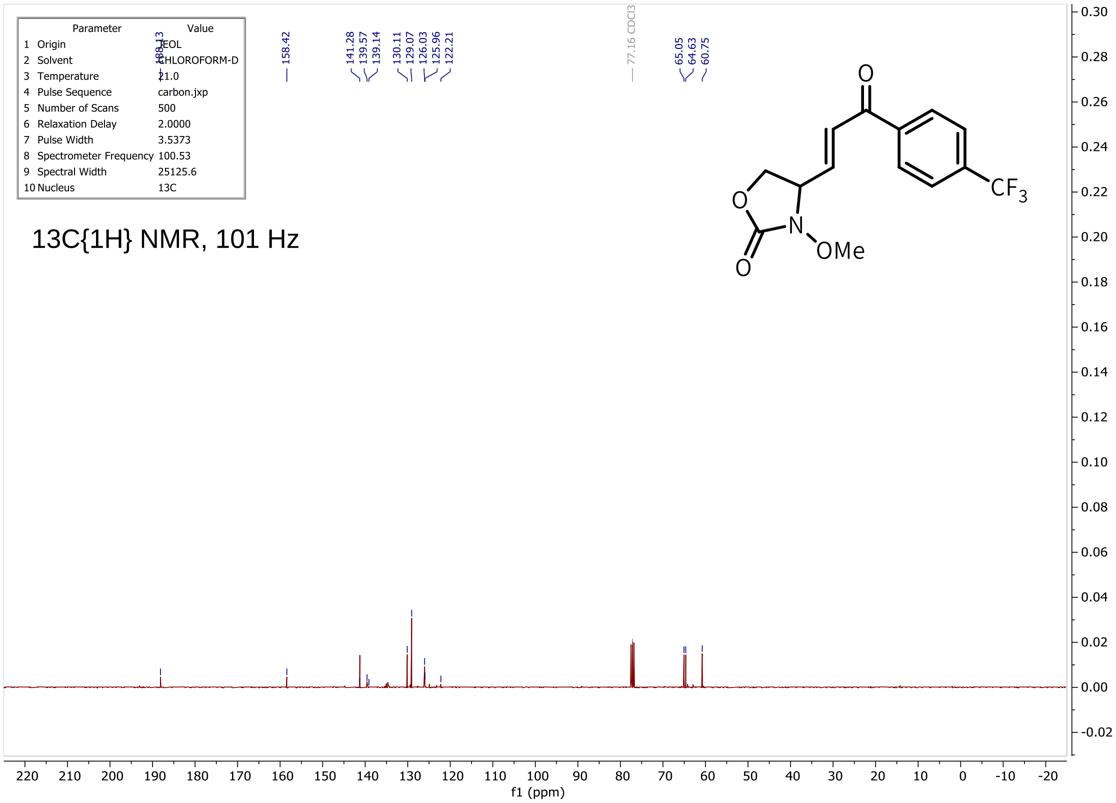

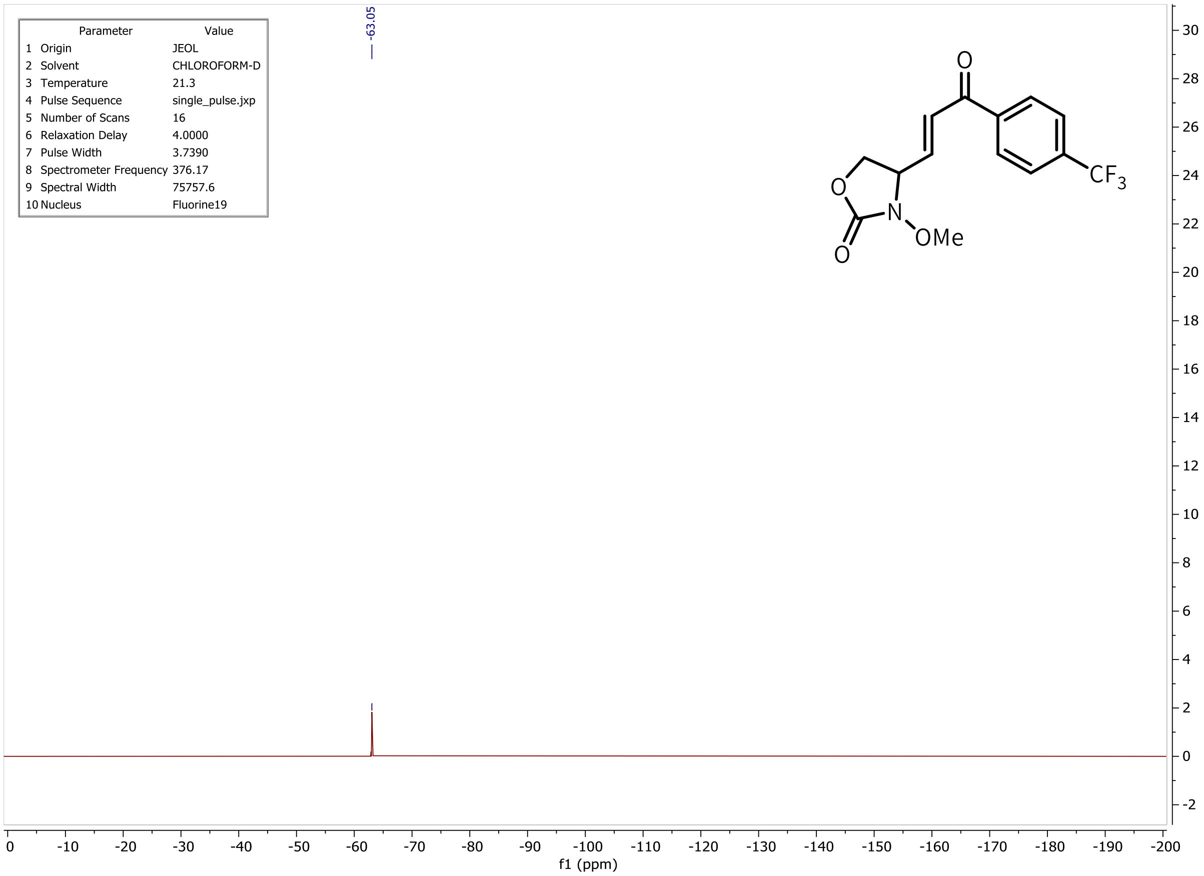

Parameter Value
1 Origin JEOL
2 Solvent CHLOROFORM-D
3 Temperature 21.3
4 Pulse Sequence single_pulse.jxp
5 Number of Scans 16
6 Relaxation Delay 4.0000
7 Pulse Width 3.7390
8 Spectrometer Frequency 376.17
9 Spectral Width 75757.6
10 Nucleus Fluorine19
-63.05
O
O
N
OMe
O
CF3
f1 (ppm)

(*E*)-3-methoxy-4-(3-(4-(trifluoromethyl)phenyl)acryloyl)oxazolidin-2-one (2e’)

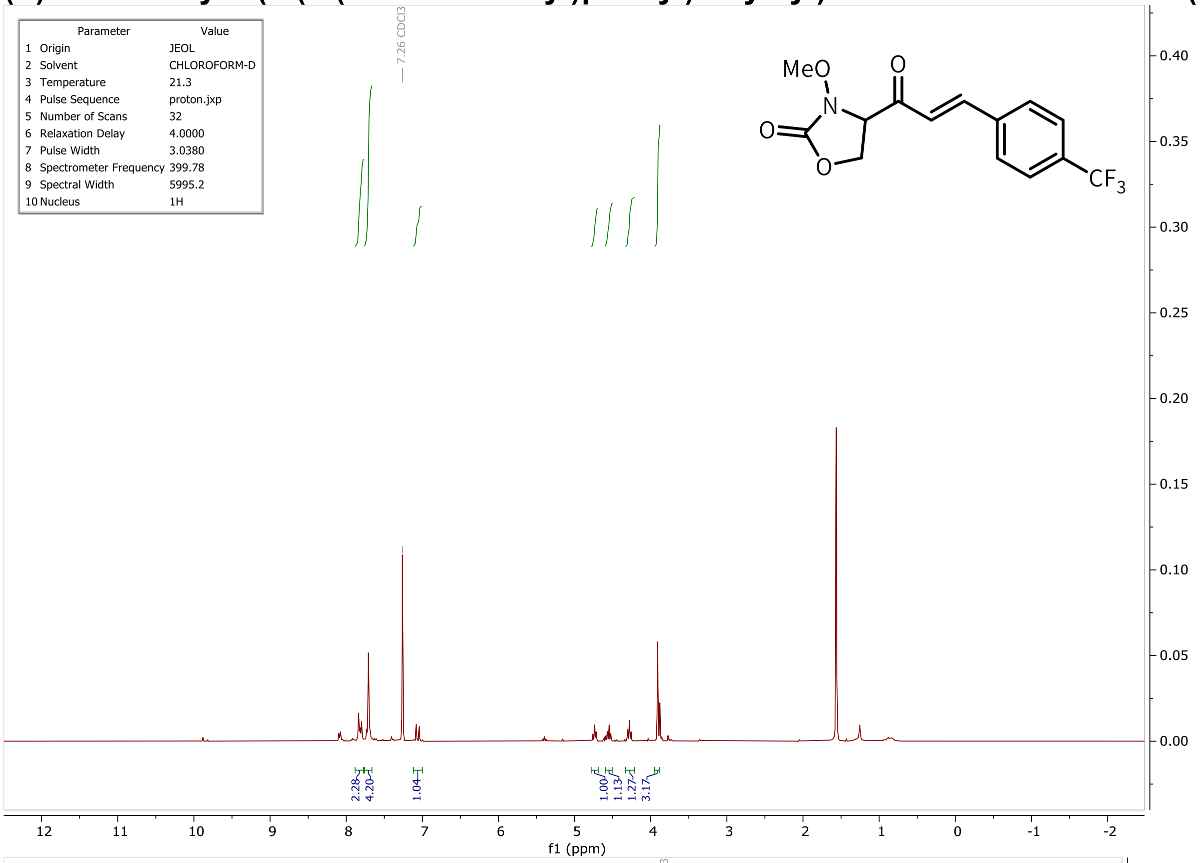

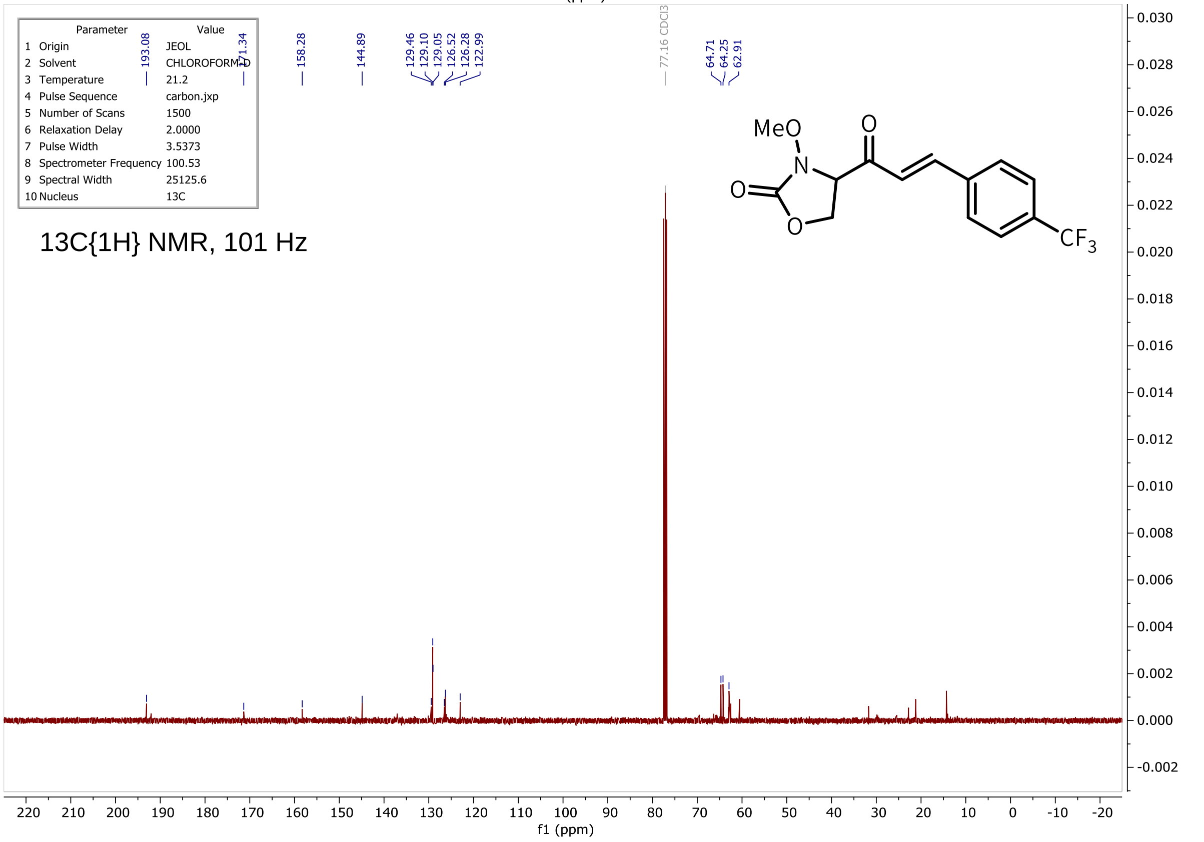

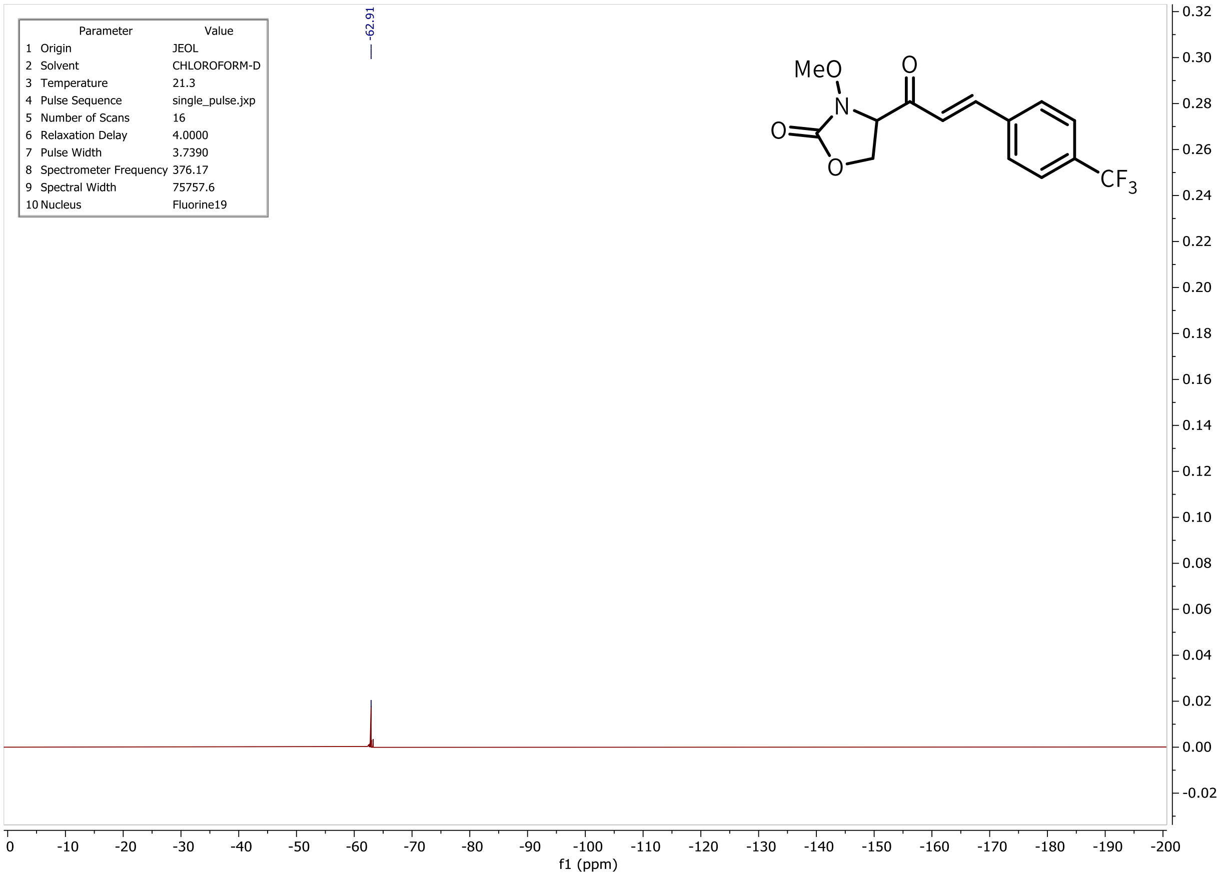

Parameter Value
1 Origin JEOL
2 Solvent CHLOROFORM-D
3 Temperature 21.3
4 Pulse Sequence single_pulse.jxp
5 Number of Scans 16
6 Relaxation Delay 4.0000
7 Pulse Width 3.7390
8 Spectrometer Frequency 376.17
9 Spectral Width 75757.6
10 Nucleus Fluorine19
-62.91
MeO
N
O
O
O
CF3
f1 (ppm)

(*E*)-3-methoxy-4-(3-oxo-3-(o-tolyl)prop-1-en-1-yl)oxazolidin-2-one (2f)

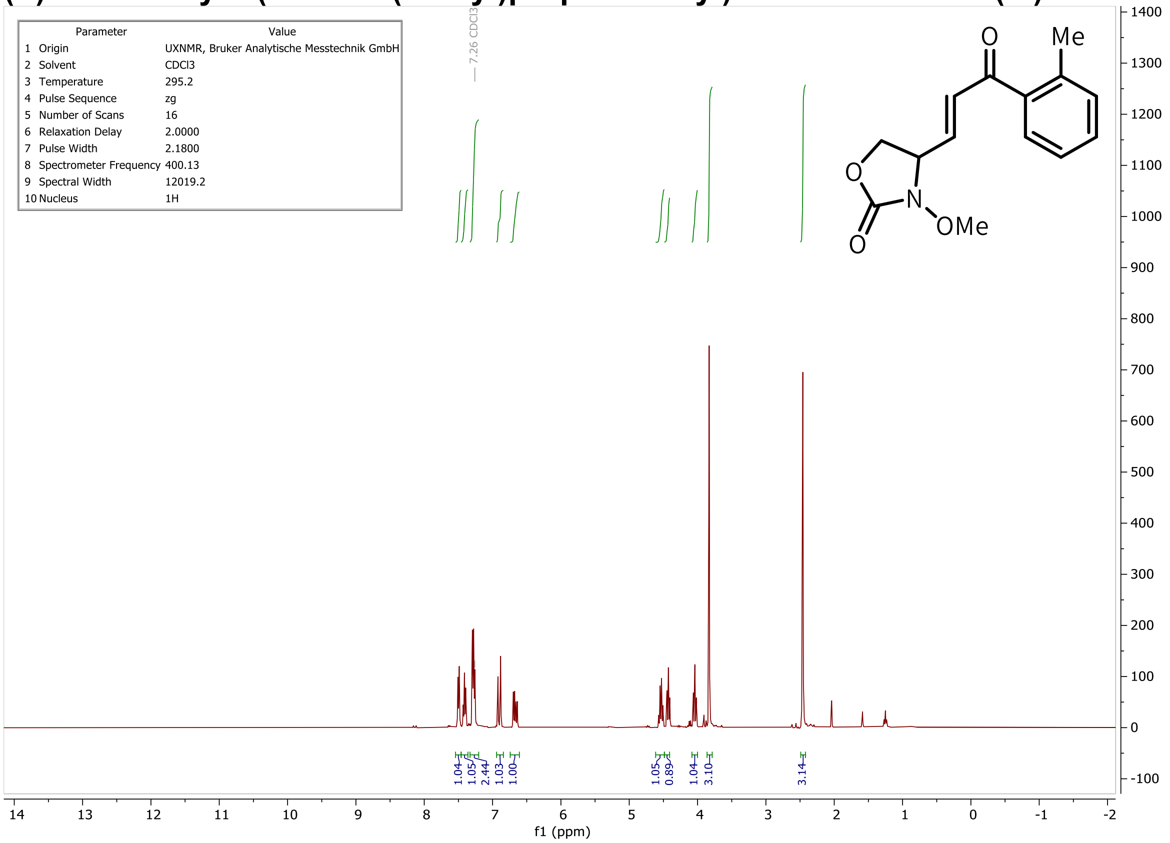

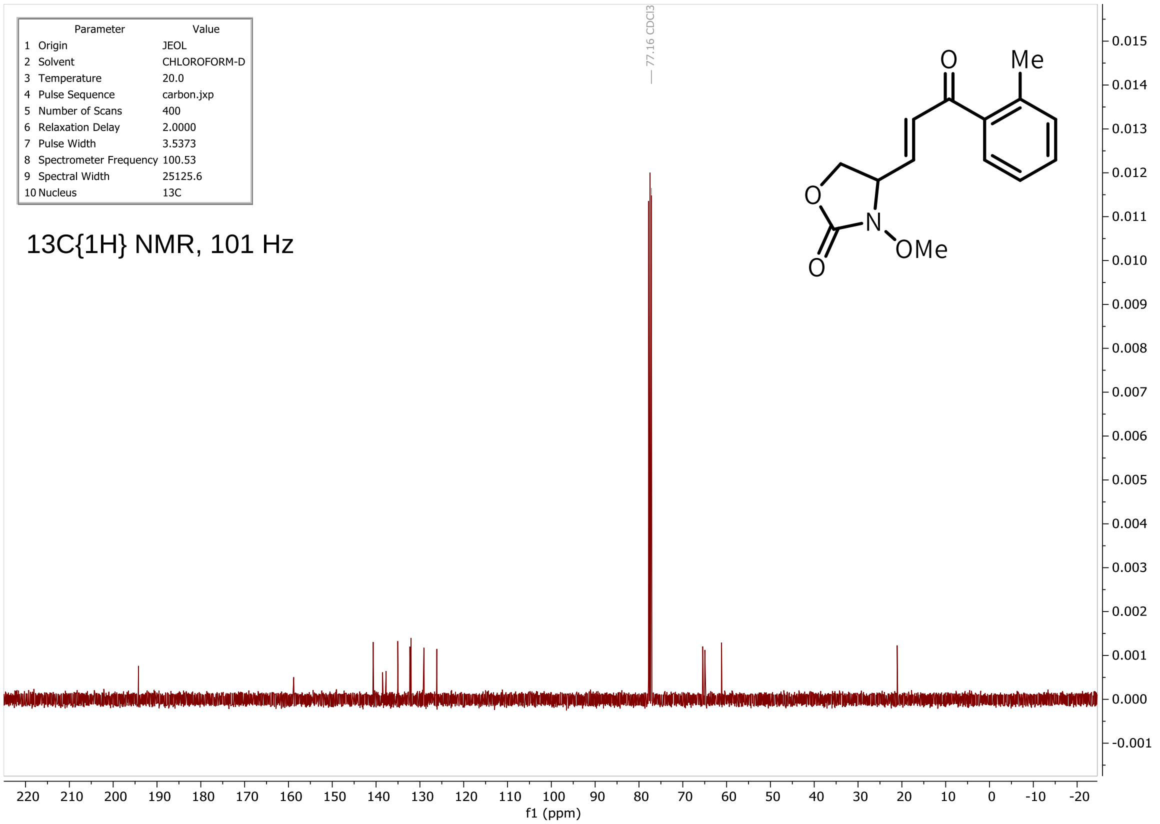

(*E*)-3-methoxy-4-(3-(o-tolyl)acryloyl)oxazolidin-2-one (2f')

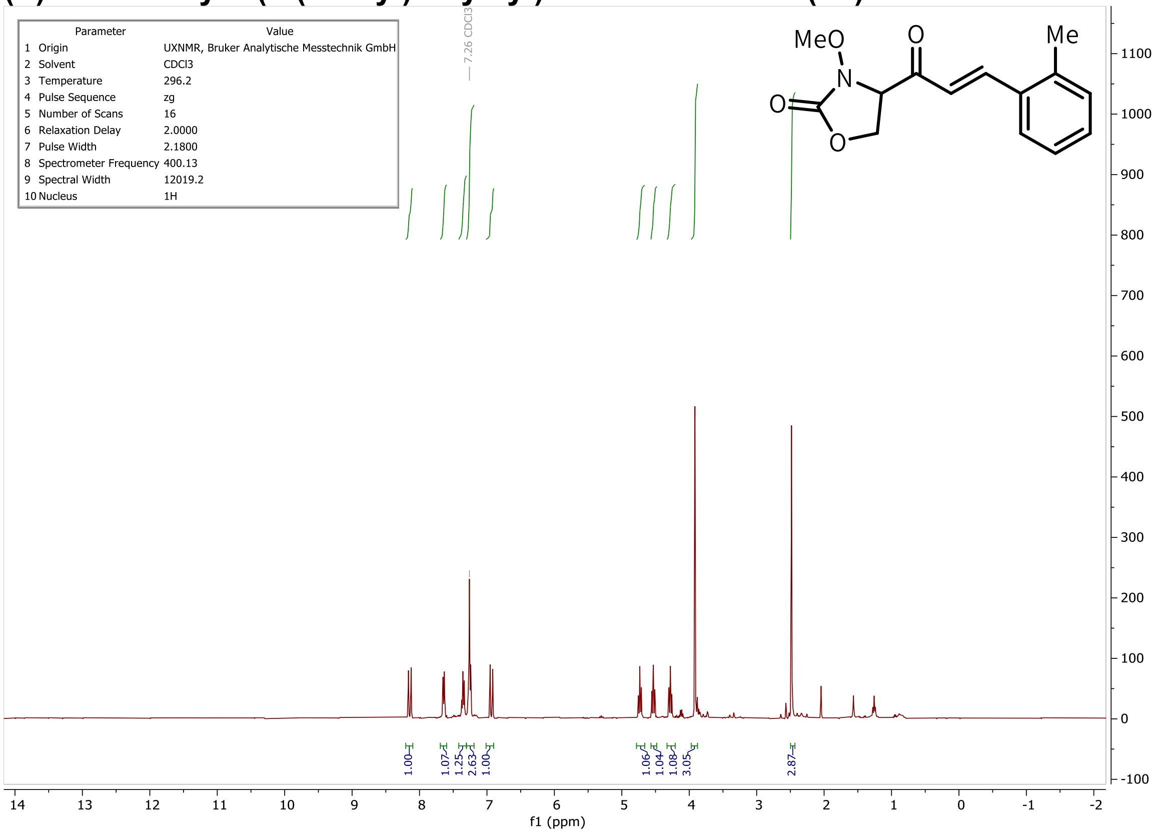

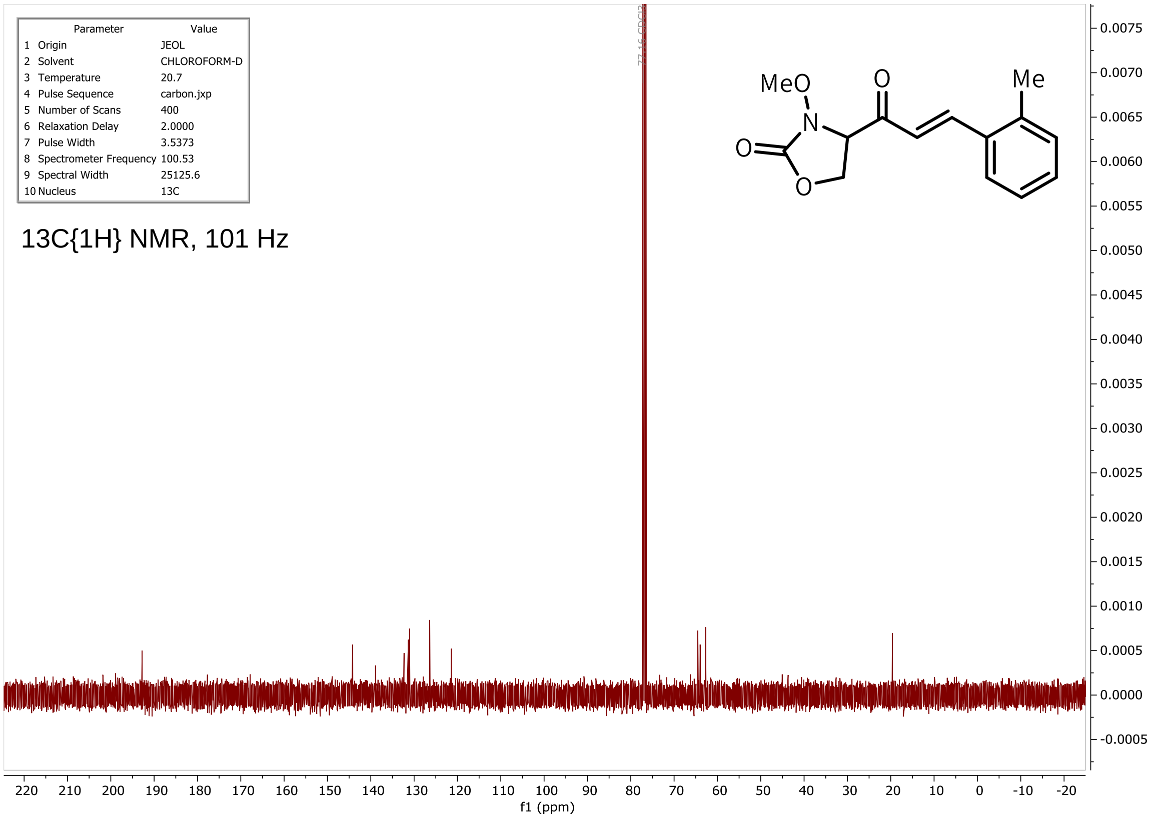

(*E*)-3-methoxy-4-(2-methyl-3-oxo-3-phenylprop-1-en-1-yl)oxazolidin-2-one (2g)

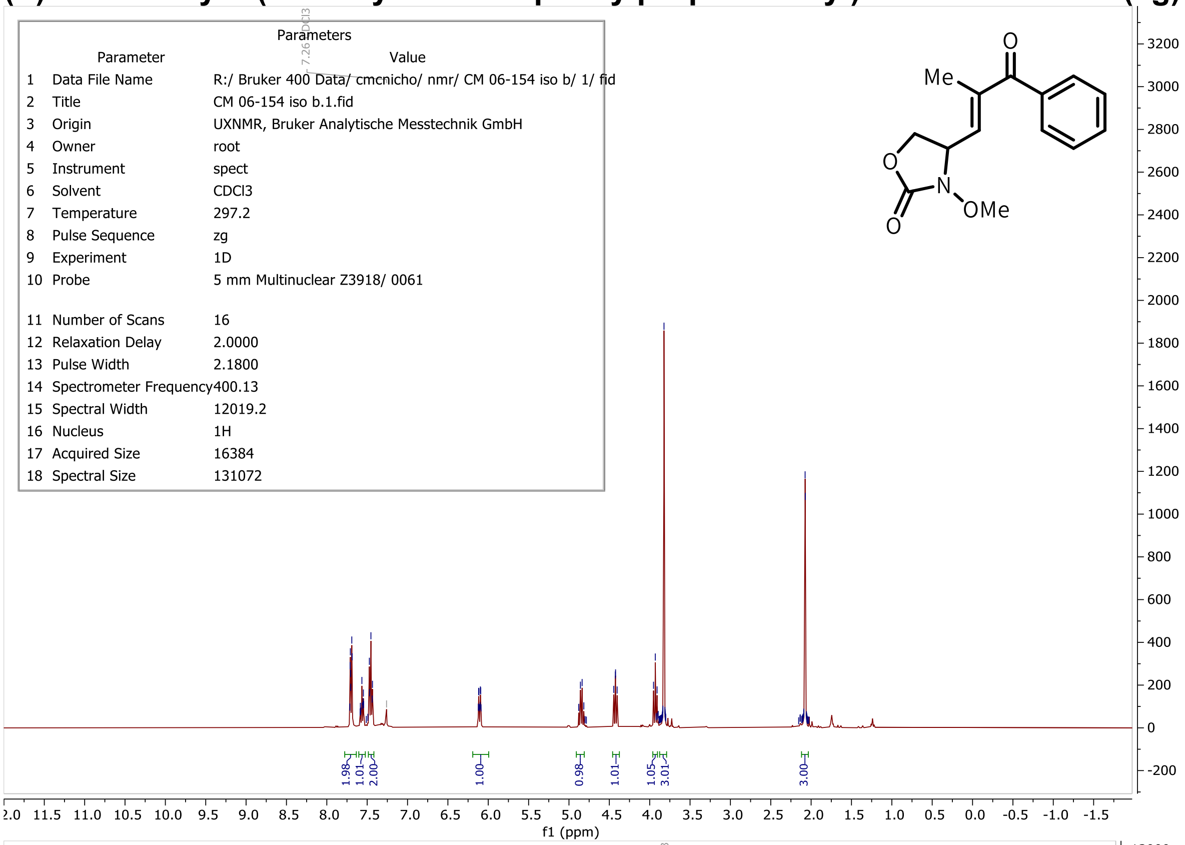

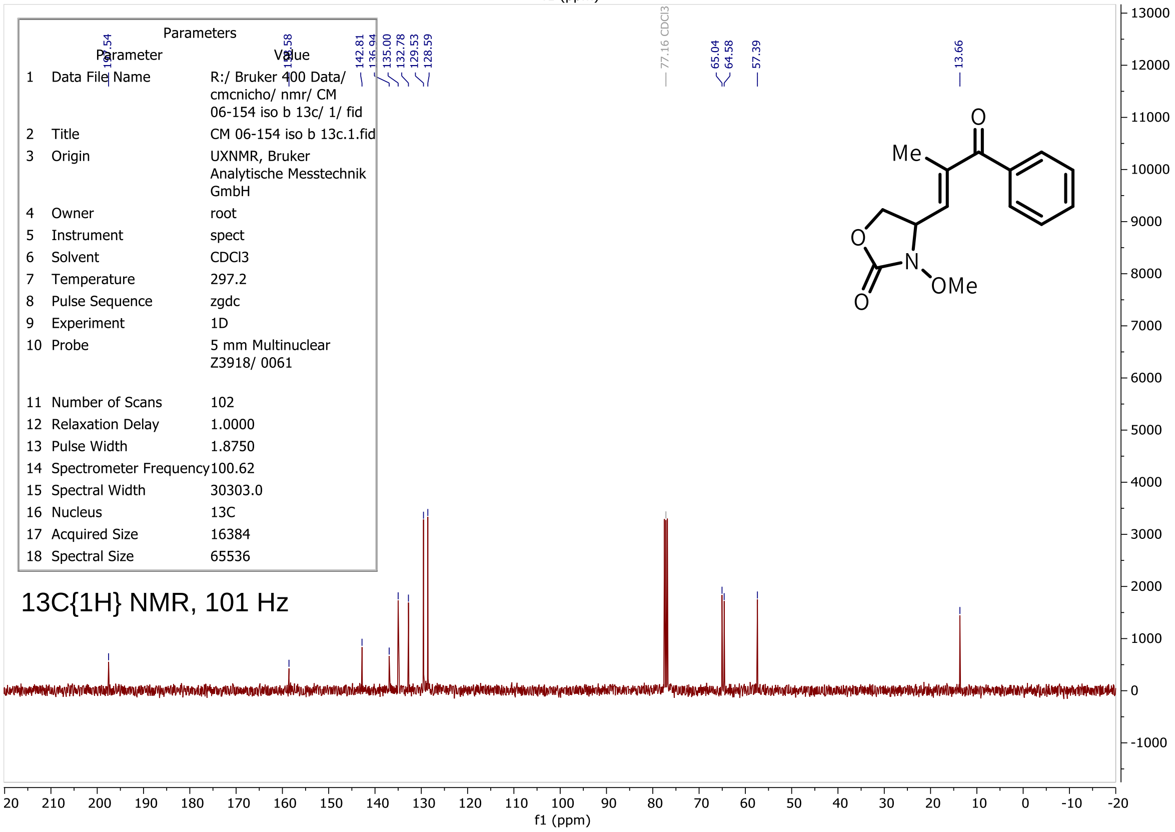

(*Z*)-4-(2-bromo-3-oxo-3-phenylprop-1-en-1-yl)-3-methoxyoxazolidin-2-one (2h)

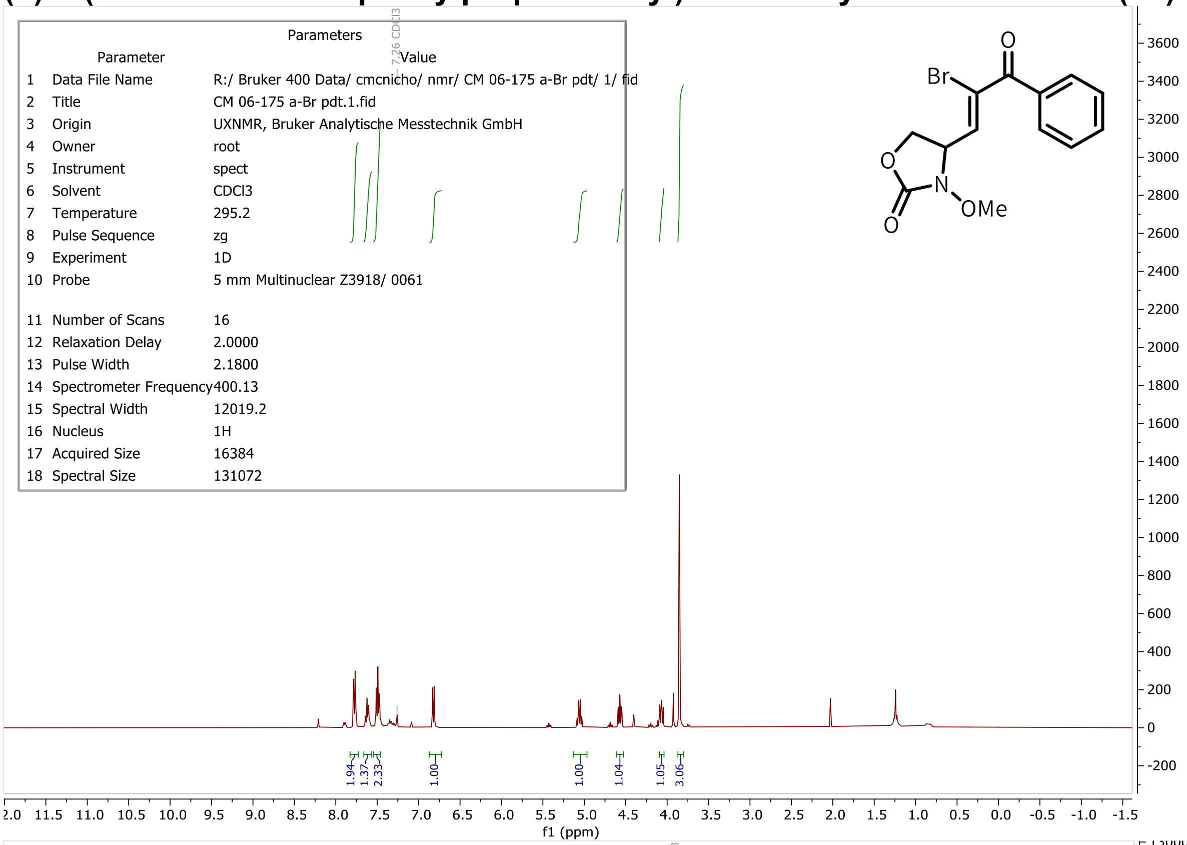

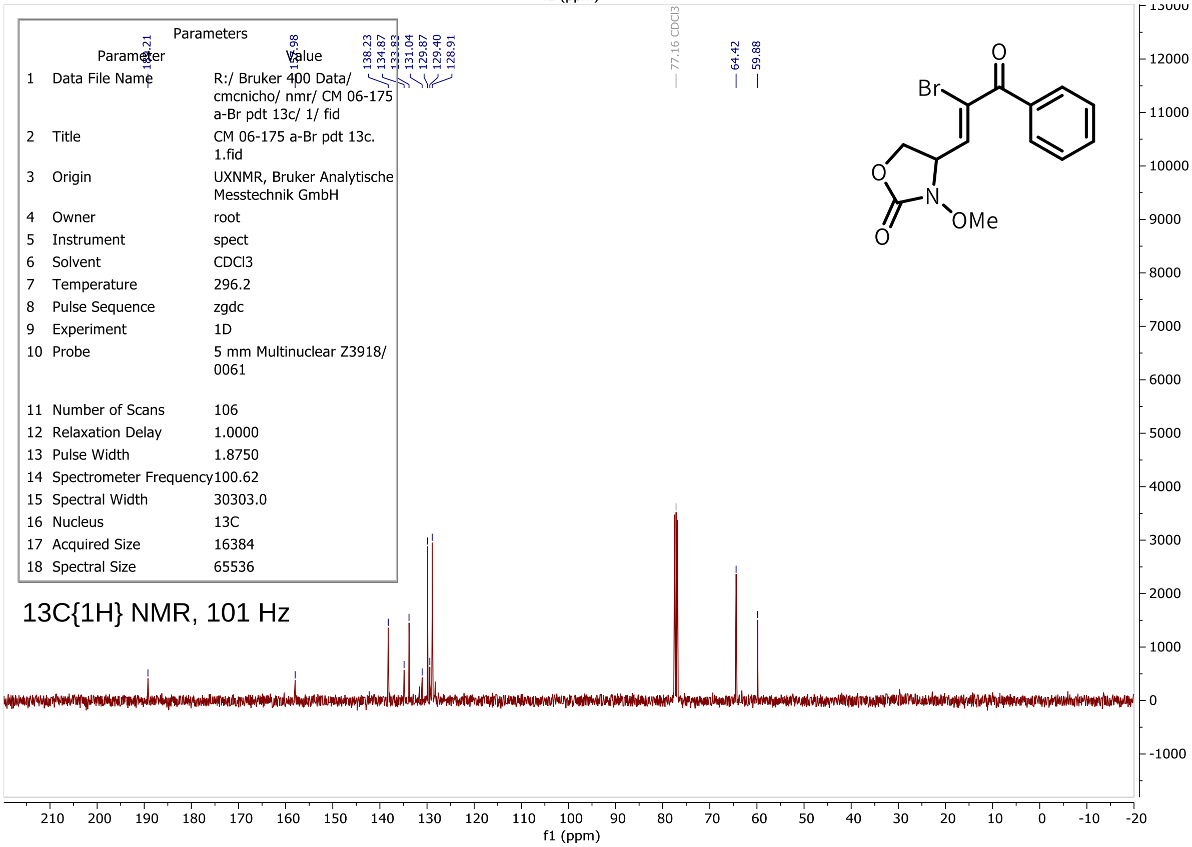

(*E*)-3-methoxy-4-(3-oxobut-1-en-1-yl)oxazolidin-2-one (2j)

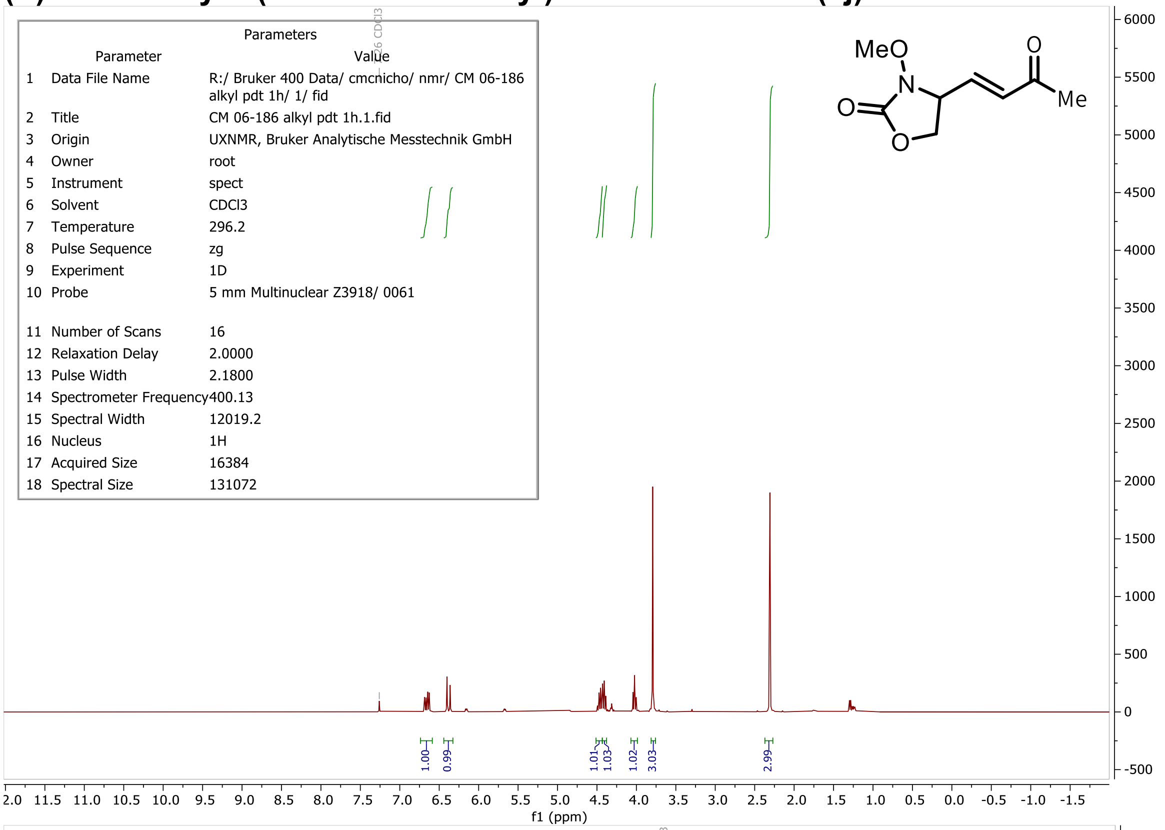

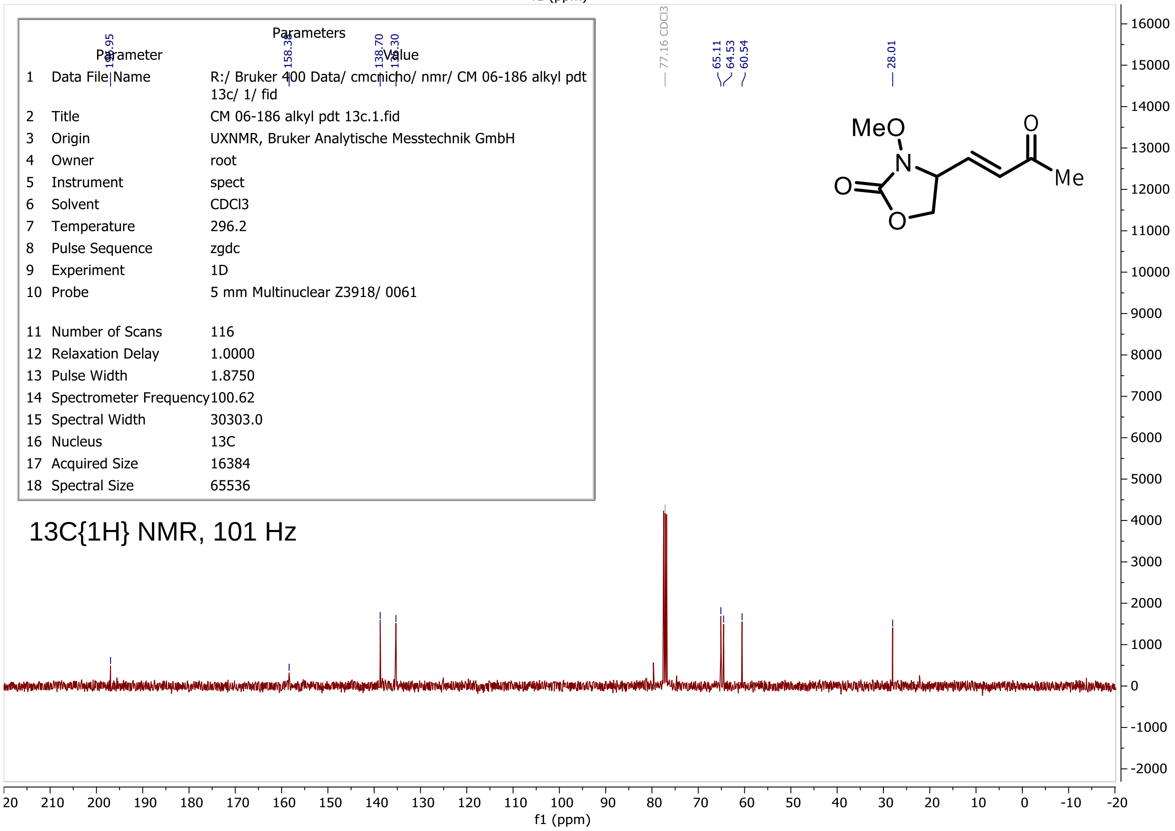

(*E*)-4-(3-(furan-2-yl)acryloyl)-3-methoxyoxazolidin-2-one (2k')

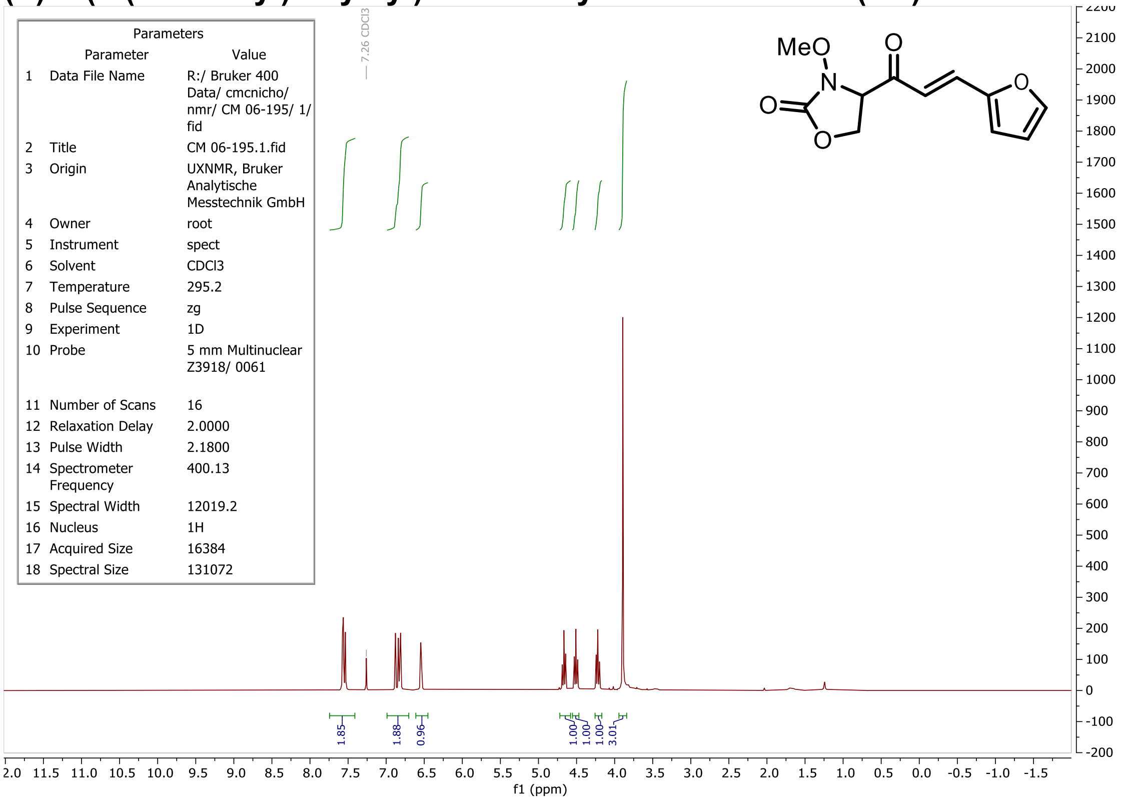

Parameters

	Parameter	Value
1	Data File Name	R:/ Bruker 400 Data/ cmcnicho/ nmr/ CM 06-195/ 1/ fid
2	Title	CM 06-195.1.fid
3	Origin	UXNMR, Bruker Analytische Messtechnik GmbH
4	Owner	root
5	Instrument	spect
6	Solvent	CDCl3
7	Temperature	295.2
8	Pulse Sequence	zg
9	Experiment	1D
10	Probe	5 mm Multinuclear Z3918/ 0061
11	Number of Scans	16
12	Relaxation Delay	2.0000
13	Pulse Width	2.1800
14	Spectrometer Frequency	400.13
15	Spectral Width	12019.2
16	Nucleus	1H
17	Acquired Size	16384
18	Spectral Size	131072

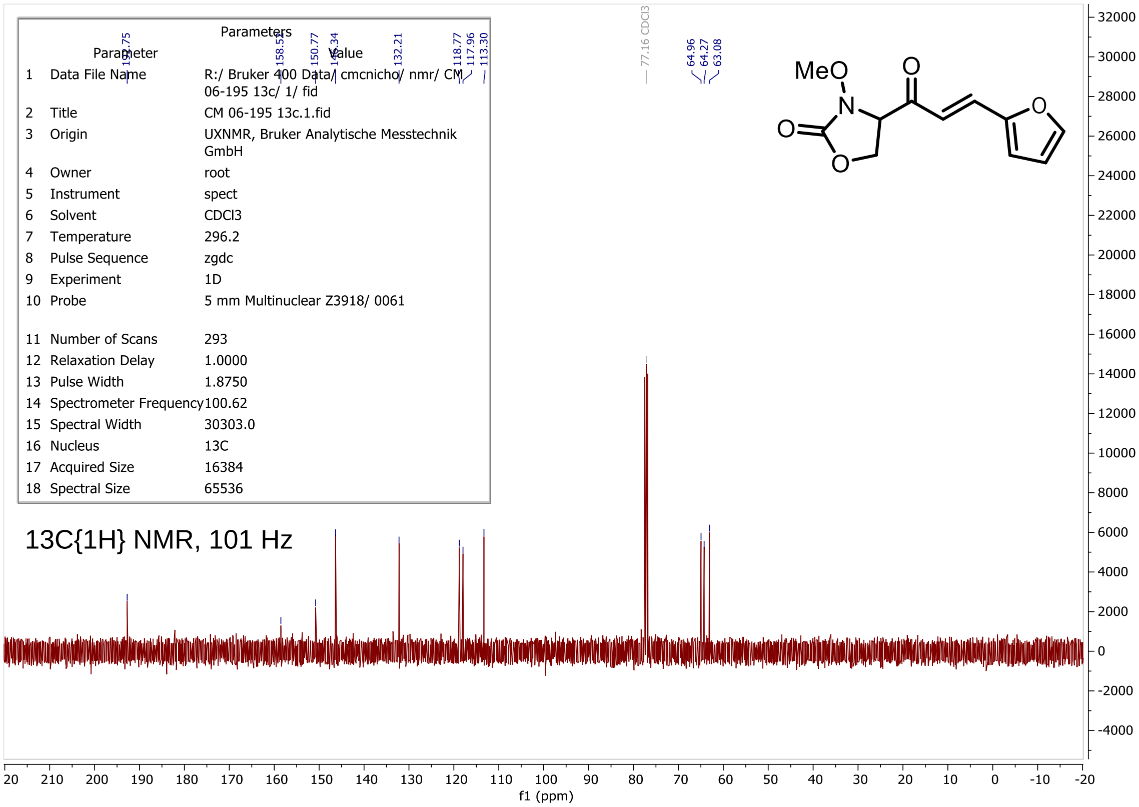

Parameters

	Parameter	Value
1	Data File Name	R:/ Bruker 400 Data/ cmcnicho/ nmr/ CM 06-195 13c/ 1/ fid
2	Title	CM 06-195 13c.1.fid
3	Origin	UXNMR, Bruker Analytische Messtechnik GmbH
4	Owner	root
5	Instrument	spect
6	Solvent	CDCl3
7	Temperature	296.2
8	Pulse Sequence	zgdc
9	Experiment	1D
10	Probe	5 mm Multinuclear Z3918/ 0061
11	Number of Scans	293
12	Relaxation Delay	1.0000
13	Pulse Width	1.8750
14	Spectrometer Frequency	100.62
15	Spectral Width	30303.0
16	Nucleus	13C
17	Acquired Size	16384
18	Spectral Size	65536

(*E*)-3-methoxy-4-(3-oxo-3-(pyridin-2-yl)prop-1-en-1-yl)oxazolidin-2-one (2l)

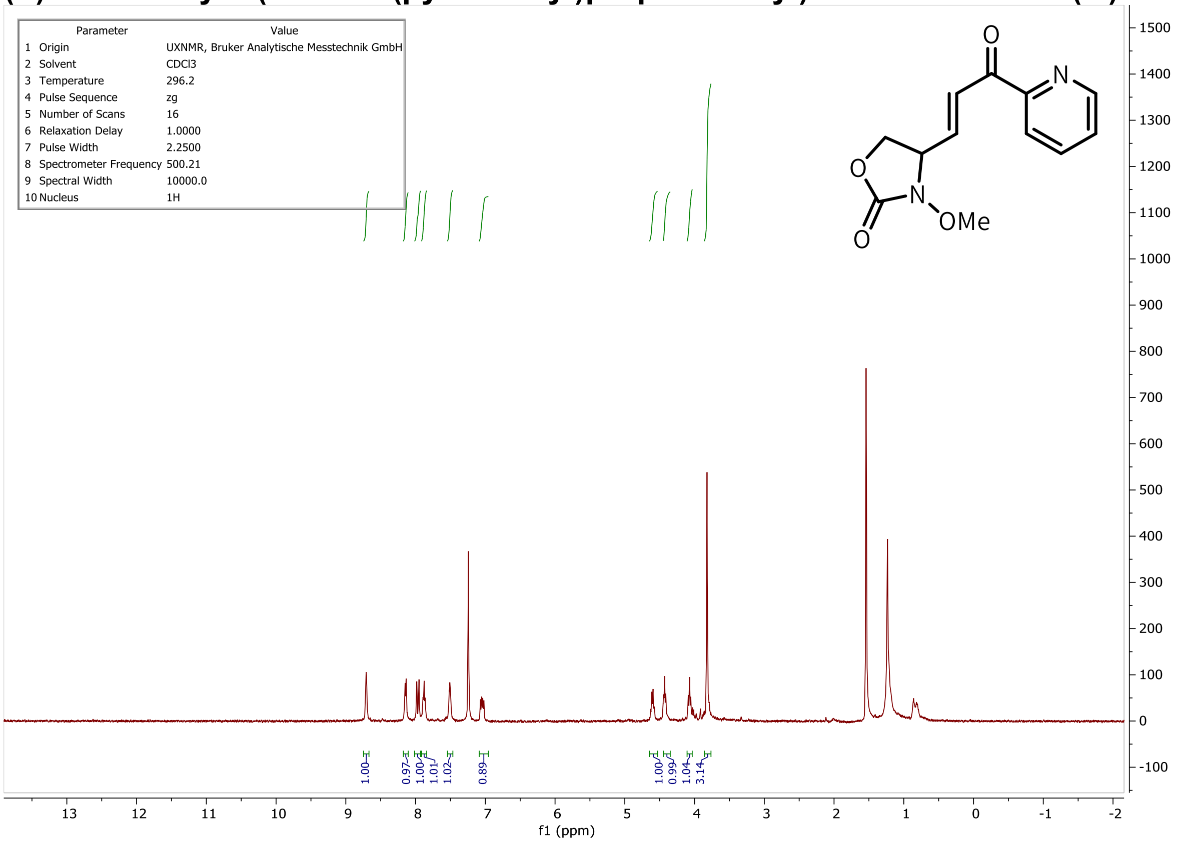

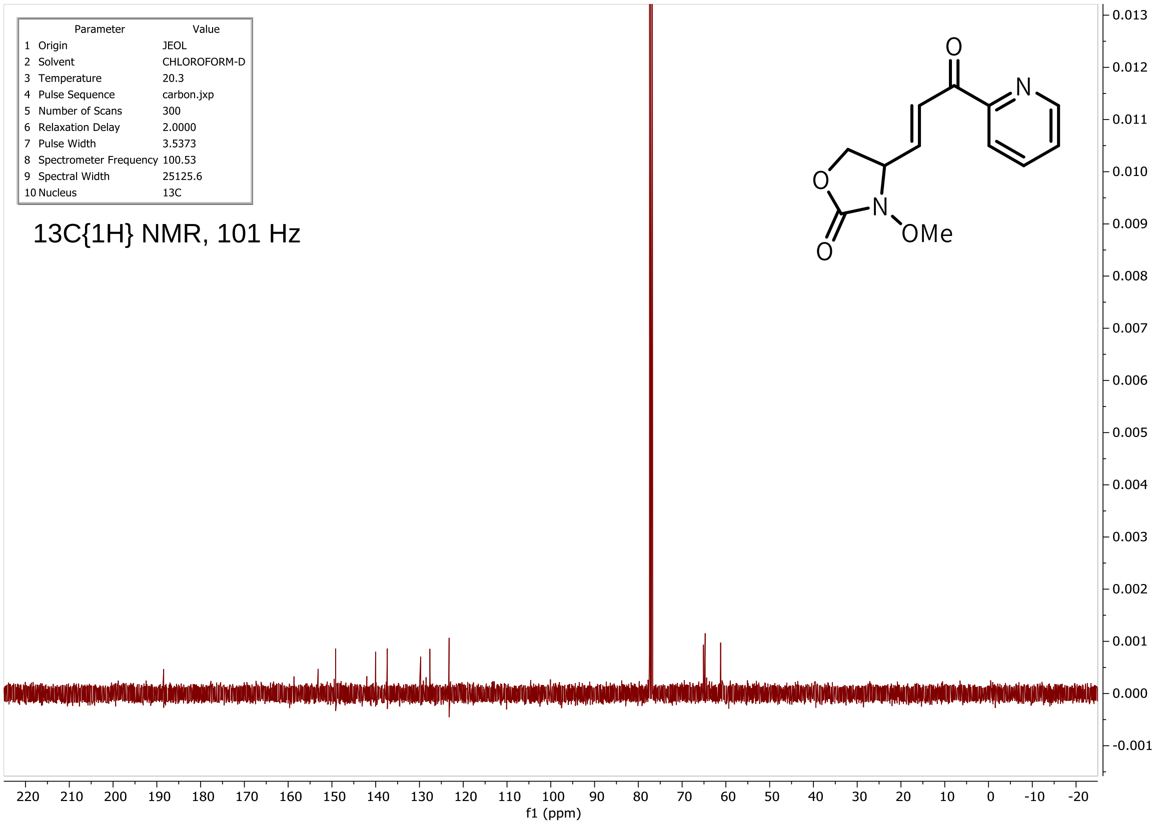

(*E*)-3-methoxy-4-(3-(pyridin-2-yl)acryloyl)oxazolidin-2-one (2l’)

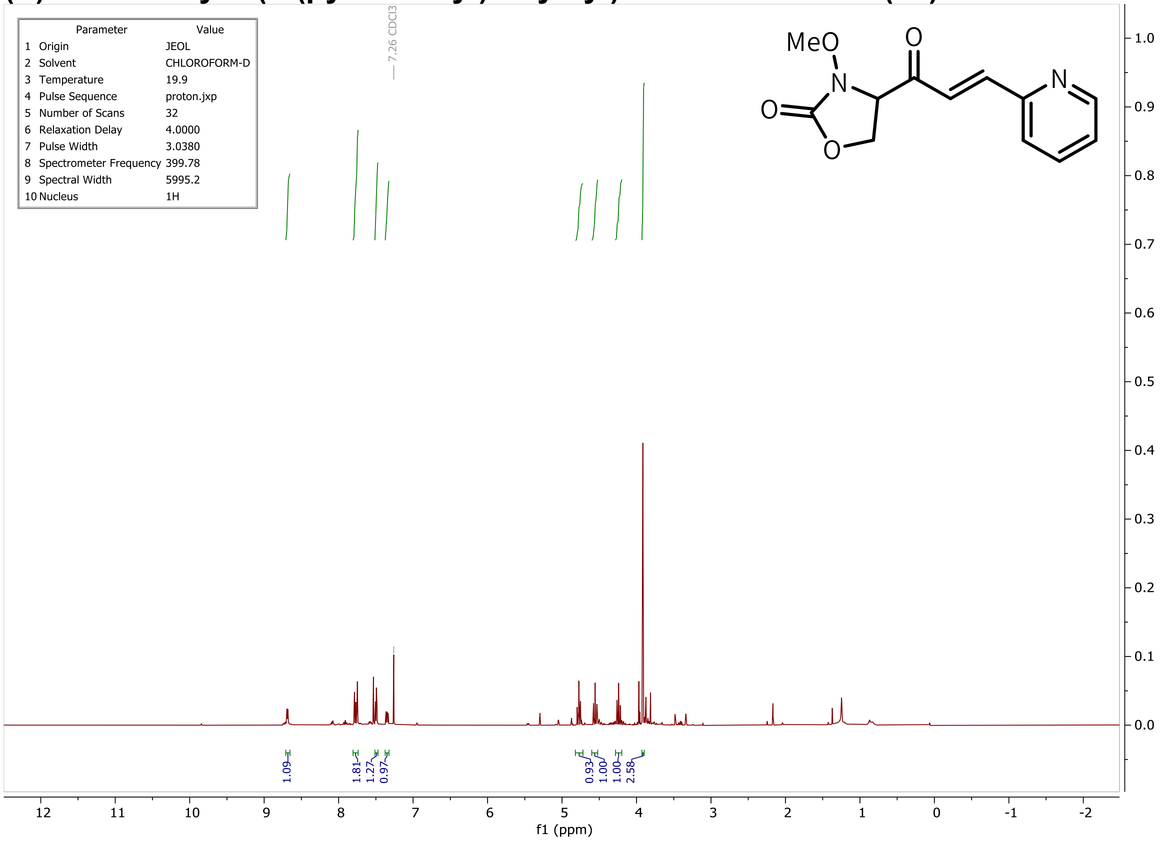

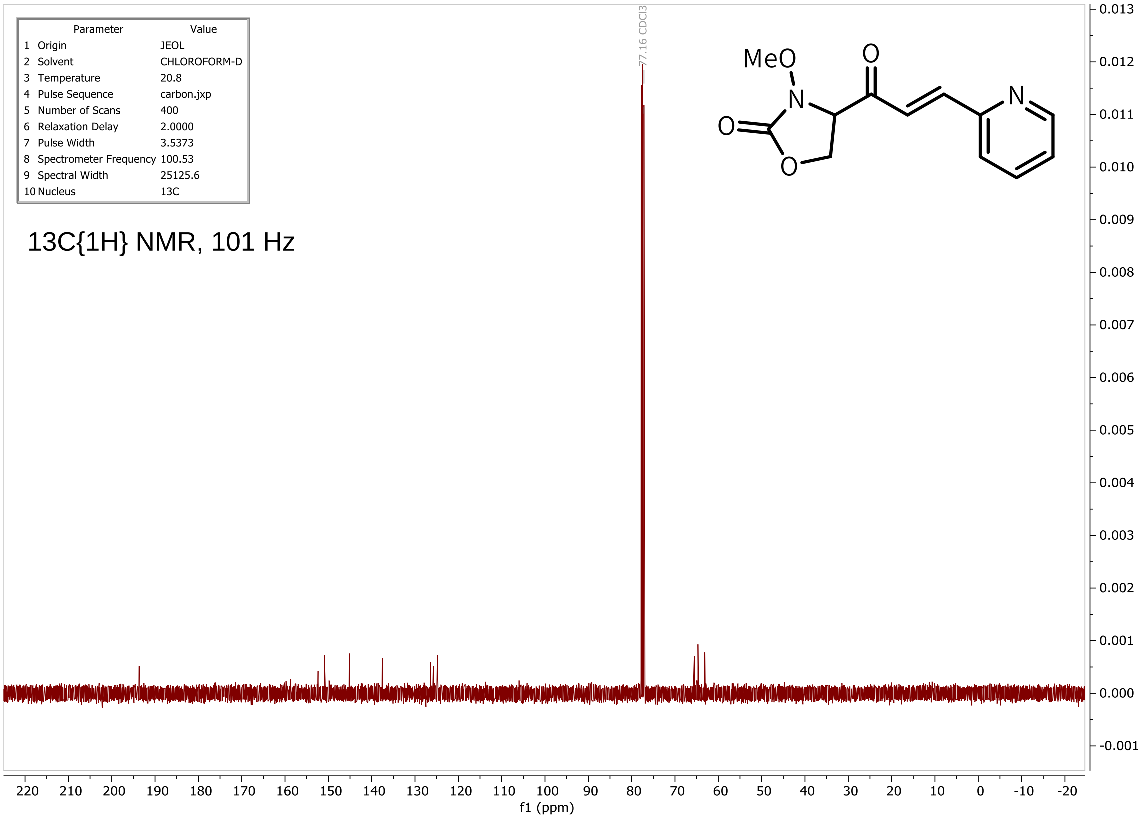

(*E*)-3-methoxy-4-(3-oxo-3-(3-(trifluoromethyl)phenyl)prop-1-en-1-yl)oxazolidin-2-one (2m)

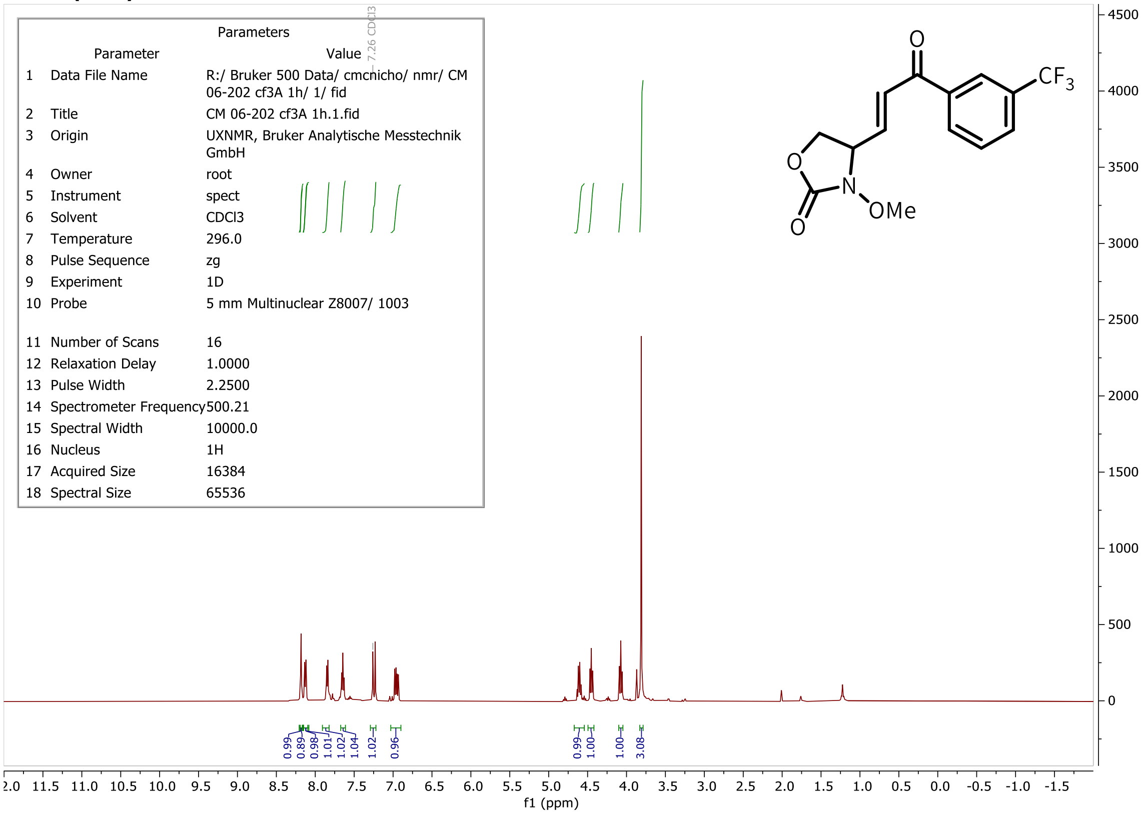

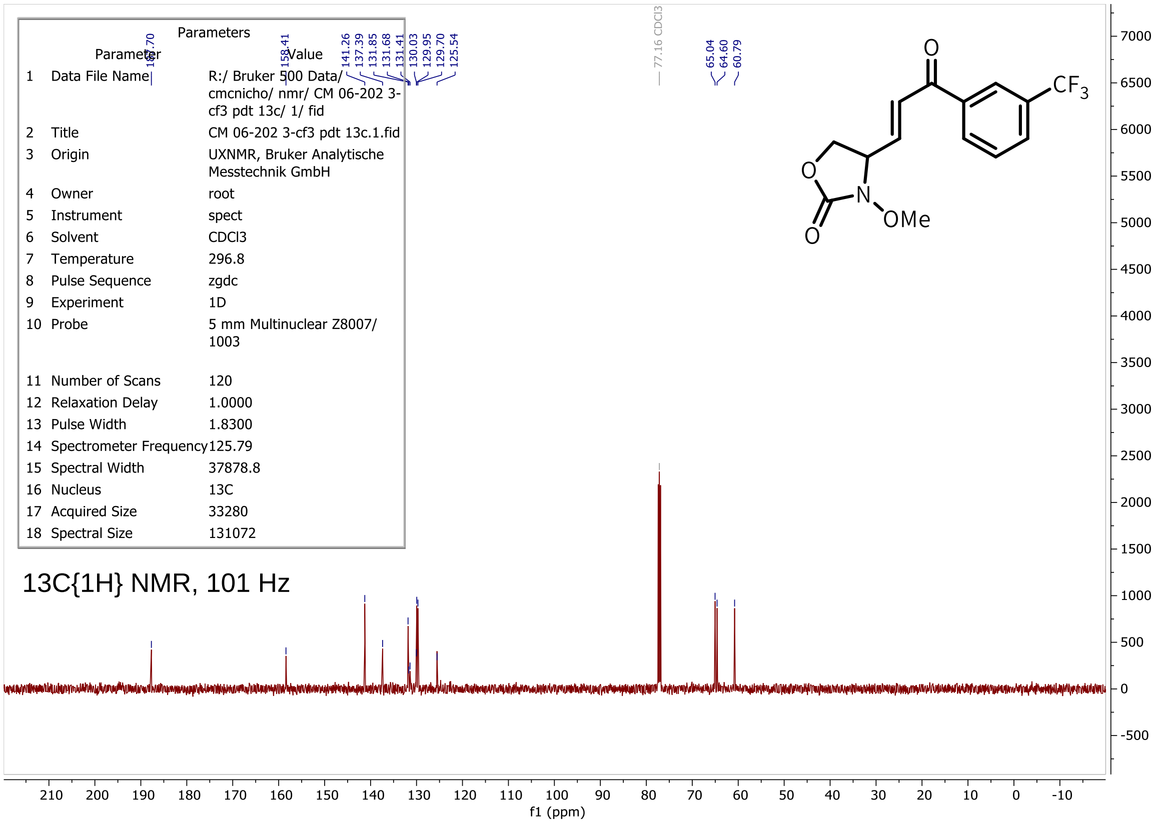

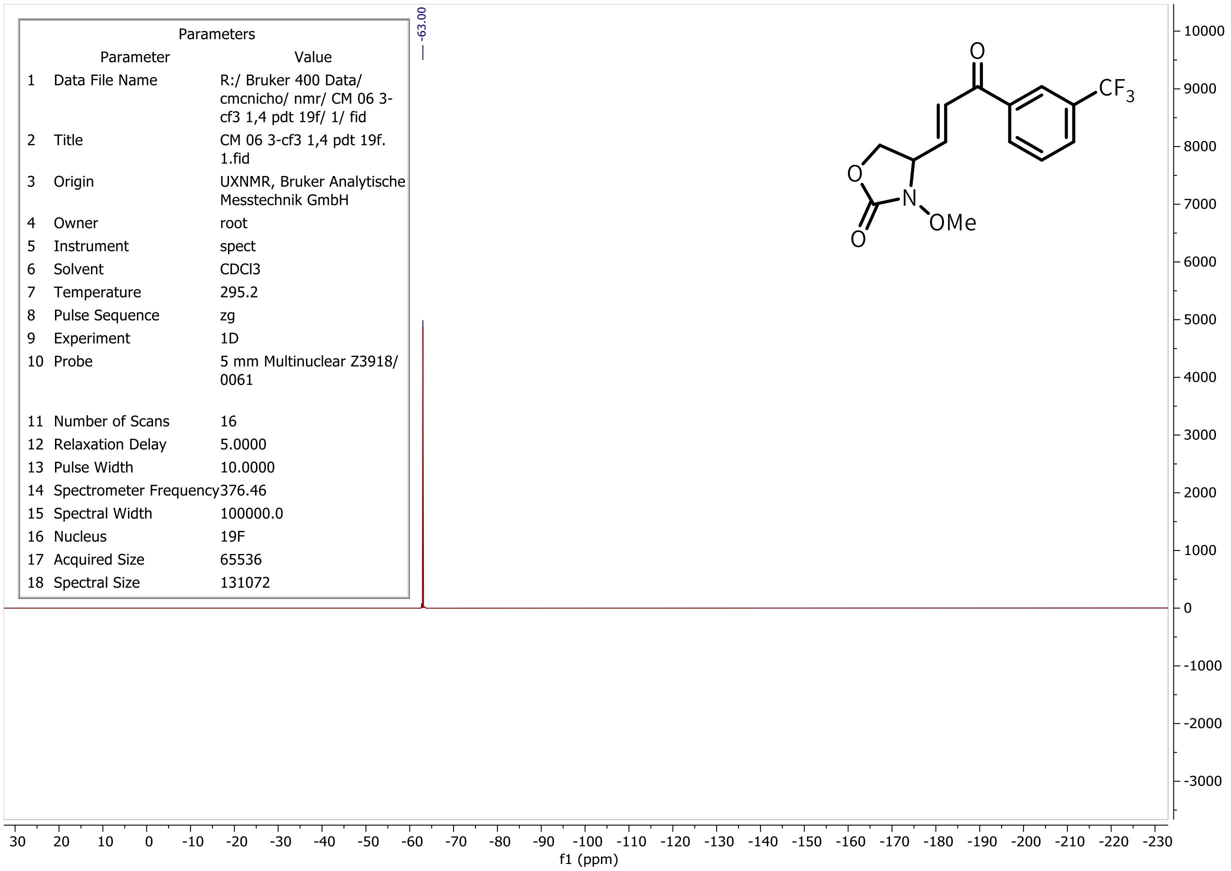
Parameters
Parameter Value
1 Data File Name R:/ Bruker 400 Data/ cmcnicho/ nmr/ CM 06 3-cf3 1,4 pdt 19f/ 1/ fid
2 Title CM 06 3-cf3 1,4 pdt 19f. 1.fid
3 Origin UXNMR, Bruker Analytische Messtechnik GmbH
4 Owner root
5 Instrument spect
6 Solvent CDCl3
7 Temperature 295.2
8 Pulse Sequence zg
9 Experiment 1D
10 Probe 5 mm Multinuclear Z3918/ 0061
11 Number of Scans 16
12 Relaxation Delay 5.0000
13 Pulse Width 10.0000
14 Spectrometer Frequency 376.46
15 Spectral Width 100000.0
16 Nucleus 19F
17 Acquired Size 65536
18 Spectral Size 131072
-63.00
O
CF3
O
N
OMe
O
f1 (ppm)

(*E*)-3-methoxy-4-(3-(3-(trifluoromethyl)phenyl)acryloyl)oxazolidin-2-one (2m’)

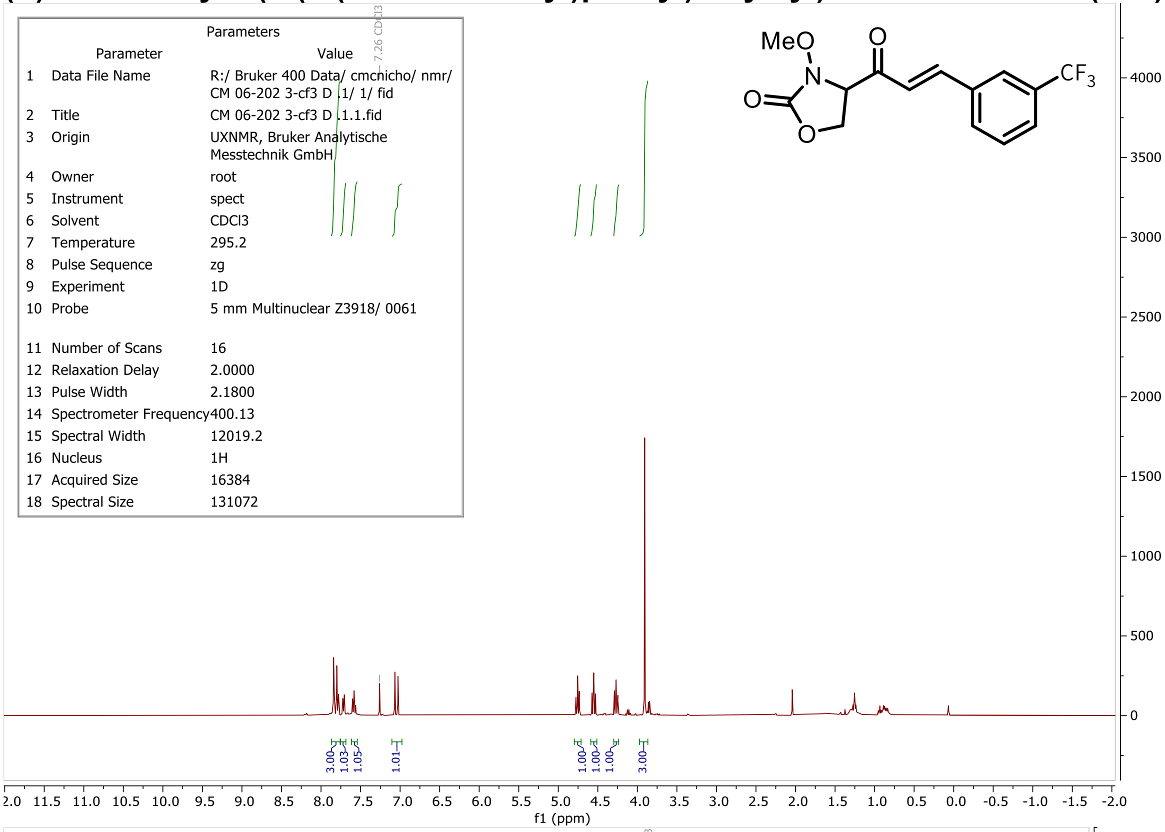

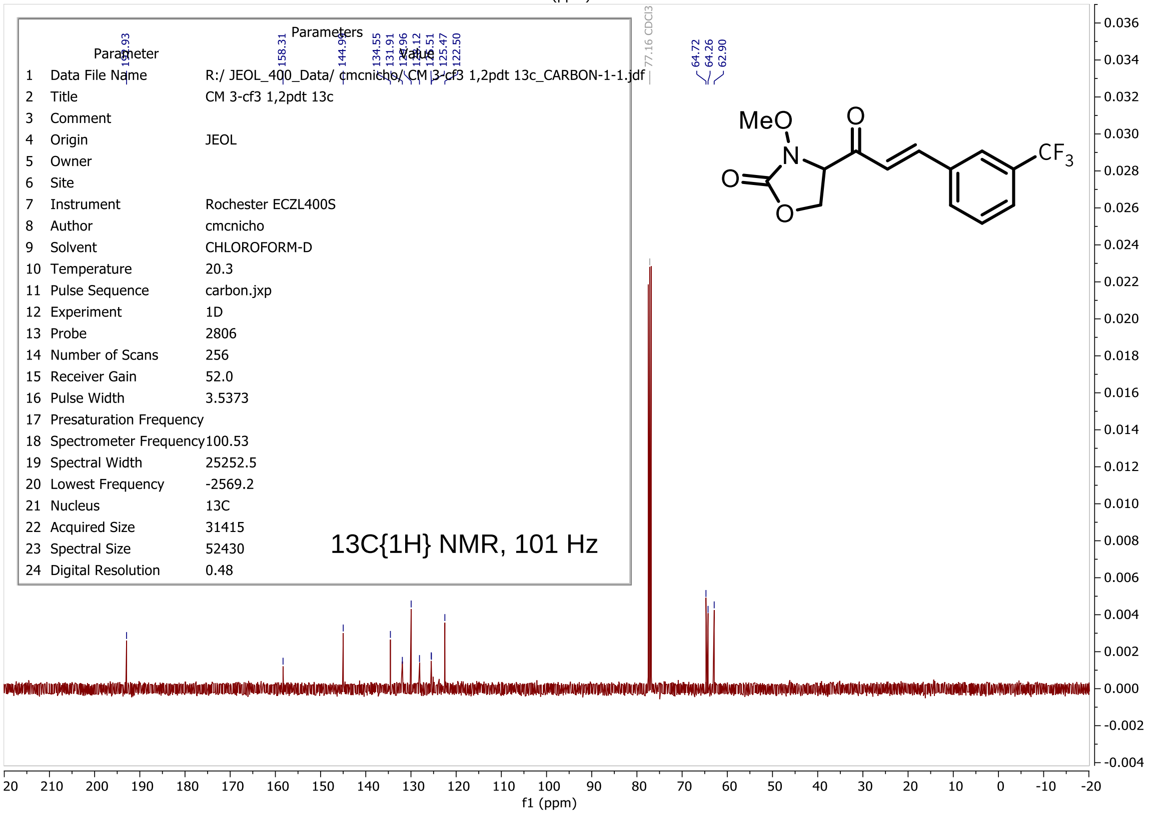

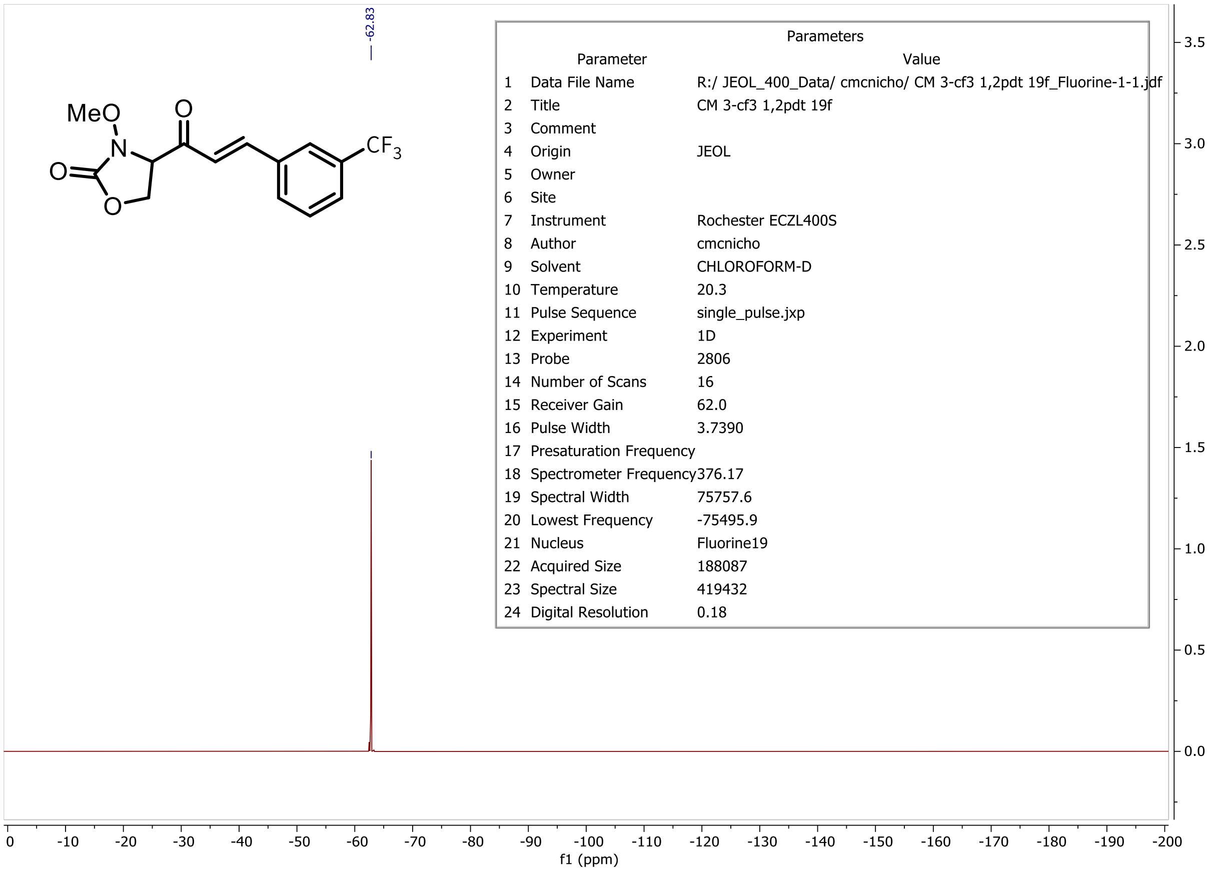

Parameters

	Parameter	Value
1	Data File Name	R:/ JEOL_400_Data/ cmcnicho/ CM 3-cf3 1,2pdt 19f_Fluorine-1-1.jdf
2	Title	CM 3-cf3 1,2pdt 19f
3	Comment	
4	Origin	JEOL
5	Owner	
6	Site	
7	Instrument	Rochester ECZL400S
8	Author	cmcnicho
9	Solvent	CHLOROFORM-D
10	Temperature	20.3
11	Pulse Sequence	single_pulse.jxp
12	Experiment	1D
13	Probe	2806
14	Number of Scans	16
15	Receiver Gain	62.0
16	Pulse Width	3.7390
17	Presaturation Frequency	
18	Spectrometer Frequency	376.17
19	Spectral Width	75757.6
20	Lowest Frequency	-75495.9
21	Nucleus	Fluorine19
22	Acquired Size	188087
23	Spectral Size	419432
24	Digital Resolution	0.18

(E)-4-(3-(benzo[d][1,3]dioxol-5-yl)acryloyl)-3-methoxyoxazolidin-2-one(2n)

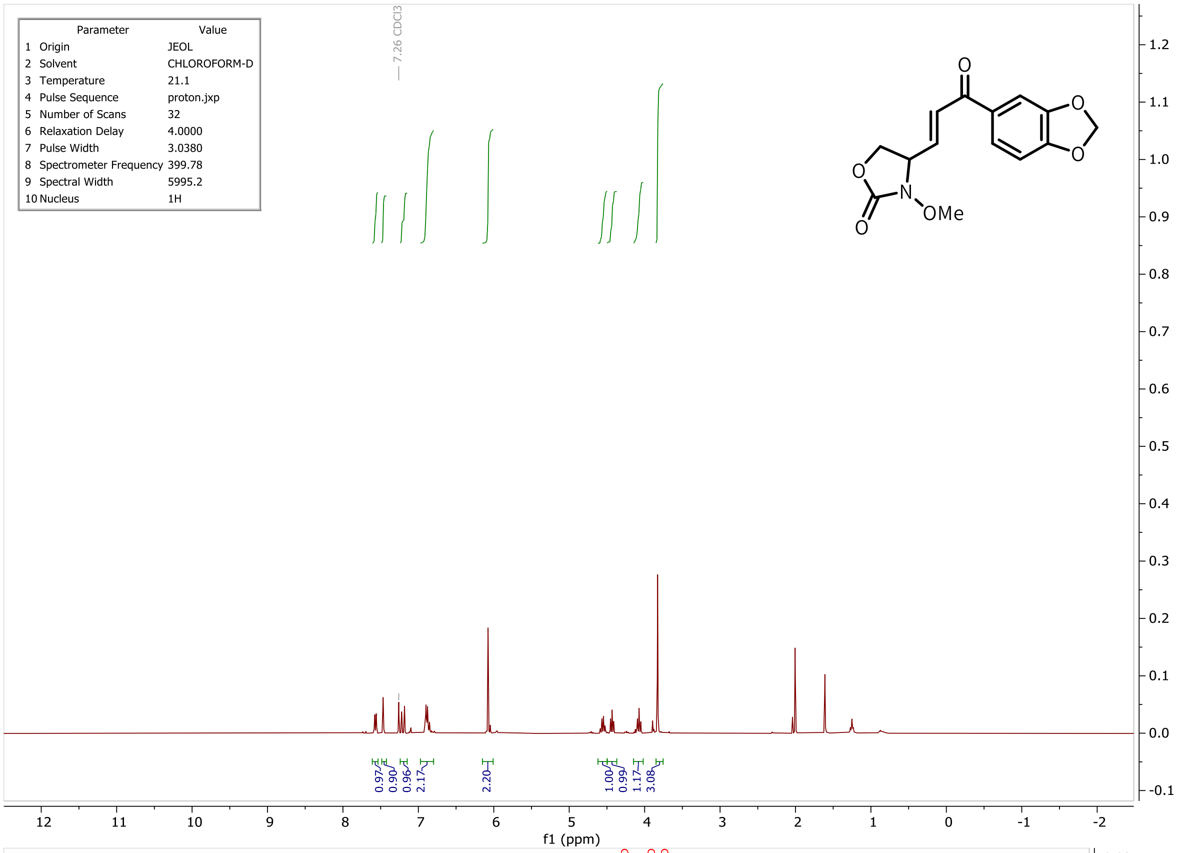

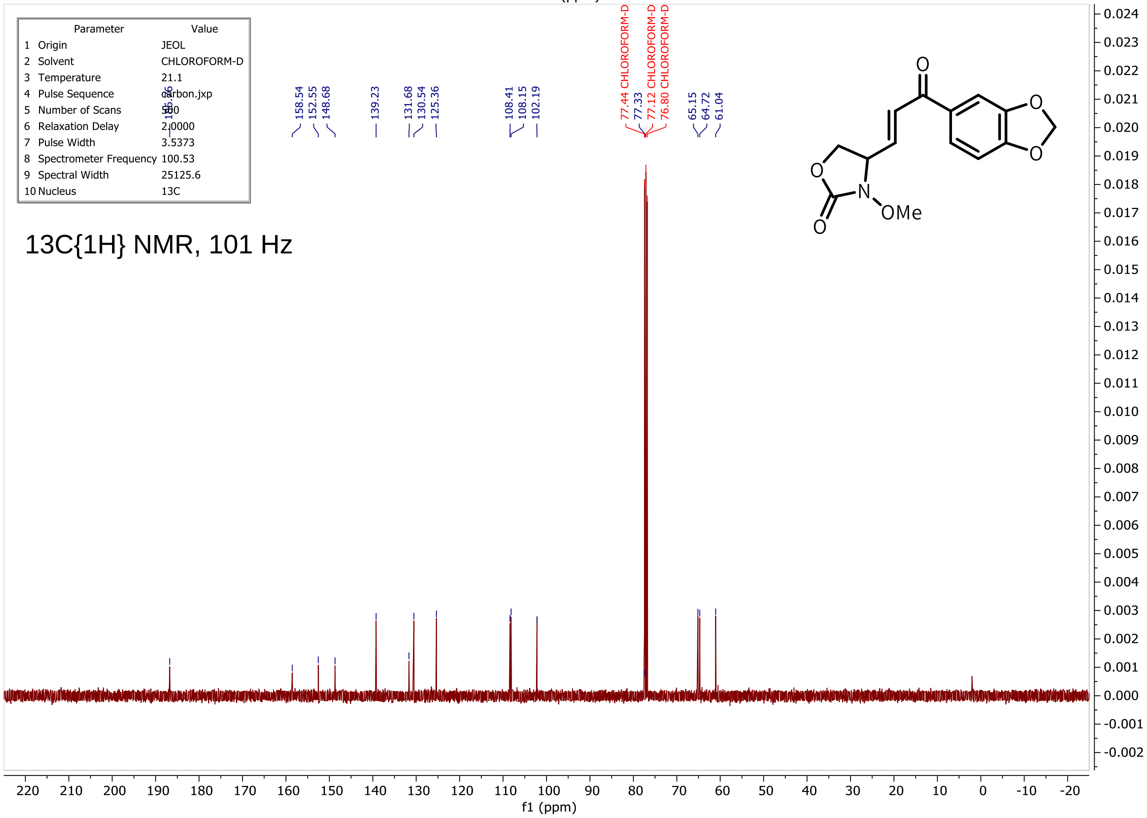

(E)-4-(3-(benzo[d][1,3]dioxol-5-yl)acryloyl)-3-methoxyoxazolidin-2-one(2n')

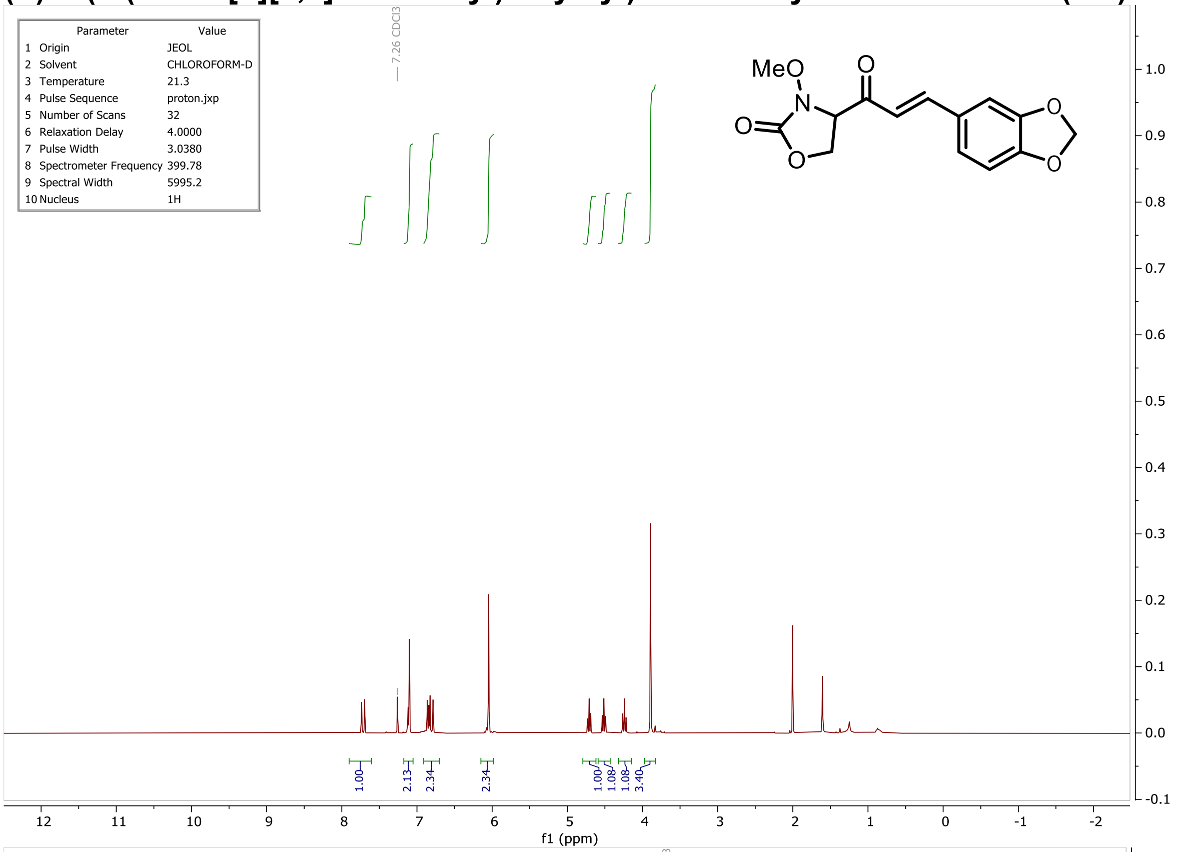

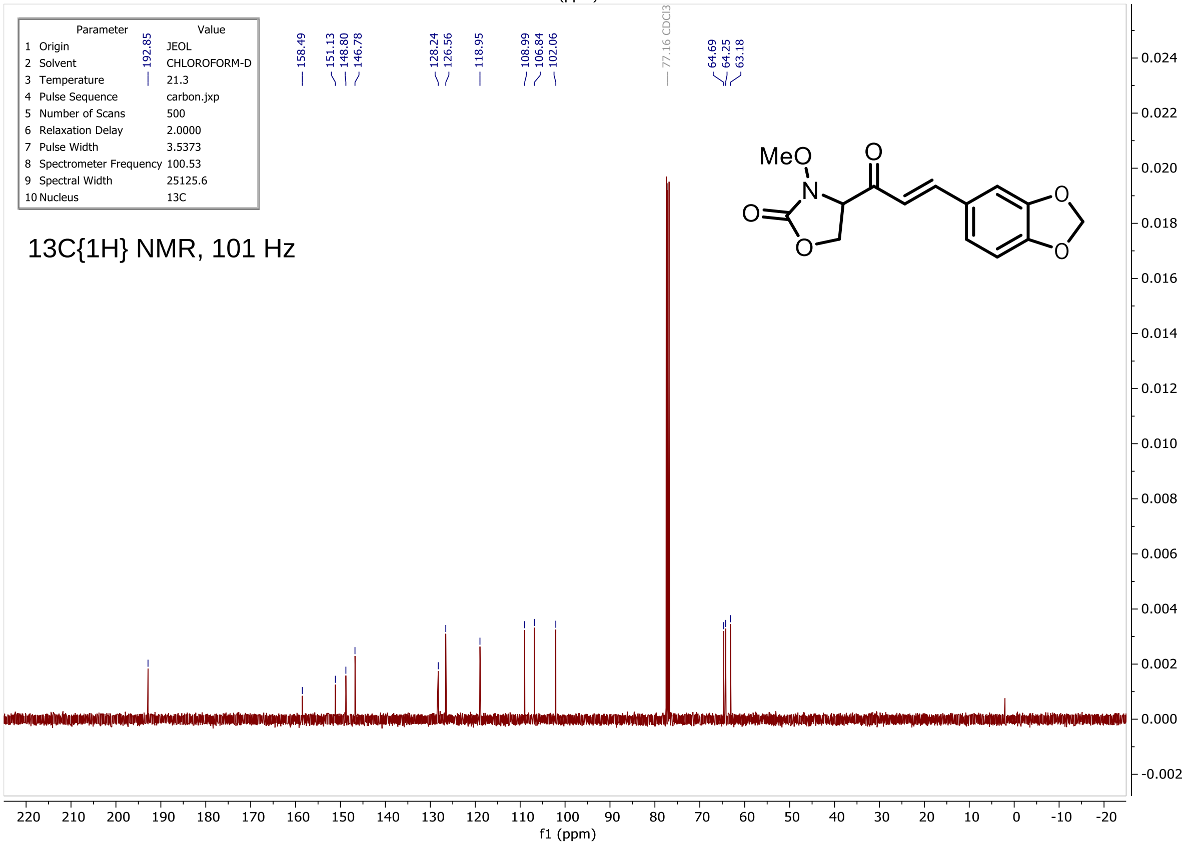

Supplement: Supplementary file 1 [file jo5c02010_si_001.pdf]
